# Preparation of Neutral *trans - cis* [Ru(O_2_CR)_2_P_2_(NN)], Cationic [Ru(O_2_CR)P_2_(NN)](O_2_CR) and Pincer [Ru(O_2_CR)(CNN)P_2_] (P = PPh_3_, P_2_ = diphosphine) Carboxylate Complexes and their Application in the Catalytic Carbonyl Compounds Reduction

**DOI:** 10.1021/acs.organomet.1c00059

**Published:** 2021-04-14

**Authors:** Salvatore Baldino, Steven Giboulot, Denise Lovison, Hans Günter Nedden, Alexander Pöthig, Antonio Zanotti-Gerosa, Daniele Zuccaccia, Maurizio Ballico, Walter Baratta

**Affiliations:** † Dipartimento DI4A, Università di Udine, Via del Cotonificio 108, I-33100 Udine, Italy; ‡ Dipartimento di Chimica, Università di Torino, Via Pietro Giuria, 7, I-10125 Torino, Italy; § Department of Chemistry & Catalysis Research Center, TUM, Lichtenbergstraße 4, 85747 Garching b. München, Germany; ∥ Johnson Matthey, 28 Cambridge Science Park, Milton Road, Cambridge CB4 0FP, United Kingdom

## Abstract

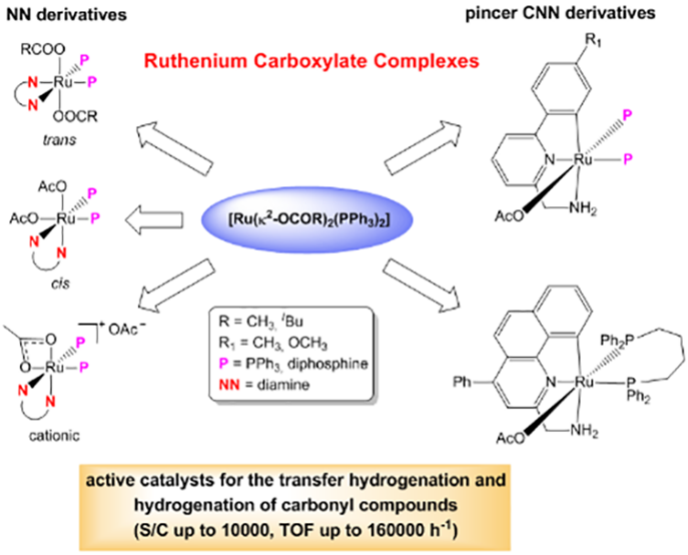

The diacetate complexes *trans*-[Ru(κ^1^-OAc)_2_(PPh_3_)_2_(NN)] (NN = ethylenediamine (en) (**1**), 2-(aminomethyl)pyridine (ampy) (**2**), 2-(aminomethyl)pyrimidine (ampyrim) (**3**)) have been isolated in 76–88% yield by reaction of [Ru(κ^2^-OAc)_2_(PPh_3_)_2_] with the corresponding nitrogen ligands. The ampy-type derivatives **2** and **3** undergo isomerization to the thermodynamically most stable cationic complexes [Ru(κ^1^-OAc)(PPh_3_)_2_(NN)]OAc (**2a** and **3a**) and *cis*-[Ru(κ^1^-OAc)_2_(PPh_3_)_2_(NN)] (**2b** and **3b**) in methanol at RT. The *trans*-[Ru(κ^1^-OAc)_2_(P_2_)_2_] (P_2_ = dppm (**4**), dppe (**5**)) compounds have been synthesized from [Ru(κ^2^-OAc)_2_(PPh_3_)_2_] by reaction with the suitable diphosphine in toluene at 95 °C. The complex *cis*-[Ru(κ^1^-OAc)_2_(dppm)(ampy)](**6**) has been obtained from [Ru(κ^2^-OAc)_2_(PPh_3_)_2_] and dppm in toluene at reflux and reaction with ampy. The derivatives *trans*-[Ru(κ^1^-OAc)_2_P_2_(NN)] (**7**–**16**; NN = en, ampy, ampyrim, 8-aminoquinoline; P_2_ = dppp, dppb, dppf, (*R*)-BINAP) can be easily synthesized from [Ru(κ^2^-OAc)_2_(PPh_3_)_2_] with a diphosphine and treatment with the NN ligands at RT. Alternatively these compounds have been prepared from *trans*-[Ru(OAc)_2_(PPh_3_)_2_(NN)] by reaction with the diphosphine in MEK at 50 °C. The use of (*R*)-BINAP affords *trans*-[Ru(κ^1^-OAc)_2_((*R*)-BINAP)(NN)] (NN = ampy (**11**), ampyrim (**15**)) isolated as single stereoisomers. Treatment of the ampy-type complexes **8**–**15** with methanol at RT leads to isomerization to the cationic derivatives [Ru(κ^2^-OAc)P_2_(NN)]OAc (**8a**–**15a**; NN = ampy, ampyrim; P_2_ = dppp, dppb, dppf, (*R*)-BINAP). Similarly to **2**, the dipivalate *trans*-[Ru(κ^1^-OPiv)_2_(PPh_3_)_2_(ampy)] (**18**) is prepared from [Ru(κ^2^-OPiv)_2_(PPh_3_)_2_] (**17**) and ampy in CHCl_3_. The pincer acetate [Ru(κ^1^-OAc)(CNN^OMe^)(PPh_3_)_2_] (**19**) has been synthesized from [Ru(κ^2^-OAc)_2_(PPh_3_)_2_] and HCNN^OMe^ ligand in 2-propanol with NEt_3_ at reflux. In addition, the dppb pincer complexes [Ru(κ^1^-OAc)(CNN)(dppb)] (CNN = AMTP (**20**), AMBQ^Ph^ (**21**)) have been obtained from [Ru(κ^2^-OAc)_2_(PPh_3_)_2_], dppb, and HAMTP or HAMBQ^Ph^ with NEt_3_, respectively. The acetate NN and pincer complexes are active in transfer hydrogenation with 2-propanol and hydrogenation with H_2_ of carbonyl compounds at S/C values of up to 10000 and with TOF values of up to 160000 h^–1^.

## Introduction

The ever-increasing need to produce valuable organic compounds by industry requires the development of new and more efficient homogeneous transition-metal catalysts. Selective transformations can be achieved through an appropriate choice of ligands at the metal, leading to well-designed catalysts characterized by high productivity. Polydentate nitrogen and phosphine ligands have been extensively employed with the aim to obtain robust and catalytically active species. More recently, the use of carboxylates as ancillary ligands has been demonstrated to be particularly promising in many catalytic processes, since carboxylate may play a non-innocent role, acting as a proton acceptor for H–H and C–H splitting reactions^1^ and stabilizing monomeric species on account of the facile switching capability from a mono- to a bidentate mode of coordination. Furthermore, carboxylates are labile ligands that can dissociate easily, allowing a free site for substrate coordination and formation of the catalytically active species. With regard to ruthenium, which has been widely employed in homogeneous catalysis for its high performance and versatility,^2^ it is worth mentioning that ruthenium carboxylates have been shown to catalyze the hydrogenation (HY) of olefins^3^ and carbonyl compounds.^4^ These types of complexes can also promote alcohol dehydrogenation^5^ and the cycloisomerization of alkynols to five- to seven-membered endocyclic enol ethers.^6^ Ruthenium carboxylate catalysts have been found to activate C–H bonds,^7^ promote functionalization reactions,^8^ efficiently direct C–H/C–O bond arylations with phenols in water,^9^ and react with aldehydes.^10^ Ruthenium phosphine carboxylate complexes have been reported to catalyze the hydrogenation of carboxylic acids and their derivatives to alcohols,^11^ while the employment of BINAP-Ru(II) dicarboxylates^12^ afforded the asymmetric hydrogenation of unsaturated carboxylic acids to the corresponding saturated products.^13^ Furthermore, [Ru(O_2_CR)_2_(CO)_2_(PPh_3_)_2_] (R = CH_2_OCH_3_, *i*Pr, *t*Bu, 2-*c*C_4_H_3_O, Ph) were successfully applied as catalysts in the addition of carboxylic acids to propargylic alcohols to give the corresponding β-oxo esters used in the pharma industry.^14^ Among organic transformations entailing ruthenium catalysts, the reduction of carbonyl compounds via HY^15^ and transfer hydrogenation (TH)^16^ are environmentally benign methods and core processes accepted by the industry for the synthesis of alcohols. Several highly efficient ruthenium catalysts have been developed for both TH and HY, namely [RuCl(η^6^-arene)(TsDPEN)]^12,17^ and *trans*-[RuCl_2_P_2_(diamine)] (P_2_ = diphosphine) complexes, which represent a milestone for these types of catalytic processes.^18^ The employment of the ampy^12^ ligand in place of diamines has resulted in the isolation of *cis*-[RuCl_2_P_2_(ampy)] derivatives that show high catalytic activity for enantioselective TH and HY.^19^ In addition, the related pincer CNN complexes [RuCl(CNN)P_2_], containing functionalized ampy ligands, have proved to be exceptionally productive catalysts for TH and HY, including those of biomass-derived carbonyl compounds.^20^ The replacement of the chloride in *trans*-[RuCl_2_P_2_(diamine)] with sterically hindered carboxylates as anionic ligands has resulted in the highly efficient catalysts [Ru(OCOR)_2_P_2_(en)]^12^ (P_2_ = dppe, xantphos;^12^ R = ^***t***^Bu, Ph, 1-adamantyl) for the selective HY of aldehydes under base-free or acidic conditions.^21^

During our studies aiming to expand the use of ruthenium carboxylates in catalysis, we have found that the cationic monocarbonyl derivatives [RuX(CO)P_2_(NN)]X^22^ (X = Cl, OAc; NN = en, ampy: P_2_ = dppb, dppf),^12^ the trifluoroacetate [Ru(OCOCF_3_)_2_(dppb)(XCH_2_CH_2_X)]^23^ (X = NH_2_, OH) derivatives, and the mixed acetate acetylacetonate complex [Ru(OAc)(acac)(dppb)(ampy)]^24^ have been proven to be highly active catalysts in the TH and HY reductions. The pincer CNN ruthenium acetate complex [Ru(OAc)(AMTP)(dppb)]^12^ has shown the highest activity in TH with a TOF value ot up to 3.8 × 10^6^ h^–1^, consistent with the easier substitution of the carboxylate vs Cl in protic media.^25^ Acetate ruthenium compounds in combination with carbene ligands, namely [RuBr(OAc)(PPh_3_)(P-aNHC)] and [Ru(OAc)(P-aNHC)_2_]Br (P-aNHC = phosphine-abnormal-NHC ligands), have displayed high rates and productivities in TH and in fast Oppenauer-type oxidation reactions (TOFs of up to 600000 h^–1^).^26^ With regard to other applications, ruthenium carboxylate complexes have been described as efficient photosensitizers for TiO_2_ semiconductor solar cells.^27^ New anticancer agents have been prepared by employing ruthenium carboxylate complexes with the aim of obtaining compounds with good solubility in the culture medium.^28^ We have recently reported the synthesis of a new class of cationic carboxylate ruthenium complexes, [Ru(κ^1^-OCOR)(CO)(dppb)(phen)](OCOR)^12^ (R = Me, ^*t*^Bu), that display high cytotoxic activity against anaplastic thyroid cancer cell lines, with EC_50_ values much lower than that of cisplatin, leading to an increment of apoptosis and decrease in cancer cell aggressiveness.^29^

This paper discloses a convenient procedure for the preparation of a series of neutral *trans*/*cis* and cationic carboxylate ruthenium complexes containing bidentate nitrogen and phosphine ligands through straightforward syntheses by starting from the [Ru(κ^2^-OCOR)_2_(PPh_3_)_2_] (R = Me, ^*t*^Bu) precursors. Pincer CNN acetate complexes have also been easily obtained through this synthetic route. The carboxylate ruthenium complexes show activity in TH and HY reactions, allowing the reduction of carbonyl compounds at S/C values of up to 10000 and TOF values of up to 160000 h^–1^.

## Results and Discussion

### Synthesis of Diacetate Ruthenium Complexes with PPh_3_ and NN Ligands

**Scheme 1:**
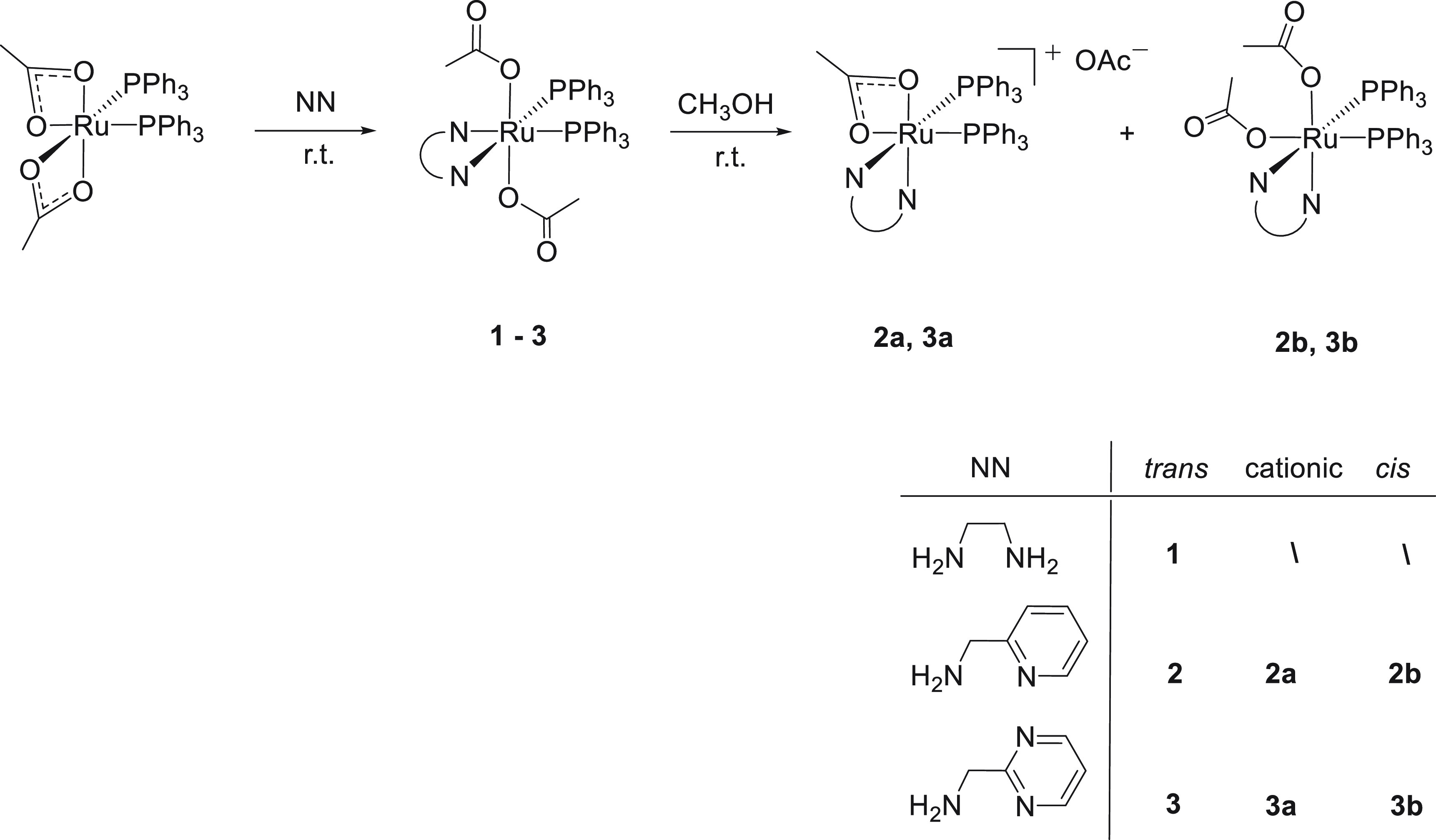
Synthesis of Diacetate Ruthenium Complexes with PPh_3_ and NN Ligands

Treatment of [Ru(κ^2^-OAc)_2_(PPh_3_)_2_] with 1 equiv of en in methyl ethyl ketone (MEK) at room temperature for 45 min affords the complex *trans*,*cis*-[Ru(κ^1^-OAc)_2_(PPh_3_)_2_(en)] (**1**), isolated in 84% yield (Scheme 1).

The ^1^H NMR spectrum of **1** in CDCl_3_ displays two broad singlets at δ 5.31 and 2.67 for the amino and the methylene groups of the en ligand, respectively, with a singlet at δ 1.67 for the methyl acetate. In a fashion similar to that for **1**, the derivative *trans*,*cis*-[Ru(κ^1^-OAc)_2_(PPh_3_)_2_(ampy)] (**2**) is synthesized in high yield (85%) by the reaction of [Ru(κ^2^-OAc)_2_(PPh_3_)_2_] with ampy at room temperature in MEK or dichloromethane (Scheme 1 and methods 1 and 2 in the Experimental Section). Alternatively, **2** has been obtained in 76% yield through a one-pot reaction from [RuCl_2_(PPh_3_)_3_], NaOAc and ampy in acetone via the intermediate [Ru(κ^2^-OAc)_2_(PPh_3_)_2_] (method 3). The ^31^P{^1^H} NMR spectrum of **2** in CD_2_Cl_2_ exhibits two doublets at δ 44.6 and 39.4 with a ^2^*J*(P,P) value of 31.3 Hz, whereas the methylene protons of the ampy appear in the ^1^H NMR spectrum as a broad multiplet at δ 4.18 and the NH_2_ signal is at δ 6.70. This downfield chemical shift is consistent with the presence of a NH···O=C hydrogen-bond interaction of the NH_2_ protons with the two acetate ligands, in contrast with the related complexes *trans*-[Ru(κ^1^-OAc)_2_P_2_(ampy)] (P_2_ = DiPPF, DCyPF)^12^ containing a bulky diphosphine,^30^ in which only one NH interacts with an acetate group.^31^

Recently, we reported that *trans*-[Ru(κ^1^-OAc)_2_(D*i*PPF)_2_(NN)] derivatives, displaying the bulky diphosphine D*i*PPF, are quickly obtained from [Ru(κ^2^-OAc)_2_(D*i*PPF)] and NN (en, ampy) at low temperature.^31^ While the en complex is thermally stable, the ampy compound undergoes rapid isomerization at room temperature to the thermodynamically most stable cationic and *cis* complexes in methanol. Accordingly, dissolution of **2** in methanol at RT for 24 h afforded a mixture of the cationic *cis*-[Ru(κ^2^-OAc)(PPh_3_)_2_(ampy)]OAc (**2a**) and *cis*,*cis*-[Ru(κ^1^-OAc)_2_(PPh_3_)_2_(ampy)] (**2b**) in a 3:2 molar ratio (method 1), isolated in 85% yield (Scheme 1). Alternatively, the same mixture is formed from [Ru(κ^1^-OAc)_2_(PPh_3_)_2_] and ampy in methanol at RT and was isolated in 83% yield (method 2). Attempts to isomerize **2** to **2a**,**b** in toluene at 100 °C failed, leading to decomposition with release of PPh_3_, as inferred from ^31^P{^1^H} NMR analysis. The ^31^P{^1^H} NMR spectrum of **2a**,**b** in CD_3_OD displays two doublets at δ 60.2 and 47.1 (^2^*J*(P,P) = 32.6 Hz) for the cationic complex **2a** and two doublets at δ 65.0 and 49.0 (^2^*J*(P,P) = 29.3 Hz) for the *cis* isomer **2b**. The doublet at δ_H_ 7.98 (^3^*J*(H,H) = 5.7 Hz) and the multiplet a δ_H_ 8.20 are for the *ortho* pyridine signals of **2a** and **2b**, respectively. The diastereotopic methylene protons of the ampy ligand appear as two doublets at δ 4.10 and 3.92 (d, ^2^*J*(H,H) = 16.1 Hz) for the cationic complex **2a**, while the *cis* derivative **2b** displays these signals at δ 4.48 and 4.42. Control ^1^H NMR experiments show that adding sodium acetate (1.0 and 3.5 equiv) to the **2a**,**b** mixture in CD_3_OD showed a progressive increase in the signal at δ 1.93 attributed to the free acetate of the cationic derivative **2a**, confirming the proposed structure (see Figure S10 in the Supporting Information), as observed for [Ru(κ^2^-OAc)(PPh_3_)(NN)(CO)]OAc (NN = en, ampy).^22^ Finally, in the ^13^C{^1^H} NMR spectrum the carbonyl acetate carbon atoms of the cationic **2a** are at δ 190.3 and 180.3, while the acetate resonances of the *cis* derivative **2b** are at δ 191.7 and 190.4.

The reaction of [Ru(κ^2^-OAc)_2_(PPh_3_)_2_] with ampyrim^12^ leads to species similar to those observed with ampy. Thus, the complex *trans*,*cis*-[Ru(κ^1^-OAc)_2_(PPh_3_)_2_(ampyrim)] (**3**) is quickly obtained from [Ru(κ^2^-OAc)_2_(PPh_3_)_2_] and ampyrim in MEK at room temperature and isolated in 88% yield (Scheme 1). Complex **3** isomerizes in methanol at RT within 48 h, leading to a 2:1 mixture of the cationic complexes *cis*-[Ru(κ^2^-OAc)(PPh_3_)_2_(ampyrim)]OAc (**3a**) and *cis*,*cis*-[Ru(κ^1^-OAc)_2_(PPh_3_)_2_(ampyrim)] (**3b**), isolated in 78% yield (Scheme 1). The ^31^P{^1^H} NMR spectroscopic data of **3a**,**b** resemble those of the analogue ampy derivatives **2a**,**b**, with two doublets at δ 58.8 and 47.7 with ^2^*J*(P,P) = 32.8 Hz for **3a** and at δ 64.3 and 49.0 with ^2^*J*(P,P) = 28.0 Hz for **3b**.

### Synthesis of Diacetate Ruthenium Complexes with Diphosphines and NN Ligands

The synthesis of Ru acetate complexes with the ligands dppm and dppe^12^ has been reported by Wong et al. by starting from [Ru(κ^2^-OAc)_2_(PPh_3_)_2_] and the diphosphines in toluene at reflux for 12 h. With dppm the cationic [Ru(κ^2^-OAc)(dppm)]OAc was isolated, whereas with dppe a mixture of the three isomers *cis*- and *trans*-[Ru(κ^1^-OAc)_2_(dppe)_2_] and the cationic [Ru(κ^2^-OAc)(dppe)]OAc were formed and were separated by fractional crystallization.^32^ A reexamination of this procedure under milder reaction conditions show that the *trans*-[Ru(κ^1^-OAc)_2_(P_2_)_2_] (P_2_ = dppm (**4**), dppe (**5**)) derivatives (δ_P_ −5.9 and 44.7, respectively) have been obtained as single products in 68% and 71% yields, respectively, by treatment of [Ru(κ^2^-OAc)_2_(PPh_3_)_2_] with 2 equiv of dppm or dppe in toluene at 95 °C for 20 min (eq 1).
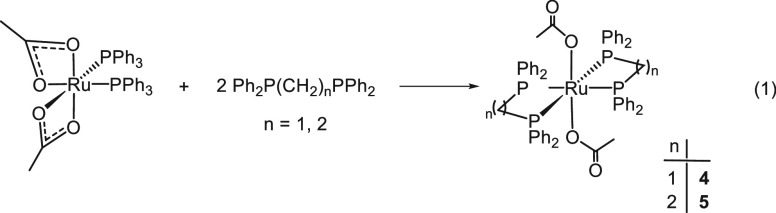
1

**Scheme 2:**
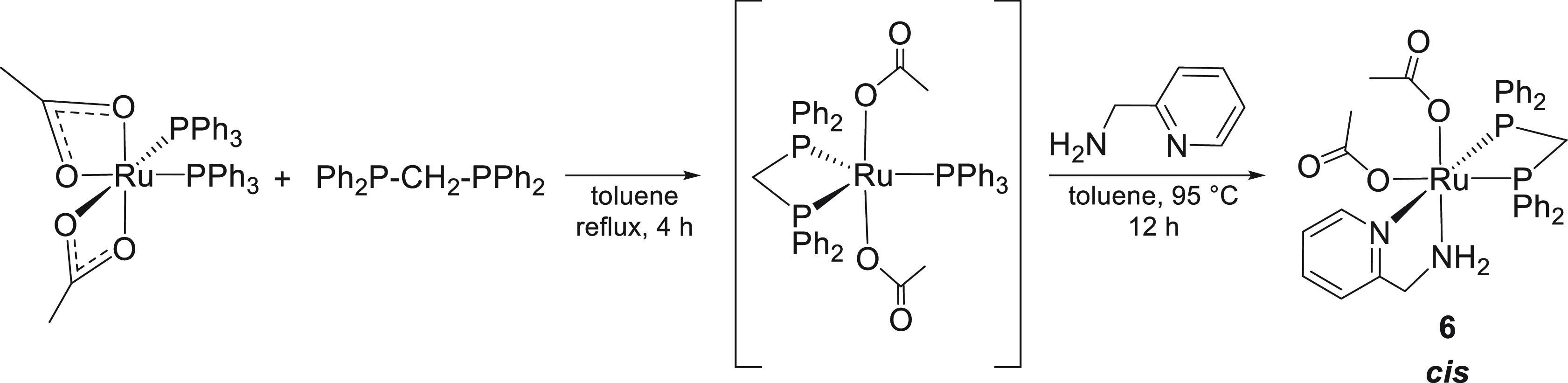
Synthesis of *cis*-[Ru(κ^1^-OAc)_2_(dppm)(ampy)] (**6**)

The reaction of [Ru(κ^2^-OAc)_2_(PPh_3_)_2_] with 1 equiv of dppm in MEK at room temperature afforded a mixture of *trans*-[Ru(κ^1^-OAc)_2_(dppm)_2_] (**4**) and the unreacted precursor. Addition of an excess of ampy (1.2 equiv) at RT results in a partial decoordination of dppm from **4**, with the formation of *trans*-[Ru(κ^1^-OAc)_2_(dppm)(ampy)] in the presence of **4** in a 2:1 molar ratio, as inferred from NMR analysis (see Figures S23 and S24 in the Supporting Information). Interestingly, the thermodynamically most stable isomer, *cis*-[Ru(κ^1^-OAc)_2_(dppm)(ampy)] (**6**), has been isolated in 76% yield from [Ru(κ^2^-OAc)_2_(PPh_3_)_2_] and dppm (1 equiv) in toluene at reflux (4 h), followed by reaction with ampy at 95 °C for 12 h, via the intermediate [Ru(κ^1^-OAc)_2_(dppm)(PPh_3_)] species^32^ (Scheme 2).

**Scheme 3:**
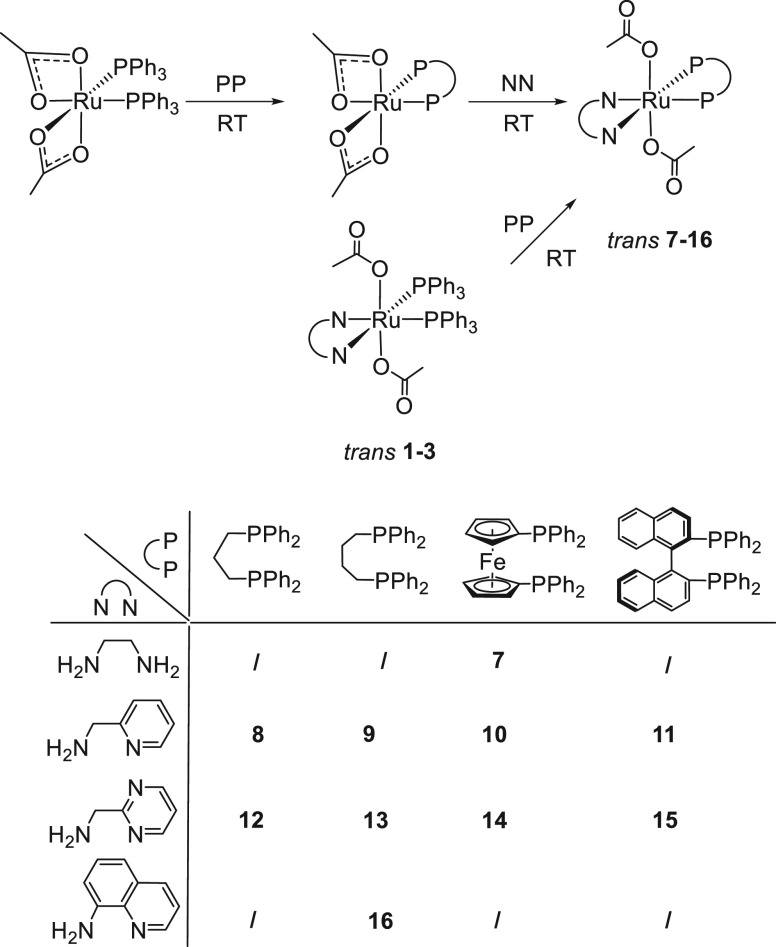
Synthesis of Neutral *trans*-[Ru(κ^1^-OAc)_2_P_2_(NN)] (P_2_ = Diphosphine) Complexes

The ^31^P{^1^H} NMR spectrum of **6** in CDCl_3_ displays two doublets at δ 23.4 and 7.9 with ^2^*J*(P,P) = 94.4 Hz, whereas the methylene protons of the ampy ligand give a doublet of doublets at δ_H_ 3.58 (^2^*J*(H,H) = 16.1 Hz and ^3^*J*(H,H) = 5.0 Hz) and a multiplet at δ_H_ 3.32. A ^15^N–^1^H HSQC 2D NMR analysis reveals that the NH_2_ signals are at δ 9.74 and 1.13 ppm, consistent with the presence of one NH···O hydrogen bond interaction with one acetate. The ^1^H NMR spectrum of **6** in CD_3_OD shows two resonances at δ 2.03 and 1.66 for the methyl groups, indicating that the OAc ligands are coordinated, as was also confirmed by addition of NaOAc (1.0–3.5 equiv) to **6** (δ_H_ 1.92 for the free OAc) (see Figure S27 in the Supporting Information). Attempts to isolate the analogous dppe derivative by the reaction of [Ru(κ^2^-OAc)_2_(PPh_3_)_2_] with dppe in toluene and treatment with ampy failed, resulting in the formation of two [Ru(OAc)_2_(dppe)(ampy)] species in the presence of uncharacterized complexes (see Figures S31 and S32 in the Supporting Information). The employment of diphosphines with a longer backbone leads to the isolation of *trans* diphosphine/NN derivatives at room temperature. The reaction of [Ru(κ^2^-OAc)_2_(PPh_3_)_2_] with dppf in CH_2_Cl_2_ at RT for 1 h, followed by reaction with en for 30 min, affords the complex *trans*-[Ru(κ^1^-OAc)_2_(dppf)(en)] (**7**), isolated in 90% yield (Scheme 3).

**Figure 1:**
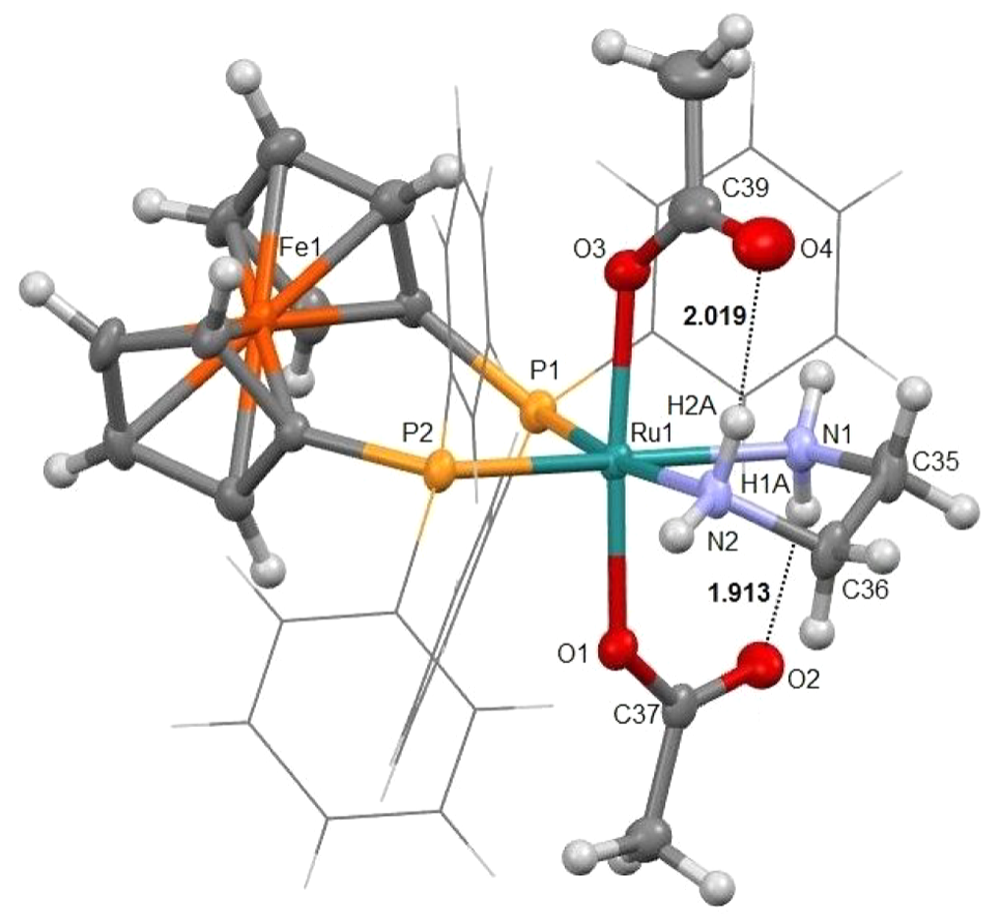
ORTEP style plot of compound **7** in the solid state (CCDC 2058063). Ellipsoids are drawn at the 50% probability level. The phenyl groups are simplified as wireframes for clarity (as well as disorder of one phenyl group is not shown). Selected bond lengths (Å) and angles (deg): Ru1–O1 2.109(3), Ru1–O3 2.118(3), Ru1–N1 2.164(4), Ru1–N2 2.155(3), Ru1–P1 2.2934(11), Ru1–P2 2.2816(11), O1–Ru1–O3 174.90(10), O1–Ru1–N2 87.73(13), O3–Ru1–N2 92.89(13), O1–Ru1–N1 89.91(13), O3–Ru1–N1 85.28(13), N2–Ru1–N1 77.99(13), O1–Ru1–P2 92.37(8), O3–Ru1–P2 92.69(8), N2–Ru1–P2 89.74(9), N1–Ru1–P2 167.42(10), N2–Ru1–P2 89.74(9), N1–Ru1–P2 167.42(10), O1–Ru1–P1 95.44(8), O3–Ru1–P1 83.23(8), N2–Ru1–P1 171.04(10), N1–Ru1–P1 93.61(10), P2–Ru1–P1 98.48(4). Hydrogen-bond distances measured for O2···H1A and O4···H2A are 1.913 and 2.019 Å, respectively.

An X-ray diffraction experiment carried out for **7** shows that this complex crystallizes in a distorted-octahedral geometry with two *trans* acetate groups (Figure 1).

Complex **7** displays Ru–O (2.109(3), 2.118(3) Å) distances in line with the data reported for analogous monodentate diacetate ruthenium complexes,^32,33^ with the Ru–N (2.164(4), 2.155(3) Å) distances being slightly shorter in comparison to those of the related dichloride compound *trans*-[RuCl_2_(dppf)(en)] (Ru–N 2.167(3), 2.171(3) Å)) and consistent with the strong *trans* influence exerted by the diphosphine.^32,34^ The O1–Ru1–O3 angle is almost linear (174.90(10)°) and is greater with respect to that of the analogous chloride compound (Cl–Ru–Cl angle of 166.31(4)°). The solid-state study of **7** also revealed the presence of intramolecular hydrogen-bond interactions between the C=O acetate oxygen atoms with the axial N–H protons of the en ligand with O···H distances of 1.913 and 2.019 Å.^23,33^ The ^1^H NMR spectrum of **7** in solution (CD_2_Cl_2_) displays one triplet at δ 2.64 for the two CH_2_N groups and one broad signal for the four NH hydrogens, shifted to low field at δ 4.92, consistent with a hydrogen-bond interaction with the acetate. The ampy derivative *trans*-[Ru(κ^1^-OAc)_2_(dppp)(ampy)] (**8**) (85% yield) has been prepared from [Ru(κ^2^-OAc)_2_(PPh_3_)_2_] and dppp^12^ in CH_2_Cl_2_, followed by treatment with ampy at RT (Scheme 3, method 1). Alternatively, **8** (93% yield) has been obtained in acetone (method 2) and also by reaction of **2** with dppp in MEK (50 °C, 20 h), by PPh_3_ substitution (61% yield) (method 3). The ^31^P{^1^H} NMR spectrum of **8** displays two doublets at δ 47.8 and 33.1 with ^2^*J*(P,P) = 49.l Hz, while the NH_2_ protons give a broad singlet at δ_H_ 6.27, indicating a NH···O hydrogen bond. The diacetate derivatives *trans*-[Ru(κ^1^-OAc)_2_(dppb)(ampy)] (**9**) and *trans*-[Ru(κ^1^-OAc)_2_(dppf)(ampy)] (**10**) have been synthesized in 61–88% yields by the reaction of Ru(κ^2^-OAc)_2_(PPh_3_)_2_] with the corresponding diphosphine (dppb, dppf) and ampy in CH_2_Cl_2_ or acetone, following the procedure described for **8**. Complexes **9** and **10** have also been prepared by starting from **2** and the diphosphine dppb or dppf, respectively, and isolated in 70–73% yield. Treatment of (*R*)-BINAP with [Ru(κ^2^-OAc)_2_(PPh_3_)_2_] in toluene at reflux for 24 h, followed by reaction with ampy (RT, 1 h), afforded *trans*-[Ru(κ^1^-OAc)_2_((*R*)-BINAP)(ampy)] (**11**) in 59% yield as a single stereoisomer (Scheme 3). The ^31^P{^1^H} NMR spectrum of **11** in CD_2_Cl_2_ exhibits two doublets at δ 54.9 and 40.9 with ^2^*J*(P,P) = 36.9 Hz, whereas the ^1^H NMR spectrum reveals two broad signals for the NH_2_ protons interacting with the acetate ligands at δ 6.91 and 5.06, as inferred from a ^1^H–^15^N HSQC 2D NMR analysis (see Figure S61 in the Supporting Information). In analogy to the ampy complexes, the ampyrim derivatives *trans*-[Ru(κ^1^-OAc)_2_P_2_(ampyrim)] (P_2_ = dppp (**12**), dppb (**13**), dppf (**14**)) have been isolated in good yield (77–85%) by reaction of [Ru(κ^2^-OAc)_2_(PPh_3_)_2_] with diphosphine and ampyrim in CH_2_Cl_2_ at RT (method 1 for **12**–**14**). Alternatively, **12**–**14** have been prepared from **3** and a diphosphine in MEK at 50 °C (57–80% yields) (Scheme 3). Complex **12** shows two doublets at δ_P_ 47.9 and 32.6 with ^2^*J*(P,P) = 50.1 Hz, whereas the multiplets at δ_H_ 8.52 and 8.41 are ascribed to NCH protons of the pyrimidine. The chiral complex *trans*-[Ru(κ^1^-OAc)_2_((*R*)-BINAP)(ampyrim)] (**15**) (63% yield) has been synthesized from [Ru(κ^2^-OAc)_2_(PPh_3_)_2_] and (*R*)-BINAP in toluene at reflux and treatment with ampyrim in acetone at RT. Through a one-pot reaction *trans*-[Ru(κ^1^-OAc)_2_(dppb)(8-aminoquinoline)] (**16**) (90% yield) is obtained from [Ru(κ^2^-OAc)_2_(PPh_3_)_2_], dppb, and 8-aminoquinoline in CH_2_Cl_2_ at RT (Scheme 3). Complex **16** displays two doublets at δ_P_ 50.5 and 37.2 (^2^*J*(P,P) = 36.7 Hz), whereas the multiplet at δ_H_ 9.23 is attributed to the proton in position 2 of the 8-aminoquinoline, shifted to low field in comparison to the free ligand (δ 8.77),^35^ while the broad singlet at δ_H_ 8.24 is ascribed to the amino group.

**Scheme 4:**
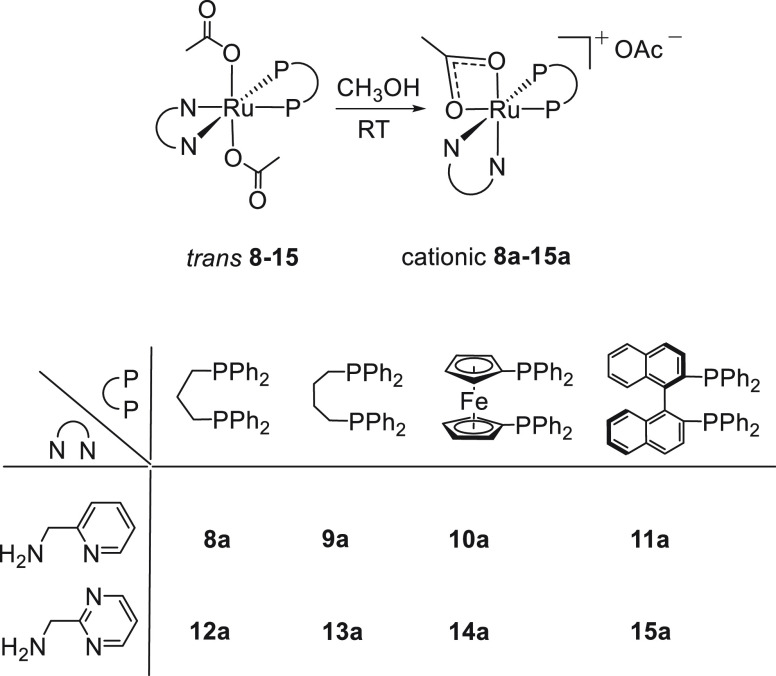
Synthesis of Cationic [Ru(κ^2^-OAc)P_2_(NN)]OAc (P_2_ = Diphosphine) Complexes

The *trans* acetate derivatives *trans*-[Ru(κ^1^-OAc)_2_P_2_(NN)], bearing ampy-type ligands, isomerize to the thermodynamically most stable cationic complexes [Ru(κ^2^-OAc)P_2_(NN)]OAc in methanol, without formation of the neutral *cis* derivative. Thus, [Ru(κ^2^-OAc)(dppp)(ampy)]OAc (**8a**) is obtained in 90% yield by dissolution of **8** in MeOH at room temperature (Scheme 4).

Complex **8a** shows in the ^31^P{^1^H} NMR spectrum in CD_3_OD two doublets at δ 55.2 and 36.7 with ^2^*J*(P,P) = 48.4 Hz. The doublet at δ_H_ 8.09 (^3^*J*(H,H) = 5.7 Hz) is ascribed to the *ortho* pyridine proton, while the two singlets at δ_H_ 1.52 and 1.92 are assigned to the methyl group of the coordinated and free acetates, respectively. The cationic complexes [Ru(κ^2^-OAc)P_2_(ampy)]OAc(P_2_ = dppb (**9a**), dppf (**10a**)) have been prepared from **9** and **10** in methanol at RT and isolated in 95–98% yields (Scheme 4). The ^31^P{^1^H} NMR spectra show the typical doublet patterns for the two complexes at δ_P_ 58.2 and 46.0 (^2^*J*(P,P) = 37.2 Hz) and at δ_P_ 59.9 and 49.5 (^2^*J*(P,P) = 35.4 Hz) for **9a** and **10a**, respectively. The ^1^H NMR spectra of these complexes in CD_3_OD show diastereotopic CH_2_N protons (δ 4.03 and 3.60, with ^2^*J*(H,H) = 16.4 Hz for **9a**) and a singlet at δ 1.92 for the methyl group of the free acetate, as for **8a**. The coordinated acetate ligands of **9a** and **10a** appear as doublets at δ_C_ 189.7 and 190.8, while the free acetate appears at δ_C_ 180.4. In a similar way the cationic ampyrim derivatives [Ru(κ^2^-OAc)P_2_(ampyrim)]OAc (P_2_ = dppp (**12a**), dppb (**13a**), dppf (**14a**)) have been quantitatively isolated (87–98% yield) from the corresponding *trans* isomers in CH_3_OH, the NMR data resembling those of the analogous ampy complexes. The isomerization of the BINAP derivatives **11** and **15** in methanol at RT leads to the cationic [Ru(κ^2^-OAc)((*R*)-BINAP)(NN)]OAc (NN = ampy (**11a**), ampyrim (**15a**)) in 86–96% yields as a mixture of two isomers in about a 2:1 molar ratio for **11a** and 1:1 for **15a**, respectively (Scheme 4), as inferred from NMR measurements in CD_3_OD. Complex **11a** shows a two doublets at δ_P_ 61.1 and 52.4 (^2^*J*(P,P) = 38.8 Hz) for the major isomer and two doublets at δ_P_ 68.3 and 58.1 (^2^*J*(P,P) = 38.7 Hz) for the minor species. Finally, the two singlets at δ_H_ 1.50 and 1.41 are assigned to the methyl acetate ligands of the two isomers, whereas the signal at δ_H_ 1.92 is assigned to the free acetate.

### Synthesis of Dipivalate Ruthenium Complexes with PPh_3_ and ampy

**Scheme 5:**
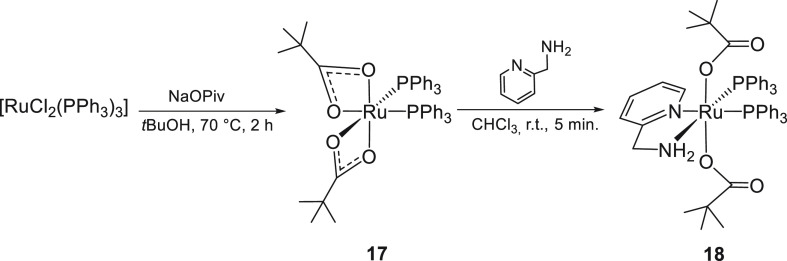
Synthesis of the Pivalate **17** and the ampy Derivative **18**

The precursor [Ru(κ^2^-OPiv)_2_(PPh_3_)_2_] (**17**) has been isolated in 75% yield by treatment of [RuCl_2_(PPh_3_)_3_] with sodium pivalate in *tert*-butyl alcohol at 70 °C by a slight modification of the synthesis reported by Wilkinson^3d^ (Scheme 5).

Reaction of **17** with ampy in chloroform at RT affords the pivalate ruthenium derivative *trans*,*cis*-[Ru(κ^1^-OPiv)_2_(PPh_3_)_2_(ampy)]^12^ (**18**), isolated in 85% yield (Scheme 5). Complex **18** shows two ^31^P{^1^H} NMR doublets at δ 45.8 and 38.5 with ^2^*J*(P,P) = 30.5 Hz. The ampy NCH_2_ protons appear as a broad multiplet at δ_H_ 4.03, with the NH_2_ signal superimposed on those of the aromatic protons, in agreement with a NH···O interaction, whereas the pivalate CO groups give a doublet at δ_C_ 188.2 (^3^*J*(C,P) = 1.3 Hz).

### Synthesis of Pincer CNN Ruthenium Acetate Complexes

**Scheme 6:**
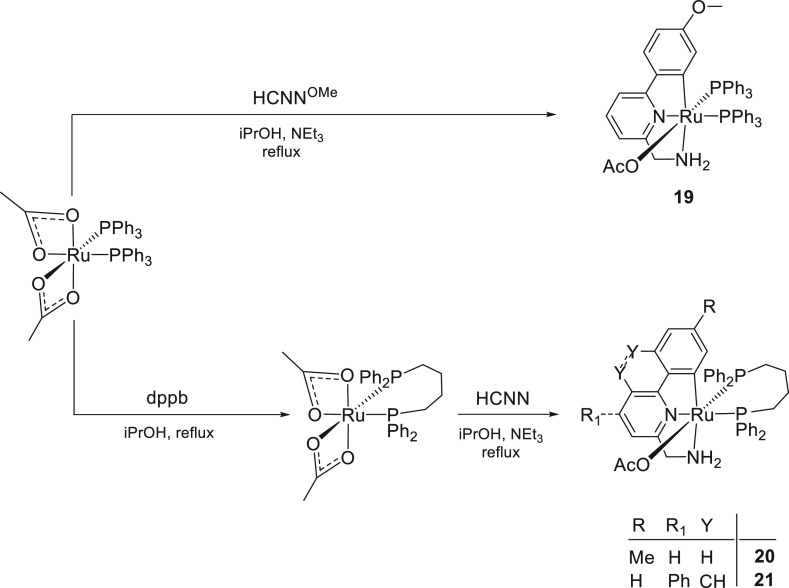
Synthesis of Pincer CNN Ruthenium Acetate Complexes

The pincer acetate complex [Ru(κ^1^-OAc)(CNN^OMe^)(PPh_3_)_2_] (**19**) has been easily prepared in 75% yield by treatment of [Ru(κ^2^-OAc)_2_(PPh_3_)_2_] with the ligand HCNN^OMe^ ^12^ in the presence of the weak base NEt_3_ (10 equiv) in 2-propanol at reflux, through the elimination of acetic acid and cyclometalation (Scheme 6).

The ^31^P{^1^H} NMR spectrum of **19** in CD_2_Cl_2_ shows two doublets at δ 57.2 and 52.9 with ^2^*J*(P,P) = 33.3 Hz, whereas the signals of the NH_2_ group are at δ_H_ 8.86 and 1.92. The low-field resonance is consistent with an intramolecular NH···O hydrogen bond interaction with the acetate ligand (see Figure 117 in the Supporting Information). The singlet at δ 7.68 is attributed to the CH proton close to the ortho-metalated carbon atom, while the diastereotopic CH_2_N gives a doublet of doublets at δ 4.09 (^2^*J*(H,H) = 17.3 Hz and ^3^*J*(H,H) = 6.0 Hz) and a multiplet at δ 3.42. Finally, the cyclometalated carbon appears at δ_C_ 185.5 (dd with ^2^*J*(C,P) = 14.3 and 8.4 Hz), whereas the signal at δ 180.1 can be attributed to the carboxylate CO group. Accordingly, the diphosphine pincer complex [Ru(κ^1^-OAc)(AMTP)(dppb)] (**20**) (85% yield) has been obtained from [Ru(κ^2^-OAc)_2_(dppb)] with HAMTP^12^ and NEt_3_ in 2-propanol at reflux (Scheme 6). Alternatively, **20** can be prepared (46% yield) directly from [Ru(κ^2^-OAc)_2_(PPh_3_)_2_], dppb, and HCNN, through a one-pot reaction. Notably, this new route is more straightforward for preparative scope, with respect to that described, involving the protonation with HOAc of the air- and moisture-sensitive isopropoxide [Ru(O*i*Pr)(AMTP)(dppb)], which equilibrates with the hydride complex [RuH(AMTP)(dppb)].^25^ Similarly to **20**, the benzo[*h*]quinoline CNN derivative [Ru(κ^1^-OAc)(AMBQ^Ph^)(dppb)] (**21**) (59% yield) was obtained from [Ru(κ^2^-OAc)_2_(dppb)], HAMBQ^Ph^,^12^ and NEt_3_ in 2-propanol at reflux (Scheme 6). Conversely, **21** (65% yield) can also be synthesized by a one-pot reaction from [Ru(κ^2^-OAc)_2_(PPh_3_)_2_], dppb, and HAMBQ^Ph^. In CD_2_Cl_2_**21** shows two doublets at δ_P_ 59.8 and 44.9 with ^2^*J*(P,P) = 37.9 Hz, while the NH_2_ resonances are at δ_H_ 8.61 and 0.98, consistent with a N–H···O interaction as for **19** and **20**.^25^ Finally, the broad singlet at δ_C_ 180.4 is assigned to the carboxylate, a value close to that of the doublet of doublets at δ_C_ 180.3 with ^2^*J*(C,P) = 16.1 and 8.8 Hz for the Ru–*C* atom.

### Catalytic Reduction of Carbonyl Compounds via TH and HY Reactions

**Scheme 7:**
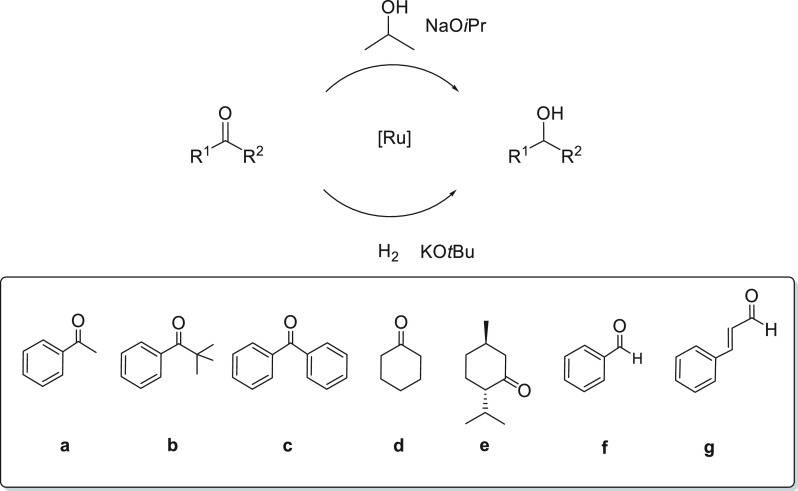
TH and HY of Ketones and Aldehydes Catalyzed by Ruthenium Diacetate Complexes **7**–**11**, **16**, and **21**

The acetate complexes display good to high catalytic activity in the reduction of the C=O bond with 2-propanol in the presence of base and H_2_ under pressure (S/C = 1000–10000) (Scheme 7).

The ethylenediamine dppf derivative **7** displays poor activity in the TH of model substrate acetophenone **a** (0.1 M) in 2-propanol at reflux with NaO*i*Pr (2 mol %), affording 1-phenylethanol (59% of conversion) in 20 h at S/C = 2000 (Table 1, entry 1). Conversely, the related ampy complex **10** shows a significantly higher activity with S/C = 10000, leading to 90% of the alcohol in 3 h (TOF = 28000 h^–1^; entry 7). The ampy compounds **8** and **9**, bearing the dppp and dppb ligands, give 95% and 87% conversion of **a** in 3 and 20 h, respectively, at S/C = 10000 (entries 2 and 3). The use of a higher amount of **9** (S/C = 2000) leads to a dramatic increase in the activity (90% conversion in 10 min, TOF = 11000 h^–1^; entry 4), thus indicating that the dppb derivative undergoes easier deactivation with respect to the ferrocenyl diphosphine complex.

**Table 1:** Catalytic TH of Acetophenone **a** (0.1 M) with Complexes **7**–**11** and **21** (S/C = 2000–10000) and NaO*i*Pr (2 mol %) in 2-Propanol at 82 °C

entry	complex	S/C	time	conversion^a^ (%)	TOF^b^ (h^–1^)
1	**7**	2000	20 h	59	70
2	**8**	10000	4 h	95	5200
3	**9**	10000	20 h	87	1200
4	**9**	2000	10 min	90	11000
5	**9a**	10000	20 h	87	1300
6	**9a**	2000	20 min	93	21000
7	**10**	10000	3 h	90	28000
8	**11**	2000	5 h	94	6400^c^
9	**21**	10000	20 min	97	160000

a Conversions have been determined by GC analyses.

b Turnover frequency (moles of ketone converted to alcohol per mole of catalyst per hour) at 50% conversion.

c 30% ee.

The cationic dppb derivative **9a** shows an activity (87% and 93% conv. at S/C 10000 and 2000) comparable with that of **9**, suggesting that under these catalytic conditions the neutral *trans***9** and the cationic **9a** lead to the same catalytically active species (Table 1, entries 3–6). Use of the (*R*)-BINAP complex **11** (S/C = 2000) affords 94% conversion in 5 h, but with poor enantioselectivity (30% ee; entry 8), while the 8-aminoquinoline derivative **16** gives incomplete reduction (29% conversion in 5 h). Finally, the pincer complex **21** was proven to be highly efficient in the TH of **a**, giving quantitative conversion in 20 min at S/C = 10000 and TOF = 160000 h^–1^ (entry 9), a value comparable to that observed using the corresponding chloride-containing complex.^20b,20c,25^ Catalysts **8**, **10**, and the pincer **21** were tested in the reduction of (bulky) ketones. Thus, **8** and **10** (at S/C = 5000) catalyze the quantitative reduction of *tert*-butyl phenyl ketone **b** to 2,2-dimethyl-1-phenyl-1-propanol in 18 and 20 h, respectively (Table 2, entries 1 and 2), whereas the pincer CNN compound **21** leads to 90% conversion in 18 h, with rates lower than those observed for the TH of **a** (entry 3). Benzophenone **c** was converted to benzhydrol (86 and 78% yields) at S/C = 10000 in 18–20 h with **8** and **10** (entries 4 and 5), whereas with the pincer **21** the reaction is faster with 85% conversion in 2 h (entry 6). Complex **8** catalyzes the TH of **d**, leading to cyclohexanol (98% conversion) at S/C = 5000 in 1.5 h (TOF = 19000 h^–1^, entry 7), while with **10** and **21**, substrate **d** is quantitatively reduced in 10 and 5 min, respectively, with TOF values of 30000 and 150000 h^–1^, which much of the same values obtained for **a** (entries 8 and 9). Complexes **8** and **10** promote the reduction of (−)-menthone **e** to (+)-neomenthol as the main isomer in 58 and 65% yields, in addition to (+)-isomenthol, (−)-menthol, and (+)-neoisomenthol (entries 10 and 11).

**Table 2:** Catalytic TH of Carbonyl Compounds (0.1 M) to Alcohols with Complexes **8**, **10**, and **21** (S/C = 2000–10000) and NaO*i*Pr (2 mol %) in 2-Propanol at 82 °C

entry	substrate	complex	S/C	time	conversion^a^ (%)	TOF^b^ (h^–1^)
1	**b**	**8**	5000	20 h	96	700
2	**b**	**10**	5000	18 h	98	1800
3	**b**	**21**	5000	18 h	99	2500
4	**c**	**8**	10000	20 h	86	1800
5	**c**	**10**	10000	18 h	78	19000
6	**c**	**21**	10000	2 h	85	59000
7	**d**	**8**	5000	1.5 h	98	19000
8	**d**	**10**	5000	10 min	98	30000
9	**d**	**21**	5000	5 min	99	150000
10	**e**	**8**	2000	20 h	86^c^	1100
11	**e**	**10**	2000	18 h	98^d^	7000

a Conversions have been determined by GC analyses.

b Turnover frequency (moles of ketone converted to alcohol per mole of catalyst per hour) at 50% conversion.

c Mixture of diastereoisomeric alcohols: (+)-neomenthol (58%), (+)-isomenthol (11%), (−)-menthol (15%), (+)-neoisomenthol (16%).

d Mixture of diastereoisomeric alcohols: (+)-neomenthol (65%), (+)-isomenthol (11%), (−)-menthol (15%), (+)-neoisomenthol (9%).

A comparison of the activity of the acetate vs the analogous chloride complexes show that the latter undergo a slightly shorter induction period for the formation of the catalytically active species with NaO*i*Pr, whereas the productivity depends on the stereoelectronic properties of the diphosphine, dppf being strongly beneficial to achieving efficient TH.^19a,19d^

Complexes **7**, **9**, **10**, and **19** were also studied in the hydrogenation (HY) of ketones and aldehydes (2 M) at 20–30 atm of H_2_ pressure and 40–70 °C in ethanol or methanol with KO*t*Bu at S/C values of up to 10000 (Scheme 7). The HY reactions have been carried out in a catalyst screening system (eight-vessels Endeavor Biotage system), which allows parallel reactions to be followed.

The en and ampy derivatives **7** and **10** catalyze the quantitative HY of **a** at 40 °C under 30 atm of H_2_ pressure with S/C = 10000 in EtOH (Table 3, entries 1 and 2), whereas the pincer **19** shows low activity (40% conversion) at 70 °C (entry 3). Complex **9** catalyzes the HY of benzaldehyde **f** with low conversion (55%) at 50 °C in MeOH (S/C = 1000) (entry 4). Conversely, **9** promotes the complete HY of *trans*-cinnamaldehyde **g**, affording cinnamyl alcohol (93%) as the main product of the C=O reduction and 3-phenylpropan-1-ol (6%) as a byproduct of the additional C=C HY (entry 5). In addition, the cationic isomer **9a** displays very poor activity in the HY of **f** in MeOH with 12% conversion.

**Table 3:** HY of Carbonyl Compounds (2 M) with Complexes **7**, **9**, **10**, and **19** under H_2_ with KO*t*Bu (2 mol %) after 16 h

entry	complex	substrate	S/C	solvent	*T* (°C)	*p*(H_2_) (atm)	conversion^a^ (%)	alcohol^a^ (%)	byproducts^a^ (%)
1	**7**	**a**	10000	EtOH	40	30	99	99	
2	**10**	**a**	10000	EtOH	40	30	99	99	
3	**19**	**a**	10000	EtOH	70	30	40	40	
4	**9**	**f**	1000	MeOH	50	20	56	55	
5	**9**	**g**	1000	MeOH	50	20	>99	93	6^b^

a The HY experiments were carried out in an eight-vessel Endeavor Biotage system, and the conversions were determined by GC analysis.

b 3-phenylpropan-1-ol.

With regard to the mechanism of the TH and HY reductions, it is likely that the catalytically active mono- or dihydride Ru species are obtained from the ruthenium carboxylate precursors by reaction with alkoxides or H_2_.^36^ For the TH reactions in 2-propanol, the presence of an amino group *cis* to the Ru carboxylate allows the easy formation of Ru–H species via a Ru amide complex and alcohol^37^ or via a Ru amine/alkoxide intermediate.^37−40^ In the HY reactions in basic alcohol media, H_2_ splitting leads to the formation of the ruthenium hydride active species from the carboxylate precursor through a 16-electron Ru amide complex^38b,38c^ or via a Ru amine/alkoxide derivative.^39^ The CNN pincer derivatives undergo elimination of the labile carboxylate group, affording the corresponding hydride species, i.e. [RuH(CNN)(dppb)], a reaction which is facilitated by the *cis* NH_2_ function.^38a,40^ The low activity of the PPh_3_ pincer derivatives in HY may be ascribed to the formation of *trans*-[RuH(CNN^OMe^)(PPh_3_)_2_], in which the H is *trans* to N, with respect to the more active [RuH(CNN)(dppb)], where H is *trans* to P, affording a more hydridic hydride species (see Figure S127 in the Supporting Information).^19d,41^

## Concluding Remarks

In conclusion, we have described the preparation of a class of carboxylate ruthenium complexes containing PPh_3_ and diphosphines in combination with bidentate NN ligands. Neutral *trans* and *cis* complexes of the formula [Ru(OCOR)_2_P_2_(NN)] and the cationic complexes [Ru(O_2_CR)P_2_(NN)](O_2_CR) have been isolated through straightforward syntheses from [Ru(κ^2^-OCOR)_2_(PPh_3_)_2_], a diphosphine, and NN ligands. While the *trans* diamine derivatives [Ru(κ^1^-OAc)_2_P_2_(en)] are thermally stable, the related 2-(aminomethyl)pyridine-type complexes *trans*-[Ru(κ^1^-OAc)_2_P_2_(NN)] easily undergo isomerization at room temperature to the more stable *cis*-[Ru(κ^1^-OAc)_2_P_2_(NN)] and/or the cationic [Ru(κ^1^-OAc)P_2_(NN)]OAc complexes in methanol. In addition, pincer complexes of the formula [Ru(κ^1^-OAc)(CNN)P_2_] have been obtained from [Ru(κ^2^-OAc)_2_(PPh_3_)_2_] via facile cyclometalation of HCNN ligands, and with an additional diphosphine, through a one-pot reaction. The described complexes show good to high catalytic activity in the transfer hydrogenation and hydrogenation of carbonyl compounds. Studies to extend this protocol to the preparation of ruthenium carboxylate complexes with NN and CNN pincer ligands and their application in catalytic organic transformations are currently in progress.

## Experimental Section

All reactions were carried out under an argon atmosphere using standard Schlenk techniques. The solvents were carefully dried by standard methods and distilled under argon before use. The ruthenium complexes [RuCl_2_(PPh_3_)_3_],^42^ [Ru(κ^2^-OAc)_2_(PPh_3_)_2_],^3d^ and [Ru(κ^2^-OAc)_2_(dppb)]^32^ and the ligands HAMTP,^20h^ HCNN^OMe^,^20a^ and HAMBQ^Ph ^^20b^ were prepared according to literature procedures, whereas all other chemicals were purchased from Aldrich and Strem and used without further purification. NMR measurements were recorded on Bruker AC 200 and Avance III HD NMR 400 spectrometers. Chemical shifts (ppm) are relative to TMS for ^1^H and ^13^C{^1^H}, whereas H_3_PO_4_ was used for ^31^P{^1^H}. Infrared measurements were obtained with a Bruker Vector 22 FTIR spectrometer. Elemental analyses (C, H, N) were carried out with a Carlo Erba 1106 analyzer, whereas GC analyses were performed with a Varian CP-3380 gas chromatograph equipped with a 25 m length MEGADEX-ETTBDMS-β chiral column with hydrogen (5 psi) as the carrier gas and a flame ionization detector (FID).

### Synthesis of *trans*,*cis*-[Ru(κ^1^-OAc)_2_(PPh_3_)_2_(en)] (**1**)

[Ru(κ^2^-OAc)_2_(PPh_3_)_2_] (100 mg, 0.134 mmol) and en (9.6 μL, 0.142 mmol, 1.06 equiv) were stirred in MEK (2 mL) at room temperature for 45 min. Addition of *n*-pentane (2 mL) afforded a yellow precipitate that was filtered, washed with *n*-pentane (2 mL), and dried under reduced pressure. Yield: 90 mg (84%). Anal. Calcd for C_42_H_44_N_2_O_4_P_2_Ru (803.84): C, 62.76; H, 5.52; N, 3.49. Found: C, 62.85; H, 5.60; N, 3.51. ^1^H NMR (200.1 MHz, CDCl_3_, 20 °C): δ 7.34–6.97 (m, 30H; aromatic protons), 5.31 (br s, 4H: NH_2_), 2.67 (br s, 4H; CH_2_N), 1.67 (s, 6H; OCOCH_3_). ^31^P{^1^H} NMR (81.0 MHz, CDCl_3_, 20 °C): δ 45.5 (s). ^1^H NMR (400.1 MHz, CD_2_Cl_2_, 25 °C): δ 7.42–7.23 (m, 18H; aromatic protons), 7.16 (t, ^3^*J*(H,H) = 7.4 Hz, 12H; aromatic protons), 5.37 (br s, 4H; NH_2_), 2.70 (m, 4H; NCH_2_CH_2_N), 1.71 (s, 6H; OCOCH_3_). ^13^C{^1^H} NMR (100.6 MHz, CD_2_Cl_2_, 25 °C): δ 182.7 (br s; O*C*OCH_3_), 136.0 (t, ^1^*J*(C,P) = 18.7 Hz; *ipso*-Ph), 135.6 (t, ^1^*J*(C,P) = 18.5 Hz; *ipso*-Ph), 134.2 (t, ^2^*J*(C,P) = 4.8 Hz; *ortho*-Ph), 128.8 (br s; *para*-Ph), 127.5 (t, ^3^*J*(C,P) = 4.4 Hz; *meta*-Ph), 43.9 (br s; N*C*H_2_*C*H_2_N), 25.8 (br s; OCO*C*H_3_). ^31^P{^1^H} NMR (162.0 MHz, CD_2_Cl_2_, 25 °C): δ 44.8 (s).

### Synthesis of *trans*,*cis*-[Ru(κ^1^-OAc)_2_(PPh_3_)_2_(ampy)] (**2**)

#### Method 1

Complex **2** was prepared by following the procedure used for the synthesis of **1**, with ampy (15.0 μL, 0.146 mmol, 1.09 equiv) in place of en. Yield: 113 mg (99%). Anal. Calcd for C_46_H_44_N_2_O_4_P_2_Ru (851.89): C, 64.86; H, 5.21; N, 3.29. Found: C, 64.90; H, 5.30; N, 3.31. ^1^H NMR (200.1 MHz, CD_2_Cl_2_, 20 °C): δ 8.45 (d, ^3^*J*(H,H) = 5.7 Hz, 1H; *ortho-*CH of C_5_H_4_N), 7.57–6.88 (m, 32H; aromatic protons), 6.70 (br d, ^3^*J*(H,H) = 5.4 Hz, 2H; NH_2_), 6.53 (*pseudo*-t, *J*(H,H) = 6.5 Hz, 1H; aromatic proton), 4.18 (m, 2H; CH_2_N), 1.67 (s, 6H; OCOCH_3_). ^13^C{^1^H} NMR (50.3 MHz, CD_2_Cl_2_, 20 °C): δ 180.9 (d, ^3^*J*(C,P) = l.6 Hz; O*C*OCH_3_), 166.5 (dd, ^3^*J*(C,P) = 2.5 Hz, ^3^*J*(C,P) = 1.4 Hz; N*C*CH_2_), 156.7 (d, ^3^*J*(C,P) = 4.0 Hz; N*C*H of C_5_H_4_N), 137.2–119.3 (m; aromatic carbon atoms), 51.6 (dd, ^3^*J*(C,P) = 3.5 Hz, ^3^*J*(C,P) = 2.4 Hz; CH_2_N), 26.1 (s; OCO*C*H_3_). ^31^P{^1^H} NMR (81.0 MHz, CD_2_Cl_2_, 20 °C): δ 44.6 (d, ^2^*J*(P,P) = 31.3 Hz), 39.4 (d, ^2^*J*(P,P) = 31.3 Hz).

#### Method 2

[Ru(κ^1^-OAc)_2_(PPh_3_)_2_] (37 mg, 0.050 mmol) and ampy (6.0 μL, 0.058 mmol, 1.17 equiv) were dissolved in CD_2_Cl_2_ (0.45 mL). After 5 min at room temperature quantitative formation of **2** was observed by NMR analysis.

#### Method 3

[RuCl_2_(PPh_3_)_3_] (450 mg, 0.469 mmol) and NaOAc (385 mg, 4.69 mmol, 10 equiv) were suspended in degassed acetone (5 mL), and the mixture was refluxed for 3 h, affording a bright orange precipitate of [Ru(κ^2^-OAc)_2_(PPh_3_)_2_]. When the reaction mixture was cooled to room temperature, ampy (52 μL, 0.504 mmol, 1.07 equiv) was added and the mixture was stirred for 30 min, leading to a bright yellow precipitate. After the addition of *n*-heptane (8 mL), the solid was filtered, washed with water (3 × 10 mL), 2-propanol (1 mL), and *n*-pentane (3 × 5 mL), and dried under reduced pressure. Yield: 303 mg (76%).

### Synthesis of *cis*-[Ru(κ^2^-OAc)(PPh_3_)_2_(ampy)]OAc (**2a**) and *cis*,*cis*-[Ru(κ^1^-OAc)_2_(PPh_3_)_2_(ampy)] (**2b**)

#### Method 1

Complex **2** (20 mg, 0.023 mmol) was dissolved in CH_3_OH (2 mL), and the orange solution was stirred for 24 h at RT. The solvent was evaporated under reduced pressure, and the residue was dissolved in CH_2_Cl_2_ (0.5 mL). Addition of *n*-pentane (2 mL) gave a yellow-orange precipitate, which was filtered, washed with *n*-pentane (3 × 2 mL), and dried under reduced pressure. The product consists of **2a**,**b** in a 3:2 molar ratio. Yield: 17 mg (85%). Anal. Calcd for C_46_H_44_N_2_O_4_P_2_Ru (851.89): C, 64.86; H, 5.21; N, 3.29. Found: C, 64.80; H, 5.17; N, 3.37. ^1^H NMR (400.1 MHz, CD_3_OD, 25 °C): δ 8.20 (m, 1H; *ortho-*CH of C_5_H_4_N minor isomer), 7.98 (d, ^3^*J*(H,H) = 5.7 Hz, 1H; *ortho-*CH of C_5_H_4_N major isomer), 7.73 (td, ^3^*J*(H,H) = 7.7 Hz, ^4^*J*(H,H) = 1.8 Hz, 1H; *para-*CH of C_5_H_4_N major isomer), 7.70–7.63 (m, 3H; aromatic protons both isomers), 7.62–7.49 (m, 4H; aromatic protons both isomers), 7.48–7.08 (m, 22H; aromatic protons both isomers), 6.96 (t, ^3^*J*(H,H) = 6.4 Hz, 1H; *meta-*CH of C_5_H_4_N major isomer), 6.73 (d, ^3^*J*(H,H) = 6.0 Hz, 1H; *meta-*CH of C_5_H_4_N minor isomer), 5.84 (ddd, ^3^*J*(H,H) = 7.6 Hz, ^3^*J*(H,H) = 5.8 Hz, ^4^*J*(H,H) = 1.6 Hz, 1H; *meta-*CH of C_5_H_4_N minor isomer), 4.48 (d, ^2^*J*(H,H) = 15.5 Hz, 1H; CH_2_N minor isomer), 4.42 (dd, ^2^*J*(H,H) = 15.5 Hz, ^4^*J*(H,P) = 4.6 Hz, 1H; CH_2_N minor isomer), 4.10 (d, ^2^*J*(H,H) = 16.1 Hz, 1H; CH_2_N major isomer), 3.92 (d, ^2^*J*(H,H) = 16.1 Hz, 1H; CH_2_N major isomer), 1.93 (s, 3H; OCOCH_3_ major isomer), 1.70 (s, 3H; OCOCH_3_ minor isomer), 1.36 (s, 3H; OCOCH_3_ minor isomer), 1.15 (s, 3H; OCOCH_3_ major isomer). ^13^C{^1^H} NMR (100.6 MHz, CD_3_OD, 25 °C): δ 191.7 (d, ^2^*J*(C,P) = 2.2 Hz; O*C*OCH_3_ minor isomer), 190.4 (br s; O*C*OCH_3_ minor isomer), 190.3 (br s; O*C*OCH_3_ major isomer), 180.3 (br s; O*C*OCH_3_ major isomer), 165.3 (d, ^3^*J*(C,P) = 1.6 Hz; N*C*CH_2_ minor isomer), 162.1 (d, ^3^*J*(C,P) = 1.4 Hz; N*C*CH_2_ major isomer), 160.7 (d, ^3^*J*(C,P) = 2.3 Hz; N*C*H of C_5_H_4_N minor isomer), 151.4 (br s; N*C*H of C_5_H_4_N major isomer), 138.9–121.7 (m; aromatic carbon atoms both isomers), 53.6 (d, ^3^*J*(C,P) = 2.9 Hz; CH_2_N major isomer), 51.2 (d, 3*J*(C,P) = 2.3 Hz; CH_2_N minor isomer), 24.3 (br s; OCO*C*H_3_ major isomer), 24.2 (m; OCO*C*H_3_ both isomers), 24.0 (d, ^4^*J*(C,P) = 1.5 Hz; OCO*C*H_3_ minor isomer). ^31^P{^1^H} NMR (162.0 MHz, CD_3_OD, 25 °C): δ 65.3 (d, ^2^*J*(P,P) = 29.3 Hz; minor isomer), 60.6 (d, ^2^*J*(P,P) = 32.6 Hz; major isomer), 49.4 (d, ^2^*J*(P,P) = 29.3 Hz; minor isomer), 47.4 (d, ^2^*J*(P,P) = 32.6 Hz; major isomer).

#### Method 2

[Ru(κ^1^-OAc)_2_(PPh_3_)_2_](20 mg, 0.0269 mmol) and ampy (3.0 μL, 0.0291 mmol, 1.08 equiv) were dissolved in CH_3_OH (2 mL), and the mixture was stirred for 36 h at RT. The solvent was evaporated under reduced pressure, and the residue was dissolved in CH_2_Cl_2_ (0.5 mL). Addition of *n*-pentane (2 mL) afforded a yellow-orange precipitate that was filtered, washed with *n*-pentane (3 × 2 mL), and dried under reduced pressure, leading to **2a**,**b** in a 3:2 molar ratio. Yield: 19 mg (83%).

### Synthesis of *trans*,*cis*-[Ru(κ^1^-OAc)_2_(PPh_3_)_2_(ampyrim)] (**3**)

Complex **3** was prepared byfollowing the procedure used for the synthesis of **1** (method 1), with ampyrim^43^ (16.1 μL, 0.168 mmol, 1.25 equiv) in place of en. Yield: 101 mg (88%). Anal. Calcd for C_45_H_43_N_3_O_4_P_2_Ru (852.87): C, 63.37; H, 5.08; N, 4.93. Found: C, 63.45; H, 5.10; N, 4.91. ^1^H NMR (200.1 MHz, CDCl_3_, 20 °C): δ 8.49 (m, 1H; RuNC*H* of C_4_H_3_N_2_), 8.36 (m, 1H; NC*H* of C_4_H_3_N_2_), 7.48–7.06 (m, 24H: aromatic protons), 7.05–6.88 (m, 6H: aromatic protons), 6.48 (*pseudo*-t, *J*(H,H) = 5.1 Hz, 1H: aromatic proton), 6.22 (m, 2H; NH_2_), 4.31 (m, 2H; CH_2_N), 1.71 (s, 6H; OCOCH_3_). ^13^C{^1^H} NMR (50.3 MHz, CDCl_3_, 20 °C): δ 180.9 (br s; O*C*OCH_3_), 176.3 (dd, ^3^*J*(CP) = 3.5 Hz, ^3^*J*(CP) = 1.4 Hz; N*C*CH_2_), 162.6 (d, ^3^*J*(CP) = 3.3 Hz; RuN*C*H of C_4_H_3_N_2_), 155.1 (s; N*C*H of C_4_H_3_N_2_), 136.3–117.6 (m; aromatic carbon atoms), 51.5 (t, ^3^*J*(CP) = 2.4 Hz; CH_2_N), 25.9 (s; OCO*C*H_3_). ^31^P{^1^H} NMR (81.0 MHz, CDCl_3_, 20 °C): δ 43.6 (d, ^2^*J*(P,P) = 32.3 Hz), 39.7 (d, ^2^*J*(P,P) = 32.3 Hz).

### Synthesis of *cis*-[Ru(κ^2^-OAc)(PPh_3_)_2_(ampyrim)]OAc (**3a**) and *cis*,*cis*-[Ru(κ^1^-OAc)_2_(PPh_3_)_2_(ampyrim)] (**3b**)

Complex **3** (27 mg, 0.032 mmol) was dissolved in CH_3_OH (2 mL), and the orange solution was stirred for 48 h at RT. The solvent was evaporated under reduced pressure, and the residue was dissolved in CH_2_Cl_2_ (0.5 mL). Addition of *n*-pentane (2 mL) afforded a yellow-orange precipitate that was filtered, washed with *n*-pentane (3 × 2 mL), and dried under reduced pressure. The product consists of **3a**,**b** in a 2:1 molar ratio. Yield: 21 mg (78%). Anal. Calcd for C_45_H_43_N_3_O_4_P_2_Ru (852.87): C, 63.37; H, 5.08; N, 4.93. Found: C, 63.35; H, 5.14; N, 4.97. ^1^H NMR (400.1 MHz, CD_3_OD, 25 °C): δ 8.82 (d, ^3^*J*(H,H) = 5.0 Hz, 1H; RuNC*H* of C_4_H_3_N_2_ minor isomer), 8.66 (dd, 1H, ^3^*J*(H,H) = 4.9 Hz, ^4^*J*(H,H) = 2.1 Hz; RuNC*H* of C_4_H_3_N_2_ major isomer), 8.25 (dt, ^3^*J*(H,H) = 5.1 Hz, ^4^*J*(H,H) = 2.4 Hz, 1H; NC*H* of C_4_H_3_N_2_ major isomer), 8.21 (dd, ^3^*J*(H,H) = 4.8 Hz, ^4^*J*(H,H) = 2.0 Hz, 1H; NC*H* of C_4_H_3_N_2_ minor isomer), 7.61 (t, ^3^*J*(H,H) = 8.6 Hz, 4H; aromatic protons major isomer), 7.43–7.33 (m, 6H; aromatic protons both isomers), 7.33–7.09 (m, 20H; aromatic protons both isomers), 7.06 (t, ^3^*J*(H,H) = 5.4 Hz, 1H; aromatic proton major isomer), 7.03 (m, 1H; aromatic proton minor isomer), 6.90 (d, ^3^*J*(H,H) = 6.3 Hz, 1H; aromatic proton minor isomer), 5.98 (t, ^3^*J*(H,H) = 5.4 Hz, 1H; aromatic proton minor isomer), 4.54–4.41 (m, 2H; CH_2_N minor isomer), 4.17 (d, ^2^*J*(H,H) = 17.1 Hz, 1H; CH_2_N major isomer), 3.96 (d, ^2^*J*(H,H) = 17.1 Hz, 1H; CH_2_N major isomer), 1.91 (s, 3H; OCOCH_3_ major isomer), 1.75 (s, 3H; OCOCH_3_ minor isomer), 1.36 (s, 3H; OCOCH_3_ minor isomer), 1.18 (s, 3H; OCOCH_3_ major isomer). ^13^C{^1^H} NMR (100.6 MHz, CD_3_OD, 25 °C): δ 192.2 (d, ^2^*J*(C,P) = 2.1 Hz; O*C*OCH_3_ minor isomer), 190.9 (br s; O*C*OCH_3_ minor isomer), 190.8 (br s; O*C*OCH_3_ major isomer), 180.0 (br s; O*C*OCH_3_ major isomer), 174.2 (d, ^3^*J*(C,P) = 1.6 Hz; N*C*CH_2_ minor isomer), 172.2 (d, ^3^*J*(C,P) = 1.5 Hz; N*C*CH_2_ major isomer), 167.0 (d, ^3^*J*(C,P) = 1.5 Hz; RuN*C*H of C_4_H_3_N_2_ minor isomer), 158.7 (s; RuN*C*H of C_4_H_3_N_2_ major isomer), 158.5 (s; N*C*H of C_4_H_3_N_2_ major isomer), 156.4 (s; N*C*H of C_4_H_3_N_2_ minor isomer), 136.4–129.4 (m; aromatic carbon atoms both isomers), 122.4 (d, *J*(C,P) = 1.5 Hz; aromatic carbon atom major isomer), 120.4 (br s; aromatic carbon atom minor isomer), 53.9 (d, ^3^*J*(C,P) = 2.0 Hz; CH_2_N major isomer), 51.7 (d, ^3^*J*(C,P) = 2.2 Hz; CH_2_N minor isomer), 24.3 (d, ^4^*J*(C,P) = 1.4 Hz; OCO*C*H_3_ minor isomer), 24.1 (br s; OCO*C*H_3_ both isomers), 23.9 (d, ^4^*J*(C,P) = 1.3 Hz; OCO*C*H_3_ major isomer). ^31^P{^1^H} NMR (162.0 MHz, CD_3_OD, 25 °C): δ 64.3 (d, ^2^*J*(P,P) = 28.0 Hz; minor isomer), 58.8 (d, ^2^*J*(P,P) = 32.8 Hz; major isomer), 49.0 (d, ^2^*J*(P,P) = 28.0 Hz; minor isomer), 47.7 (d, ^2^*J*(P,P) = 32.8 Hz; major isomer).

### Synthesis of *trans*-[Ru(κ^1^-OAc)_2_(dppm)_2_] (**4**)

Complex **4** was prepared by following a slight modification of a procedure described for the synthesis of the cationic isomer [Ru(κ^2^-OAc)(dppm)_2_]OAc.^32^ [Ru(κ^2^-OAc)_2_(PPh_3_)_2_] (50.0 mg, 0.067 mmol) and dppm (51.9 mg, 0.135 mmol, 2.0 equiv) were stirred in toluene (0.75 mL) at 95 °C for 20 min. The solvent was evaporated under reduced pressure, and the residue was added to *n*-heptane (4 mL). The mixture was stirred for 10 min, giving a suspension, which was filtered; the solid was washed with *n*-heptane (2 × 1 mL) and *n*-pentane (2 × 1 mL) and dried under reduced pressure. Yield: 45 mg (68%). Anal. Calcd for C_54_H_50_O_4_P_4_Ru (987.96): C, 65.65; H, 5.10. Found: C, 65.70; H, 5.15. ^1^H NMR (200.1 MHz, CDCl_3_, 20 °C): δ 7.41–7.03 (m, 40H; aromatic protons), 5.84 (m, 4H; PCH_2_), 0.80 (s, 6H; OCOCH_3_). ^31^P{^1^H} NMR (81.0 MHz, CDCl_3_, 20 °C): δ - 5.9 (s).

### Synthesis of *trans*-[Ru(κ^1^-OAc)_2_(dppe)_2_] (**5**)

Complex **5** was prepared by following the procedure used for the synthesis of **4**, with dppe (53.8 mg, 0.135 mmol, 2.0 equiv) in place of dppm. This method presents some slight modifications in comparison to that already reported for the synthesis of **5**.^32^ Yield: 49 mg (72%). Anal. Calcd for C_56_H_54_O_4_P_4_Ru (1016.01): C, 66.20; H, 5.36. Found: C, 66.26; H, 5.43. ^1^H NMR (200.1 MHz, CDCl_3_, 20 °C): δ 7.56–6.88 (m, 40H; aromatic protons), 3.20 (br m, 8H; PCH_2_CH_2_P), 0.80 (s, 6H; OCOCH_3_). ^31^P{^1^H} NMR (81.0 MHz, CDCl_3_, 20 °C): δ 44.7 (s).

### Synthesis of *cis*-[Ru(κ^1^-OAc)_2_(dppm)(ampy)] (**6**)

[Ru(κ^2^-OAc)_2_(PPh_3_)_2_] (200 mg, 0.269 mmol) and dppm (104 mg, 0.270 mmol, 1.01 equiv) were dissolved in toluene (1 mL), and the mixture was refluxed for 4 h, until the precursor was fully converted to [Ru(κ^1^-OAc)_2_(dppm)(PPh_3_)] as verified by NMR analysis. ampy (31 μL, 0.300 mmol, 1.11 equiv) was added, and the solution was stirred at 95 °C for 14 h. The solvent was evaporated under reduced pressure, and the residue was added to *n*-heptane (6 mL). The suspension was stirred for 10 min, and the solid was filtered, washed with *n*-heptane (2 × 2 mL) and *n*-pentane (2 × 2 mL), and dried under reduced pressure. Yield: 145 mg (76%). Anal. Calcd for C_35_H_36_N_2_O_4_P_2_Ru (711.70): C, 59.07; H, 5.10; N, 3.94. Found C, 59.15; H, 5.18; N, 3.97. ^1^H NMR (200.1 MHz, CDCl_3_, 20 °C): δ 9.74 (m, 1H; NH_2_), 9.59 (m, 1H; *ortho-*CH of C_5_H_4_N), 8.01 (br t, ^3^*J*(H,H) = 8.1 Hz, 2H; aromatic protons), 7.92–6.86 (m, 19H; aromatic protons), 6.70 (t, ^3^*J*(H,H) = 7.5 Hz, 2H; aromatic protons), 5.84 (*pseudo*-q, *J*(H,H) = 13.1 Hz, 1H; PCH_2_), 5.11 (m, 1H; PCH_2_), 3.58 (dd, ^2^*J*(H,H) = 16.1 Hz, ^3^*J*(H,H) = 5.0 Hz, 1H; CH_2_N), 3.32 (m, 1H; CH_2_N), 2.01 (s, 3H; OCOCH_3_), 1.58 (s, 3H; OCOCH_3_), 1.13 (m, 1H; NH_2_). ^31^P{^1^H} NMR (81.0 MHz, CDCl_3_, 20 °C): δ 23.4 (d, ^2^*J*(P,P) = 94.4 Hz), 7.9 (d, ^2^*J*(P,P) = 94.4 Hz). ^1^H NMR (400.1 MHz, CD_3_OD, 20 °C): δ 9.19 (m, 1H; *ortho-*CH of C_5_H_4_N), 7.81 (t, ^3^*J*(H,H) = 15.3 Hz, 2H; aromatic protons), 7.64 (br t, ^3^*J*(H,H) = 16.2 Hz, 1H; aromatic proton), 7.51 (br t, ^3^*J*(H,H) = 13.6 Hz, 1H; aromatic proton), 7.44–6.90 (m, 17H; aromatic protons), 6.68 (t, ^3^*J*(H,H) = 14.9 Hz, 2H; aromatic protons), 5.92 (m, 1H; PCH_2_), 5.33 (m, 1H; PCH_2_), 3.61 (dd, ^2^*J*(H,H) = 16.2 Hz, ^3^*J*(H,H) = 5.6 Hz, 1H; CH_2_N), 2.35 (m, 1H; CH_2_N), 2.03 (s, 3H; OCOCH_3_), 1.66 (s, 3H; OCOCH_3_). ^13^C{^1^H} NMR (100.6 MHz, CD_3_OD, 20 °C): δ 181.5 (br s; O*C*OCH_3_), 178.4 (br s; O*C*OCH_3_), 162.5 (s; N*C*CH_2_), 154.5 (s; N*C*H of C_5_H_4_N), 138.5–120.1 (m; aromatic carbon atoms), 50.9 (t, ^3^*J*(C,P) = 5.6 Hz; CH_2_N), 27.0 (m; PCH_2_), 23.9 (s; OCO*C*H_3_), 22.6 (s; OCO*C*H_3_). ^31^P{^1^H} NMR (162.0 MHz, CD_3_OD, 20 °C): δ 21.1 (d, ^2^*J*(P,P) = 84.1 Hz), 7.4 (d, ^2^*J*(P,P) = 84.1 Hz).

### Synthesis of *trans*-[Ru(κ^1^-OAc)_2_(dppf)(en)] (**7**)

[Ru(κ^2^-OAc)_2_(PPh_3_)_2_] (100 mg, 0.134 mmol) and dppf (75 mg, 0.135 mmol, 1.0 equiv) were dissolved in CH_2_Cl_2_ (1.5 mL) and stirred at room temperature for 1 h. After addition of ethylenediamine (en) (13 μL, 0.195 mmol, 1.46 equiv), the solution was stirred at room temperature for 30 min until a yellow precipitate was formed. *n*-Pentane (3 mL) was added to the mixture, which was stirred for 30 min and filtered, giving a yellow compound, which was washed with *n*-pentane (3 × 5 mL) and dried under reduced pressure. Yield: 101 mg (90%). Anal. Calcd for C_40_H_42_FeN_2_O_4_P_2_Ru (833.65): C, 57.63; H, 5.08; N, 3.36. Found: C, 57.65; H, 5.14; N, 3.40. ^1^H NMR (200.1 MHz, CD_2_Cl_2_, 20 °C): δ 7.69–7.55 (m, 8H; aromatic protons), 7.45–7.20 (m, 12H; aromatic protons), 4.92 (br s, 4H; NH_2_), 4.46 (m, 4H; C_5_H_4_), 4.24 (*pseudo*-t, *J*(H,H) = 1.8 Hz, 4H; C_5_H_4_), 2.64 (br t, *J*(H,H) = 4.6 Hz, 4H; NCH_2_CH_2_N), 1.69 (s, 6H; OCOCH_3_). ^13^C{^1^H} NMR (50.3 MHz, CD_2_Cl_2_, 20 °C): δ 182.0 (t, ^3^*J*(C,P) = 1.0 Hz; O*C*OCH_3_), 138.0 (*pseudo*-t, *J*(C,P) = 18.3 Hz; *ipso*-Ph), 134.5 (t, *J*(C,P) = 5.1 Hz; *ortho*-Ph), 129.3 (t, *J*(C,P) = 1.0 Hz; *para*-Ph), 127.7 (t, *J*(C,P) = 4.3 Hz; *meta*-Ph), 83.2 (*pseudo*-t, *J*(C,P) = 23.8 Hz; *ipso*-C_5_H_4_), 75.1 (t, *J*(C,P) = 4.0 Hz; *C*_5_H_4_), 71.6 (t, ^3^*J*(C,P) = 2.8 Hz; *C*_5_H_4_), 43.9 (m; CH_2_N), 26.0 (s; OCO*C*H_3_). ^31^P{^1^H} NMR (81.0 MHz, CD_2_Cl_2_, 20 °C): δ 48.3 (s).

### Synthesis of *trans*-[Ru(κ^1^-OAc)_2_(dppp)(ampy)] (**8**)

#### Method 1

[Ru(κ^2^-OAc)_2_(PPh_3_)_2_] (100 mg, 0.134 mmol) and dppp (56.1 mg, 0.136 mmol, 1.01 equiv) were dissolved in CH_2_Cl_2_ (2 mL) and stirred at room temperature for 1 h. ampy (20 μL, 0.194 mmol, 1.45 equiv) was added to the mixture, and the resulting light orange solution was stirred for 1 h at room temperature. The solvent was removed under reduced pressure, *n*-pentane (5 mL) was added, and the suspension was stirred for 10 min. After filtration, the yellow product was washed with *n*-pentane (4 × 3 mL) and dried under reduced pressure. Yield: 84 mg (85%). Anal. Calcd for C_37_H_40_N_2_O_4_P_2_Ru (739.75): C, 60.07; H, 5.45; N, 3.79. Found: C, 60.12; H, 5.44; N, 3.83. ^1^H NMR (200.1 MHz, CD_2_Cl_2_, 20 °C): δ 8.42 (d, ^3^*J*(H,H) = 4.8 Hz, 1H; *ortho-*CH of C_5_H_4_N), 7.78–6.96 (m, 22H; aromatic protons), 6.71 (*pseudo*-t, *J*(H,H) = 6.5 Hz, 1H; aromatic proton), 6.28 (br s, 2H; NH_2_), 4.12 (br m, 2H; CH_2_N), 2.62–2.35 (m, 4H; PCH_2_), 2.30–1.80 (m, 2H; CH_2_), 1.68 (s, 6H; OCOCH_3_). ^13^C{^1^H} NMR (50.3 MHz, CD_2_Cl_2_, 20 °C): δ 180.9 (d, ^3^*J*(C,P) = 1.7 Hz; O*C*OCH_3_), 166.3 (dd, ^3^*J*(C,P) = 2.7 Hz, ^3^*J*(C,P) = 1.4 Hz; N*C*CH_2_), 154.7 (dd, ^3^*J*(C,P) = 3.9 Hz, ^3^*J*(C,P) = 0.5 Hz; N*C*H of C_5_H_4_N), 138.6–119.7 (m; aromatic carbon atoms), 50.5 (dd, ^3^*J*(C,P) = 3.6 Hz, ^3^*J*(C,P) = 2.0 Hz; *C*H_2_N), 27.4 (m; PCH_2_), 26.8 (m; P*C*H_2_), 25.2 (s; OCO*C*H_3_), 19.5 (dd, ^2^*J*(C,P) = 2.4 Hz, ^2^*J*(C,P) = 0.5 Hz; PCH_2_*C*H_2_). ^31^P{^1^H} NMR (81.0 MHz, CD_2_Cl_2_, 20 °C): δ 47.8 (d, ^2^*J*(P,P) = 49.1 Hz), 33.1 (d, ^2^*J*(P,P) = 49.l Hz).

#### Method 2

[Ru(κ^2^-OAc)_2_(PPh_3_)_2_] (200 mg, 0.269 mmol) and dppp (112 mg, 0.272 mmol) were suspended in acetone (2 mL) and stirred for 3 h at room temperature. ampy (40 μL, 0.388 mmol, 1.44 equiv) was added, and the suspension was stirred for 1 h at room temperature. The solid was filtered, washed with *n*-hexane (3 × 2 mL), and dried under reduced pressure. Yield: 185 mg (93%).

#### Method 3

*trans*,*cis*-[Ru(OAc)_2_(PPh_3_)_2_(ampy)] (**2**) (100 mg, 0.117 mmol) and dppp (49.5 mg, 0.120 mmol, 1.03 equiv) were stirred in MEK (1 mL) at 50 °C for 20 h. The solution was evaporated under reduced pressure, and the residue was added to *n*-heptane (5 mL). The suspension was stirred for 10 min at room temperature, and the solid was filtered, washed with *n*-heptane (2 × 3 mL) and *n*-pentane (2 × 2 mL), and dried under reduced pressure. Yield: 53 mg (61%).

### Synthesis of [Ru(κ^2^-OAc)(dppp)(ampy)]OAc (**8a**)

Complex **8** (20 mg, 0.027 mmol) was dissolved in CH_3_OH (2 mL), and the solution was stirred at room temperature for 56 h. The solvent was evaporated under reduced pressure, and the residue was dissolved in CH_2_Cl_2_ (0.5 mL). Addition of *n*-pentane (2 mL) afforded a yellow-orange precipitate, which was filtered, washed with *n*-pentane (3 × 2 mL), and dried under reduced pressure. Yield: 18 mg (90%). Anal. Calcd for C_37_H_40_N_2_O_4_P_2_Ru (739.75): C, 60.07; H, 5.45; N, 3.79. Found: C, 60.10; H, 5.47; N, 3.81. ^1^H NMR (400.1 MHz, CD_3_OD, 20 °C): δ 8.09 (d, ^3^*J*(H,H) = 5.7 Hz, 1H; *ortho-*CH of C_5_H_4_N), 7.82 (t, ^3^*J*(H,H) = 8.8 Hz, 2H; aromatic protons), 7.76–6.94 (m, 18H; aromatic protons), 6.87 (t, ^3^*J*(H,H) = 6.8 Hz, 2H; aromatic protons), 6.80 (t, ^3^*J*(H,H) = 8.6 Hz, 1H; aromatic protons), 3.92 (d, ^2^*J*(H,H) = 16.9 Hz, 1H; CH_2_N), 3.29 (d, ^2^*J*(H,H) = 16.9 Hz, 1H; CH_2_N), 3.16–2.77 (m, 4H, PCH_2_), 2.74–2.35 (m, 2H, CH_2_), 1.92 (s, 3H; CH_3_CO_2_), 1.52 (s, 3H; CH_3_CO_2_). ^13^C{^1^H} NMR (100.6 MHz, CD_3_OD, 20 °C): δ 189.6 (d, ^2^*J*(C,P) = 2.8 Hz; O*C*OCH_3_), 180.4 (br s; O*C*OCH_3_), 162.9 (d, ^3^*J*(C,P) = 1.4 Hz; N*C*CH_2_), 149.9 (br s; N*C*H of C_5_H_4_N), 139.0–122.2 (m; aromatic carbon atoms), 53.5 (d, ^3^*J*(C,P) = 3.0 Hz; CH_2_N), 29.8 (dd, ^1^*J*(C,P) = 31.7 Hz, ^3^*J*(C,P) = 2.7 Hz; P*C*H_2_), 29.6 (dd, ^1^*J*(C,P) = 30.3 Hz, ^3^*J*(C,P) = 2.7 Hz; P*C*H_2_), 24.8 (br s; OCO*C*H_3_), 24.4 (s; OCO*C*H_3_), 21.9 (t, *J*(C,P) = 1.9 Hz; *C*H_2_). ^31^P{^1^H} NMR (162.0 MHz, CD_3_OD): δ 55.2 (d, ^2^*J*(P,P) = 48.4 Hz), 36.7 (d, ^2^*J*(P,P) = 48.4 Hz).

### Synthesis of *trans*-[Ru(κ^1^-OAc)_2_(dppb)(ampy)] (**9**)

#### Method 1

Complex **9** was prepared by following the procedure used for the synthesis of **8** (method 1), with dppb (57.8 mg, 0.136 mmol, 1.01 equiv) in place of dppp. Yield: 62 mg (61%). Anal. Calcd for C_38_H_42_N_2_O_4_P_2_Ru (753.78): C, 60.55; H, 5.62; N, 3.72. Found: C, 60.50; H, 5.65; N, 3.70. ^1^H NMR (200.1 MHz, CD_2_Cl_2_, 20 °C): δ 8.95 (m, 1H; *ortho-*CH of C_5_H_4_N), 7.83–7.08 (m, 22H; aromatic protons), 6.81 (*pseudo*-t, ^3^*J*(H,H) = 6.6 Hz, 1H; aromatic proton), 6.03 (m, 2H; NH_2_), 4.06 (m, 2H; CH_2_N), 2.78 (m, 2H; PCH_2_), 2.25 (m, 2H; PCH_2_), 1.94–1.64 (m, 4H; PCH_2_C*H*_2_C*H*_2_), 1.53 (s, 6H; OCOCH_3_). ^13^C{^1^H} NMR (50.3 MHz, CD_2_Cl_2_, 20 °C): δ 181.0 (d, ^3^*J*(C,P) = 1.5 Hz; O*C*OCH_3_), 167.5 (dd, ^3^*J*(C,P) = 2.9 Hz, ^3^*J*(C,P) = 1.4 Hz; N*C*CH_2_), 154.9 (d, *J*(C,P) = 3.7 Hz; N*C*H of C_5_H_4_N), 139.4–119.9 (m; aromatic carbon atoms), 50.6 (dd, ^3^*J*(C,P) = 3.8 Hz, ^3^*J*(C,P) = 2.0 Hz; CH_2_N), 33.9 (dd, ^1^*J*(C,P) = 27.1 Hz, ^3^*J*(C,P) = 3.0 Hz; PCH_2_), 27.7 (d, ^1^*J*(C,P) = 25.3 Hz; PCH_2_), 26.5 (m; PCH_2_*C*H_2_), 25.1 (m; OCO*C*H_3_), 19.9 (m; PCH_2_*C*H_2_). ^31^P{^1^H} NMR (81.0 MHz, CD_2_Cl_2_, 20 °C): δ 51.1 (d, ^2^*J*(P,P) = 36.6 Hz), 36.5 (d. ^2^*J*(P,P) = 36.6 Hz).

#### Method 2

Complex **9** was prepared by following the procedure used for the synthesis of **8** (method 2), with dppb (115.6 mg, 0.271 mmol, 1.01 equiv) in place of dppp. Yield: 156 mg (77%).

#### Method 3

Complex **9** was prepared by following the procedure used for the synthesis of **8** (method 3), with dppb (51.2 mg, 0.120 mmol, 1.03 equiv) in place of dppp. Yield: 62 mg (70%).

### Synthesis of [Ru(κ^2^-OAc)(dppb)(ampy)]OAc (**9a**)

Complex **9a** was prepared by following the procedure used for the synthesis of **8a**, employing *trans*-[Ru(κ^1^-OAc)_2_(dppb)(ampy)] (**9**) (20 mg, 0.0265 mmol) in place of **8**. The solution of **9** in CH_3_OH was stirred for 48 h at room temperature. Yield: 19.6 mg (98%). Anal. Calcd for C_38_H_42_N_2_O_4_P_2_Ru (753.78): C, 60.55; H, 5.62; N, 3.72. Found: C, 60.60; H, 5.64; N, 3.76. ^1^H NMR (400.1 MHz, CD_3_OD, 20 °C): δ 8.26 (d, ^3^*J*(H,H) = 5.6 Hz, 1H; *ortho-*CH of C_5_H_4_N), 7.90 (ddd, ^3^*J*(H,H) = 9.6 Hz, ^3^*J*(H,H) = 7.9 Hz, ^4^*J*(H,H) = 1.6 Hz, 2H; aromatic protons), 7.77 (m, 2H; aromatic protons), 7.67 (td, ^3^*J*(H,H) = 7.7 Hz, ^4^*J*(H,H) = 1.6 Hz, 1H; aromatic proton), 7.63–7.49 (m, 6H, aromatic protons), 7.44–7.25 (m, 5H; aromatic protons), 7.22 (d, ^3^*J*(H,H) = 8.0 Hz, 1H; aromatic proton), 7.15 (t, ^3^*J*(H,H) = 6.2 Hz, 1H; aromatic proton), 7.06 (m, 1H; aromatic proton), 6.98 (td, ^3^*J*(H,H) = 7.9 Hz, ^4^*J*(H,H) = 1.6 Hz, 2H; aromatic protons), 6.87 (t, ^3^*J*(H,H) = 8.6 Hz, 2H; aromatic protons), 4.03 (d, ^2^*J*(H,H) = 16.4 Hz, 1H; CH_2_N), 3.60 (d, ^2^*J*(H,H) = 16.4 Hz, 1H; CH_2_N), 3.17–2.93 (m, 2H, PCH_2_), 2.46 (m, 1H; PCH_2_), 2.30 (m, 1H; PCH_2_), 2.20–1.99 (m, 2H, CH_2_), 1.92 (s, 3H; CH_3_CO_2_), 1.80 (*pseudo*-q, *J*(H,H) = 13.6 Hz, 1H; CH_2_), 1.73–1.54 (m, 1H; CH_2_), 1.45 (s, 3H; CH_3_CO_2_). ^13^C{^1^H} NMR (100.6 MHz, CD_3_OD, 20 °C): δ 189.7 (t, ^2^*J*(C,P) = 2.0 Hz; O*C*OCH_3_), 180.5 (s; O*C*OCH_3_), 162.0 (d, ^3^*J*(C,P) = 1.5 Hz; N*C*CH_2_), 150.9 (s; N*C*H of C_5_H_4_N), 140.4–121.5 (m; aromatic carbon atoms), 53.6 (d, ^3^*J*(C,P) = 2.9 Hz; CH_2_N), 31.3 (d, ^1^*J*(C,P) = 29.3 Hz; P*C*H_2_), 29.4 (*pseudo*-t, *J*(C,P) = 27.9 Hz; P*C*H_2_), 26.4 (br s; *C*H_2_), 24.7 (d; ^4^*J*(C,P) = 1.4 Hz; OCO*C*H_3_), 24.4 (s; OCO*C*H_3_), 23.6 (br s; *C*H_2_). ^31^P{^1^H} NMR (162.0 MHz, CD_3_OD, 20 °C): δ 58.2 (d, ^2^*J*(P,P) = 37.2 Hz), 46.0 (d, ^2^*J*(P,P) = 37.2 Hz).

### Synthesis of *trans*-[Ru(κ^1^-OAc)_2_(dppf)(ampy)] (**10**)

#### Method 1

Complex **10** was prepared by following the procedure used for the synthesis of **8** (method 1), with dppf (75 mg, 0.135 mmol, 1.01 equiv) in place of dppp. Yield: 87 mg (74%). Anal. Calcd for C_44_H_42_FeN_2_O_4_P_2_Ru (881.69): C, 59.94; H, 4.80; N, 3.18. Found: C, 60.00; H, 4.85; N, 3.20. ^1^H NMR (200.1 MHz, CD_2_Cl_2_, 20 °C): δ 8.62 (d, ^3^*J*(H,H) = 3.1 Hz, 1H; *ortho-*CH of C_5_H_4_N), 7.81 (t, ^3^*J*(H,H) = 7.9 Hz, 3H; aromatic protons), 7.56 (t, ^3^*J*(H,H) = 8.8 Hz, 4H; aromatic protons), 7.49–6.92 (m, 15H; aromatic protons), 6.68 (*pseudo*-t, *J*(H,H) = 6.3 Hz, 1H; aromatic proton), 6.34 (*pseudo*-q, *J*(H,H) = 5.7 Hz, 2H; NH_2_), 4.68 (br s, 2H; C_5_H_4_), 4.32 (br s, 2H; C_5_H_4_), 4.15–3.88 (m, 6H; C_5_H_4_ and CH_2_N), 1.55 (s, 6H; OCOCH_3_). ^13^C{^1^H} NMR (50.3 MHz, CD_2_Cl_2_, 20 °C): δ 181.2 (d, ^3^*J*(C,P) = 1.4 Hz, O*C*OCH_3_), 167.7 (dd, ^3^*J*(C,P) = 2.9 Hz, ^3^*J*(C,P) = 1.6 Hz; N*C*CH_2_), 154.6 (d, ^3^*J*(C,P) = 3.2 Hz; N*C*H of C_5_H_4_N), 136.6–120.1 (m; aromatic carbon atoms), 82.7 (dd, ^1^*J*(C,P) = 43.7 Hz, ^3^*J*(C,P) = 4.0 Hz; *ipso*-C_5_H_4_), 81.4 (dd, ^1^*J*(C,P) = 47.3 Hz, ^3^*J*(C,P) = 2.2 Hz; *ipso*-C_5_H_4_), 75.5 (*pseudo*-t, *J*(C,P) = 8.0 Hz; C_5_H_4_), 72.9 (d, *J*(C,P) = 5.6 Hz; C_5_H_4_), 70.8 (d, *J*(C,P) = 4.5 Hz; C_5_H_4_), 50.7 (m; CH_2_N), 25.5 (s; OCO*C*H_3_). ^31^P{^1^H} NMR (81.0 MHz, CD_2_Cl_2_, 20 °C): δ 56.2 (d, ^2^*J*(P,P) = 37.8 Hz), 35.0 (d, ^2^*J*(P,P) = 37.8 Hz).

#### Method 2

Complex **10** was prepared by following the procedure used for the synthesis of **8** (method 2), with dppf (149 mg, 0.269 mmol, 1.0 equiv) in place of dppp. Yield: 209 mg (88%).

#### Method 3

Complex **10** was prepared by following the procedure used for the synthesis of **8** (method 3), with dppf (66.5 mg, 0.120 mmol, 1.03 equiv) in place of dppp. Yield: 75 mg (73%).

### Synthesis of [Ru(κ^2^-OAc)(dppf)(ampy)]OAc (**10a**)

Complex **10a** was prepared by following the procedure used for the synthesis of **8a**, employing *trans*-[Ru(κ^1^-OAc)_2_(dppf)(ampy)] (**10**) (20 mg, 0.0227 mmol) in place of **8**. The solution of **10** in CH_3_OH was stirred for 4 h at room temperature. Yield: 19 mg (95%). Anal. Calcd for C_44_H_42_FeN_2_O_4_P_2_Ru (881.69): C, 59.94; H, 4.80; N, 3.18. Found: C, 60.01; H, 4.84; N, 3.16. ^1^H NMR (400.1 MHz, CD_3_OD, 20 °C): δ 7.94 (d, ^3^*J*(H,H) = 5.7 Hz, 1H; *ortho-*CH of C_5_H_4_N), 7.82 (t, ^3^*J*(H,H) = 7.5 Hz, 1H; aromatic proton), 7.70–7.33 (m, 14H, aromatic protons), 7.32–7.23 (m, 4H; aromatic protons), 7.15 (t, ^3^*J*(H,H) = 8.8 Hz, 2H; aromatic protons), 7.08 (t, ^3^*J*(H,H) = 6.7 Hz, 1H; aromatic proton), 4.55 (s, 1H; C_5_H_4_), 4.52 (s, 2H; C_5_H_4_), 4.43 (s, 1H; C_5_H_4_), 4.41 (s, 2H; C_5_H_4_), 4.38 (s, 1H; C_5_H_4_), 4.24 (s, 1H; C_5_H_4_), 3.91 (d, ^2^*J*(H,H) = 16.3 Hz, 1H; CH_2_N), 3.60 (d, ^2^*J*(H,H) = 16.3 Hz, 1H; CH_2_N), 1.92 (s, 3H; OCOCH_3_), 1.26 (s, 3H; OCOCH_3_). ^13^C{^1^H} NMR (100.6 MHz, CD_3_OD, 20 °C): δ 190.8 (t, ^2^*J*(C,P) = 2.9 Hz; O*C*OCH_3_), 180.4 (s; O*C*OCH_3_), 162.4 (d, ^3^*J*(C,P) = 1.5 Hz; N*C*CH_2_), 151.2 (br s; N*C*H of C_5_H_4_N), 139.2–122.3 (m; aromatic carbon atoms), 80.6 (dd, ^1^*J*(C,P) = 55.0 Hz, ^3^*J*(C,P) = 3.6 Hz; *ipso*-C_5_H_4_), 78.5 (d, *J*(C,P) = 11.7 Hz; C_5_H_4_), 77.3 (dd, ^2^*J*(C,P) = 11.7 Hz, ^3^*J*(C,P) = 0.7 Hz; C_5_H_4_), 76.3 (dd, ^1^*J*(C,P) = 54.5 Hz, ^3^*J*(C,P) = 1.3 Hz; *ipso*-C_5_H_4_), 76.0 (d, *J*(C,P) = 15.3 Hz; C_5_H_4_), 75.9 (d, *J*(C,P) = 15.4 Hz; C_5_H_4_), 74.5 (*pseudo*-t, *J*(C,P) = 7.3 Hz; C_5_H_4_), 74.3 (d, *J*(C,P) = 7.3 Hz; C_5_H_4_), 74.0 (d, *J*(C,P) = 5.9 Hz; C_5_H_4_), 53.1 (d, ^3^*J*(C,P) = 2.3 Hz; CH_2_N), 24.4 (s; OCO*C*H_3_), 24.3 (br s; OCO*C*H_3_). ^31^P{^1^H} NMR (162.0 MHz, CD_3_OD, 20 °C): δ 59.9 (d, ^2^*J*(P,P) = 35.3 Hz), 49.6 (d, ^2^*J*(P,P) = 35.3 Hz).

### Synthesis of *trans*-[Ru(κ^1^-OAc)_2_((*R*)-BINAP)(ampy)] (**11**)

[Ru(κ^2^-OAc)_2_(PPh_3_)_2_] (100 mg, 0.134 mmol) and (*R*)-BINAP (85 mg, 0.136 mmol, 1.01 equiv) were suspended in toluene (1.5 mL) and refluxed for 24 h. The resulting orange solution was cooled to room temperature, and ampy (20 μL, 0.194 mmol, 1.45 equiv) was added. The light orange solution obtained was stirred for 1 h at room temperature and the solvent removed under reduced pressure. Treatment of the residue with *n*-pentane (10 mL) led to a suspension, which was stirred for 10 min, and the yellow precipitate obtained was filtered, washed with *n*-pentane (3 × 5 mL), and dried under reduced pressure. Yield: 75.1 mg (59%). Anal. Calcd for C_54_H_46_N_2_O_4_P_2_Ru (949.99): C, 68.27; H, 4.88; N, 2.95. Found: C, 68.35; H, 4.85; N, 3.01. ^1^H NMR (400.1 MHz, CD_2_Cl_2_, 25 °C): δ 8.48 (m, 1H; *ortho-*CH of C_5_H_4_N), 8.18 (t, ^3^*J*(H,H) = 8.1 Hz, 1H; aromatic proton), 7.75–7.69 (m, 2H; aromatic protons), 7.65–7.26 (m, 16H; aromatic protons), 7.25–7.18 (m, 4H; aromatic protons), 7.03–6.91 (m, 2H; aromatic proton and NH_2_), 6.88 (t, ^3^*J*(H,H) = 8.0 Hz, 1H; aromatic proton), 6.84–6.76 (m, 2H; aromatic protons), 6.72 (t, ^3^*J*(H,H) = 7.1 Hz, 2H; aromatic protons), 6.62 (t, ^3^*J*(H,H) = 6.5 Hz, 1H; aromatic proton), 6.56 (d, ^3^*J*(H,H) = 8.6 Hz, 1H; aromatic proton), 6.43 (m, 3H; aromatic protons), 6.26 (d, ^3^*J*(H,H) = 8.7 Hz, 1H; aromatic proton), 5.06 (br q, ^3^*J*(H,H) = 6.6 Hz, 1H; NH_2_), 4.07 (dt, ^2^*J*(H,H) = 16.0 Hz, ^3^*J*(H,H) = 5.6 Hz, 1H; CH_2_N), 3.97 (m, 1H; CH_2_N), 1.83 (s, 3H; OCOCH_3_), 1.61 (s, 3H; OCOCH_3_). ^13^C{^1^H} NMR (100.6 MHz, CD_2_Cl_2_, 25 °C): δ 181.8 (d, ^3^*J*(C,P) = 1.5 Hz; O*C*OCH_3_), 181.3 (d, ^3^*J*(C,P) = 1.4 Hz; O*C*OCH_3_), 166.9 (dd, ^3^*J*(C,P) = 2.7 Hz, ^3^*J*(C,P) = 1.1 Hz; N*C*CH_2_), 155.5 (d, ^3^*J*(C,P) = 3.7 Hz; N*C*H of C_5_H_4_N), 139.0–119.9 (m; aromatic carbon atoms), 50.6 (t, ^3^*J*(C,P) = 2.1 Hz; *C*H_2_N), 25.8 (br s; OCO*C*H_3_), 25.2 (br s; OCO*C*H_3_). ^31^P{^1^H} NMR (162.0 MHz, CD_2_Cl_2_, 25 °C): δ 54.9 (d, ^2^*J*(P,P) = 36.9 Hz), 40.9 (d, ^2^*J*(P,P) = 36.9 Hz).

### Synthesis of [Ru(κ^2^-OAc)((*R*)-BINAP)(ampy)]OAc (**11a**)

Complex **11a** was prepared by following the procedure used for the synthesis of **8a**, employing *trans*-[Ru(κ^1^-OAc)_2_((*R*)-BINAP)(ampy)] (**11**) (22 mg, 0.0232 mmol) in place of **8**. The solution of **11** in CH_3_OH was stirred for 18 h at room temperature. The product consists of a 2:1 molar mixture of two stereoisomers. Yield: 21.1 mg (96%). Anal. Calcd for C_54_H_46_N_2_O_4_P_2_Ru (949.99): C, 68.27; H, 4.88; N, 2.95. Found: C, 68.32; H, 4.83; N, 2.99. ^1^H NMR (400.1 MHz, CD_3_OD, 25 °C): δ 8.38 (d, ^3^*J*(H,H) = 2.9 Hz, 1H; *ortho-*CH of C_5_H_4_N major isomer), 8.21 (d, ^3^*J*(H,H) = 3.9 Hz, 1H; *ortho-*CH of C_5_H_4_N minor isomer), 8.09–5.94 (m, 22H; aromatic protons both isomers), 4.39 (d, ^2^*J*(H,H) = 16.2 Hz, 1H; CH_2_N major isomer), 4.16 (d, ^2^*J*(H,H) = 16.2 Hz, 1H; CH_2_N major isomer), 4.08 (d, ^2^*J*(H,H) = 16.4 Hz, 1H; CH_2_N minor isomer), 3.94 (d, ^2^*J*(H,H) = 16.4 Hz, 1H; CH_2_N minor isomer), 1.92 (s, 3H; OCOCH_3_ minor and major isomers), 1.50 (s, 3H; OCOCH_3_ major isomer), 1.41 (s, 3H; OCOCH_3_ minor isomer). ^13^C{^1^H} NMR (100.6 MHz, CD_3_OD, 25 °C): δ 189.3 (d, ^2^*J*(C,P) = 2.4 Hz; O*C*OCH_3_ major isomer), 189.0 (d, ^2^*J*(C,P) = 2.3 Hz; O*C*OCH_3_ minor isomer), 180.4 (s; O*C*OCH_3_ both isomers), 162.4 (br s; N*C*CH_2_ minor isomer), 162.1 (br s; N*C*CH_2_ major isomer), 151.7 (br s; N*C*H of C_5_H_4_N minor isomer), 151.2 (br s; N*C*H of C_5_H_4_N major isomer), 143.0–122.2 (m; aromatic carbon atoms both isomers), 53.1 (d, ^3^*J*(C,P) = 2.2 Hz; CH_2_N major isomer), 52.4 (d, ^3^*J*(C,P) = 2.4 Hz; CH_2_N minor isomer), 24.4 (br s; OCO*C*H_3_ major isomer), 24.2 (br s; OCO*C*H_3_ minor isomer), 23.9 (d; ^4^*J*(C,P) = 3.4 Hz; OCO*C*H_3_ minor isomer), 23.8 (br s; OCO*C*H_3_ major isomer). ^31^P{^1^H} NMR (162.0 MHz, CD_3_OD, 25 °C): δ 68.3 (d, ^2^*J*(P,P) = 38.7 Hz; minor isomer), 61.1 (d, ^2^*J*(P,P) = 38.8 Hz; major isomer), 58.1 (d, ^2^*J*(P,P) = 38.7 Hz; minor isomer), 52.4 (d, ^2^*J*(P,P) = 38.8 Hz; major isomer).

### Synthesis of *trans*-[Ru(κ^1^-OAc)_2_(dppp)(ampyrim)] (**12**)

#### Method 1

Complex **12** was prepared by following the procedure used for the synthesis of **8** (method 1), with ampyrim (18.7 μL, 0.195 mmol, 1.45 equiv) in place of ampy. Yield: 82 mg (82%).

#### Method 2

*trans*,*cis*-[Ru(κ^1^-OAc)_2_(PPh_3_)_2_(ampyrim)] (**3**) (100 mg, 0.117 mmol) and dppp (49.5 mg, 0.120 mmol, 1.03 equiv) were stirred in MEK (1 mL) at 50 °C for 18 h. The resulting solution was evaporated under reduced pressure and the residue added to *n*-heptane (5 mL). The suspension was stirred for 10 min and the solid filtered, washed with *n*-heptane (2 × 3 mL) and *n*-pentane (2 × 2 mL), and dried under reduced pressure. Yield: 49 mg (57%). Anal. Calcd for C_36_H_39_N_3_O_4_P_2_Ru (740.74): C, 58.37; H, 5.31; N, 5.67. Found: C, 58.35; H, 5.35; N, 5.71. ^1^H NMR (400.1 MHz, CD_2_Cl_2_, 25 °C): δ 8.52 (dd, ^3^*J*(H,H) = 4.5 Hz, ^4^*J*(H,H) = 1.9 Hz, 1H; RuNC*H* of C_4_H_3_N_2_), 8.41 (m, 1H; NC*H* of C_4_H_3_N_2_), 7.66 (t, ^3^*J*(H,H) = 8.0 Hz, 4H; aromatic protons), 7.47 (t, ^3^*J*(H,H) = 6.8 Hz, 2H; aromatic protons), 7.43–7.30 (m, 8H; aromatic protons), 7.27 (t, ^3^*J*(H,H) = 7.1 Hz, 2H; aromatic protons), 7.12 (t, ^3^*J*(H,H) = 6.6 Hz, 4H; aromatic protons), 6.74 (t, ^3^*J*(H,H) = 5.1 Hz, 1H; aromatic proton), 6,07 (br s, 2H; NH_2_), 4.28 (td, ^3^*J*(H,H) = 6.7 Hz, ^4^*J*(H,H) = 2.6 Hz, 2H; CH_2_N), 2.59–2.44 (m, 4H; PCH_2_), 2.02–1.85 (m, 2H; CH_2_), 1.75 (s, 6H; OCOCH_3_). ^13^C{^1^H} NMR (100.6 MHz, CD_2_Cl_2_, 25 °C): δ 182.3 (d, ^3^*J*(CP) = 2.2 Hz; O*C*OCH_3_), 177.8 (dd, ^3^*J*(CP) = 2.5 Hz, ^3^*J*(CP) = 1.3 Hz; N*C*CH_2_), 162.1 (d, ^3^*J*(CP) = 3.1 Hz; RuN*C*H of C_4_H_3_N_2_), 157.4 (s; N*C*H of C_4_H_3_N_2_), 138.4 (d, ^1^*J*(CP) = 41.1 Hz; *ipso* aromatic carbon atoms), 134.9–128.7 (m; aromatic carbon atoms), 119.8 (t, *J*(CP) = 1.5 Hz; aromatic carbon atom), 52.0 (t, ^3^*J*(CP) = 2.2 Hz; *C*H_2_N), 28.1 (dd, ^1^*J*(CP) = 30.1 Hz, ^3^*J*(CP) = 5.1 Hz; P*C*H_2_), 28.0 (d, ^1^*J*(CP) = 30.2 Hz; P*C*H_2_), 26.4 (s; OCO*C*H_3_), 20.7 (d, ^2^*J*(CP) = 2.2 Hz; *C*H_2_). ^31^P{^1^H} NMR (162.0 MHz, CD_2_Cl_2_, 25 °C): δ 47.9 (d, ^2^*J*(P,P) = 50.1 Hz), 32.6 (d, ^2^*J*(P,P) = 50.1 Hz).

### Synthesis of [Ru(κ^2^-OAc)(dppp)(ampyrim)]OAc (**12a**)

Complex **12a** was prepared by following the procedure used for the synthesis of **8a**, employing *trans*-[Ru(κ^1^-OAc)_2_(dppp)(ampyrim)] (**12**) (23 mg, 0.0310 mmol) in place of **8**. The solution of **12** in CH_3_OH was stirred for 64 h at room temperature. Yield: 20 mg (87%). Anal. Calcd for C_36_H_39_N_3_O_4_P_2_Ru (740.74): C, 58.37; H, 5.31; N, 5.67. Found: C, 58.32; H, 5.37; N, 5.63. ^1^H NMR (400.1 MHz, CD_3_OD, 25 °C): δ 8.93 (dd, ^3^*J*(H,H) = 4.8 Hz, ^4^*J*(H,H) = 2.9 Hz, 1H; RuNC*H* of C_4_H_3_N_2_), 8.47 (dd, ^3^*J*(H,H) = 4.8 Hz, ^4^*J*(H,H) = 1.9 Hz, 1H; NC*H* of C_4_H_3_N_2_), 7.88 (tt, ^3^*J*(H,H) = 8.5 Hz, ^4^*J*(H,H) = 1.3 Hz, 3H; aromatic protons), 7.74–7.63 (m, 5H; aromatic protons), 7.56–7.46 (m, 5H; aromatic protons), 7.43–6.97 (m, 7H; aromatic protons), 6.81 (td, ^3^*J*(H,H) = 8.2 Hz, ^4^*J*(H,H) = 3.9 Hz, 1H; aromatic proton), 4.30 (d, ^2^*J*(H,H) = 17.5 Hz, 1H; CH_2_N), 4.07 (d, ^2^*J*(H,H) = 17.5 Hz, 1H; CH_2_N), 3.12–2.99 (m, 4H; PCH_2_), 2.61–2.45 (m, 1H; CH_2_), 2.40–2.22 (m, 1H; CH_2_), 1.92 (s, 3H; OCOCH_3_), 1.49 (s, 3H; OCOCH_3_). ^13^C{^1^H} NMR (100.6 MHz, CD_3_OD, 25 °C): δ 190.3 (d, ^2^*J*(C,P) = 2.2 Hz; O*C*OCH_3_), 179.8 (br s; O*C*OCH_3_), 172.3 (d, ^3^*J*(C,P) = 1.4 Hz; N*C*CH_2_), 159.5 (s; RuN*C*H of C_4_H_3_N_2_), 158.0 (br s; N*C*H of C_4_H_3_N_2_), 137.7–128.8 (m; aromatic carbon atoms), 121.1 (br s; aromatic carbon atom), 53.9 (t, ^3^*J*(C,P) = 1.4 Hz; CH_2_N), 29.4 (dd, ^1^*J*(C,P) = 31.3 Hz, ^3^*J*(C,P) = 3.1 Hz; P*C*H_2_), 28.7 (d, ^1^*J*(C,P) = 34.5 Hz; P*C*H_2_), 24.5 (d, ^4^*J*(C,P) = 1.4 Hz; OCO*C*H_3_), 24.0 (br s; OCO*C*H_3_), 21.0 (br s; *C*H_2_). ^31^P{^1^H} NMR (162.0 MHz, CD_3_OD, 25 °C): δ 56.1 (d, ^2^*J*(P,P) = 49.2 Hz), 37.7 (d, ^2^*J*(P,P) = 49.2 Hz).

### Synthesis of *trans*-[Ru(κ^1^-OAc)_2_(dppb)(ampyrim)] (**13**)

#### Method 1

Complex **13** was prepared by following the procedure used for the synthesis of **12** (method 1), with dppb (58.5 mg, 0.137 mmol, 1.02 equiv) in place of dppp. Yield: 78.2 mg (77%).

#### Method 2

Complex **13** was prepared by following the procedure used for the synthesis of **12** (method 2), with dppb (51.2 mg, 0.120 mmol, 1.03 equiv) in place of dppp. Yield: 66 mg (75%). Anal. Calcd for C_37_H_41_N_3_O_4_P_2_Ru (754.77): C, 58.88; H, 5.48; N, 5.57. Found: C, 58.92; H, 5.41; N, 5.51. ^1^H NMR (400.1 MHz, CD_2_Cl_2_, 25 °C): δ 9.00 (dt, ^3^*J*(H,H) = 5.8 Hz, ^4^*J*(H,H) = 2.9 Hz, 1H; RuNC*H* of C_4_H_3_N_2_), 8.56 (dd, ^3^*J*(H,H) = 4.8 Hz, ^4^*J*(H,H) = 2.2 Hz, 1H; NC*H* of C_4_H_3_N_2_), 7.74–7.66 (m, 4H; aromatic protons), 7.48–7.31 (m, 12H; aromatic protons), 7.23 (ddd, ^3^*J*(H,H) = 9.1 Hz, ^3^*J*(H,H) = 7.1 Hz, ^4^*J*(H,H) = 2.0 Hz, 4H; aromatic protons), 6.84 (t, ^3^*J*(H,H) = 5.2 Hz, 1H; aromatic proton), 5.79 (*pseudo*-q, *J*(H,H) = 5.4 Hz, 2H; NH_2_), 4.21 (td, ^3^*J*(H,H) = 6.3 Hz, ^4^*J*(H,H) = 2.8 Hz, 2H; CH_2_N), 2.83 (dt, ^3^*J*(H,H) = 10.0 Hz, ^3^*J*(H,H) = 6.3 Hz, 2H; PCH_2_), 2.30 (ddd, ^3^*J*(H,H) = 11.6 Hz, ^3^*J*(H,H) = 6.3 Hz, ^4^*J*(H,H) = 3.2 Hz, 2H; PCH_2_), 1.90–1.78 (m, 2H; CH_2_), 1.76–1.63 (m, 2H, CH_2_), 1.61 (s, 6H; OCOCH_3_). ^13^C{^1^H} NMR (100.6 MHz, CD_2_Cl_2_, 25 °C): δ 182.3 (d, ^3^*J*(CP) = 1.5 Hz; O*C*OCH_3_), 178.6 (dd, ^3^*J*(CP) = 3.3 Hz, ^3^*J*(CP) = 1.4 Hz; N*C*CH_2_), 162.5 (d, ^3^*J*(CP) = 3.5 Hz; RuN*C*H of C_4_H_3_N_2_), 157.5 (s; N*C*H of C_4_H_3_N_2_), 139.4 (d, ^1^*J*(CP) = 38.3 Hz; *ipso* aromatic carbon atoms), 139.0 (d, ^1^*J*(CP) = 31.9 Hz; *ipso* aromatic carbon atoms), 135.3–129.1 (m; aromatic carbon atoms), 119.9 (t, *J*(CP) = 1.4 Hz; aromatic carbon atom), 52.2 (t, ^3^*J*(CP) = 2.2 Hz; *C*H_2_N), 34.8 (dd, ^1^*J*(CP) = 27.3 Hz, ^3^*J*(CP) = 2.6 Hz; P*C*H_2_), 28.5 (d, ^1^*J*(CP) = 25.0 Hz; P*C*H_2_), 27.7 (s; *C*H_2_), 26.4 (s; OCO*C*H_3_), 21.0 (*pseudo*-t, *J*(CP) = 2.8 Hz; *C*H_2_). ^31^P{^1^H} NMR (162.0 MHz, CD_2_Cl_2_, 25 °C): δ 50.0 (d, ^2^*J*(P,P) = 37.7 Hz), 36.6 (d, ^2^*J*(P,P) = 37.7 Hz).

### Synthesis of [Ru(κ^2^-OAc)(dppb)(ampyrim)]OAc (**13a**)

Complex **13a** was prepared by following the procedure used for the synthesis of **8a**, employing *trans*-[Ru(κ^1^-OAc)_2_(dppb)(ampyrim)] (**13**) (25 mg, 0.0331 mmol) in place of **8**. The solution of **13** in CH_3_OH was stirred for 54 h at room temperature. Yield: 22 mg (88%). Anal. Calcd for C_37_H_41_N_3_O_4_P_2_Ru (754.77): C, 58.88; H, 5.48; N, 5.57. Found: C, 58.95; H, 5.44; N, 5.60. ^1^H NMR (400.1 MHz, CD_3_OD, 25 °C): δ 8.57 (dd, ^3^*J*(H,H) = 3.4 Hz, ^4^*J*(H,H) = 1.4 Hz, 1H; RuNC*H* of C_4_H_3_N_2_), 8.49 (d, ^3^*J*(H,H) = 3.7 Hz, 1H; NC*H* of C_4_H_3_N_2_), 8.00–7.84 (m, 3H; aromatic protons), 7.74 (t, ^3^*J*(H,H) = 8.6 Hz, 2H; aromatic protons), 7.67–7.48 (m, 5H; aromatic protons), 7.45–7.26 (m, 4H; aromatic protons), 7.21 (m, 2H; aromatic protons), 7.13–7.00 (m, 2H; aromatic protons), 6.91 (t, ^3^*J*(H,H) = 8.2 Hz, 2H; aromatic protons), 6,67 (t, ^3^*J*(H,H) = 8.0 Hz, 1H; aromatic proton), 4.09 (d, ^2^*J*(H,H) = 16.8 Hz, 1H; CH_2_N), 3.65 (d, ^2^*J*(H,H) = 16.8 Hz, 1H; CH_2_N), 3.19–3.03 (m, 2H; PCH_2_), 2.60–2.41 (m, 1H; PCH_2_), 2.33–2.23 (m, 1H; PCH_2_), 2.32–2.05 (m, 2H; CH_2_), 1.95–1.78 (m, 1H, CH_2_), 1.91 (s, 3H; OCOCH_3_), 1.64 (m, 1H, CH_2_), 1.49 (s, 3H; OCOCH_3_). ^13^C{^1^H} NMR (100.6 MHz, CD_3_OD, 25 °C): δ 190.2 (d, ^2^*J*(C,P) = 1.3 Hz; O*C*OCH_3_), 180.3 (s; O*C*OCH_3_), 172.2 (dd, ^3^*J*(C,P) = 3.5 Hz, ^3^*J*(C,P) = 1.4 Hz; N*C*CH_2_), 158.7 (s; RuN*C*H of C_4_H_3_N_2_), 158.3 (s; N*C*H of C_4_H_3_N_2_), 139.9–128.2 (m; aromatic carbon atoms), 122.0 (br s; aromatic carbon atom), 53.9 (d, ^3^*J*(C,P) = 1.5 Hz; CH_2_N), 29.3 (d, ^1^*J*(C,P) = 26.3 Hz; P*C*H_2_), 29.2 (d, ^1^*J*(C,P) = 30.3 Hz; P*C*H_2_), 26.6 (t, ^3^*J*(C,P) = 1.7 Hz; *C*H_2_), 24.8 (d, ^4^*J*(C,P) = 1.4 Hz; OCO*C*H_3_), 24.3 (br s; OCO*C*H_3_), 23.4 (br s; *C*H_2_). ^31^P{^1^H} NMR (162.0 MHz, CD_3_OD, 25 °C): δ 57.5 (d, ^2^*J*(P,P) = 37.2 Hz), 46.0 (d, ^2^*J*(P,P) = 37.2 Hz).

### Synthesis of *trans*-[Ru(κ^1^-OAc)_2_(dppf)(ampyrim)] (**14**)

#### Method 1

Complex **14** was prepared by following the procedure used for the synthesis of **12** (method 1), with dppf (76.0 mg, 0.137 mmol, 1.02 equiv) in place of dppp. Yield: 101 mg (85%).

#### Method 2

Complex **14** was prepared by following the procedure used for the synthesis of **12** (method 2), with dppf (66.5 mg, 0.120 mmol, 1.03 equiv) in place of dppp. The solution was heated at 50 °C for 36 h instead of the usual 18 h. Yield: 83 mg (80%). Anal. Calcd for C_43_H_41_FeN_3_O_4_P_2_Ru (882.68): C, 58.51; H, 4.68; N, 4.76. Found: C, 58.52; H, 4.71; N, 4.80. ^1^H NMR (400.1 MHz, CD_2_Cl_2_, 25 °C): δ 8.68 (d, ^3^*J*(H,H) = 5.8 Hz, 1H; RuNC*H* of C_4_H_3_N_2_), 8.55 (d, ^3^*J*(H,H) = 4.8 Hz, 1H; NC*H* of C_4_H_3_N_2_), 7.82–7.76 (m, 4H; aromatic protons), 7.62 (t, ^3^*J*(H,H) = 8.8 Hz, 4H; aromatic protons), 7.53–7.24 (m, 12H; aromatic protons), 6.74 (t, ^3^*J*(H,H) = 5.4 Hz, 1H; aromatic proton), 6.05 (br s, 2H; NH_2_), 4.80 (br s, 2H; C_5_H_4_), 4.38 (s, 2H; C_5_H_4_), 4.23–4.09 (m, 4H; C_5_H_4_ and CH_2_N), 4.03 (s, 2H; C_5_H_4_), 1.63 (s, 6H; OCOCH_3_). ^13^C{^1^H} NMR (100.6 MHz, CD_2_Cl_2_, 25 °C): δ 182.3 (d, ^3^*J*(CP) = 1.4 Hz; O*C*OCH_3_), 178.9 (dd, ^3^*J*(C,P) = 3.8 Hz, ^3^*J*(C,P) = 1.4 Hz; N*C*CH_2_), 162.2 (d, ^3^*J*(CP) = 2.2 Hz; RuN*C*H of C_4_H_3_N_2_), 157.7 (s; N*C*H of C_4_H_3_N_2_), 137.2–128.7 (m; aromatic carbon atoms), 119.8 (t, *J*(C,P) = 1.4 Hz; aromatic carbon atom), 83.4 (dd, ^1^*J*(C,P) = 44.4 Hz, ^3^*J*(C,P) = 4.0 Hz; *ipso*-C_5_H_4_), 82.3 (dd, ^1^*J*(C,P) = 48.4 Hz, ^3^*J*(C,P) = 2.2 Hz; *ipso*-C_5_H_4_), 76.8 (d, *J*(C,P) = 8.1 Hz; *C*_5_H_4_), 76.7 (d, *J*(C,P) = 8.8 Hz; *C*_5_H_4_), 74.2 (d, *J*(C,P) = 5.9 Hz; *C*_5_H_4_), 72.2 (d, *J*(C,P) = 5.1 Hz), 52.3 (t, ^3^*J*(CP) = 1.5 Hz; *C*H_2_N), 26.8 (s; OCO*C*H_3_). ^31^P{^1^H} NMR (162.0 MHz, CD_2_Cl_2_, 25 °C): δ 55.3 (d, ^2^*J*(P,P) = 38.2 Hz), 35.2 (d, ^2^*J*(P,P) = 38.2 Hz).

### Synthesis of [Ru(κ^2^-OAc)(dppf)(ampyrim)]OAc (**14a**)

Complex **14a** was prepared by following the procedure used for the synthesis of **8a**, employing *trans*-[Ru(κ^1^-OAc)_2_(dppf)(ampyrim)] (**14**) (23 mg, 0.0261 mmol) in place of **8**. The solution of **14** in CH_3_OH was stirred for 4 h at room temperature. Yield: 22.5 mg (98%). Anal. Calcd for C_43_H_41_FeN_3_O_4_P_2_Ru (882.68): C, 58.51; H, 4.68; N, 4.76. Found: C, 58.56; H, 4.73; N, 4.74. ^1^H NMR (400.1 MHz, CD_3_OD, 25 °C): δ 8.71 (d, ^3^*J*(H,H) = 5.9 Hz, 1H; RuNC*H* of C_4_H_3_N_2_), 8.09 (d, ^3^*J*(H,H) = 4.3 Hz, 1H; NC*H* of C_4_H_3_N_2_), 7.70–7.57 (m, 6H, aromatic protons), 7.55–7.34 (m, 12H; aromatic protons), 7.32–7.11 (m, 7H; aromatic protons), 4.66 (s, 1H; C_5_H_4_), 4.58–4.39 (m, 5H; C_5_H_4_), 4.37–4.26 (m, 2H; C_5_H_4_), 3.96 (d, ^2^*J*(H,H) = 17.2 Hz, 1H; CH_2_N), 3.51 (d, ^2^*J*(H,H) = 17.2 Hz, 1H; CH_2_N), 1.91 (s, 3H; OCOCH_3_), 1.31 (s, 3H; OCOCH_3_). ^13^C{^1^H} NMR (100.6 MHz, CD_3_OD, 25 °C): δ 191.4 (d, ^2^*J*(C,P) = 2.1 Hz; O*C*OCH_3_), 180.4 (s; O*C*OCH_3_), 172.4 (d, ^3^*J*(C,P) = 1.5 Hz; N*C*CH_2_), 158.9 (s; RuN*C*H of C_4_H_3_N_2_), 158.0 (s; N*C*H of C_4_H_3_N_2_), 138.5–129.0 (m; aromatic carbon atoms), 122.4 (d, *J*(C,P) = 2.2 Hz; aromatic carbon atom), 80.0 (dd, ^1^*J*(C,P) = 55.7 Hz, ^3^*J*(C,P) = 2.9 Hz; *ipso*-C_5_H_4_), 78.7 (d, *J*(C,P) = 12.5 Hz; C_5_H_4_), 77.3 (d, ^2^*J*(C,P) = 9.5 Hz; C_5_H_4_), 76.1 (m; C_5_H_4_), 75.9 (dd, ^1^*J*(C,P) = 52.8 Hz, ^3^*J*(C,P) = 2.7 Hz; *ipso*-C_5_H_4_), 75.1 (d, *J*(C,P) = 7.3 Hz; C_5_H_4_), 75.0 (d, *J*(C,P) = 5.9 Hz; C_5_H_4_), 74.0 (d, *J*(C,P) = 6.6 Hz; C_5_H_4_), 73.9 (d, *J*(C,P) = 5.9 Hz; C_5_H_4_), 53.7 (d, ^3^*J*(C,P) = 1.5 Hz; CH_2_N), 24.6 (d, ^4^*J*(C,P) = 1.4 Hz; OCO*C*H_3_), 24.4 (br s; OCO*C*H_3_). ^31^P{^1^H} NMR (162.0 MHz, CD_3_OD, 25 °C): δ 59.8 (d, ^2^*J*(P,P) = 35.3 Hz), 47.6 (d, ^2^*J*(P,P) = 35.3 Hz).

### Synthesis of *trans*-[Ru(κ^1^-OAc)_2_((*R*)-BINAP)(ampyrim)] (**15**)

[Ru(κ^2^-OAc)_2_(PPh_3_)_2_] (100 mg, 0.134 mmol) and (*R*)-BINAP (85 mg, 0.136 mmol, 1.01 equiv) were suspended in toluene (1.5 mL) and refluxed for 24 h. The resulting orange solution was cooled to room temperature and evaporated to dryness. The residue was dissolved in acetone (2 mL), and ampyrim (18.7 μL, 0.195 mmol, 1.45 equiv) was added. The dark orange solution obtained was stirred for 18 h at room temperature and the solvent removed under reduced pressure. Treatment of the residue with a *n*-pentane/diethyl ether mixture (3/1; 5 mL) led to a suspension, which was stirred for 10 min. The resulting yellow precipitate was filtered, washed with an *n*-pentane/diethyl ether mixture (3/1; 4 × 5 mL) and then with *n*-pentane (2 × 5 mL), and finally dried under reduced pressure. Yield: 80.3 mg (63%). Anal. Calcd for C_53_H_45_N_3_O_4_P_2_Ru (950.98): C, 66.94; H, 4.77; N, 4.42. Found: C, 66.98; H, 4.82; N, 4.46. ^1^H NMR (400.1 MHz, CD_2_Cl_2_, 25 °C): δ 8.72 (m, 1H; RuNC*H* of C_4_H_3_N_2_), 8.46 (m, 1H; NC*H* of C_4_H_3_N_2_), 8.22 (t, ^3^*J*(H,H) = 8.1 Hz, 1H; aromatic proton), 7.70–7.55 (m, 6H; aromatic protons), 7.53–7.44 (m, 4H; aromatic protons), 7.44–7.17 (m, 11H; aromatic protons), 7.06–6.88 (m, 4H; aromatic protons), 6.79 (m, 1H; aromatic proton), 6.71 (t, ^3^*J*(H,H) = 6.8 Hz, 1H; aromatic proton), 6.61 (d, ^3^*J*(H,H) = 7.2 Hz, 2H; aromatic protons), 6.43 (m, 2H; aromatic protons), 6.24 (d, ^3^*J*(H,H) = 8.5 Hz, 1H; aromatic proton), 5.89 (br s, 1H; NH_2_), 5.34 (br s, 1H; NH_2_ (overlapped with the solvent signal)), 4.21–4.04 (m, 2H; CH_2_N), 1.84 (s, 3H; OCOCH_3_), 1.68 (s, 3H; OCOCH_3_). ^13^C{^1^H} NMR (100.6 MHz, CD_2_Cl_2_, 25 °C): δ 182.7 (br s; O*C*OCH_3_), 182.6 (s; O*C*OCH_3_), 177.9 (d, ^3^*J*(C,P) = 3.8 Hz; N*C*CH_2_), 163.1 (d, ^3^*J*(C,P) = 2.9 Hz; RuN*C*H of C_4_H_3_N_2_), 157.2 (s; N*C*H of C_4_H_3_N_2_), 139.7–126.1 (m; aromatic carbon atoms), 119.5 (s; aromatic carbon atom), 52.1 (d, ^3^*J*(C,P) = 1.8 Hz; *C*H_2_N), 26.9 (s; OCO*C*H_3_), 26.5 (s; OCO*C*H_3_). ^31^P{^1^H} NMR (162.0 MHz, CD_2_Cl_2_, 25 °C): δ 53.7 (d, ^2^*J*(P,P) = 37.2 Hz), 41.9 (d, ^2^*J*(P,P) = 37.2 Hz).

### Synthesis of [Ru(κ^2^-OAc)((*R*)-BINAP)(ampyrim)]OAc (**15a**)

Complex **15a** was prepared by following the procedure used for the synthesis of **8a**, employing *trans*-[Ru(κ^1^-OAc)_2_((*R*)-BINAP)(ampyrim)] (**15**) (21 mg, 0.022 mmol) in place of **8**. The solution of **15** in CH_3_OH was stirred for 18 h at room temperature. The product consists of a mixture of two stereoisomers in about a 1:1 ratio. Yield: 18 mg (86%). Anal. Calcd for C_53_H_45_N_3_O_4_P_2_Ru (950.98): C, 66.94; H, 4.77; N, 4.42. Found: C, 66.90; H, 4.72; N, 4.37. ^1^H NMR (400.1 MHz, CD_3_OD, 25 °C): δ 8.94 (m, 1H; RuNC*H* of C_4_H_3_N_2_ first isomer), 8.75 (m, 1H; RuNC*H* of C_4_H_3_N_2_ second isomer), 8.61 (m, 1H; NC*H* of C_4_H_3_N_2_ first isomer), 8.49 (dd, ^3^*J*(H,H) = 4.3 Hz, ^4^*J*(H,H) = 1.3 Hz, 1H; NC*H* of C_4_H_3_N_2_ second isomer), 8.13–5.66 (m, 33H; aromatic protons both isomers), 4.63 (d, ^2^*J*(H,H) = 17.0 Hz, 1H; CH_2_N first isomer), 4.52 (dd, ^2^*J*(H,H) = 17.0 Hz, ^4^*J*(H,H) = 3.0 Hz, 1H; CH_2_N first isomer), 4.40 (d, ^2^*J*(H,H) = 17.7 Hz, 1H; CH_2_N second isomer), 4.07 (d, ^2^*J*(H,H) = 17.7 Hz, 1H; CH_2_N second isomer), 1.92 (s, 3H; OCOCH_3_ both isomers), 1.64 (s, 3H; OCOCH_3_ second isomer), 1.58 (s, 3H; OCOCH_3_ first isomer). ^13^C{^1^H} NMR (100.6 MHz, CD_3_OD, 25 °C): δ 190.2 (d, ^2^*J*(C,P) = 2.9 Hz; O*C*OCH_3_ first isomer), 189.9 (d, ^2^*J*(C,P) = 2.3 Hz; O*C*OCH_3_ second isomer), 179.8 (br s; O*C*OCH_3_ both isomers), 172.8 (br s,; N*C*CH_2_ second isomer), 172.4 (br s,; N*C*CH_2_ first isomer), 159.3 (s; RuN*C*H of C_4_H_3_N_2_ second isomer), 159.2 (s; RuN*C*H of C_4_H_3_N_2_ first isomer), 158.0 (s; N*C*H of C_4_H_3_N_2_ second isomer), 157.9 (s; N*C*H of C_4_H_3_N_2_ first isomer), 137.8–126.9 (m; aromatic carbon atoms both isomers), 122.2 (d, *J*(C,P) = 1.5 Hz; aromatic carbon atom first isomer), 120.2 (s; aromatic carbon atom second isomer), 54.0 (d, ^3^*J*(C,P) = 1.5 Hz; CH_2_N second isomer), 51.4 (d, ^3^*J*(C,P) = 2.4 Hz; CH_2_N first isomer), 24.2 (d; ^4^*J*(C,P) = 2.0 Hz; OCO*C*H_3_ second isomer), 24.0 (br s; OCO*C*H_3_ both isomers), 23.9 (br s; OCO*C*H_3_ first isomer). ^31^P{^1^H} NMR (162.0 MHz, CD_3_OD, 25 °C): δ 67.5 (d, ^2^*J*(P,P) = 37.2 Hz; first isomer), 59.9 (d, ^2^*J*(P,P) = 39.1 Hz; second isomer), 50.8 (d, ^2^*J*(P,P) = 37.2 Hz; first isomer), 50.3 (d, ^2^*J*(P,P) = 39.1 Hz; second isomer).

### Synthesis of *trans*-[Ru(κ^1^-OAc)_2_(dppb)(8-aminoquinoline)] (**16**)

[Ru(κ^2^-OAc)_2_(PPh_3_)_2_] (100 mg, 0.134 mmol) and dppb (58 mg, 0.136 mmol, 1.01 equiv) were dissolved in dichloromethane (1.5 mL) and stirred for 1 h at room temperature. 8-Aminoquinoline (28 mg, 0.194 mmol, 1.45 equiv) was added, and the resulting light orange solution was stirred for 1 h at room temperature. The solvent was removed under reduced pressure, and *n*-pentane (5 mL) was added to the residue, leading to a suspension, which was stirred for 10 min at room temperature. The resulting yellow precipitate was filtered, washed with *n*-pentane (3 × 5 mL), and dried under reduced pressure. Yield: 95 mg (90%). Anal. Calcd for C_41_H_42_N_2_O_4_P_2_Ru (789.81): C, 62.35; H, 5.36; N, 3.55. Found: C, 62.32; H, 5.40; N, 3.53. ^1^H NMR (400.1 MHz, CD_2_Cl_2_, 20 °C): δ 9.23 (t, ^3^*J*(H,H) = 4.1 Hz, 1H; NCH of C_9_H_6_N), 8.24 (m, 2H; NH_2_), 8.14 (dd, ^3^*J*(H,H) = 8.3 Hz, ^4^*J*(H,H) = 1.4 Hz, 1H; aromatic proton), 7.74 (td, ^3^*J*(H,H) = 8.5 Hz, ^4^*J*(H,H) = 1.5 Hz, 3H; aromatic protons), 7.64 (d, ^3^*J*(H,H) = 8.3 Hz, 1H; aromatic proton), 7.52–7.14 (m, 20H; aromatic protons), 7.02 (dd, ^3^*J*(H,H) = 8.3 Hz, ^3^*J*(H,H) = 5.0 Hz, 1H; aromatic proton), 2.84 (m, 2H; PCH_2_), 2.26 (*pseudo*-t, *J*(H,H) = 7.1 Hz, 2H; PCH_2_), 2.04–1.52 (m, 4H; CH_2_), 1.37 (s, 6H; OCOCH_3_). ^13^C{^1^H} NMR (50.3 MHz, CD_2_Cl_2_, 20 °C): δ 181.3 (br s; O*C*OCH_3_), 156.2 (d, ^3^*J*(C,P) = 3.9 Hz; N*C*H of C_9_H_6_N), 150.6–121.3 (m; aromatic carbon atoms), 34.1 (dd, ^1^*J*(C,P) = 27.3 Hz, ^3^*J*(C,P) = 2.6 Hz; P*C*H_2_), 27.8 (d, ^1^*J*(C,P) = 24.7 Hz; P*C*H_2_), 26.9 (s; PCH_2_*C*H_2_), 25.0 (s; OCO*C*H_3_), 19.3 (br s; PCH_2_*C*H_2_). ^31^P{^1^H} NMR (81.0 MHz, CD_2_Cl_2_, 20 °C): δ 50.5 (d, ^2^*J*(P,P) = 36.7 Hz), 37.2 (d, ^2^*J*(P,P) = 36.7 Hz).

### Synthesis of [Ru(κ^2^-OPiv)_2_(PPh_3_)_2_] (**17**)

Compound **17** was prepared by following a procedure different from that previously described.^44^ [RuCl_2_(PPh_3_)_3_] (1.00 g, 1.043 mmol) and sodium pivalate monohydrate (1.482 g, 10.43 mmol) were suspended in degassed *tert*-butyl alcohol (20 mL), and the mixture was heated at 70 °C for 2 h until a yellow precipitate was formed. The reaction mixture was cooled to room temperature, and diethyl ether (10 mL) was added. The suspension was stirred at room temperature for 10 min. The precipitate was filtered, washed with water (3 × 10 mL), methanol (2 × 4 mL), and diethyl ether (3 × 5 mL), and finally dried under reduced pressure, giving **17** as a pale orange powder. Yield: 650 mg (75%). Anal. Calcd for C_46_H_48_O_4_P_2_Ru (827.90): C, 66.74; H, 5.84. Found: C, 66.81; H, 5.86. ^1^H NMR (200.1 MHz, CDCl_3_, 20 °C): δ 7.33–7.19 (m, 6H; aromatic protons), 7.18–6.96 (m, 24H; aromatic protons), 0.78 (s, 18H; C(CH_3_)_3_). ^13^C{^1^H} NMR (50.3 MHz, CDCl_3_, 20 °C): δ 195.2 (br s; O*C*OC(CH_3_)_3_), 135.6–127.2 (m; aromatic carbon atoms), 39.4 (s; *C*(CH_3_)_3_), 26.6 (br s; C(*C*H_3_)_3_). ^31^P{^1^H} NMR (81.0 MHz, CDCl_3_, 20 °C): δ 64.0 (s).

### Synthesis of *trans*,*cis*-[Ru(κ^1^-OPiv)_2_(PPh_3_)_2_(ampy)] (**18**)

[Ru(κ^2^-OPiv)_2_(PPh_3_)_2_] (**17**) (50 mg, 0.0604 mmol) was dissolved in chloroform (1 mL), and ampy (6.5 μL, 0.0631 mmol, 1.04 equiv) was added. The solution was stirred for 10 min at room temperature. Addition of *n*-pentane (5 mL) afforded an orange precipitate, which was filtered, washed with *n*-pentane (3 × 2 mL), and dried under reduced pressure. Yield: 48 mg (85%). Anal. Calcd for C_52_H_56_N_2_O_4_P_2_Ru (936.05): C, 66.72; H, 6.03; N, 2.99. Found: C, 66.71; H, 6.06; N, 3.03. ^1^H NMR (200.1 MHz, CDCl_3_, 20 °C): δ 8.51 (br d, ^3^*J*(H,H) = 4.4 Hz, 1H; *ortho-*CH of C_5_H_4_N), 7.60–6.80 (m, 34H; aromatic protons and NH_2_), 6.55 (*pseudo*-t, *J*(H,H) = 6.4 Hz, 1H; aromatic proton), 4.03 (br m, 2H; CH_2_N), 0.85 (s, 18H; C(CH_3_)_3_). ^13^C{^1^H} NMR (50.3 MHz, CDCl_3_, 20 °C): δ 188.2 (d, ^3^*J*(C,P) = 1.4 Hz; O*C*OC(CH_3_)_3_), 166.5 (dd, ^3^*J*(C,P) = 2.7 Hz, ^3^*J*(C,P) = 1.5 Hz; N*C*CH_2_), 156.5 (d, ^3^*J*(C,P) = 3.8 Hz; N*C*H of C_5_H_4_N), 137.3–118.9 (m; aromatic carbon atoms), 50.9 (t, ^3^*J*(C,P) = 2.2 Hz; CH_2_N), 40.1 (s; *C*(CH_3_)_3_), 28.4 (s; C(*C*H_3_)_3_). ^31^P{^1^H} NMR (81.0 MHz, CDCl_3_, 20 °C): δ 45.8 (d, ^2^*J*(P,P) = 31.5 Hz), 38.5 (d, ^2^*J*(P,P) = 31.5 Hz).

### Synthesis of [Ru(κ^1^-OAc)(CNN^OMe^)(PPh_3_)_2_] (**19**)

The ligand HCNN^OMe^ (69.2 mg, 0.323 mmol, 1.2 equiv) and NEt_3_ (375 μL, 2.690 mmol, 10.0 equiv) were added to [Ru(κ^2^-OAc)_2_(PPh_3_)_2_] (200 mg, 0.269 mmol) in 2-propanol (2.5 mL), and the mixture was stirred at reflux for 12 h. The dark yellow precipitate was filtered, washed with *n*-pentane (5 × 3 mL), and dried under reduced pressure. Yield: 181 mg (75%). Anal. Calcd for C_51_H_46_N_2_O_3_P_2_Ru (897.96): C, 68.22; H, 5.16; N, 3.12. Found: C, 68.18; H, 5.20; N, 3.10. ^1^H NMR (400.1 MHz, CD_2_Cl_2_, 25 °C): δ 8.86 (m, 1H; NH_2_), 7.68 (s, 1H; aromatic proton), 7.64–7.04 (m, 24H; aromatic protons), 7.06–6.75 (m, 10H; aromatic protons), 6.59 (d, ^3^*J*(H,H) = 6.7 Hz, 1H; aromatic proton), 6.47 (d, ^3^*J*(H,H) = 6.0 Hz, 1H; aromatic proton), 4.09 (dd, ^2^*J*(H,H) = 17.3 Hz, ^3^*J*(H,H) = 6.0 Hz, 1H; CH_2_N), 3.53 (s, 3H; OCH_3_), 3.42 (m, 1H; CH_2_N), 1.92 (m, 1H; NH_2_), 1.09 (s, 3H; OCOCH_3_). ^13^C{^1^H} NMR (100.6 MHz, CD_2_Cl_2_, 25 °C): δ 185.5 (dd, ^2^*J*(C,P) = 14.3 Hz, ^2^*J*(C,P) = 8.4 Hz; CRu), 180.1 (br s; O*C*OCH_3_), 163.4 (s; N*C*C), 160.1 (s; C*C*OCH_3_), 157.3 (s; N*C*CH_2_), 142.8–108.6 (m; aromatic carbon atoms), 54.9 (s; *C*H_3_O), 51.0 ppm (d, ^2^*J*(C,P) = 2.2 Hz; *C*H_2_N), 25.1 (d; ^4^*J*(C,P) = 2.9 Hz; OCO*C*H_3_). ^31^P{^1^H} NMR (162.0 MHz, CD_2_Cl_2_, 25 °C): δ 57.2 (d, ^2^*J*(P,P) = 33.3 Hz), 52.9 (d, ^2^*J*(P,P) = 33.3 Hz).

### Synthesis of [Ru(κ^1^-OAc)(AMTP)(dppb)] (**20**)

#### Method 1

The ligand HAMTP (22 mg, 0.111 mmol, 1.06 equiv) and NEt_3_ (150 μL, 1.076 mmol, 10.2 equiv) were added to [Ru(κ^2^-OAc)_2_(dppb)] (68 mg, 0.105 mmol) in 2-propanol (1 mL), and the mixture was stirred at reflux for 2 h. The solvent was removed under reduced pressure, and the solid residue was washed with water (1 mL) and dried under reduced pressure for 2–3 days. Yield: 70 mg (85%). Anal. Calcd for C_43_H_44_N_2_O_2_P_2_Ru (783.85): C, 65.89; H, 5.66; N, 3.57. Found: C, 65.91; H, 5.60; N, 3.60. ^1^H NMR (200.1 MHz, toluene-*d*_8_, 20 °C): δ 8.65 (br s, 1H; NH_2_), 8.51 (t, ^3^*J*(H,H) = 9.1 Hz, 2H; aromatic protons), 8.06 (s, 1H; aromatic proton), 7.96 (t, ^3^*J*(H,H) = 7.6 Hz, 2H; aromatic protons), 7.73–7.05 (m, 12H; aromatic protons), 6.96 (m, 4H; aromatic protons), 6.72 (t, *J*(H,H) = 7.1 Hz, 2H; aromatic protons), 6.44 (d, ^3^*J*(H,H) = 6.0 Hz, 1H; aromatic protons), 6.32 (t, *J*(H,H) = 7.6 Hz, 2H; aromatic protons), 4.12 (dd, ^2^*J*(H,H) = 15.0 Hz, ^3^*J*(H,H) = 4.0 Hz, 1H; CH_2_N), 3.49 (m, 1H; CH_2_N), 3.40–3.04 (m, 2H; PCH_2_), 2.35 (s, 3H; CH_3_), 2.20–1.40 (m, 5H; CH_2_), 1.92 (s, 3H; CH_3_CO), 1.22 (m, 1H; NH_2_), 1.14 (m, 1H; CH_2_). ^31^P{^1^H} NMR (81.0 MHz, CD_2_Cl_2_, 20 °C): δ 60.8 (d, ^2^*J*(P,P) = 38.3 Hz), 44.6 (d, ^2^*J*(P,P) = 38.3 Hz). ^31^P{^1^H} NMR (81.0 MHz, toluene-*d*_8_, 20 °C): δ 60.7 (d, ^2^*J*(P,P) = 38.5 Hz), 44.5 (d, ^2^*J*(P,P) = 38.5 Hz).

#### Method 2

[Ru(κ^2^-OAc)_2_(PPh_3_)_2_] (100 mg, 0.134 mmol) and dppb (58 mg, 0.136 mmol, 1.01 equiv) were suspended in 2-propanol and refluxed for 1 h. The mixture was cooled to room temperature, and the ligand HAMTP (28 mg, 0.141 mmol, 1.05 equiv) and NEt_3_ (187 μL, 1.341 mmol, 10 equiv) were added. The mixture was then refluxed for a further 2 h. The solvent was removed under reduced pressure, and the solid residue was washed with water (1.5 mL) and dried under reduced pressure for 2–3 days. Yield: 48 mg (46%).

### Synthesis of [Ru(κ^1^-OAc)(AMBQ^Ph^)(dppb)] (**21**)

#### Method 1

The ligand HAMBQ^Ph^ (45.5 mg, 0.160 mmol, 1.03 equiv) and NEt_3_ (220 μL, 1.578 mmol, 10.2 equiv) were added to [Ru(κ^2^-OAc)_2_(dppb)] (100 mg, 0.155 mmol) in 2-propanol (1 mL), and the mixture was stirred at reflux for 3.5 h. The solvent was removed under reduced pressure and the residue added to *n*-pentane (5 mL). The suspension was stirred for 5 min, and the solid was filtered, washed with *n*-pentane (2 × 3 mL), and dried under reduced pressure. Yield: 80 mg (59%).

#### Method 2

[Ru(κ^2^-OAc)_2_(PPh_3_)_2_] (200 mg, 0.269 mmol) and dppb (115.8 mg, 0.272 mmol, 1.01 equiv) were suspended in 2-propanol (2.5 mL) and refluxed for 4 h. The mixture was cooled to room temperature, and the ligand HAMBQ^Ph^ (91.8 mg, 0.323 mmol, 1.20 equiv) and NEt_3_ (375 μL, 2.690 mmol, 10 equiv) were added. The mixture was then refluxed for further 12 h. The obtained suspension was cooled to room temperature and the orange solid was filtered, washed with 2-propanol (2 mL), *n*-pentane (4 × 5 mL) and dried under reduced pressure. Yield: 152 mg (65%). Anal. Calcd for C_50_H_46_N_2_O_2_P_2_Ru (869.95): C, 69.03; H, 5.33; N, 3.22. Found: C, 69.01; H, 5.36; N, 3.23. ^1^H NMR (200.1 MHz, CD_2_Cl_2_, 20 °C): δ 8.61 (m, 1H; NH_2_), 8.22 (*pseudo*-t, *J*(H,H) = 7.6 Hz, 2H; aromatic protons), 7.91 (d, ^3^*J*(H,H) = 7.1 Hz, 1H; aromatic proton), 7.80–7.15 (m, 21H; aromatic protons), 7.60 (d, ^3^*J*(H,H) = 8.5 Hz, 1H; H-5 benzo[*h*]quinoline), 6.96 (s, 1H; H-3 benzo[*h*]quinoline), 6.54 (t, ^3^*J*(H,H) = 7.4 Hz, 1H; aromatic proton), 6.22 (t, ^3^*J*(H,H) = 6.8 Hz, 2H; aromatic protons), 5.55 (t, ^3^*J*(H,H) = 8.4 Hz, 2H; aromatic protons), 4.45 (dd, ^2^*J*(H,H) = 16.5 Hz, ^3^*J*(H,H) = 5.2 Hz, 1H; CH_2_N), 3.97 (m, 1H; CH_2_N), 3.18 (m, 1H; PCH_2_), 2.86 (m, 1H; PCH_2_), 2.50 (m, 2H; PCH_2_), 2.20–1.57 (m, 4H; CH_2_), 1.33 (s, 3H; OCOCH_3_), 0.98 (m, 1H; NH_2_). ^13^C{^1^H} NMR (100.6 MHz, CD_2_Cl_2_, 25 °C): δ 180.4 (br s; O*C*OCH_3_), 180.3 (dd, ^2^*J*(C,P) = 16.1 Hz, ^2^*J*(C,P) = 8.8 Hz; CRu), 157.5 (s; N*C*C), 152.7 (br s; N*C*CH_2_), 146.6–116.2 (m; aromatic carbon atoms), 52.5 (br s; *C*H_2_N), 31.1 (dd, ^1^*J*(C,P)= 24.9 Hz, ^3^*J*(C,P) = 1.5 Hz; *C*H_2_P), 30.7 (d, ^1^*J*(C,P) = 32.3 Hz; *C*H_2_P), 26.0 (d, ^2^*J*(C,P) = 1.5 Hz; *C*H_2_CH_2_P), 25.7 (d; ^4^*J*(C,P) = 3.8 Hz; OCO*C*H_3_), 22.0 (t, ^2^*J*(C,P) = 2.2 Hz; *C*H_2_CH_2_P). ^31^P{^1^H} NMR (162.0 MHz, CD_2_Cl_2_, 20 °C): δ 59.8 (d, ^2^*J*(P,P) = 37.9 Hz), 44.9 (d, ^2^*J*(P,P) = 37.9 Hz).

### Typical Procedure for TH of Ketones

The ruthenium catalyst solution used for TH was prepared by dissolving the complexes **7**–**11**, **16**, and **21** (0.02 mmol) in 2-propanol (5 mL). The catalyst solution (125 μL, 0.5 μmol) and a 0.1 M solution of NaO*i*Pr (200 μL, 20 μmol) in 2-propanol were added subsequently to the carbonyl compound solution (1.0 mmol) in 2-propanol (final volume 10 mL), and the resulting mixture was heated under reflux. The reaction mixture was sampled by removing an aliquot, which was quenched by addition of diethyl ether (1/1 v/v), filtered over a short silica pad, and submitted to GC analysis. The base addition was considered as the start time of the reaction. The S/C molar ratio was 2000:1, whereas the base concentration was 2 mol % with respect to the substrate (0.1 M). The same procedure was followed for TH reactions with other S/C ratios (in the range 2000–10000), using the appropriate amount of catalysts and 2-propanol.

### Typical Procedure for HY of Ketones and Aldehydes

The HY reactions were performed in an eight-vessel Endeavor Biotage apparatus. The vessels were charged with the catalysts **7**, **9**, **10**, and **19** (5.0 μmol), loaded with 5 bar of N_2_, and slowly vented (five times). The carbonyl compounds (5 mmol) and a KO*t*Bu solution (1 mL, 0.1 mmol, 0.1 M) in methanol or ethanol were added. Further addition of the solvent (methanol or ethanol) led to a 2 M carbonyl compound solution. The vessels were purged with N_2_ and H_2_ (three times each), and then the system was charged with H_2_ (20 or 30 bar) and heated to 40 or 50 °C for the required time (16 h). The S/C molar ratio was 1000:1, whereas the base concentration was 2 mol %. A similar method was applied for the reactions with other S/C ratios (in the range 1000–10000), using the appropriate amount of catalysts and solvent. The reaction vessels were then cooled to room temperature, vented, and purged three times with N_2_. A drop of the reaction mixture was diluted with 1 mL of methanol and analyzed by GC.

### Single-Crystal X-ray Crystallographic Structure Determination of Compound **7**

Single crystals of complex **7** were obtained by slow cooling of a concentrated solution of the species in CH_2_Cl_2_. X-ray diffraction data were collected with a Bruker kappa APEX-II CCD diffractometer equipped with a rotating anode (Bruker AXS, FR591) by using graphite-monochromated Mo Kα radiation (λ = 0.71073 Å). For additional details of the collection and refinement of data, see the Supporting Information.

## Supporting Information Available

NMR data of the isolated complexes and X-ray crystallographic details of compound **7** (PDF)

## Accession Codes

CCDC 2058063 contains the supplementary crystallographic data for this paper. These data can be obtained free of charge via www.ccdc.cam.ac.uk/data_request/cif, or by emailing data_request@ccdc.cam.ac.uk, or by contacting The Cambridge Crystallographic Data Centre, 12 Union Road, Cambridge CB2 1EZ, UK; fax: +44 1223 336033.
