# Supplementary material for: Preparation of Neutral trans - cis [Ru(O2CR)2P2(NN)], Cationic [Ru(O2CR)P2(NN)](O2CR) and Pincer [Ru(O2CR)(CNN)P2] (P = PPh3, P2 = diphosphine) Carboxylate Complexes and their Application in the Catalytic Carbonyl Compounds Reduction
Source: Organometallics. 2021 Apr 14;40(8):1086–103. doi: 10.1021/acs.organomet.1c00059 (PMC8155570; doi:10.1021/acs.organomet.1c00059)
Supplement: Supplementary file 1 — om1c00059_si_001.pdf [file om1c00059_si_001.pdf]

Preparation of Neutral *trans - cis* [Ru($O_2CR)_2P_2$(NN)], Cationic [Ru(O_2CR)P_2(NN)]O_2CR and Pincer [Ru(O_2CR)(CNN)P_2] (P = PPh_3, P_2 = diphosphine) Carboxylate Complexes and their Application in the Catalytic Carbonyl Compounds Reduction

Salvatore Baldino,[#,§] Steven Giboulot,[#, £] Denise Lovison,[#] Hans Günter Nedden,[£] Alexander Pöthig,[†] Antonio Zanotti-Gerosa,[£] Daniele Zuccaccia,[#] Maurizio Ballico,[#,*] Walter Baratta[#,*]

[#]Dipartimento DI4A - Università di Udine, Via del Cotonificio 108,
I-33100 Udine, Italy
[§]Dipartimento di Chimica, Università di Torino, Via Pietro Giuria, 7,
I-10125 Torino, Italy
[†]Department of Chemistry & Catalysis Research Center,
TUM - Lichtenbergstraße 4, 85747 Garching b. München, Germany
[£] Johnson Matthey, 28 Cambridge Science Park, Milton Road
Cambridge, CB4 0FP, United Kingdom

Supporting Information

Table of Contents:

Figure S1. $^{31}P\{^1H\}$ NMR spectrum of *trans,cis*-[Ru(η^1-OAc)$_2$(PPh_3)$_2$(en)] (**1**) Pag. S10

Figure S2. ^1H NMR spectrum of *trans,cis*-[Ru(η^1-OAc)$_2$(PPh_3)$_2$(en)] (**1**) Pag. S11

Figure S3. $^{13}C\{^1H\}$ DEPTQ NMR spectrum of *trans,cis*-[Ru(η^1-OAc)$_2$(PPh_3)$_2$(en)] (**1**) Pag. S12

Figure S4. ^1H-^{13}C HSQC 2D NMR spectrum of *trans,cis*-[Ru(η^1-OAc)$_2$(PPh_3)$_2$(en)] (**1**) Pag. S13

Figure S5. $^{31}P\{^1H\}$ NMR spectrum of *trans,cis*-[Ru(η^1-OAc)$_2$(PPh_3)$_2$(ampy)] (**2**) Pag. S14

Figure S6. ^1H NMR spectrum of *trans,cis*-[Ru(η^1-OAc)$_2$(PPh_3)$_2$(ampy)] (**2**) Pag. S15

Figure S7. $^{13}C\{^{1}H\}$ PENDANT NMR spectrum of *trans,cis*-[Ru(η^1-OAc)$_2$(PPh$_3$)$_2$(ampy)] (**2**) Pag. S16

Figure S8. $^{31}P\{^{1}H\}$ NMR spectrum of the mixture 3:2 of the cationic species *cis*-[Ru(η^2-OAc)(PPh$_3$)$_2$(ampy)]OAc (**2a**) and *cis,cis*-[Ru(η^1-OAc)$_2$(PPh$_3$)$_2$(ampy)] (**2b**) Pag. S17

Figure S9. ^{1}H NMR spectrum of the mixture 3:2 of the cationic species *cis*-[Ru(η^2-OAc)(PPh$_3$)$_2$(ampy)]OAc (**2a**) and *cis,cis*-[Ru(η^1-OAc)$_2$(PPh$_3$)$_2$(ampy)] (**2b**) Pag. S18

Figure S10. Effect of the addition of NaOAc (3.5 equiv) to the mixture 3:2 of the cationic species *cis*-[Ru(η^2-OAc)(PPh$_3$)$_2$(ampy)]OAc (**2a**) and *cis*,*cis*-[Ru(η^1-OAc)$_2$(PPh$_3$)$_2$(ampy)] (**2b**) in the methyl acetate region of the ^{1}H NMR spectrum Pag. S19

Figure S11. $^{13}C\{^{1}H\}$ DEPTQ NMR spectrum of the mixture 3:2 of the cationic species *cis*-[Ru(η^2-OAc)(PPh$_3$)$_2$(ampy)]OAc (**2a**) and *cis,cis*-[Ru(η^1-OAc)$_2$(PPh$_3$)$_2$(ampy)] (**2b**) Pag. S20

Figure S12. ^{1}H-^{13}C HSQC 2D NMR spectrum of of the mixture 3:2 of the cationic species *cis*-[Ru(η^2-OAc)(PPh$_3$)$_2$(ampy)]OAc (**2a**) and *cis,cis*-[Ru(η^1-OAc)$_2$(PPh$_3$)$_2$(ampy)] (**2b**) Pag. S21

Figure S13. $^{31}P\{^{1}H\}$ NMR spectrum of *trans,cis*-[Ru(η^1-OAc)$_2$(PPh$_3$)$_2$(ampyrim)] (**3**) Pag. S22

Figure S14. ^{1}H NMR spectrum of *trans*,*cis*-[Ru(η^1-OAc)$_2$(PPh$_3$)$_2$(ampyrim)] (**3**) Pag. S23

Figure S15. $^{13}C\{^{1}H\}$ PENDANT NMR spectrum of *trans*,*cis*-[Ru(η^1-OAc)$_2$(PPh$_3$)$_2$(ampyrim)] (**3**) Pag. S24

Figure S16. $^{31}P\{^{1}H\}$ NMR spectrum of the mixture 2:1 of the cationic species *cis*-[Ru(η^2-OAc)(PPh$_3$)$_2$(ampyrim)]OAc (**3a**) and *cis*,*cis*-[Ru(η^1-OAc)$_2$(PPh$_3$)$_2$(ampyrim)] (**3b**) Pag. S25

Figure S17. ^{1}H NMR spectrum of the mixture 2:1 of the cationic species *cis*-[Ru(η^2-OAc)(PPh$_3$)$_2$(ampyrim)]OAc (**3a**) and *cis*,*cis*-[Ru(η^1-OAc)$_2$(PPh$_3$)$_2$(ampyrim)] (**3b**) Pag. S26

Figure S18. $^{13}C\{^{1}H\}$ DEPTQ NMR spectrum of the mixture 2:1 of the cationic species *cis*-[Ru(η^2-OAc)(PPh$_3$)$_2$(ampyrim)]OAc (**3a**) and *cis*,*cis*-[Ru(η^1-OAc)$_2$(PPh$_3$)$_2$(ampyrim)] (**3b**) Pag. S27

Figure S19. $^{31}P\{^{1}H\}$ NMR spectrum of *trans*-[Ru(η^1-OAc)$_2$(dppm)$_2$] (**4**) Pag. S28

Figure S20. ^{1}H NMR spectrum of *trans*-[Ru(η^1-OAc)$_2$(dppm)$_2$] (**4**) Pag. S29

Figure S21. ^{31}P{^{1}H} NMR spectrum of *trans*-[Ru(η^1-OAc)$_2$(dppe)$_2$] (**5**) Pag. S30

Figure S22. ^{1}H NMR spectrum of *trans*-[Ru(η^1-OAc)$_2$(dppe)$_2$] (**5**) Pag. S31

Figure S23. ^{31}P{^{1}H} NMR spectrum of the 2:1 mixture of *trans*-[Ru(η^1-OAc)$_2$(dppm)(ampy)] and *trans*-[Ru(η^1-OAc)$_2$(dppm)$_2$] (**4**) in CD_2Cl_2 Pag. S32

Figure S24. ^{1}H NMR spectrum of the 2:1 mixture of *trans*-[Ru(η^1-OAc)$_2$(dppm)(ampy)] and *trans*-[Ru(η^1-OAc)$_2$(dppm)$_2$] (**4**) in CD_2Cl_2 Pag. S33

Figure S25. ^{31}P{^{1}H} NMR spectrum of *cis*-[Ru(η^1-OAc)$_2$(dppm)(ampy)] (**6**) in CD_3OD Pag. S34

Figure S26. ^{1}H NMR spectrum of *cis*-[Ru(η^1-OAc)$_2$(dppm)(ampy)] (**6**) in CD_3OD Pag. S35

Figure S27. Effect of the addition of NaOAc (3.5 equiv) to *cis*-[Ru(η^1-OAc)$_2$(dppm)(ampy)] (**6**) in the methyl acetate region of the ^{1}H NMR spectrum in CD_3OD Pag. S36

Figure S28. ^{13}C{^{1}H} DEPTQ NMR spectrum of *cis*-[Ru(η^1-OAc)$_2$(dppm)(ampy)] (**6**) in CD_3OD Pag. S37

Figure S29. ^{31}P{^{1}H} NMR spectrum of *cis*-[Ru(η^1-OAc)$_2$(dppm)(ampy)] (**6**) in $CDCl_3$ Pag. S38

Figure S30. ^{1}H NMR spectrum of *cis*-[Ru(η^1-OAc)$_2$(dppm)(ampy)] (**6**) in $CDCl_3$ Pag. S39

Figure S31. ^{31}P{^{1}H} NMR spectrum of the mixture of [Ru(OAc)$_2$(dppe)(ampy)] isomers and uncharacterized products Pag. S40

Figure S32. ^{1}H NMR spectrum of the mixture of [Ru(OAc)$_2$(dppe)(ampy)] isomers and uncharacterized products Pag. S41

Figure S33. ^{31}P{^{1}H} NMR spectrum of *trans*-[Ru(η^1-OAc)$_2$(dppf)(en)] (**7**) Pag. S42

Figure S34. ^{1}H NMR spectrum of *trans*-[Ru(η^1-OAc)$_2$(dppf)(en)] (**7**) Pag. S43

Figure S35. $^{13}C\{^1H\}$ PENDANT NMR spectrum of *trans*-[Ru(η^1-OAc)$_2$(dppf)(en)] (**7**) Pag. S44

Figure S36. $^{31}P\{^1H\}$ NMR spectrum of *trans*-[Ru(η^1-OAc)$_2$(dppp)(ampy)] (**8**) Pag. S45

Figure S37. ^1H NMR spectrum of *trans*-[Ru(η^1-OAc)$_2$(dppp)(ampy)] (**8**) Pag. S46

Figure S38. $^{13}C\{^1H\}$ PENDANT NMR spectrum of *trans*-[Ru(η^1-OAc)$_2$(dppp)(ampy)] (**8**) Pag. S47

Figure S39. $^{31}P\{^1H\}$ NMR spectrum of [Ru(η^2-OAc)(dppp)(ampy)]OAc (**8a**) Pag. S48

Figure S40. ^1H NMR spectrum of [Ru(η^2-OAc)(dppp)(ampy)]OAc (**8a**) Pag. S49

Figure S41. $^{13}C\{^1H\}$ DEPTQ NMR spectrum of [Ru(η^2-OAc)(dppp)(ampy)]OAc (**8a**) Pag. S50

Figure S42. $^{31}P\{^1H\}$ NMR spectrum of *trans*-[Ru(η^1-OAc)$_2$(dppb)(ampy)] (**9**) Pag. S51

Figure S43. ^1H NMR spectrum of *trans*-[Ru(η^1-OAc)$_2$(dppb)(ampy)] (**9**) Pag. S52

Figure S44. $^{13}C\{^1H\}$ PENDANT NMR spectrum of *trans*-[Ru(η^1-OAc)$_2$(dppb)(ampy)] (**9**) Pag. S53

Figure S45. $^{31}P\{^1H\}$ NMR spectrum of [Ru(η^2-OAc)(dppb)(ampy)]OAc (**9a**) Pag. S54

Figure S46. ^1H NMR spectrum of [Ru(η^2-OAc)(dppb)(ampy)]OAc (**9a**) Pag. S55

Figure S47. Effect of the addition of NaOAc (2-8 equiv) to [Ru(η^2-OAc)(dppb)(ampy)]OAc (**9a**) in the methyl acetate region of the ^1H NMR spectrum Pag. S56

Figure S48. $^{13}C\{^1H\}$ DEPTQ NMR spectrum of [Ru(η^2-OAc)(dppb)(ampy)]OAc (**9a**) Pag. S57

Figure S49. $^{31}P\{^1H\}$ NMR spectrum of *trans*-[Ru(η^1-OAc)$_2$(dppf)(ampy)] (**10**) Pag. S58

Figure S50. ^1H NMR spectrum of *trans*-[Ru(η^1-OAc)$_2$(dppf)(ampy)] (**10**) Pag. S59

Figure S51. $^{13}C\{^1H\}$ PENDANT NMR spectrum of *trans*-[Ru(η^1-OAc)$_2$(dppf)(ampy)] (**10**) Pag. S60

Figure S52. $^{31}P\{^1H\}$ NMR spectrum of [Ru(η^2-OAc)(dppf)(ampy)]OAc (**10a**) Pag. S61

Figure S53. ^1H NMR spectrum of [Ru(η^2-OAc)(dppf)(ampy)]OAc (**10a**) Pag. S62

Figure S54. ^1H-^1H COSY 2D NMR spectrumof [Ru(η^2-OAc)(dppf)(ampy)]OAc (**10a**) Pag. S63

Figure S55. $^{13}C\{^{1}H\}$ DEPTQ NMR spectrum of [Ru(η^2-OAc)(dppf)(ampy)]OAc (**10a**) Pag. S64

Figure S56. ^{1}H-^{31}P HMBC 2D NMR spectrum of [Ru(η^2-OAc)(dppf)(ampy)]OAc (**10a**) Pag. S65

Figure S57. ^{1}H-^{13}C HSQC 2D NMR spectrum of [Ru(η^2-OAc)(dppf)(ampy)]OAc (**10a**) Pag. S66

Figure S58. $^{31}P\{^{1}H\}$ NMR spectrum of *trans*-[Ru(η^1-OAc)$_2$((*R*)-BINAP)(ampy)] (**11**) Pag. S67

Figure S59. ^{1}H NMR spectrum of *trans*-[Ru(η^1-OAc)$_2$((*R*)-BINAP)(ampy)] (**11**) Pag. S68

Figure S60. $^{13}C\{^{1}H\}$ DEPTQ NMR spectrum of *trans*-[Ru(η^1-OAc)$_2$((*R*)-BINAP)(ampy)] (**11**) Pag. S69

Figure S61. ^{1}H-^{15}N HSQC 2D NMR spectrum of *trans*-[Ru(η^1-OAc)$_2$((*R*)-BINAP)(ampy)] (**11**) Pag. S70

Figure S62. ^{1}H-^{13}C HSQC 2D NMR spectrum of *trans*-[Ru(η^1-OAc)$_2$((*R*)-BINAP)(ampy)] (**11**) Pag. S71

Figure S63. $^{31}P\{^{1}H\}$ NMR spectrum of [Ru(η^2-OAc)((*R*)-BINAP)(ampy)]OAc (**11a**) Pag. S72

Figure S64. ^{1}H NMR spectrum of [Ru(η^2-OAc)((*R*)-BINAP)(ampy)]OAc (**11a**) Pag. S73

Figure S65. $^{13}C\{^{1}H\}$ DEPTQ NMR spectrum of [Ru(η^2-OAc)((*R*)-BINAP)(ampy)]OAc (**11a**) Pag. S74

Figure S66. $^{31}P\{^{1}H\}$ NMR spectrum of *trans*-[Ru(η^1-OAc)$_2$(dppp)(ampyrim)] (**12**) Pag. S75

Figure S67. ^{1}H NMR spectrum of *trans*-[Ru(η^1-OAc)$_2$(dppp)(ampyrim)] (**12**) Pag. S76

Figure S68. $^{13}C\{^{1}H\}$ DEPTQ NMR spectrum of *trans*-[Ru(η^1-OAc)$_2$(dppp)(ampyrim)] (**12**) Pag. S77

Figure S69. ^{1}H-^{13}C HSQC 2D NMR spectrum of *trans*-[Ru(η^1-OAc)$_2$(dppp)(ampyrim)] (**12**) Pag. S78

Figure S70. $^{31}P\{^{1}H\}$ NMR spectrum of [Ru(η^2-OAc)(dppp)(ampyrim)]OAc (**12a**) Pag. S79

Figure S71. ^{1}H NMR spectrum of [Ru(η^2-OAc)(dppp)(ampyrim)]OAc (**12a**) Pag. S80

Figure S72. ^{13}C{^{1}H} DEPTQ NMR spectrum of [Ru(η^2-OAc)(dppp)(ampyrim)]OAc (**12a**) Pag. S81

Figure S73. ^{31}P{^{1}H} NMR spectrum of *trans*-[Ru(η^1-OAc)$_2$(dppb)(ampyrim)] (**13**) Pag. S82

Figure S74. ^{1}H NMR spectrum of *trans*-[Ru(η^1-OAc)$_2$(dppb)(ampyrim)] (**13**) Pag. S83

Figure S75. ^{13}C{^{1}H} DEPTQ NMR spectrum of *trans*-[Ru(η^1-OAc)$_2$(dppb)(ampyrim)] (**13**) Pag. S84

Figure S76. ^{1}H-^{1}H COSY 2D NMR spectrum of *trans*-[Ru(η^1-OAc)$_2$(dppb)(ampyrim)] (**13**) Pag. S85

Figure S77. ^{1}H-^{13}C HSQC 2D NMR spectrum of *trans*-[Ru(η^1-OAc)$_2$(dppb)(ampyrim)] (**13**) Pag. S86

Figure S78. ^{1}H-^{31}P HMBC 2D NMR spectrum of *trans*-[Ru(η^1-OAc)$_2$(dppb)(ampyrim)] (**13**) Pag. S87

Figure S79. ^{31}P{^{1}H} NMR spectrum of [Ru(η^2-OAc)(dppb)(ampyrim)]OAc (**13a**) Pag. S88

Figure S80. ^{1}H NMR spectrum of [Ru(η^2-OAc)(dppb)(ampyrim)]OAc (**13a**) Pag. S89

Figure S81. ^{13}C{^{1}H} DEPTQ NMR spectrum of [Ru(η^2-OAc)(dppb)(ampyrim)]OAc (**13a**) Pag. S90

Figure S82. ^{1}H-^{13}C HSQC 2D NMR spectrum of [Ru(η^2-OAc)(dppb)(ampyrim)]OAc (**13a**) Pag. S91

Figure S83. ^{31}P{^{1}H} NMR spectrum of *trans*-[Ru(η^1-OAc)$_2$(dppf)(ampyrim)] (**14**) Pag. S92

Figure S84. ^{1}H NMR spectrum of *trans*-[Ru(η^1-OAc)$_2$(dppf)(ampyrim)] (**14**) Pag. S93

Figure S85. ^{13}C{^{1}H} DEPTQ NMR spectrum of *trans*-[Ru(η^1-OAc)$_2$(dppf)(ampyrim)] (**14**) Pag. S94

Figure S86. ^{1}H-^{1}H COSY 2D NMR spectrum of *trans*-[Ru(η^1-OAc)$_2$(dppf)(ampyrim)] (**14**) Pag. S95

Figure S87. ^{1}H-^{13}C HSQC 2D NMR spectrum of *trans*-[Ru(η^{1}-OAc)$_2$(dppf)(ampyrim)] (**14**)

Pag. S96

Figure S88. ^{31}P{^{1}H} NMR spectrum of [Ru(η^{2}-OAc)(dppf)(ampyrim)]OAc (**14a**) Pag. S97

Figure S89. ^{1}H NMR spectrum of [Ru(η^{2}-OAc)(dppf)(ampyrim)]OAc (**14a**)) Pag. S98

Figure S90. ^{13}C{^{1}H} DEPTQ NMR spectrum of [Ru(η^{2}-OAc)(dppf)(ampyrim)]OAc (**14a**)

Pag. S99

Figure S91. ^{1}H-^{1}H COSY 2D NMR spectrum of [Ru(η^{2}-OAc)(dppf)(ampyrim)]OAc (**14a**)

Pag. S100

Figure S92. ^{1}H-^{13}C HSQC 2D NMR spectrum of [Ru(η^{2}-OAc)(dppf)(ampyrim)]OAc (**14a**)

Pag. S101

Figure S93. ^{1}H-^{31}P HMBC 2D NMR spectrum of [Ru(η^{2}-OAc)(dppf)(ampyrim)]OAc (**14a**)

Pag. S102

Figure S94. ^{31}P{^{1}H} NMR spectrum of *trans*-[Ru(η^{1}-OAc)$_2$((*R*)-BINAP)(ampyrim)] (**15**)

Pag. S103

Figure S95. ^{1}H NMR spectrum of *trans*-[Ru(η^{1}-OAc)$_2$((*R*)-BINAP)(ampyrim)] (**15**) Pag. S104

Figure S96. ^{13}C{^{1}H} DEPTQ NMR spectrum of *trans*-[Ru(η^{1}-OAc)$_2$((*R*)-BINAP)(ampyrim)] (**15**)

Pag. S105

Figure S97. ^{1}H-^{1}H COSY 2D NMR spectrum of *trans*-[Ru(η^{1}-OAc)$_2$((*R*)-BINAP)(ampyrim)] (**15**)

Pag. S106

Figure S98. ^{1}H-^{13}C HSQC 2D NMR spectrum of *trans*-[Ru(η^{1}-OAc)$_2$((*R*)-BINAP)(ampyrim)] (**15**)

Pag. S107

Figure S99. ^{31}P{^{1}H} NMR spectrum of [Ru(η^{2}-OAc)((*R*)-BINAP)(ampyrim)]OAc (**15a**)

Pag. S108

Figure S100. ^{1}H NMR spectrum of [Ru(η^{2}-OAc)((*R*)-BINAP)(ampyrim)]OAc (**15a**) Pag. S109

Figure S101. ^{13}C{^{1}H} DEPTQ NMR spectrum of [Ru(η^{2}-OAc)((*R*)-BINAP)(ampyrim)]OAc (**15a**)

Pag. S110

Figure S102. ^{1}H-^{13}C HSQC 2D NMR spectrum of [Ru(η^2-OAc)((*R*)-BINAP)(ampyrim)]OAc (**15a**) Pag. S111

Figure S103. ^{31}P{^{1}H} NMR spectrum of *trans*-[Ru(η^1-OAc)$_2$(dppb)(8-aminoquinoline)] (**16**) Pag. S112

Figure S104. ^{1}H NMR spectrum of *trans*-[Ru(η^1-OAc)$_2$(dppb)(8-aminoquinoline)] (**16**) Pag. S113

Figure S105. ^{13}C{^{1}H} PENDANT NMR spectrum of *trans*-[Ru(η^1-OAc)$_2$(dppb)(8-aminoquinoline)] (**16**) Pag. S114

Figure S106. ^{31}P{^{1}H} NMR spectrum of [Ru(η^2-OPiv)$_2$(PPh$_3$)$_2$] (**17**) Pag. S115

Figure S107. ^{1}H NMR spectrum of [Ru(η^2-OPiv)$_2$(PPh$_3$)$_2$] (**17**) Pag. S116

Figure S108. ^{13}C{^{1}H} PENDANT NMR spectrum of [Ru(η^2-OPiv)$_2$(PPh$_3$)$_2$] (**17**) Pag. S117

Figure S109. ^{31}P{^{1}H} NMR spectrum of *trans,cis*-[Ru(η^1-OPiv)$_2$(PPh$_3$)$_2$(ampy)] (**18**) Pag. S118

Figure S110. ^{1}H NMR spectrum of *trans,cis*-[Ru(η^1-OPiv)$_2$(PP$_3$)$_2$(ampy)] (**18**) Pag. S119

Figure S111. ^{13}C{^{1}H} PENDANT NMR spectrum of *trans,cis*-[Ru(η^1-OPiv)$_2$(PPh$_3$)$_2$(ampy)] (**18**) Pag. S120

Figure S112. ^{31}P{^{1}H} NMR spectrum of [Ru(η^1-OAc)(CNN^{OMe})(PPh$_3$)$_2$] (**19**) Pag. S121

Figure S113. ^{1}H NMR spectrum of [Ru(η^1-OAc)(CNN^{OMe})(PPh$_3$)$_2$] (**19**) Pag. S122

Figure S114. ^{13}C{^{1}H} DEPTQ NMR spectrum of [Ru(η^1-OAc)(CNN^{OMe})(PPh$_3$)$_2$] (**19**) Pag. S123

Figure S115. ^{1}H-^{1}H COSY 2D NMR spectrum of [Ru(η^1-OAc)(CNN^{OMe})(PPh$_3$)$_2$] (**19**) Pag. S124

Figure S116. ^{1}H-^{13}C HSQC 2D NMR spectrum of [Ru(η^1-OAc)(CNN^{OMe})(PPh$_3$)$_2$] (**19**) Pag. S125

Figure S117. ^{1}H-^{15}N HSQC 2D NMR spectrum of [Ru(η^1-OAc)(CNN^{OMe})(PPh$_3$)$_2$] (**19**) Pag. S126

Figure S118. $^{31}P\{^1H\}$ NMR spectrum of [Ru(η^1-OAc)(CNN)(dppb)] (**20**) Pag. S127

Figure S119. ^1H NMR spectrum of [Ru(η^1-OAc)(CNN)(dppb)] (**20**) Pag. S128

Figure S120. $^{31}P\{^1H\}$ NMR spectrum of [Ru(η^1-OAc)(AMBQ^{Ph})(dppb)] (**21**) Pag. S129

Figure S121. ^1H NMR spectrum of [Ru(η^1-OAc)(AMBQ^{Ph})(dppb)] (**21**) Pag. S130

Figure S122. $^{13}C\{^1H\}$ DEPTQ NMR spectrum of [Ru(η^1-OAc)(AMBQ^{Ph})(dppb)] (**21**) Pag. S131

Figure S123. ^1H-^1H COSY 2D NMR spectrum of [Ru(η^1-OAc)(AMBQ^{Ph})(dppb)] (**21**) Pag. S132

Figure S124. ^1H-^{13}C HSQC 2D NMR spectrum of [Ru(η^1-OAc)(AMBQ^{Ph})(dppb)] (**21**) Pag. S133

Figure S125. ^1H-^{15}N HSQC 2D NMR spectrum of [Ru(η^1-OAc)(AMBQ^{Ph})(dppb)] (**21**) Pag. S134

Figure S126. ^1H-^{31}P HMBC 2D NMR spectrum of [Ru(η^1-OAc)(AMBQ^{Ph})(dppb)] (**21**) Pag. S135

Figure S127. ^1H-^{31}P HMBC 2D NMR spectrum (hydridic region) of the mixture 2.8:1 of *trans*- and *cis*-[RuH$(PPh_3)_2$(CNN^{OMe})] in iPrOH/toluene-*d*^{8} Pag. S136

Single crystal X-ray structure determination of Compound 7. General data. Pag. S137

Figure S128. Molecular structure of complex **7** Pag. S138

Single crystal X-ray structure determination of complex 7. Detailed crystallographic data. Pag. S139

References Pag. S141

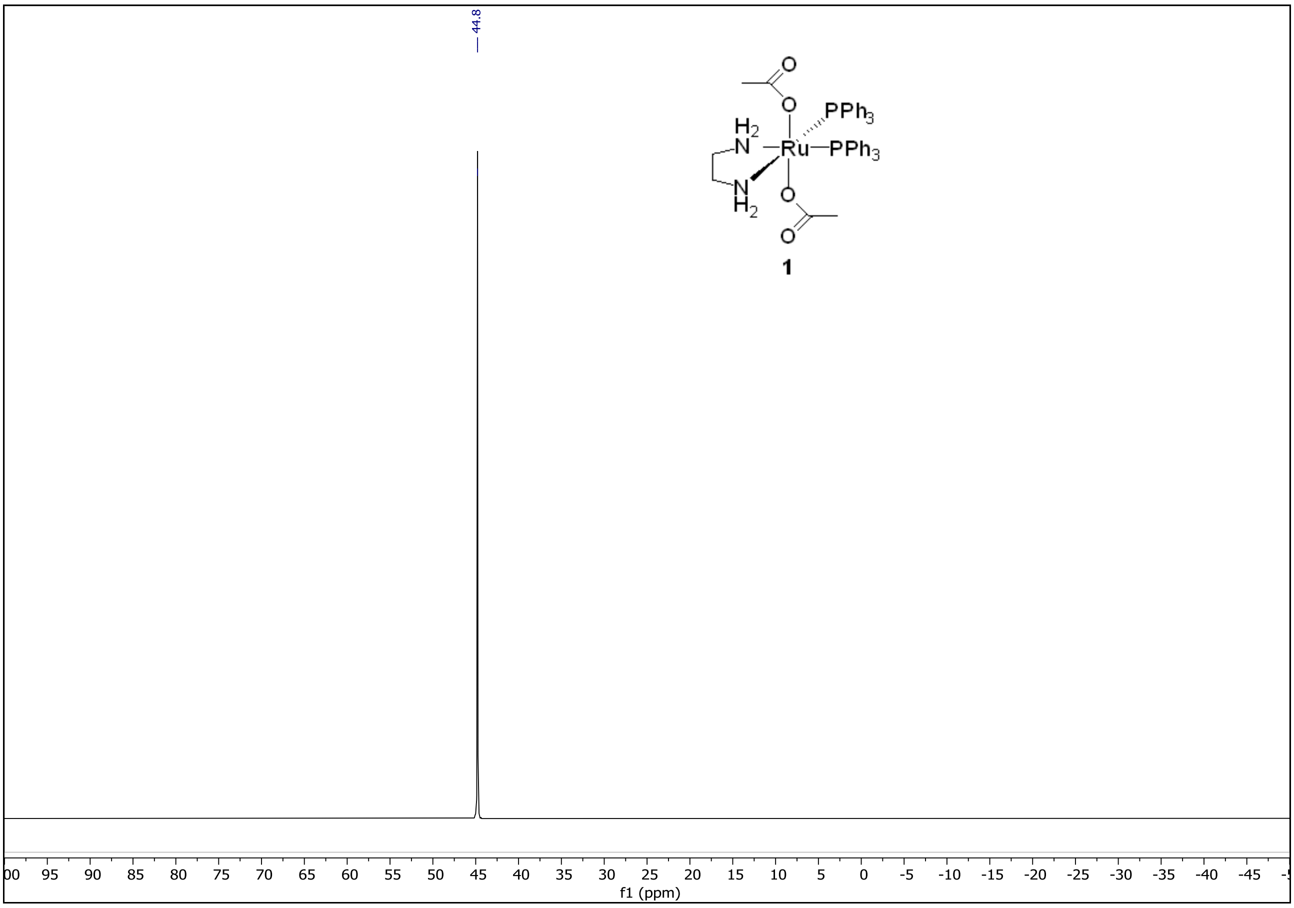

Figure S1. $^{31}P\{^1H\}$ NMR spectrum (162.0 MHz) of *trans,cis*-[Ru(η^1-OAc)$_2$(PPh$_3$)$_2$(en)] (**1**) in CD_2Cl_2 at 25 °C.

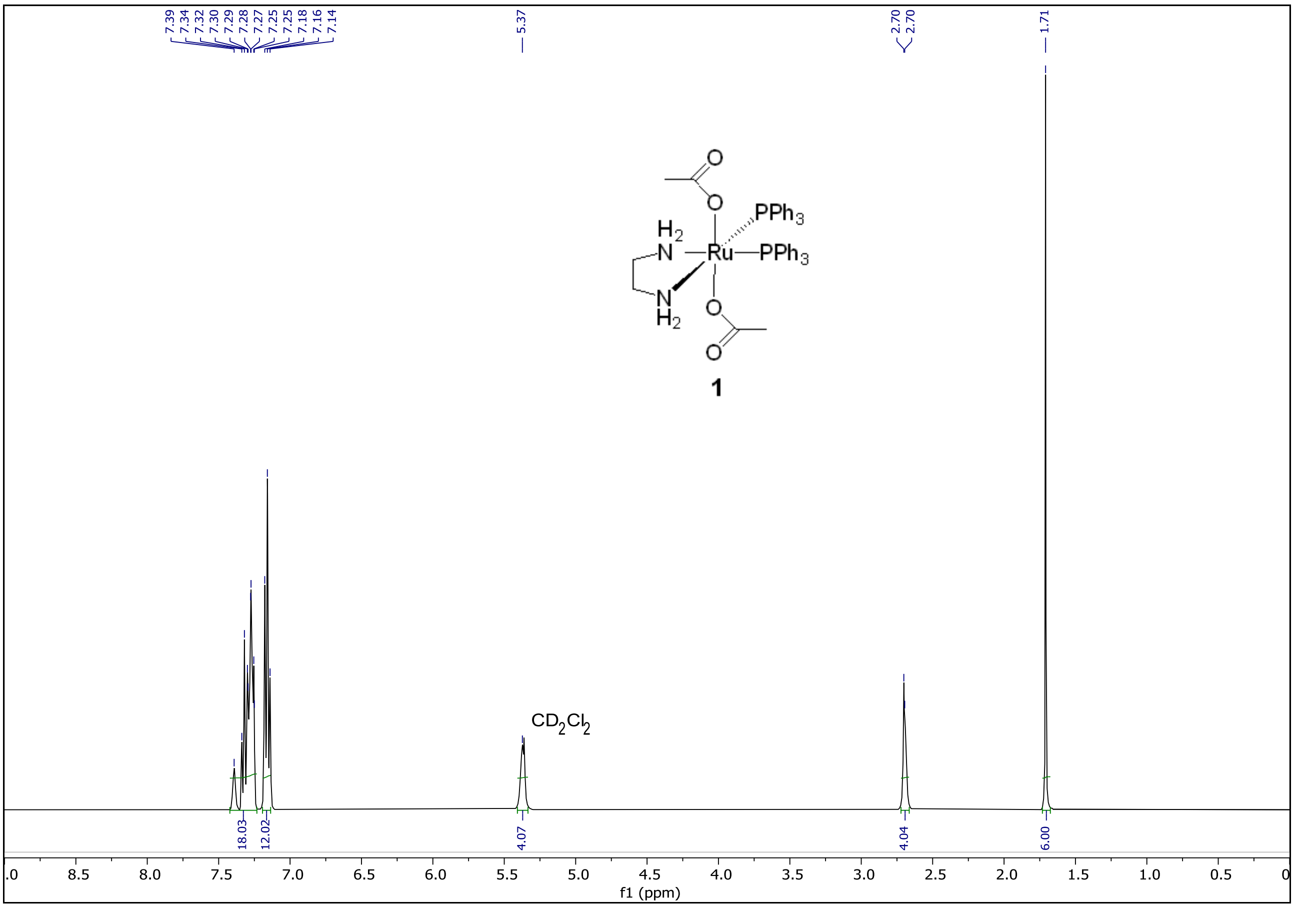

Figure S2. ^{1}H NMR spectrum (400.1 MHz) of *trans*,*cis*-[Ru(η^1-OAc)$_2$(PPh$_3$)$_2$(en)] (**1**) in CD_2Cl_2 at 25 °C.

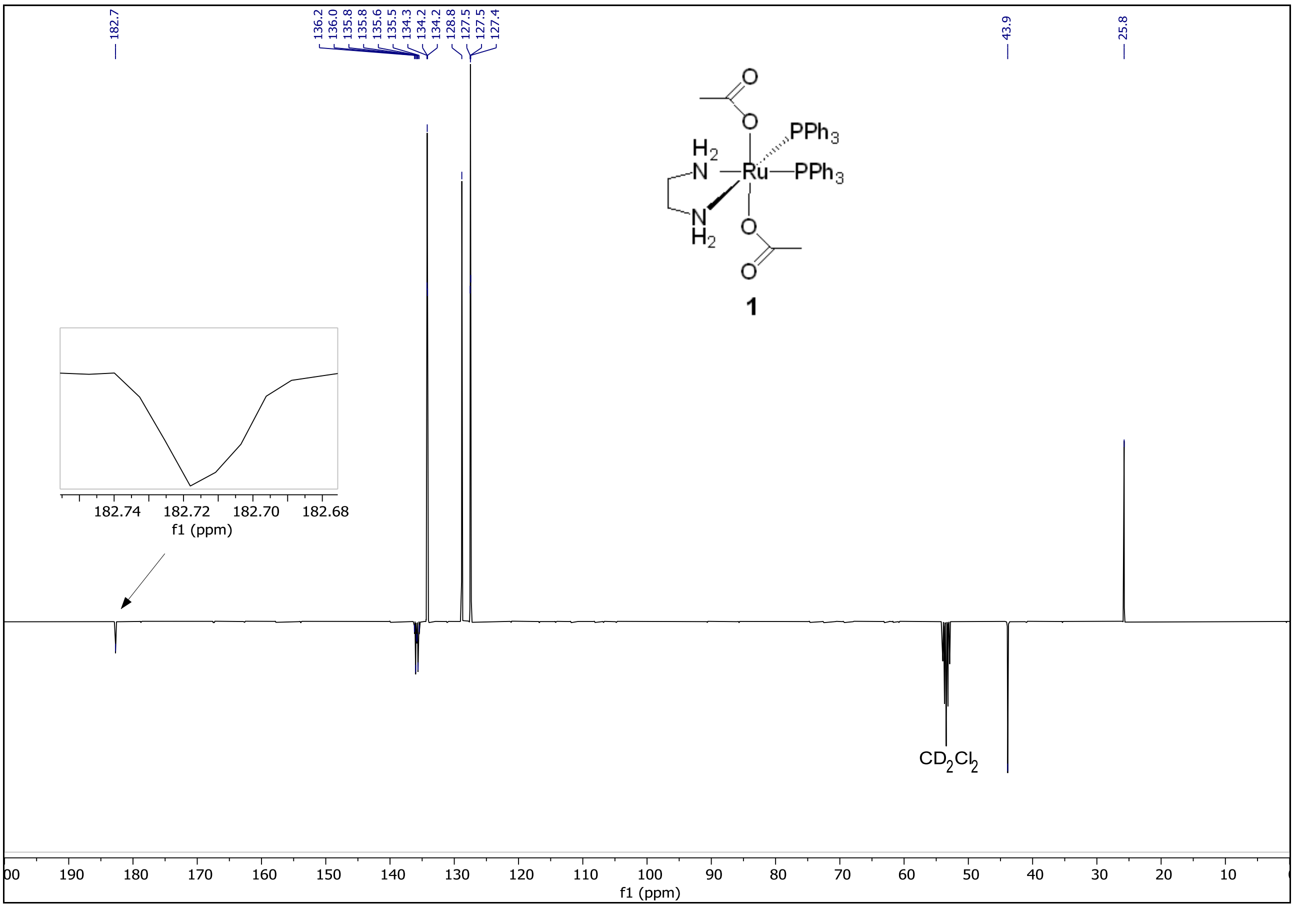

Figure S3. $^{13}C\{^{1}H\}$ DEPTQ NMR spectrum (100.6 MHz) of *trans*,*cis*-[Ru(η^1-OAc)$_2$(PPh$_3$)$_2$(en)] (**1**) in CD_2Cl_2 at 25 °C.

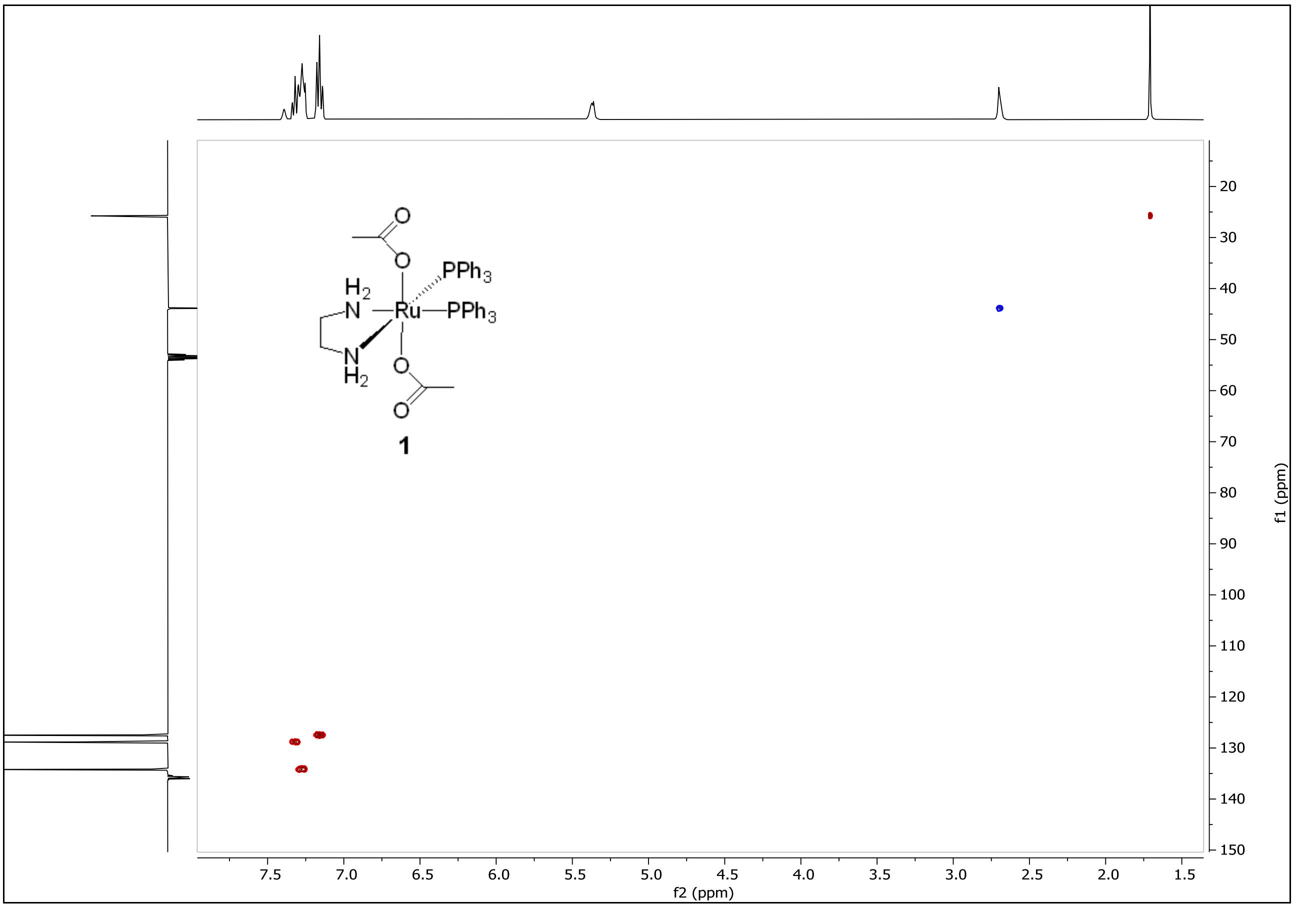

Figure S4. ^{1}H-^{13}C HSQC 2D NMR spectrum of *trans*,*cis*-[Ru(η^1-OAc)$_2$(PPh$_3$)$_2$(en)] (**1**) in CD_2Cl_2 at 25 °C.

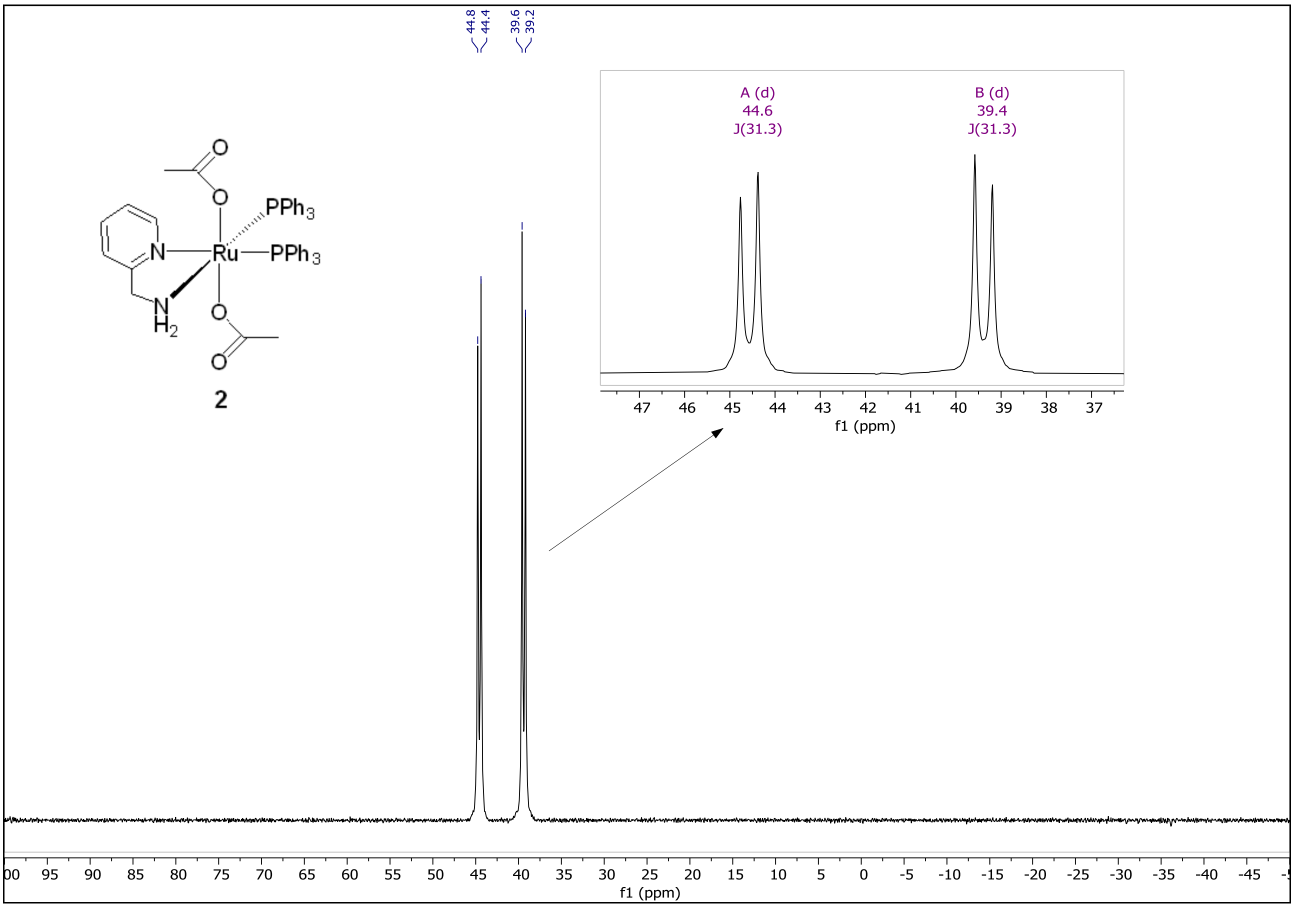

Figure S5. $^{31}P\{^1H\}$ NMR spectrum (81.0 MHz) of *trans*,*cis*-[Ru(η^1-OAc)$_2$(PPh$_3$)$_2$(ampy)] (**2**) in CD_2Cl_2 at 20 °C.

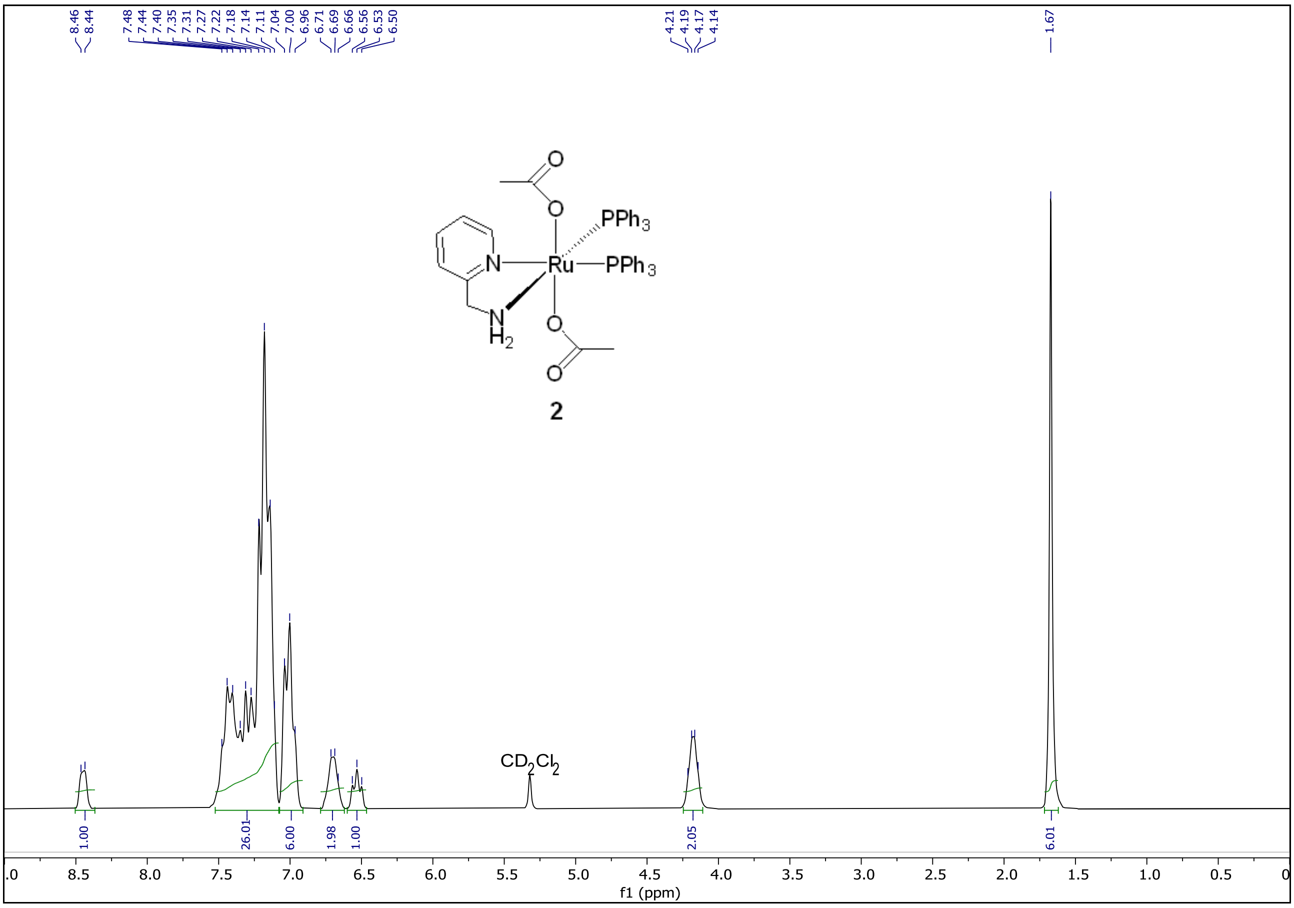

Figure S6. ^{1}H NMR spectrum (200.1 MHz) of *trans*,*cis*-[Ru(η^1-OAc)$_2$(PPh$_3$)$_2$(ampy)] (**2**) in CD_2Cl_2 at 20 °C.

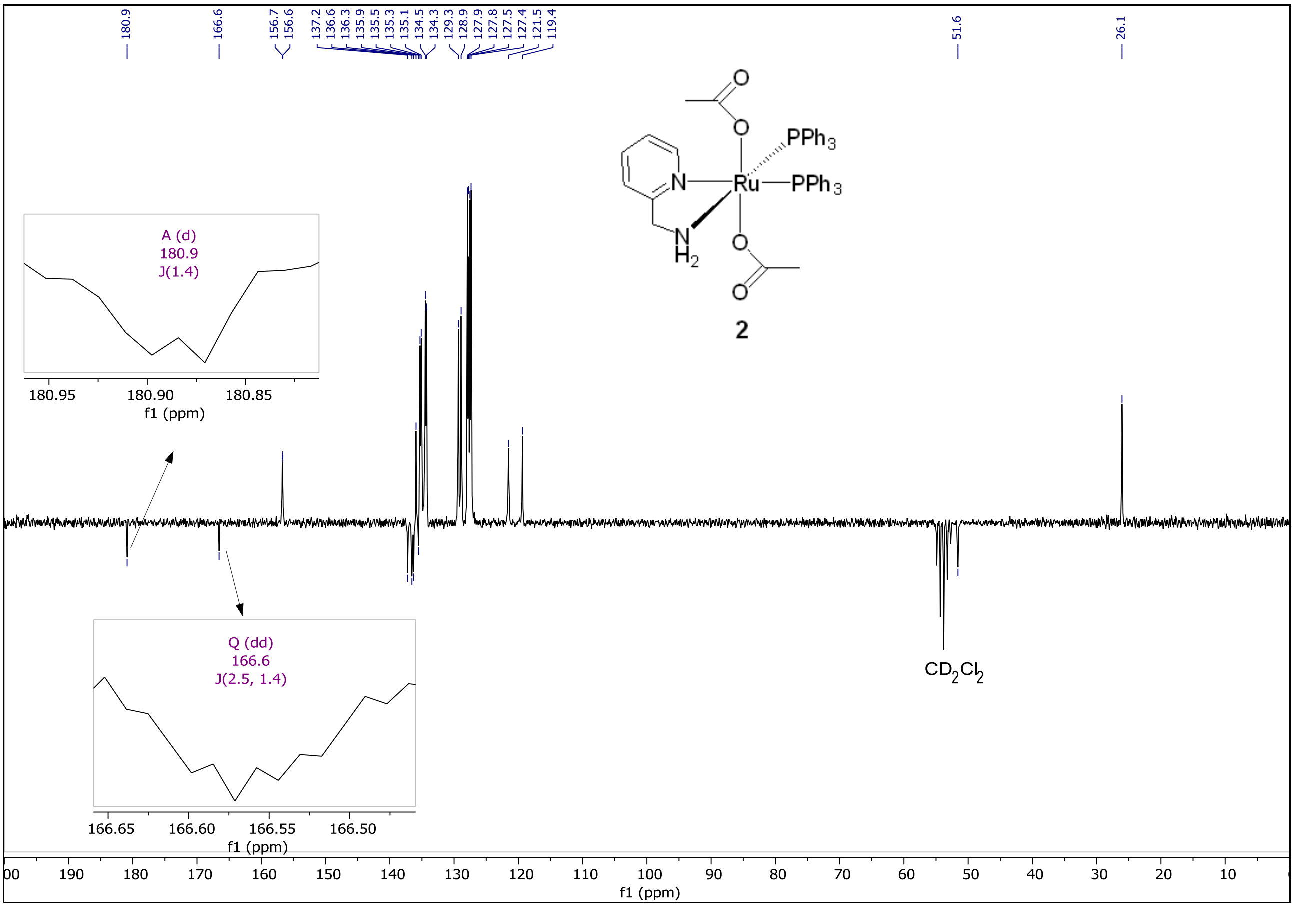

Figure S7. $^{13}C\{^1H\}$ PENDANT NMR spectrum (50.3 MHz) of *trans*,*cis*-[Ru(η^1-OAc)$_2$(PPh$_3$)$_2$(ampy)] (**2**) in CD_2Cl_2 at 20 °C.

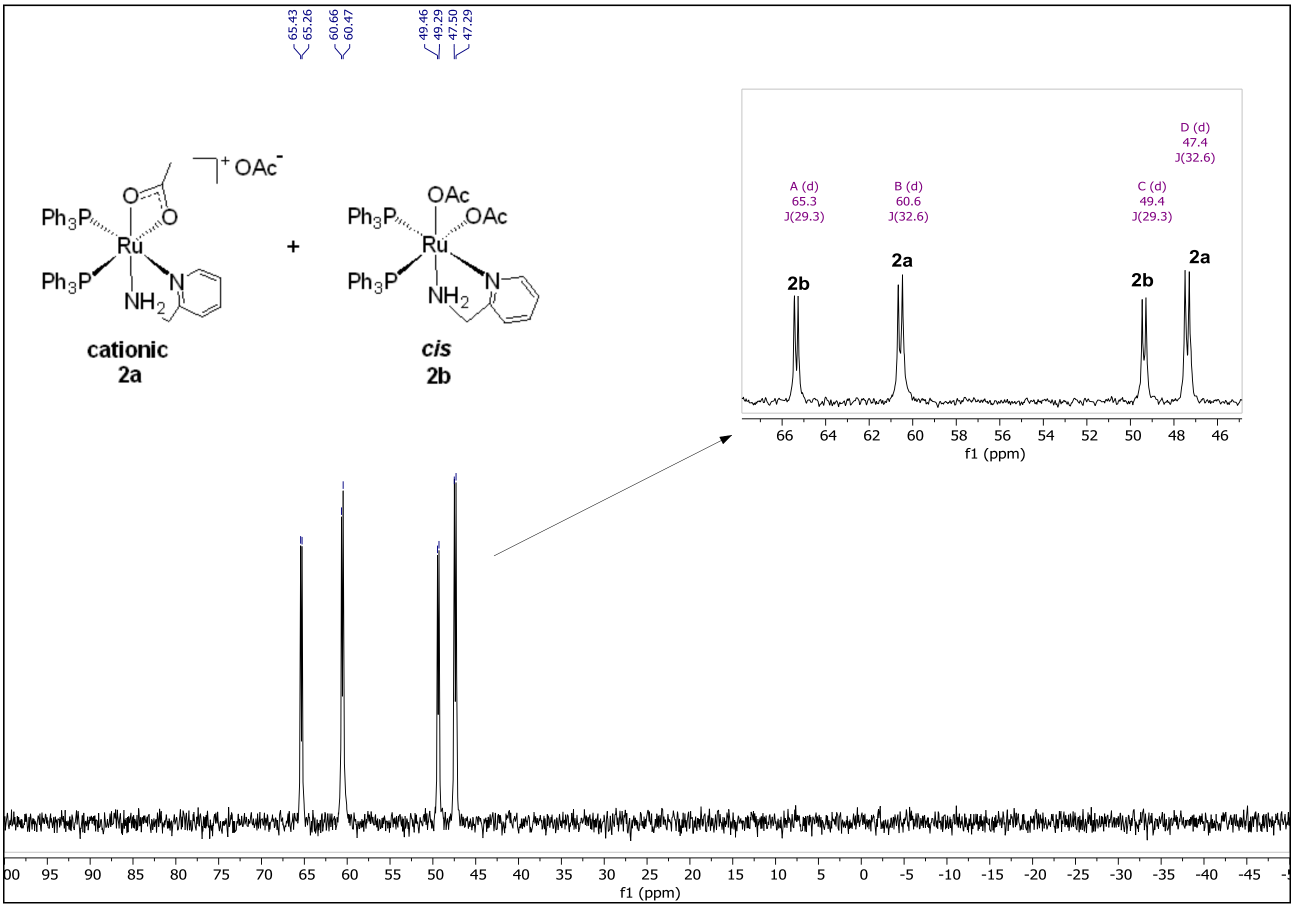

Figure S8. ${}^{31}P\{{}^{1}H\}$ NMR spectrum (162.0 MHz) of the mixture 3:2 of the cationic species *cis*-[Ru(η^2-OAc)(PPh_3)$_2$(ampy)]OAc (**2a**) and *cis*,*cis*-[Ru(η^1-OAc)$_2$(PPh_3)$_2$(ampy)] (**2b**) in CD_3OD at 25 °C.

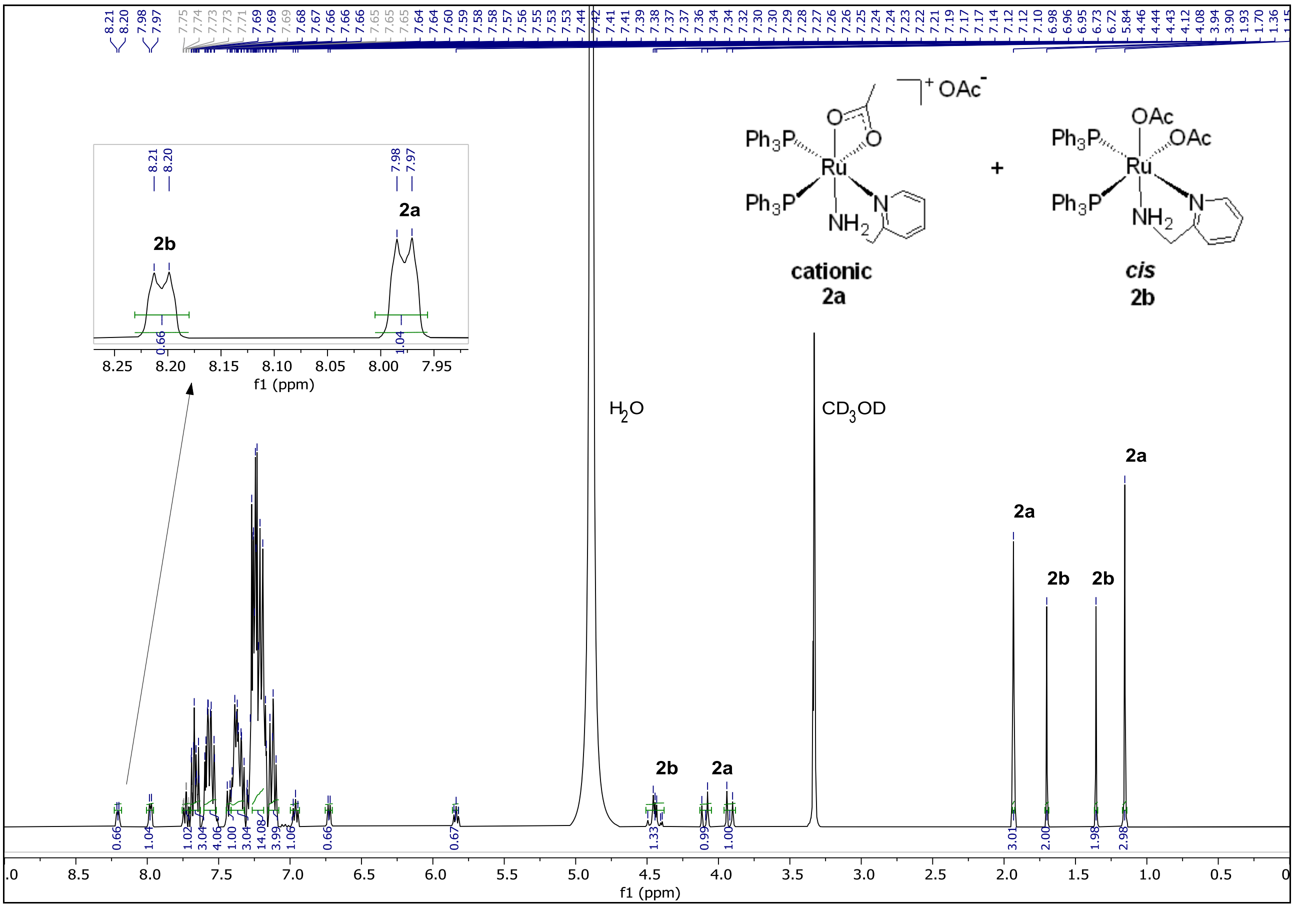

Figure S9. ^{1}H NMR spectrum (400.1 MHz) of the mixture 3:2 of the cationic species *cis*-[Ru(η^2-OAc)(PPh_3)$_2$(ampy)]OAc (**2a**) and *cis*,*cis*-[Ru(η^1-OAc)$_2$(PPh_3)$_2$(ampy)] (**2b**) in CD_3OD at 25 °C.

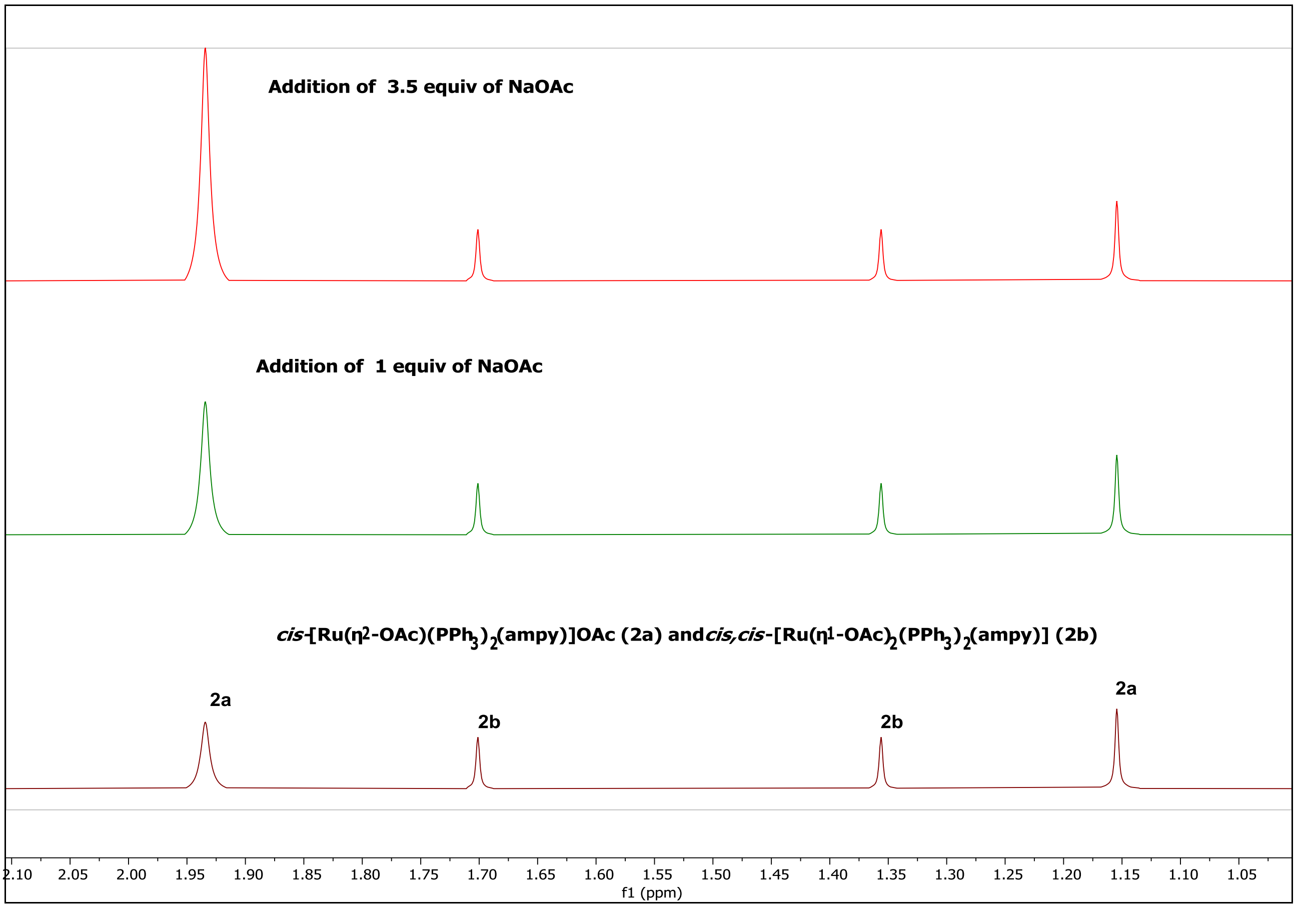

Figure S10. Effect of the addition of NaOAc (3.5 equiv) to the mixture 3:2 of the cationic species *cis*-[Ru(η^2-OAc)(PPh_3)$_2$(ampy)]OAc (**2a**) and *cis*,*cis*-[Ru(η^1-OAc)$_2$(PPh_3)$_2$(ampy)] (**2b**) in the methyl acetate region of the ^{1}H NMR spectrum (400.1 MHz) in CD_3OD at 25 °C.

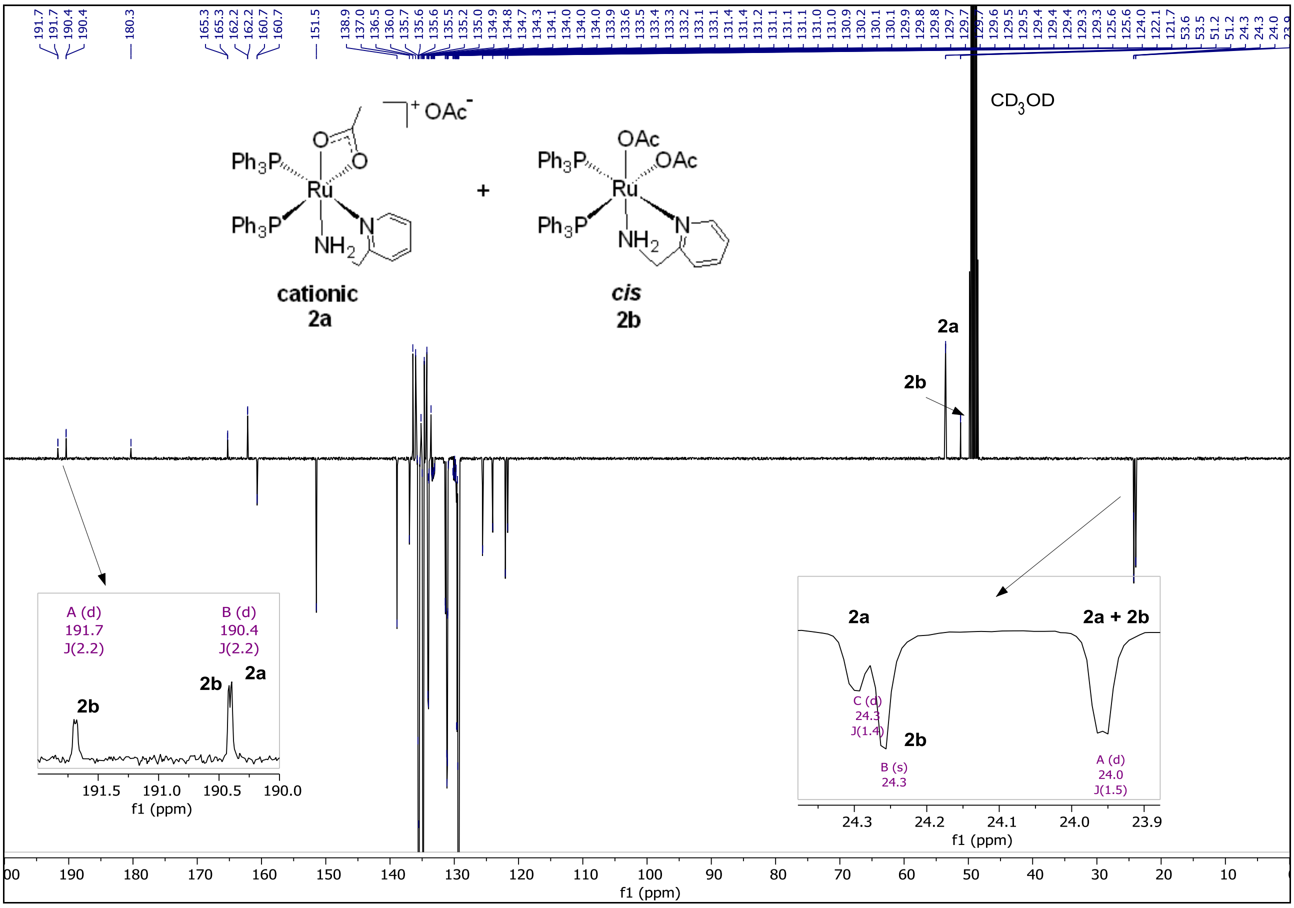

Figure S11. $^{13}C\{^{1}H\}$ DEPTQ NMR spectrum (100.6 MHz) of the mixture 3:2 of the cationic species *cis*-[Ru(η^2-OAc)(PPh_3)$_2$(ampy)]OAc (**2a**) and *cis*,*cis*-[Ru(η^1-OAc)$_2$(PPh_3)$_2$(ampy)] (**2b**) in CD_3OD at 25 °C.

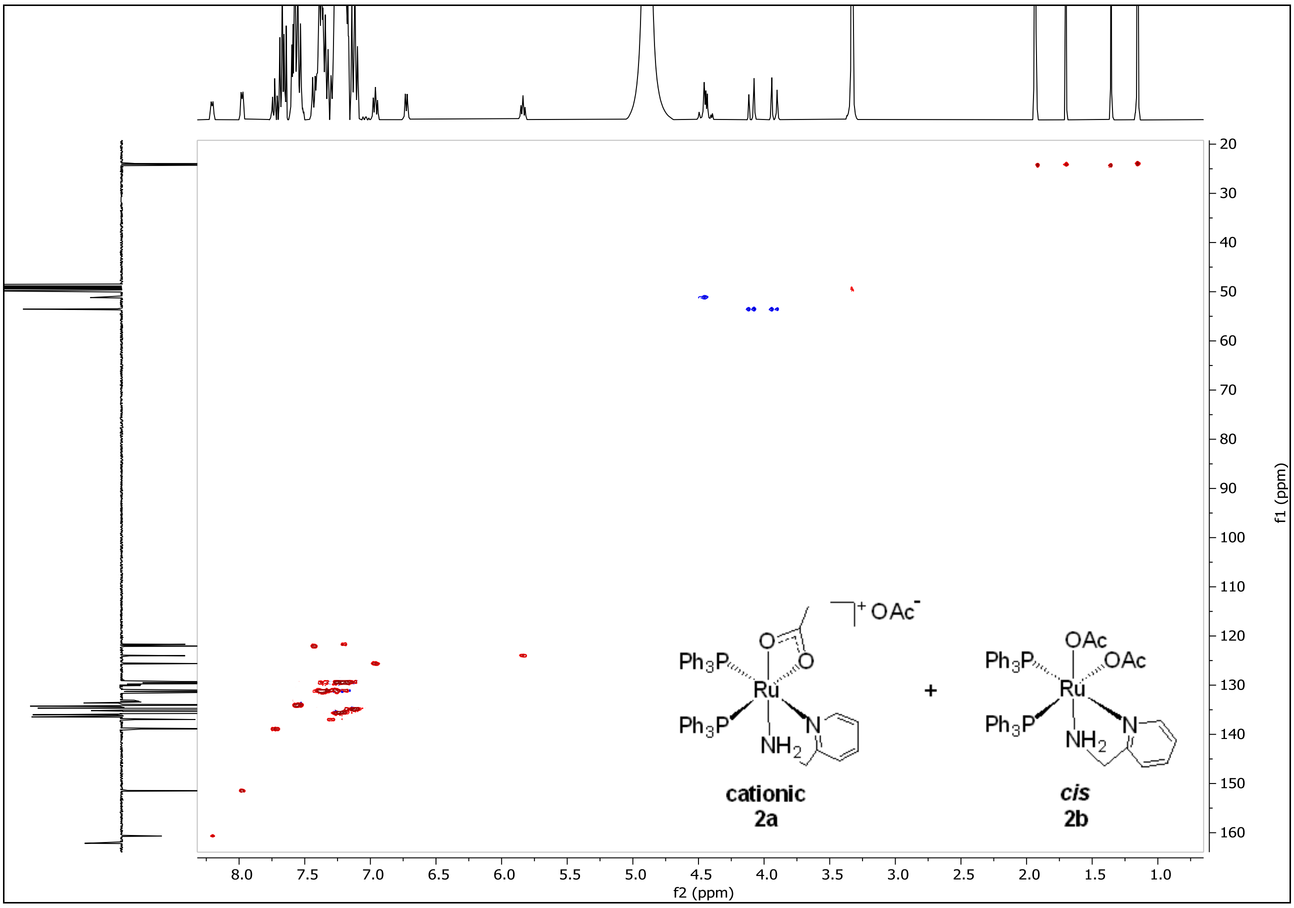

Figure S12. ^{1}H-^{13}C HSQC 2D NMR spectrum of of the mixture 3:2 of the cationic species *cis*-[Ru(η^2-OAc)(PPh_3)$_2$(ampy)]OAc (**2a**) and *cis*,*cis*-[Ru(η^1-OAc)$_2$(PPh_3)$_2$(ampy)] (**2b**) in CD_3OD at 25 °C.

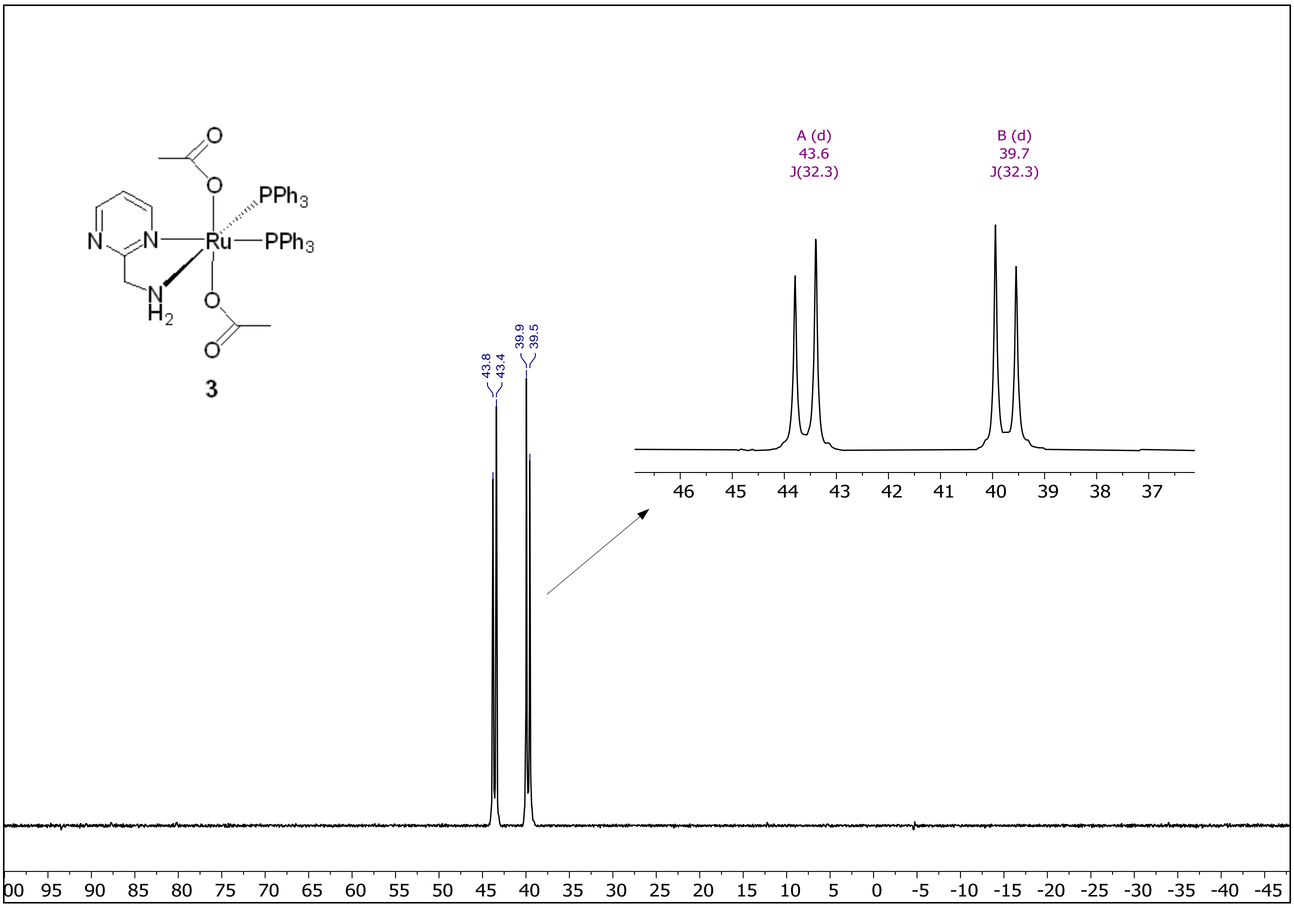

Figure S13. $^{31}P\{^1H\}$ NMR spectrum (81.0 MHz) of *trans*,*cis*-[Ru(η^1-OAc)$_2$(PPh$_3$)$_2$(ampyrim)] (**3**) in $CDCl_3$ at 20 °C.

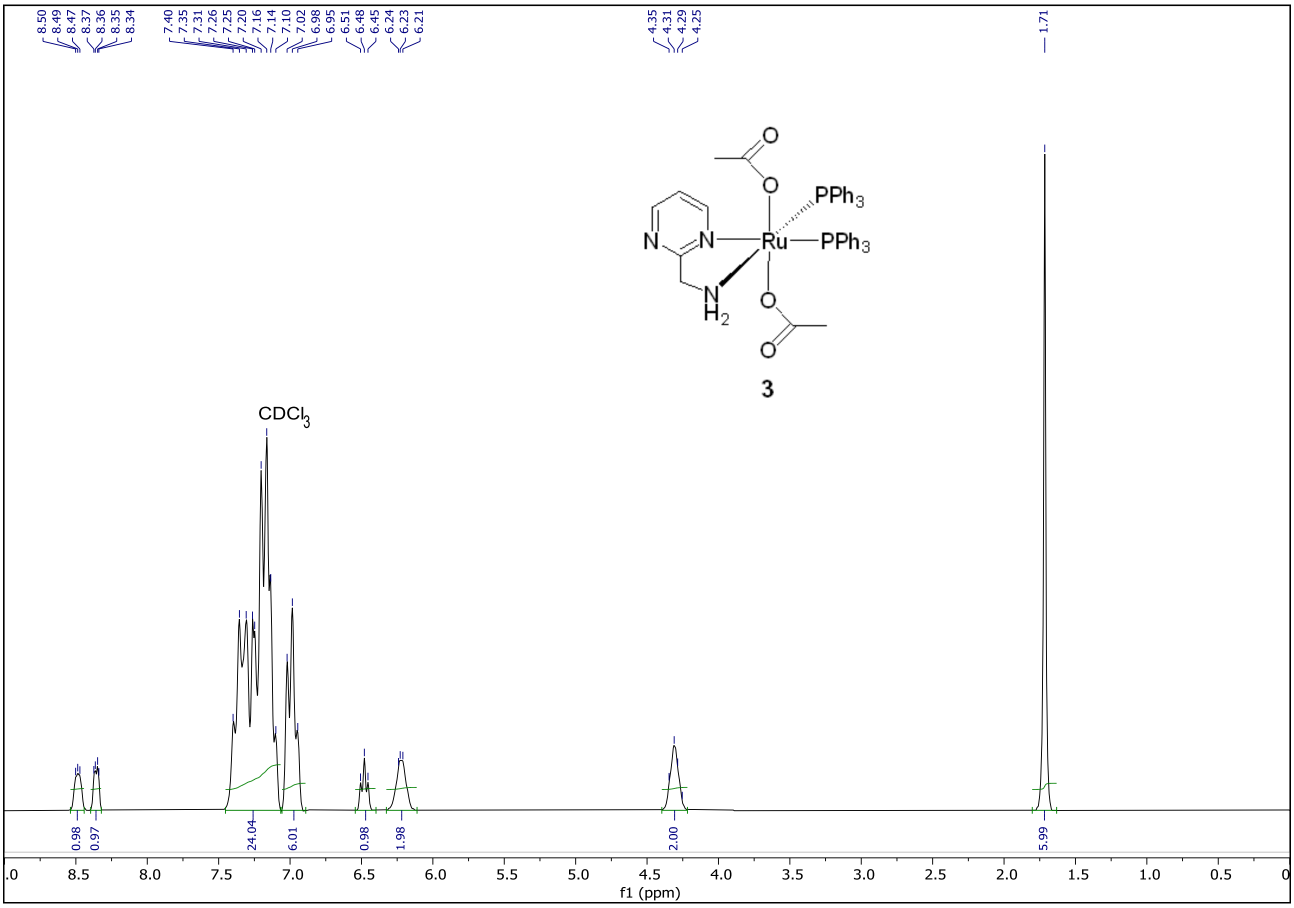

Figure S14. ^{1}H NMR spectrum (200.1 MHz) of *trans*,*cis*-[Ru(η^1-OAc)$_2$(PPh_3)$_2$(ampyrim)] (**3**) in $CDCl_3$ at 20 °C.

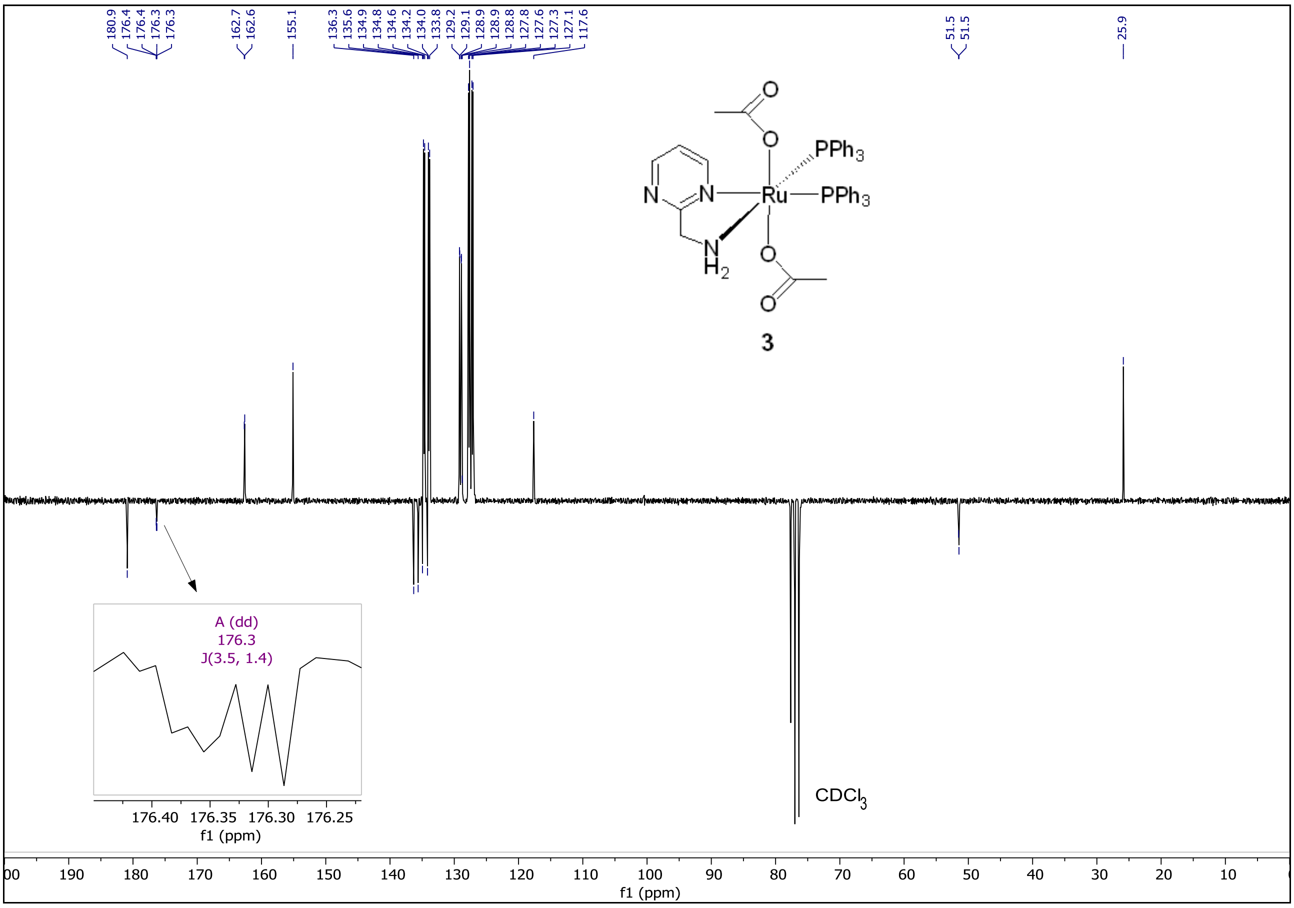

Figure S15. $^{13}C\{^{1}H\}$ PENDANT NMR spectrum (50.3 MHz) of *trans*,*cis*-[Ru(η^1-OAc)$_2$(PPh$_3$)$_2$(ampyrim)] (**3**) in $CDCl_3$ at 20 °C.

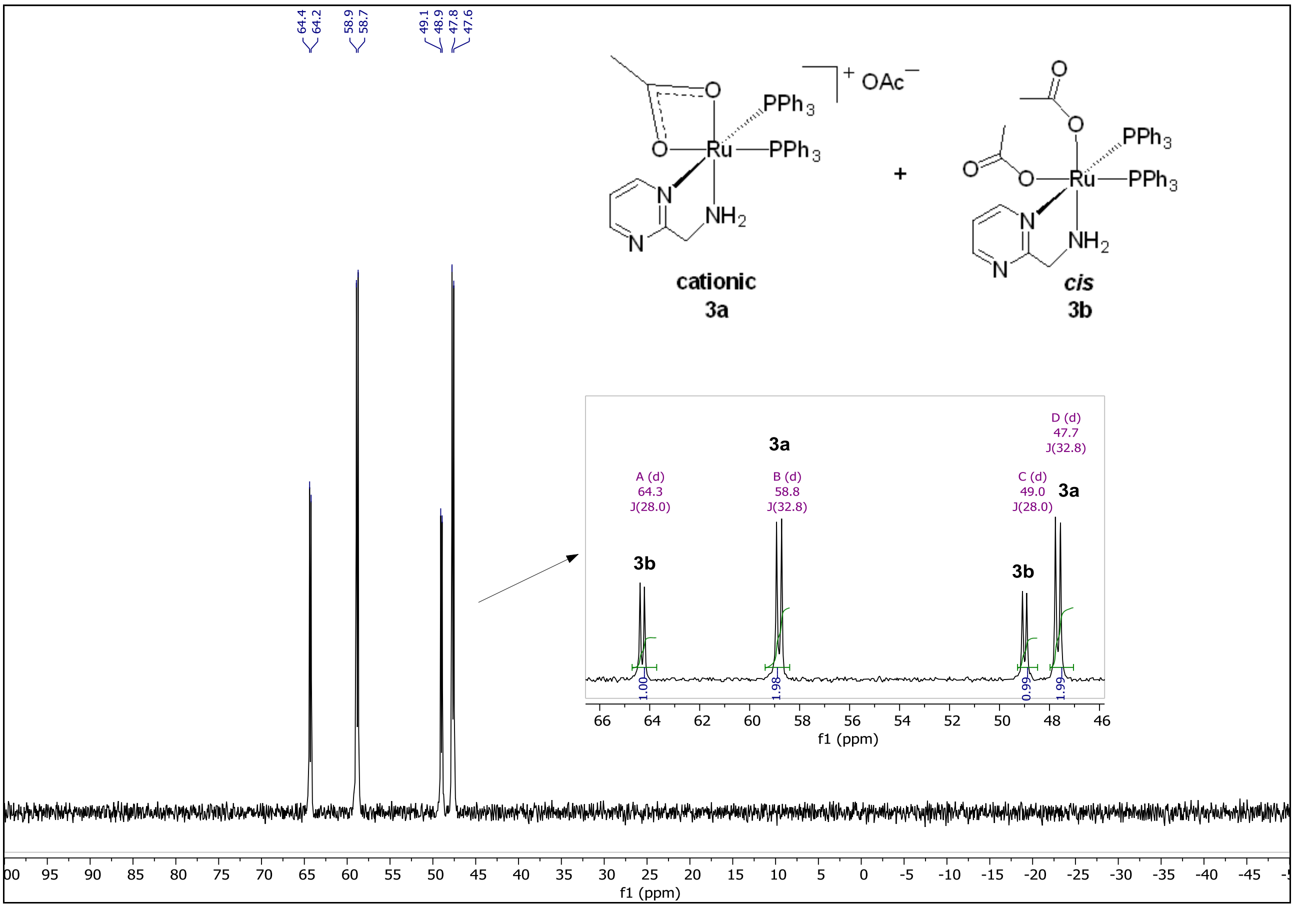

Figure S16. $^{31}P\{^{1}H\}$ NMR spectrum (162.0 MHz) of the mixture 2:1 of the cationic species *cis*-[Ru(η^2-OAc)(PPh_3)$_2$(ampyrim)]OAc (**3a**) and *cis*,*cis*-[Ru(η^1-OAc)$_2$(PPh_3)$_2$(ampyrim)] (**3b**) in CD_3OD at 25 °C.

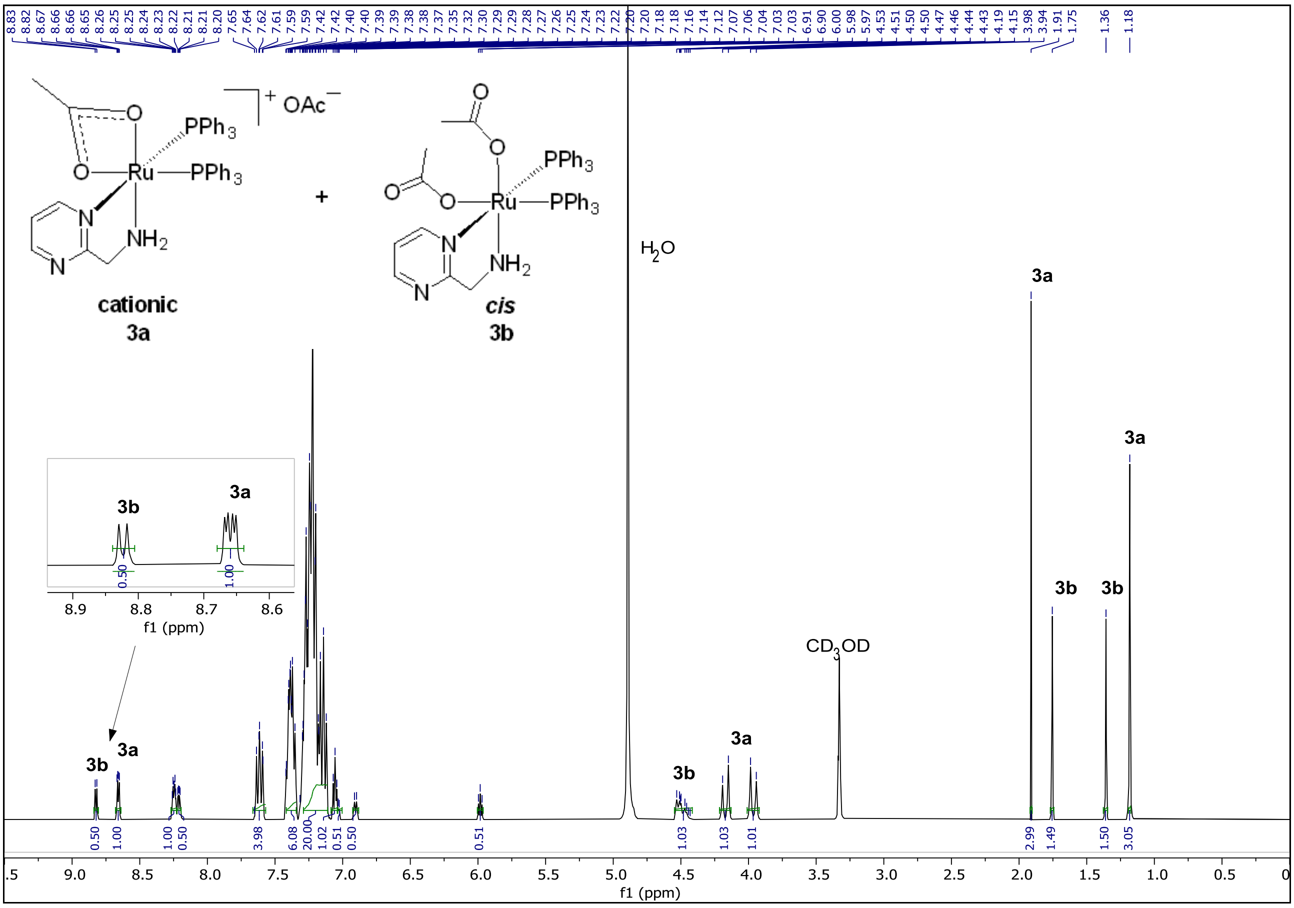

Figure S17. ^{1}H NMR spectrum (400.1 MHz) of the mixture 2:1 of the cationic species *cis*-[Ru(η^2-OAc)(PPh_3)$_2$(ampyrim)]OAc (**3a**) and *cis*,*cis*-[Ru(η^1-OAc)$_2$(PPh_3)$_2$(ampyrim)] (**3b**) in CD_3OD at 25 °C.

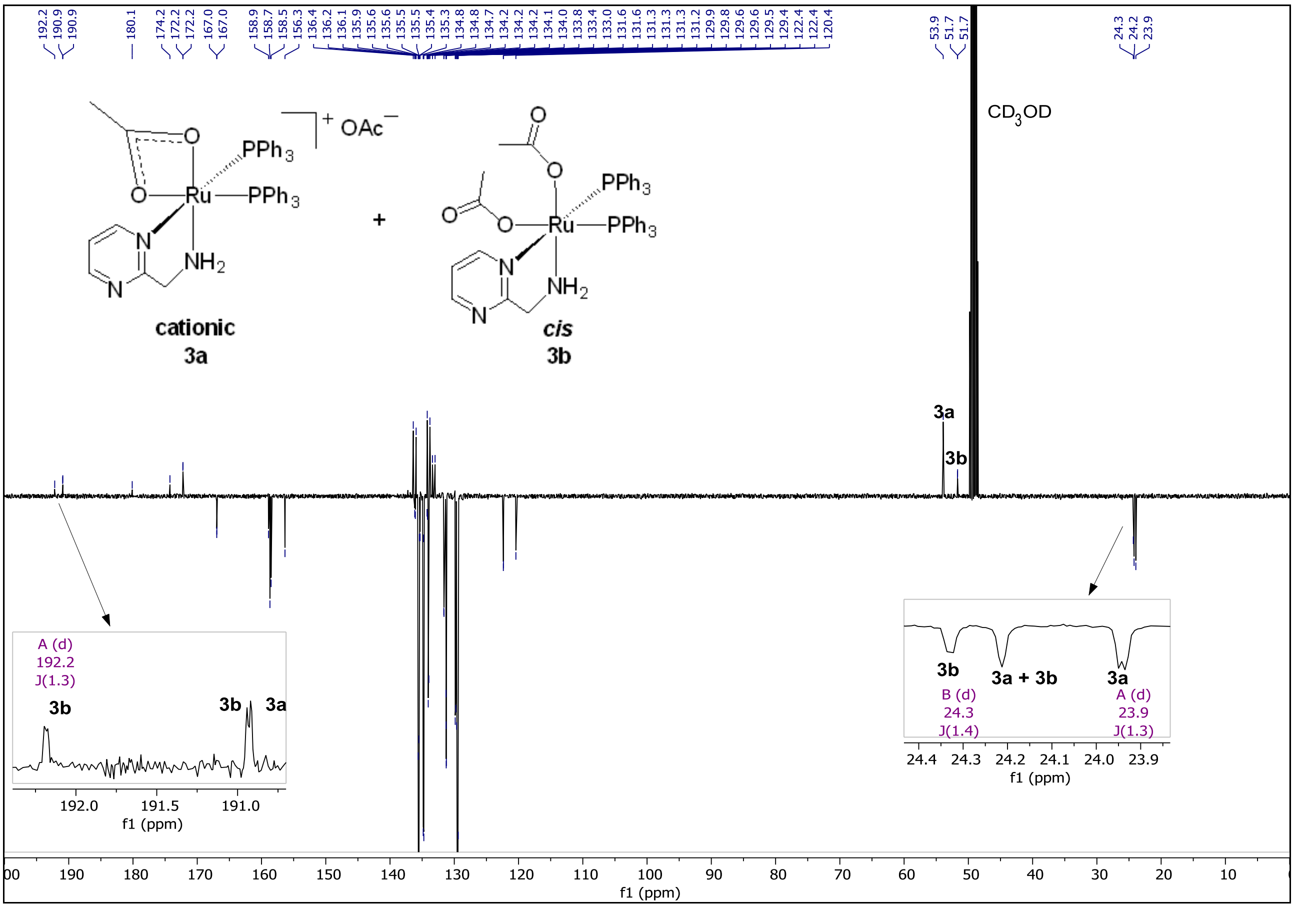

Figure S18. $^{13}C\{^{1}H\}$ DEPTQ NMR spectrum (100.6 MHz) of the mixture 2:1 of the cationic species *cis*-[Ru(η^2-OAc)(PPh_3)$_2$(ampyrim)]OAc (**3a**) and *cis*,*cis*-[Ru(η^1-OAc)$_2$(PPh_3)$_2$(ampyrim)] (**3b**) in CD_3OD at 25 °C.

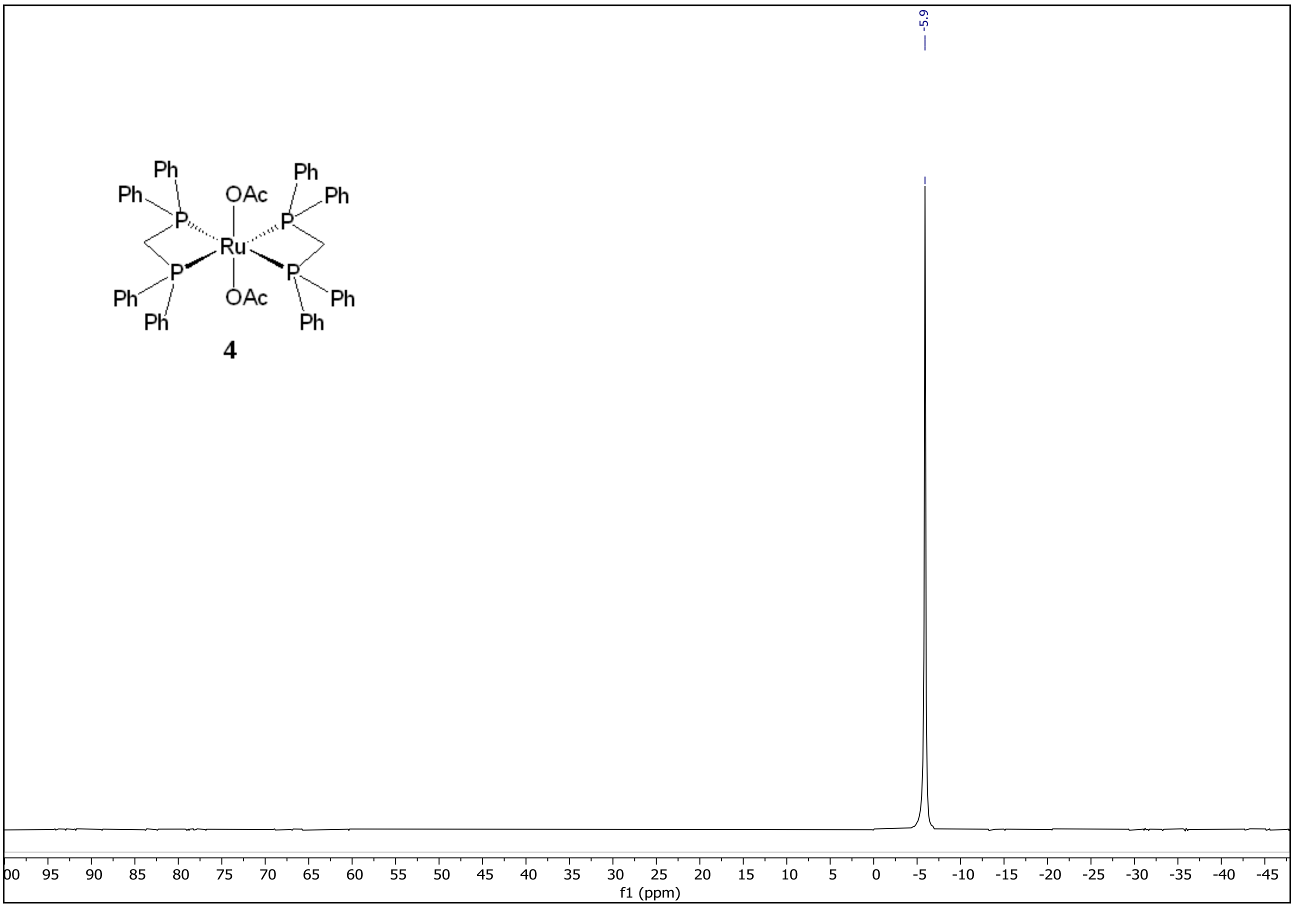

Figure S19. $^{31}P\{^{1}H\}$ NMR spectrum (81.0 MHz) of *trans*-[Ru(η^1-OAc)$_2$(dppm)$_2$] (**4**) in $CDCl_3$ at 20 °C.

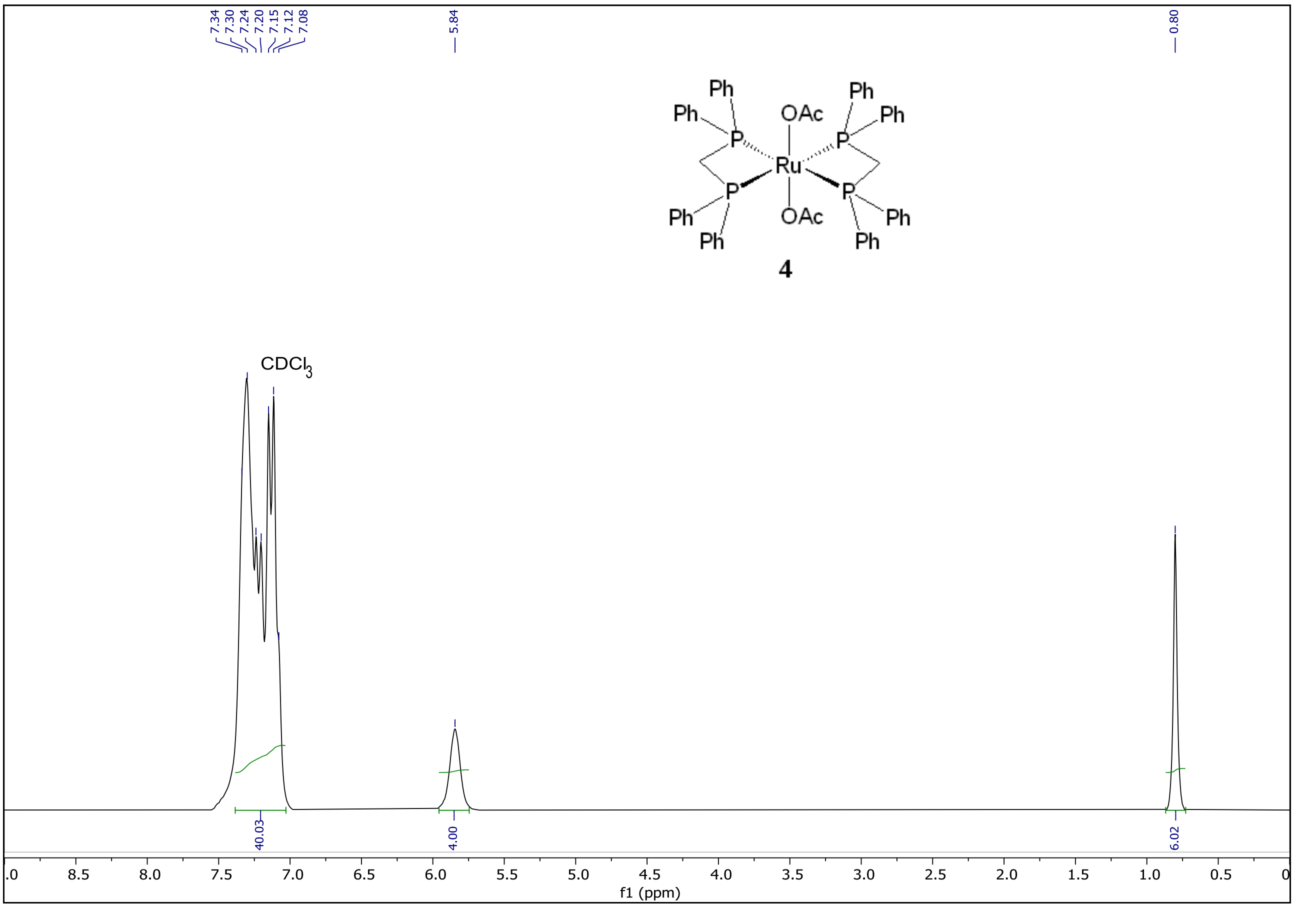

Figure S20. ^{1}H NMR spectrum (200.1 MHz) of *trans*-[Ru(η^1-OAc)$_2$(dppm)$_2$] (**4**)) in $CDCl_3$ at 20 °C.

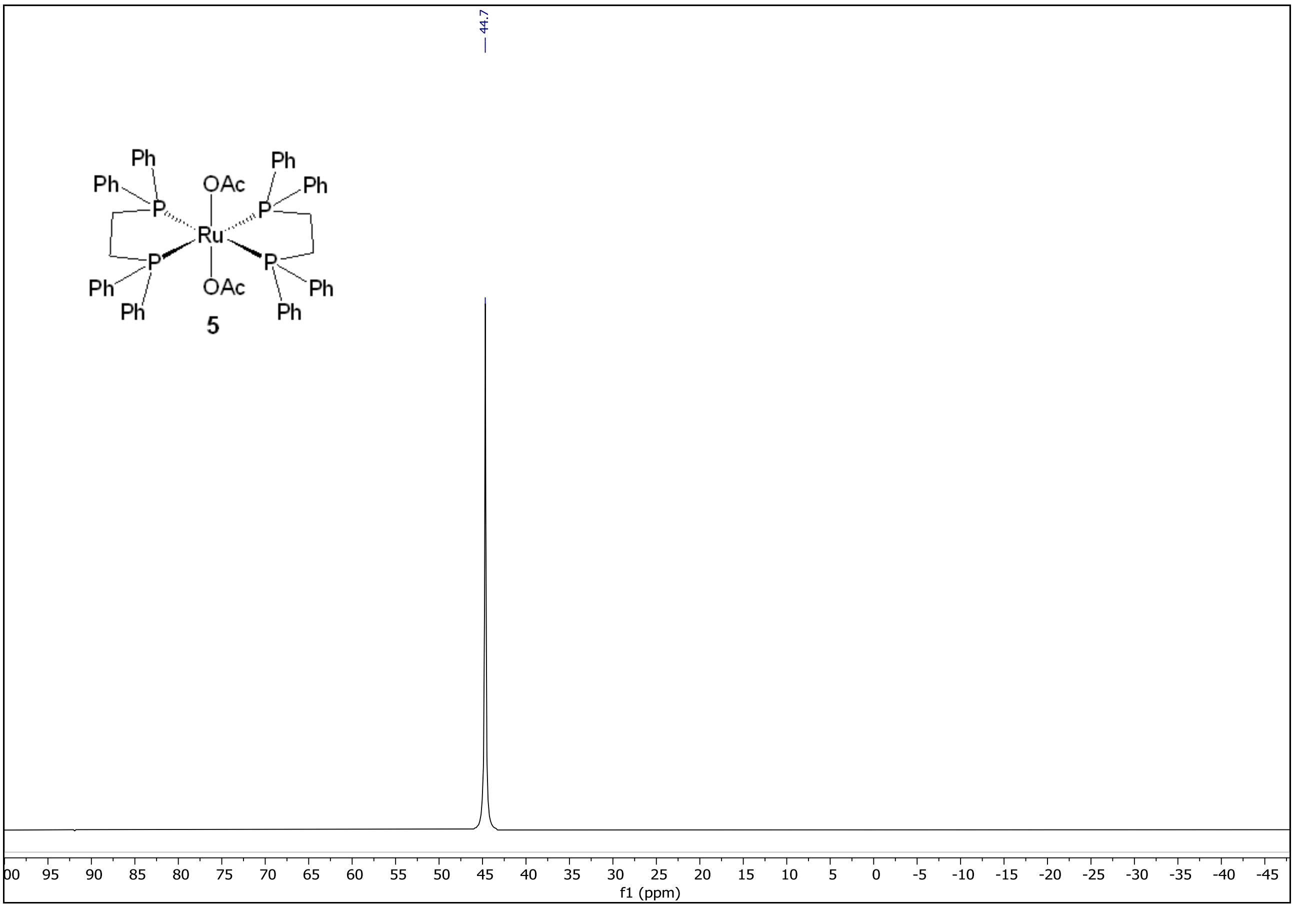

Figure S21. $^{31}P\{^{1}H\}$ NMR spectrum (81.0 MHz) of *trans*-[Ru(η^1-OAc)$_2$(dppe)$_2$] (**5**) in $CDCl_3$ at 20 °C.

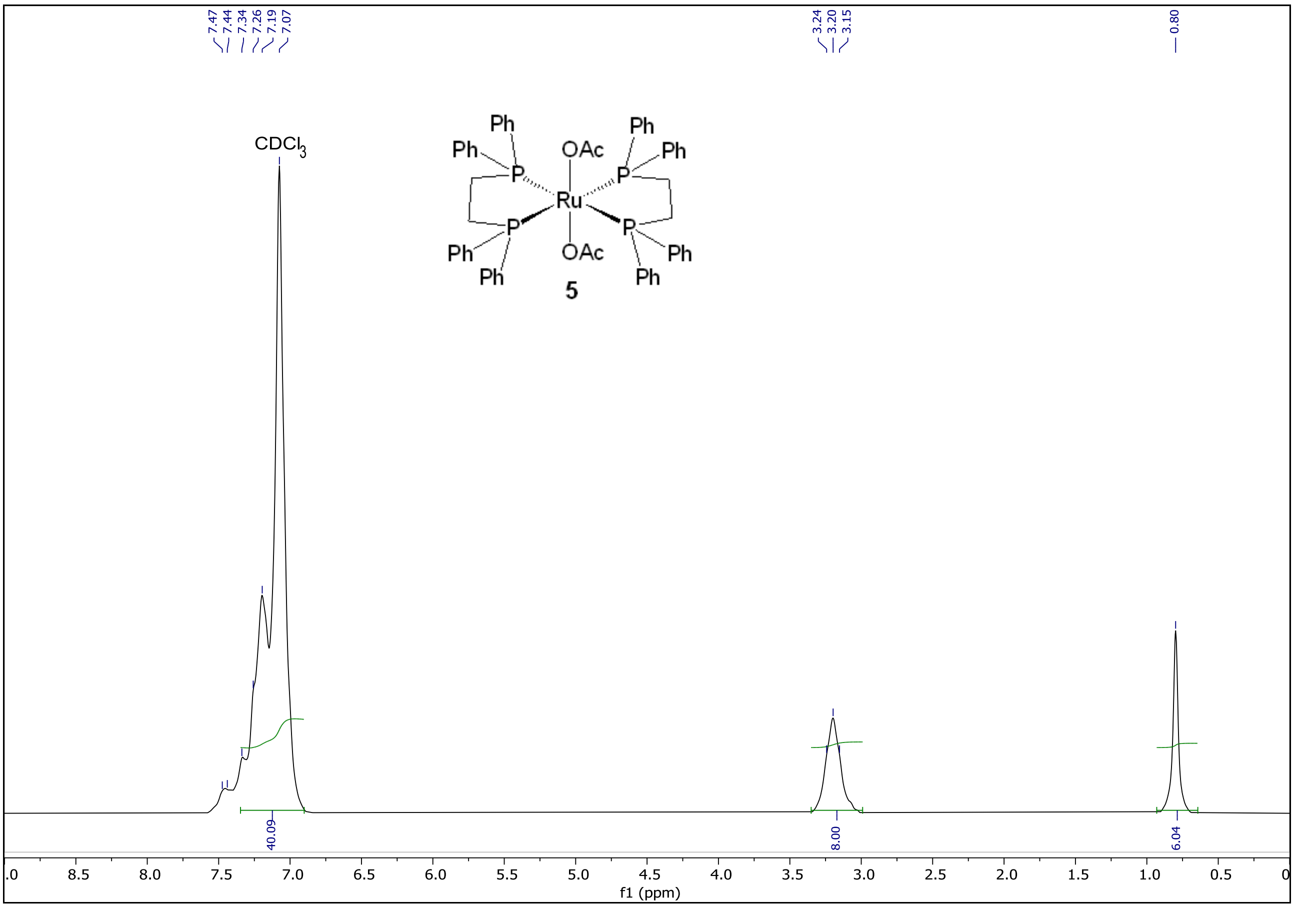

Figure S22. ^{1}H NMR spectrum (200.1 MHz) of *trans*-[Ru(η^1-OAc)$_2$(dppe)$_2$] (**5**) in $CDCl_3$ at 20 °C.

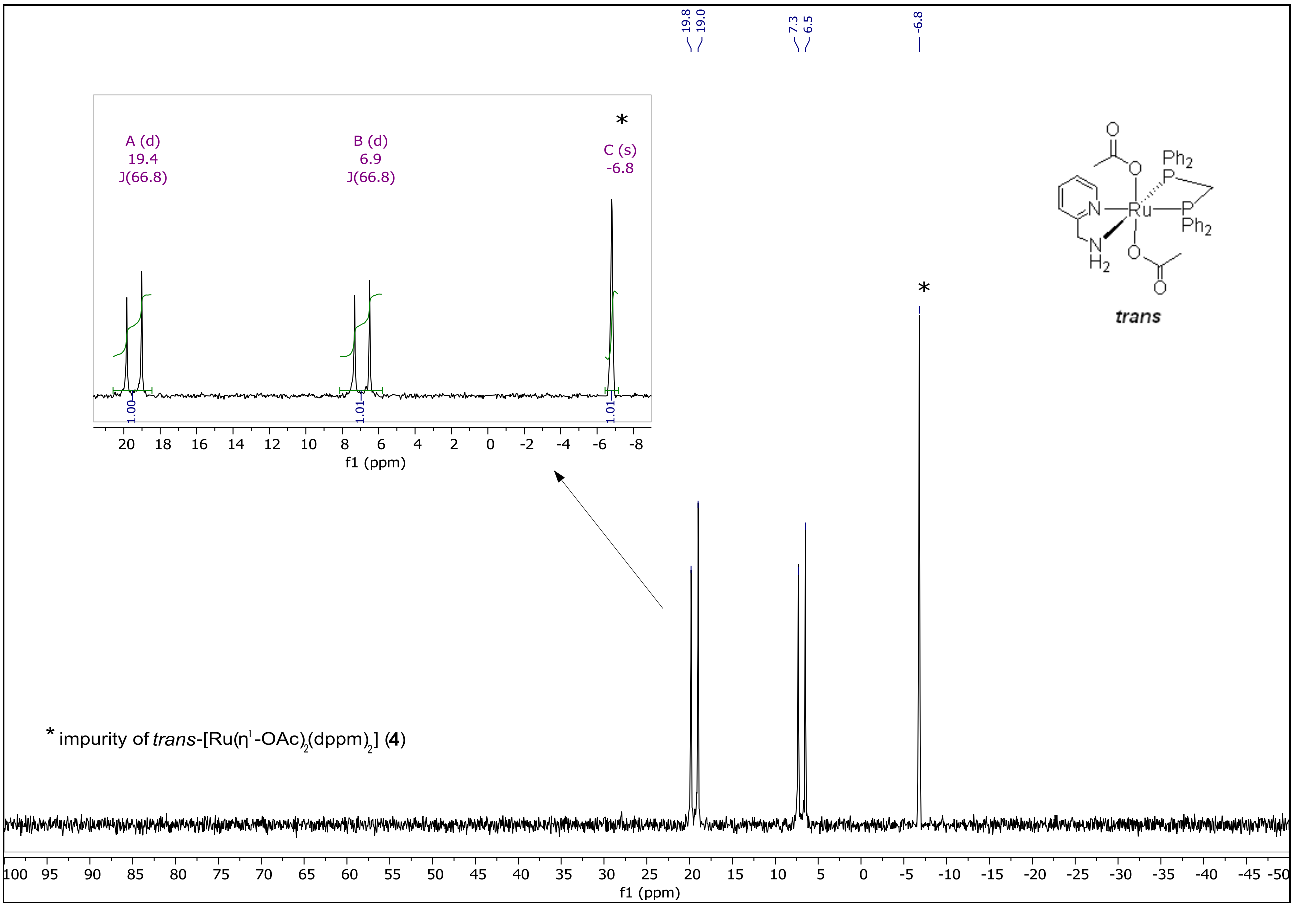

Figure S23. $^{31}P\{^1H\}$ NMR spectrum (81.0 MHz) of the 2:1 mixture of *trans*-[Ru(η^1-OAc)$_2$(dppm)(ampy)] and *trans*-[Ru(η^1-OAc)$_2$(dppm)$_2$] (**4**) in CD_2Cl_2 at 20 °C.

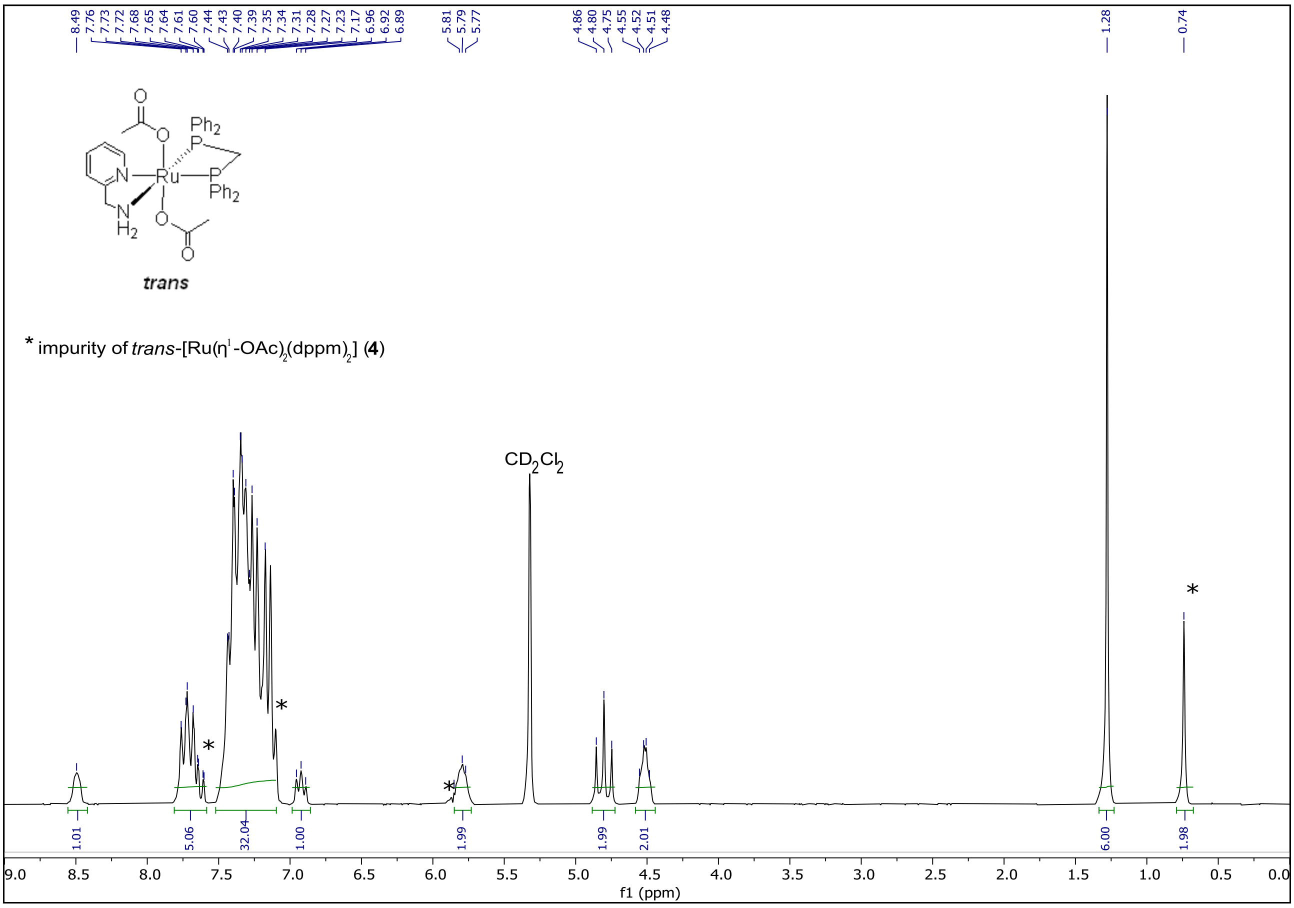

Figure S24. ^{1}H NMR spectrum (200.1 MHz) of the 2:1 mixture of *trans*-[Ru(η^1-OAc)$_2$(dppm)(ampy)] and *trans*-[Ru(η^1-OAc)$_2$(dppm)$_2$] (**4**) in CD_2Cl_2 at 20 °C.

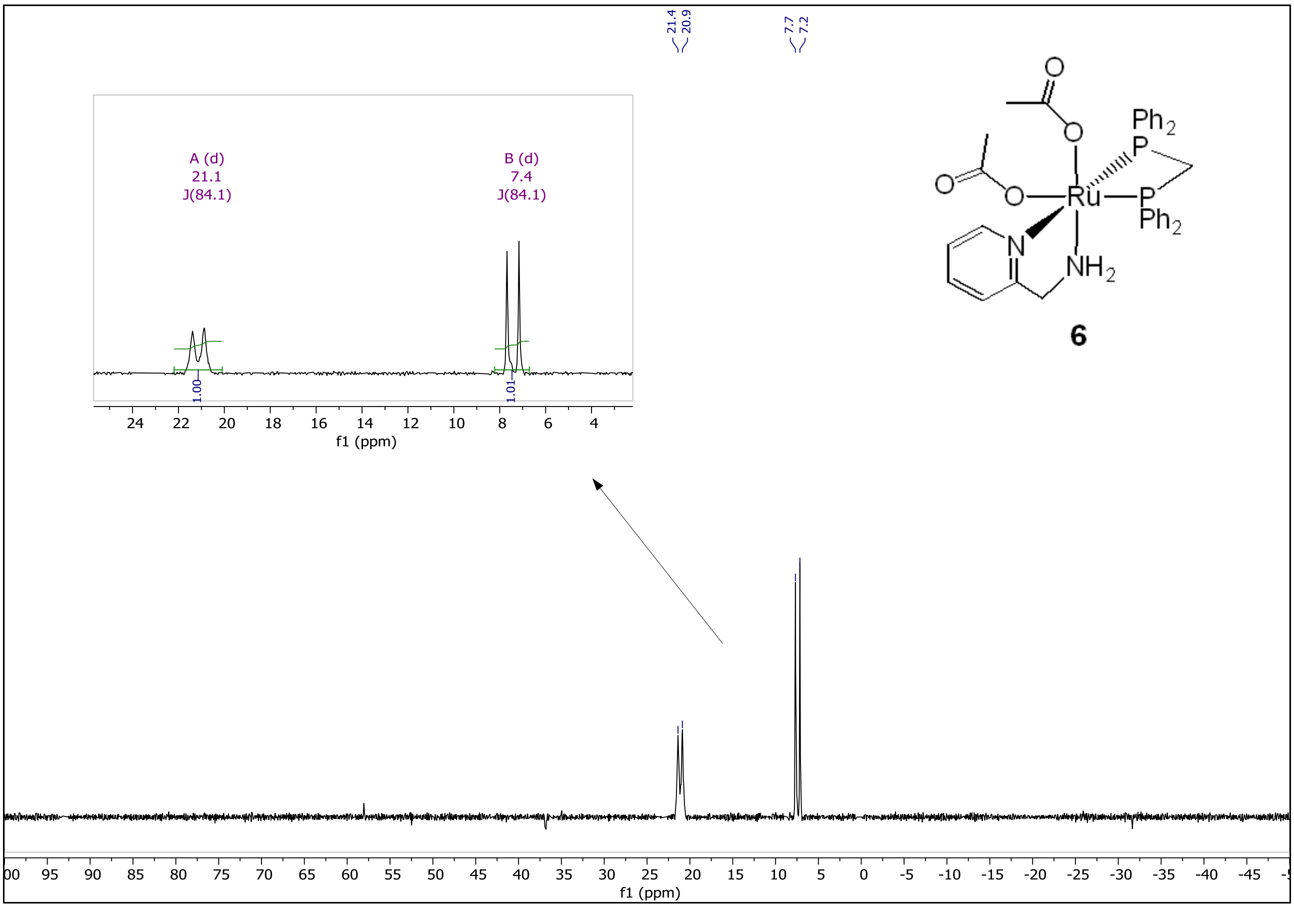

Figure S25. $^{31}P\{^{1}H\}$ NMR spectrum (162.0 MHz) of *cis*-[Ru(η^{1}-OAc)$_2$(dppm)(ampy)] (**6**) in CD_3OD at 25 °C.

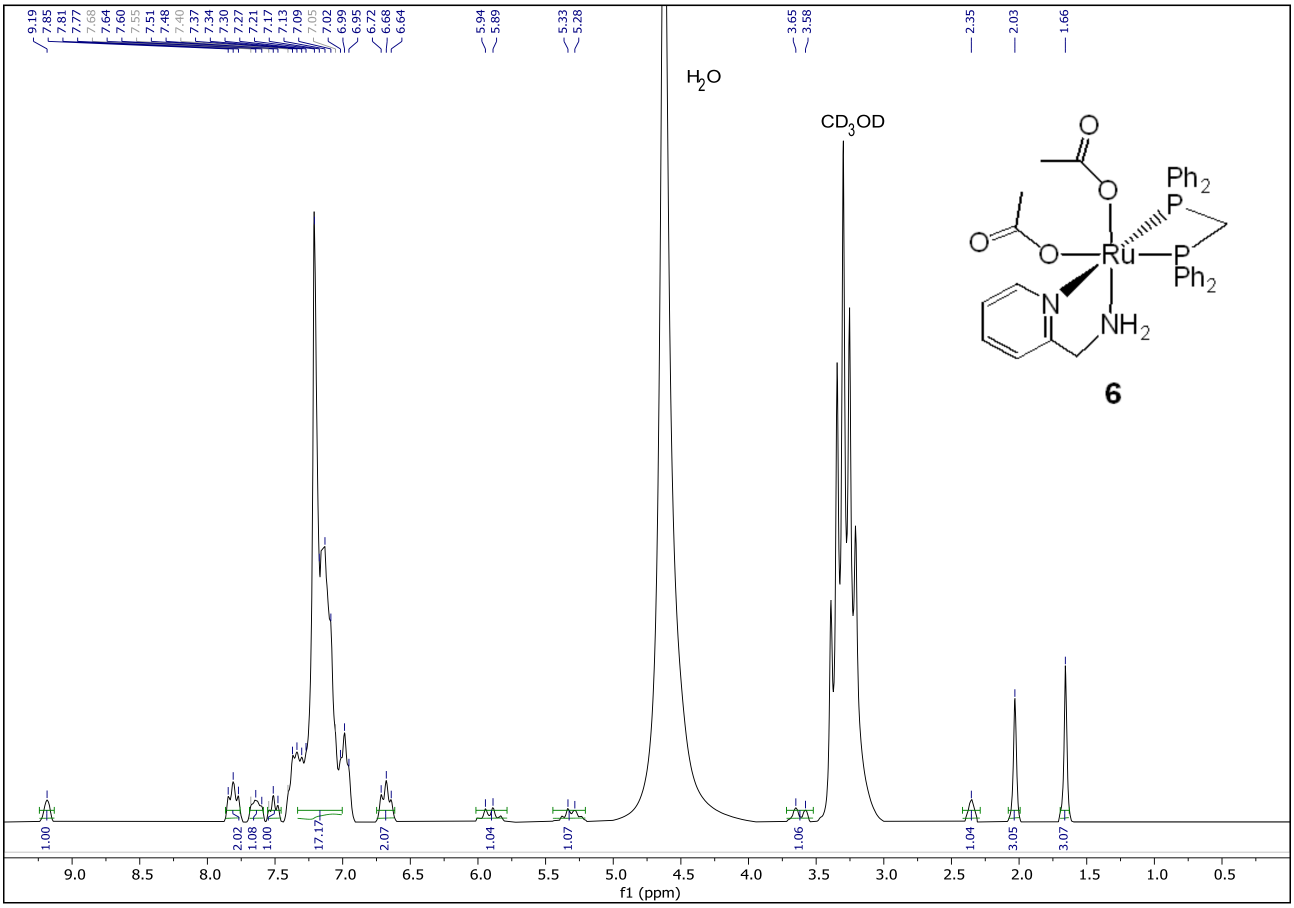

Figure S26. ^{1}H NMR spectrum (400.1 MHz) of *cis*-[Ru(η^{1}-OAc)$_2$(dppm)(ampy)] (**6**) in CD_3OD at 25 °C.

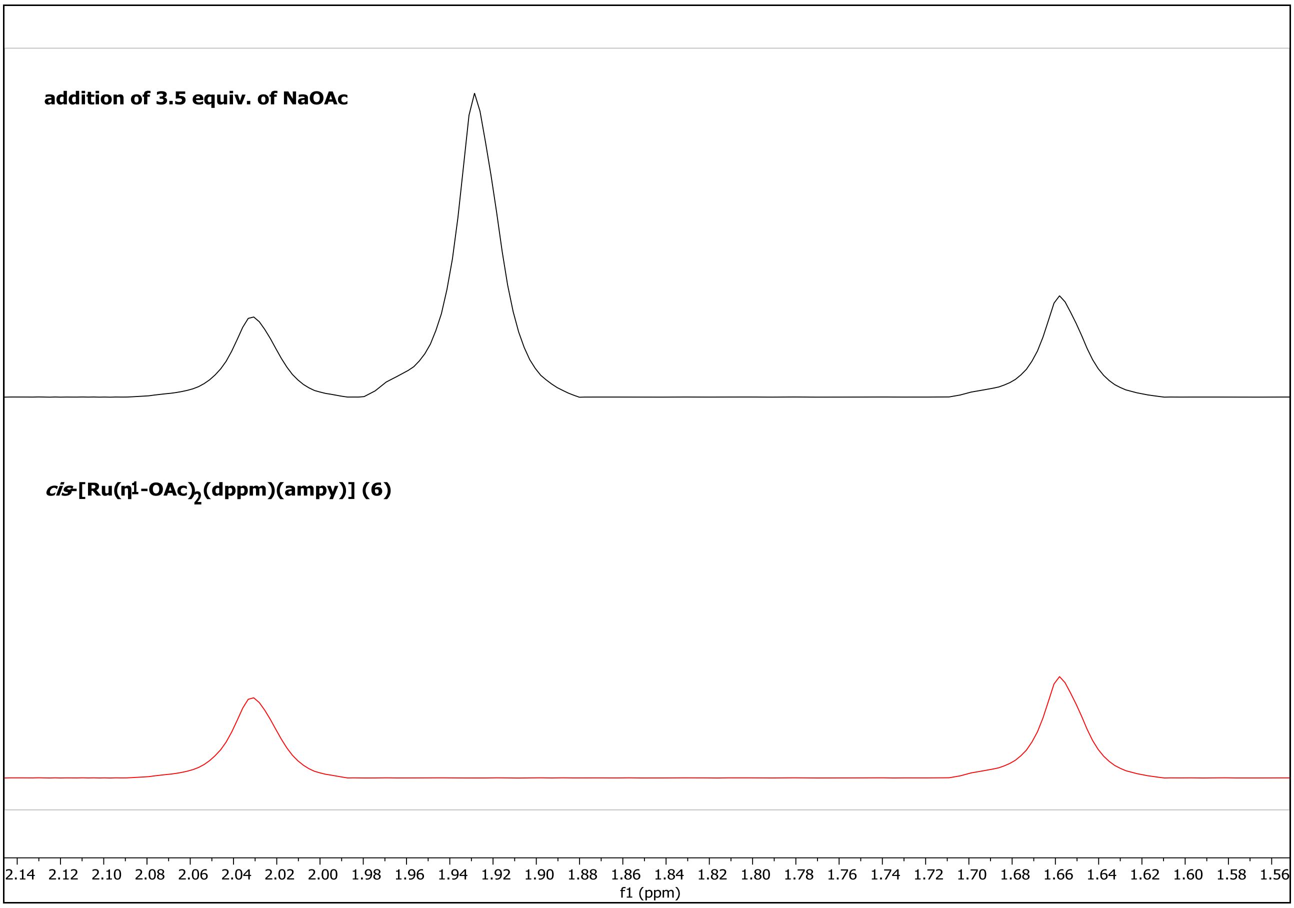

Figure S27. Effect of the addition of NaOAc (3.5 equiv) to *cis*-[Ru(η^1-OAc)$_2$(dppm)(ampy)] (**6**) in the methyl acetate region of the ^{1}H NMR spectrum (400.1 MHz) in CD_3OD at 25 °C.

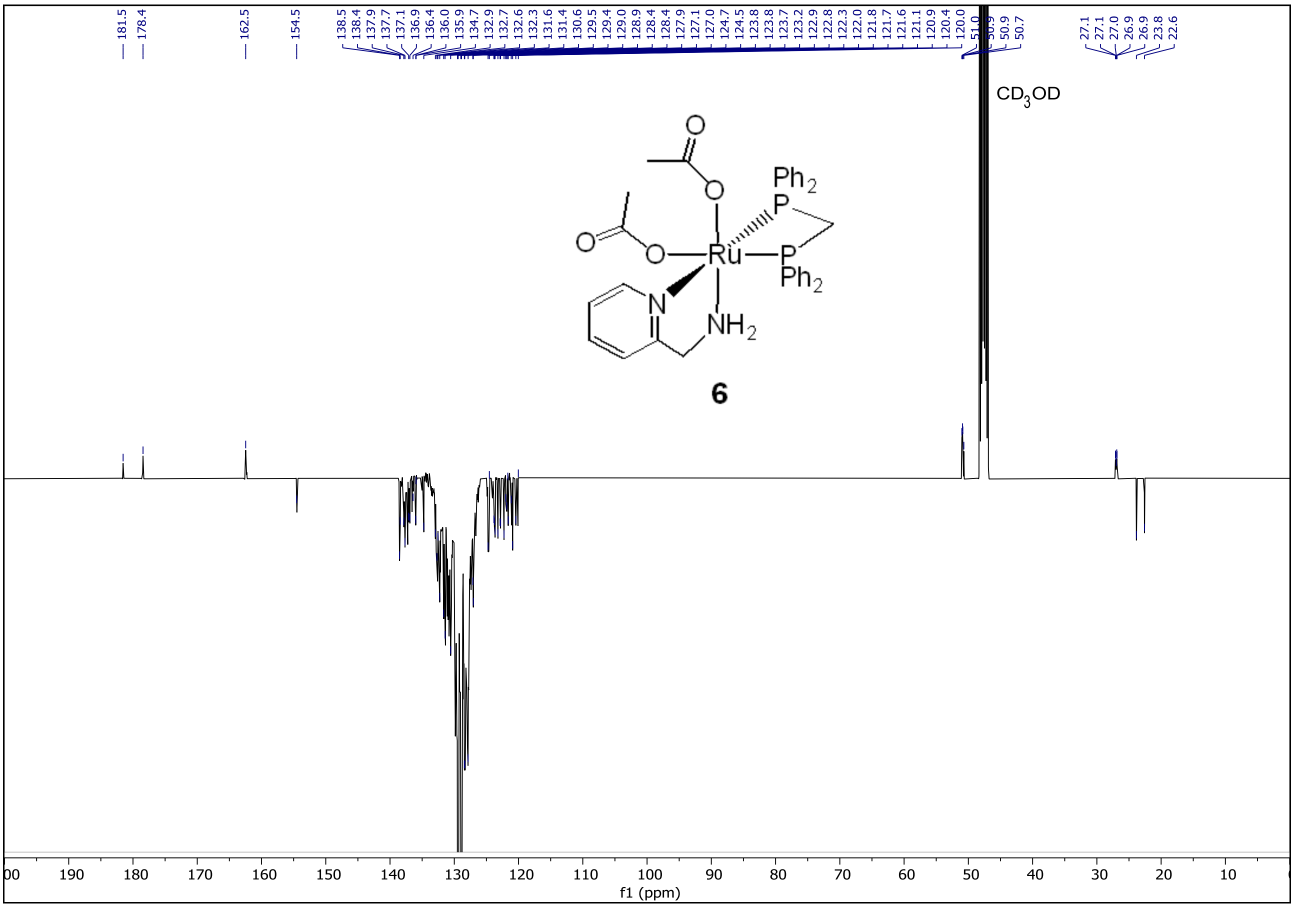

Figure S28. $^{13}C\{^{1}H\}$ DEPTQ NMR spectrum (100.6 MHz) of *cis*-[Ru(η^1-OAc)$_2$(dppm)(ampy)] (**6**) in CD_3OD at 25 °C.

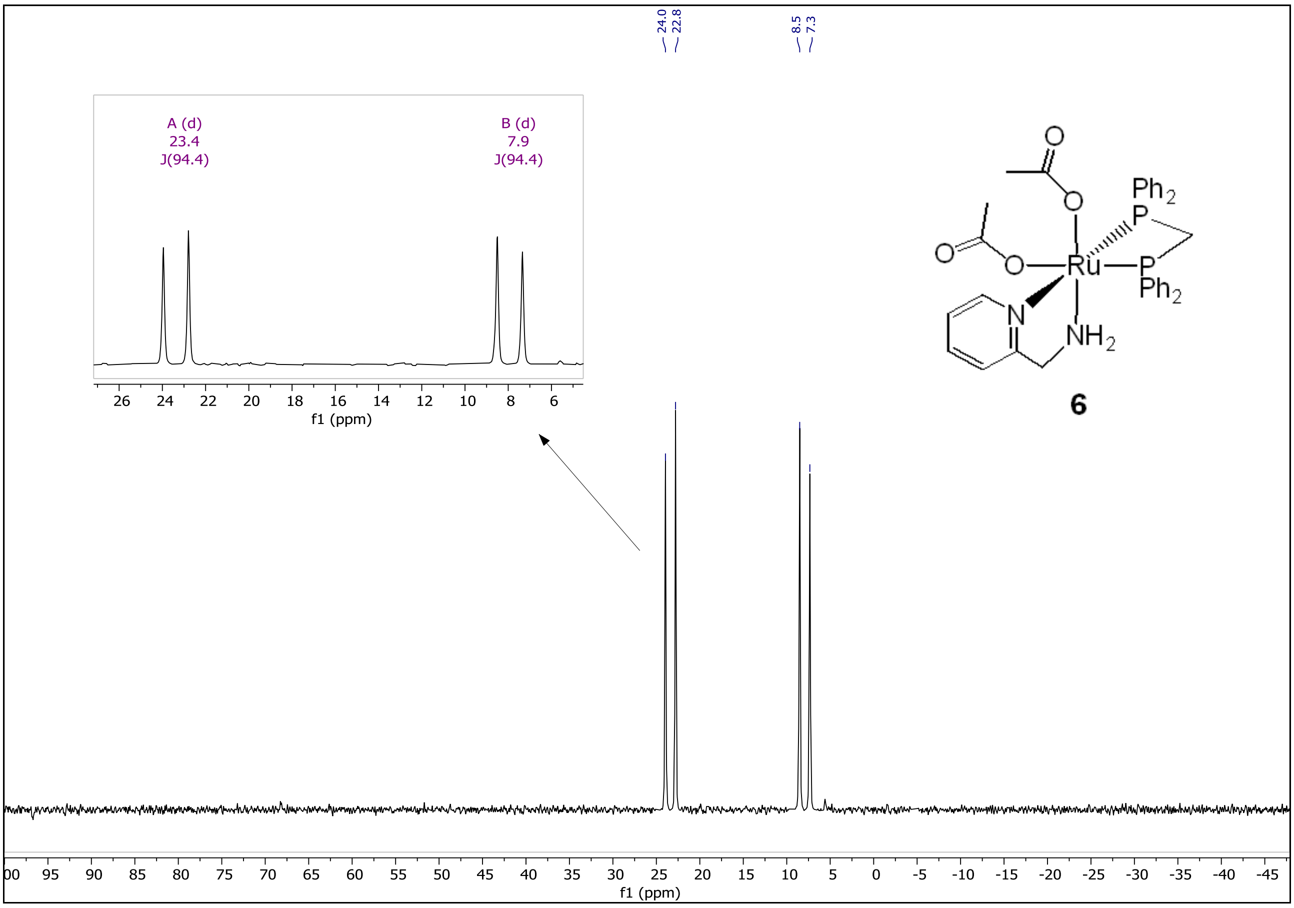

Figure S29. $^{31}P\{^{1}H\}$ NMR spectrum (81.0 MHz) of *cis*-[Ru(η^1-OAc)$_2$(dppm)(ampy)] (**6**) in $CDCl_3$ at 20 °C.

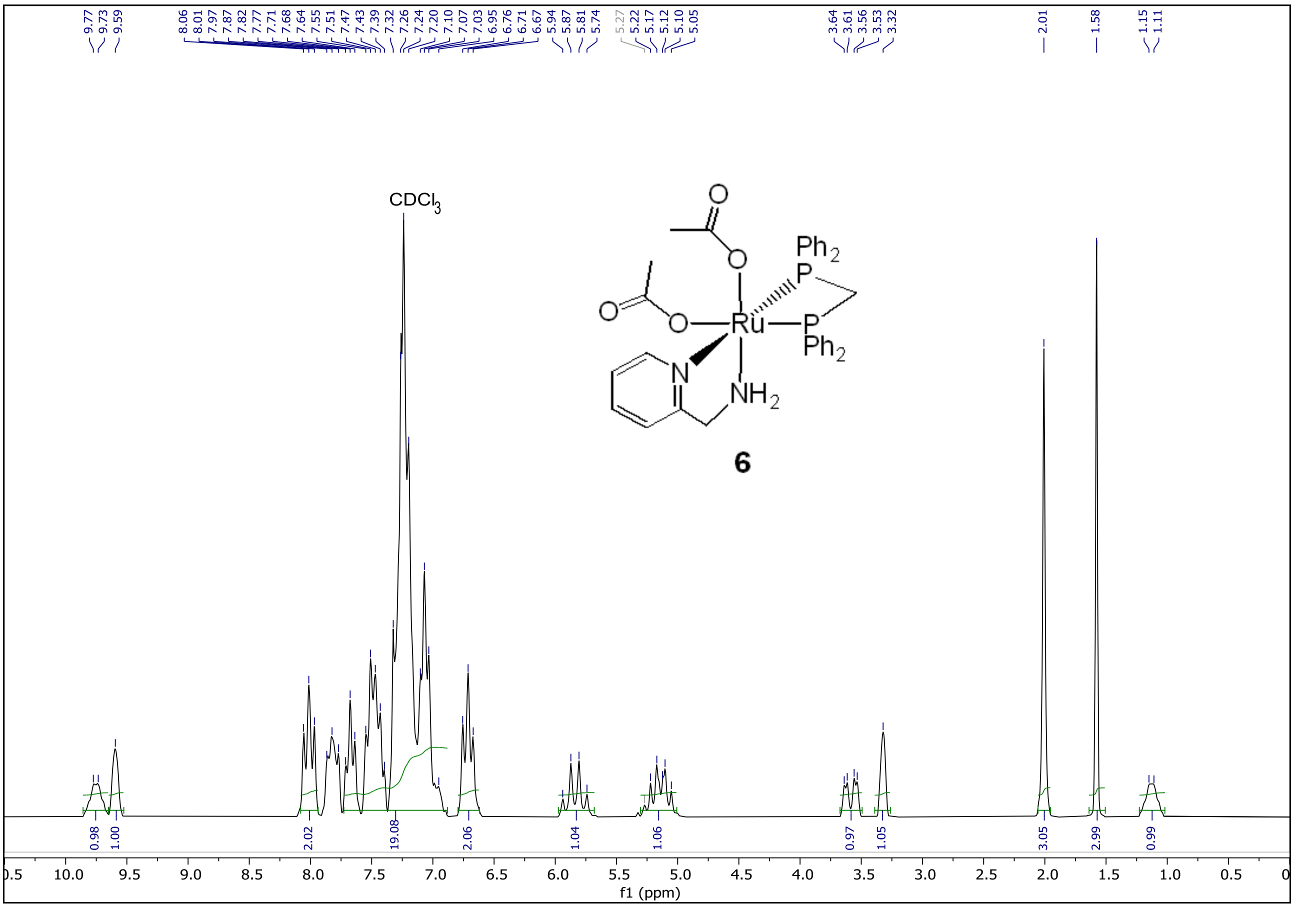

Figure S30. ^{1}H NMR spectrum (200.1 MHz) of *cis*-[Ru(η^1-OAc)$_2$(dppm)(ampy)] (**6**) in $CDCl_3$ at 20 °C.

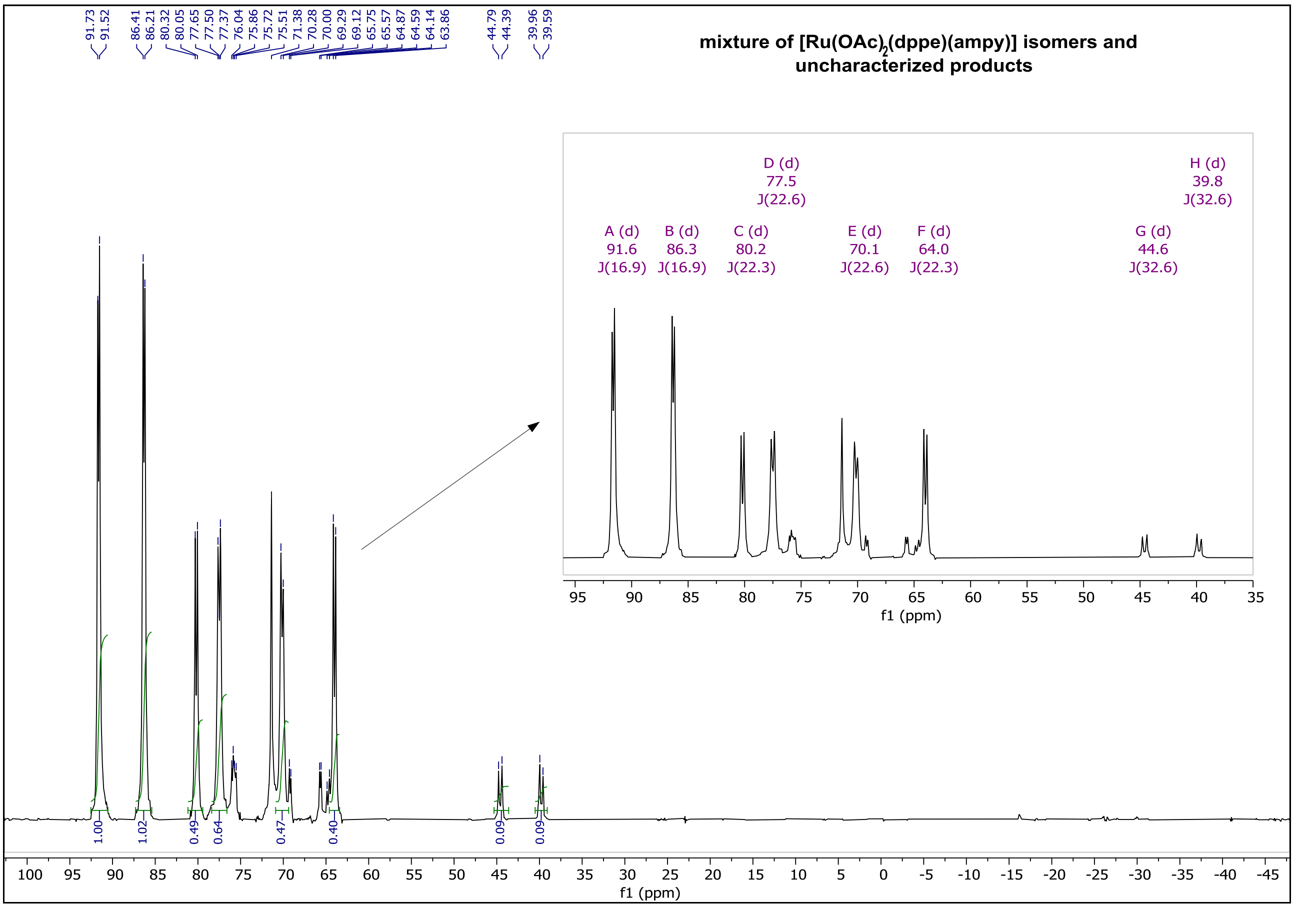

Figure S31. $^{31}P\{^1H\}$ NMR spectrum (81.0 MHz) of the mixture of [Ru(OAc)$_2$(dppe)(ampy)] isomers and uncharacterized products in toluene-d^8 at 20 °C.

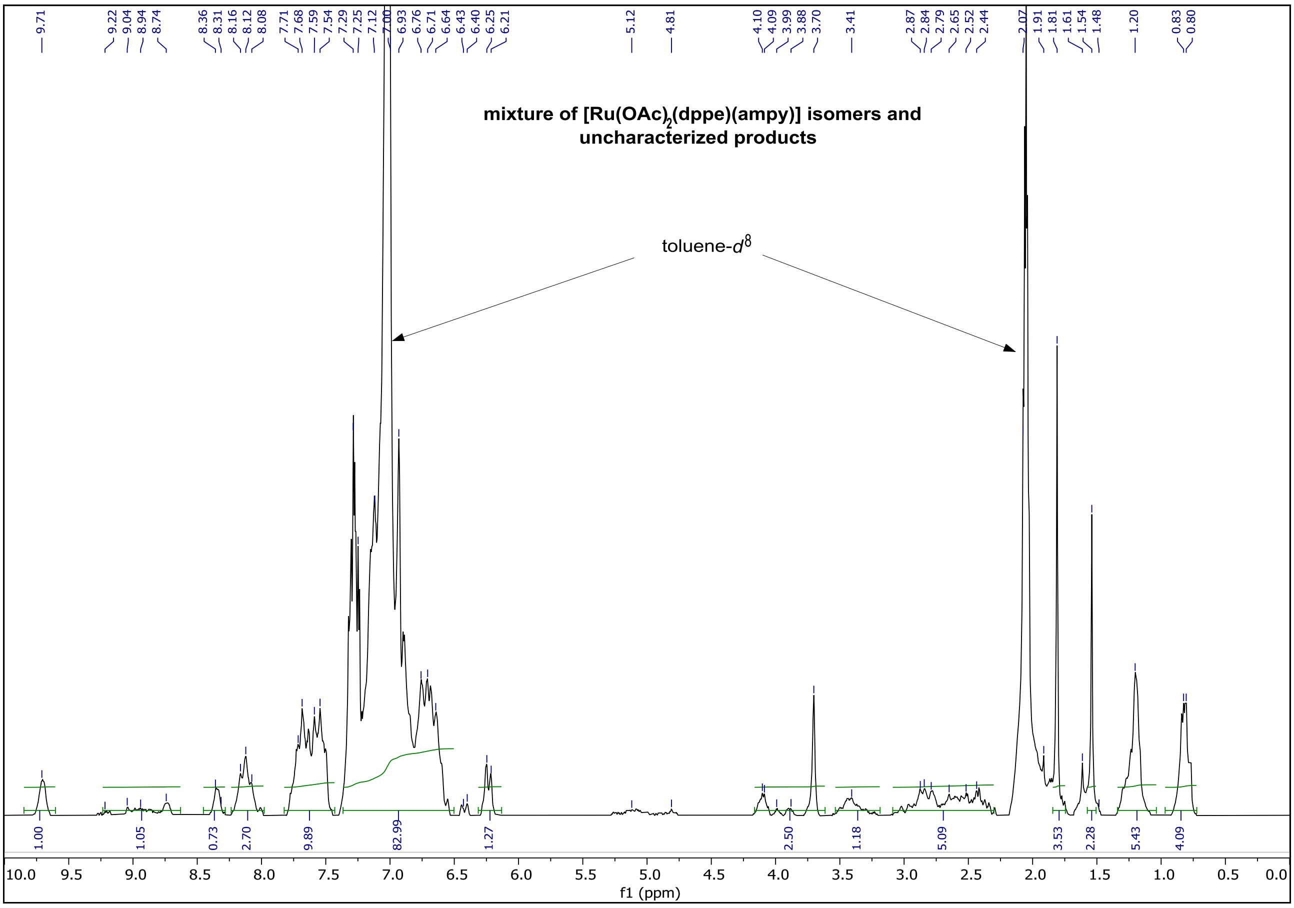

Figure S32. ^{1}H NMR spectrum (200.1 MHz) of the mixture of [Ru(OAc)$_2$(dppe)(ampy)] isomers and uncharacterized products in toluene-*d*^{8} at 20 °C.

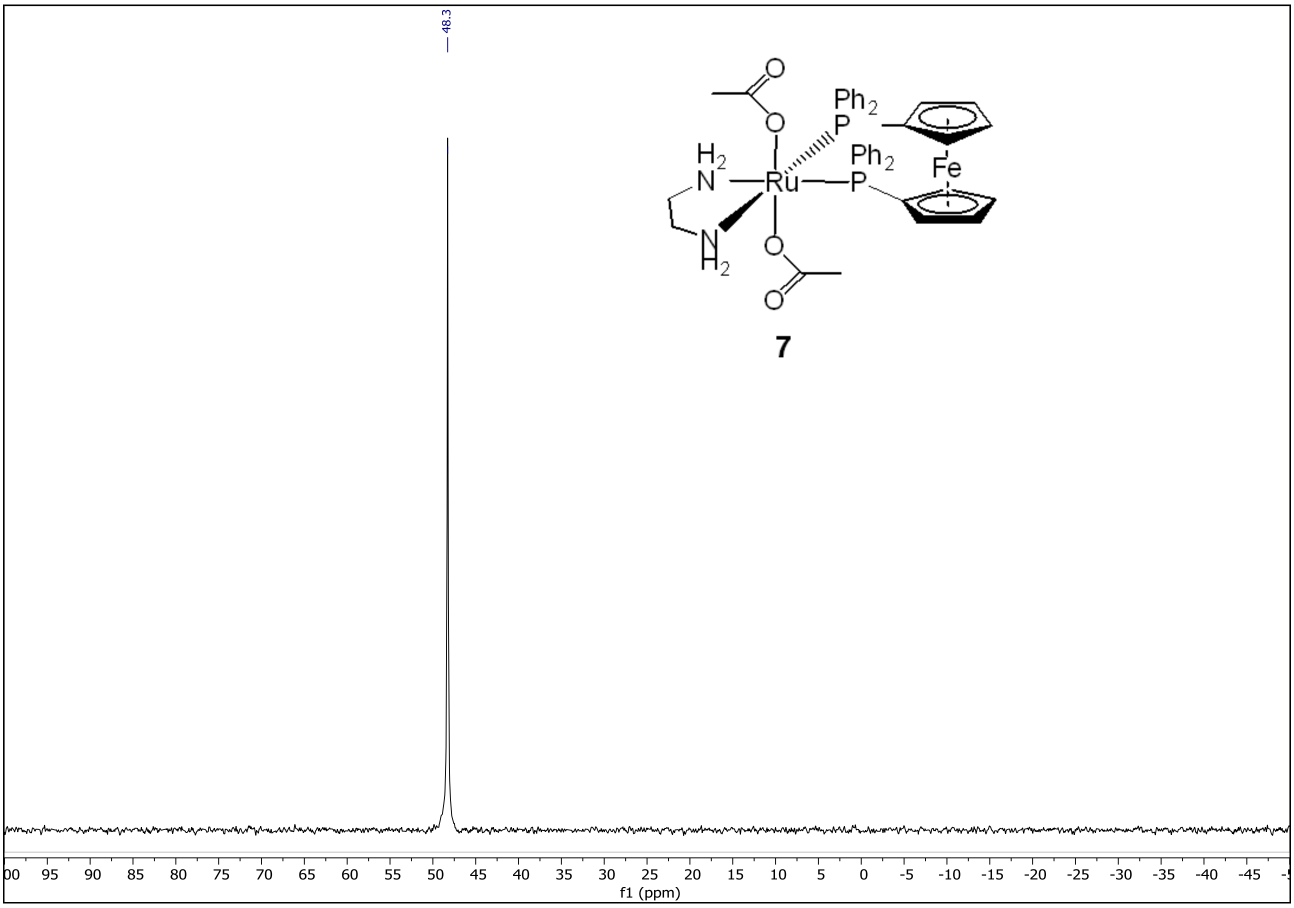

Figure S33. $^{31}P\{^{1}H\}$ NMR spectrum (81.0 MHz) of *trans*-[Ru(η^1-OAc)$_2$(dppf)(en)] (**7**) in CD_2Cl_2 at 20 °C.

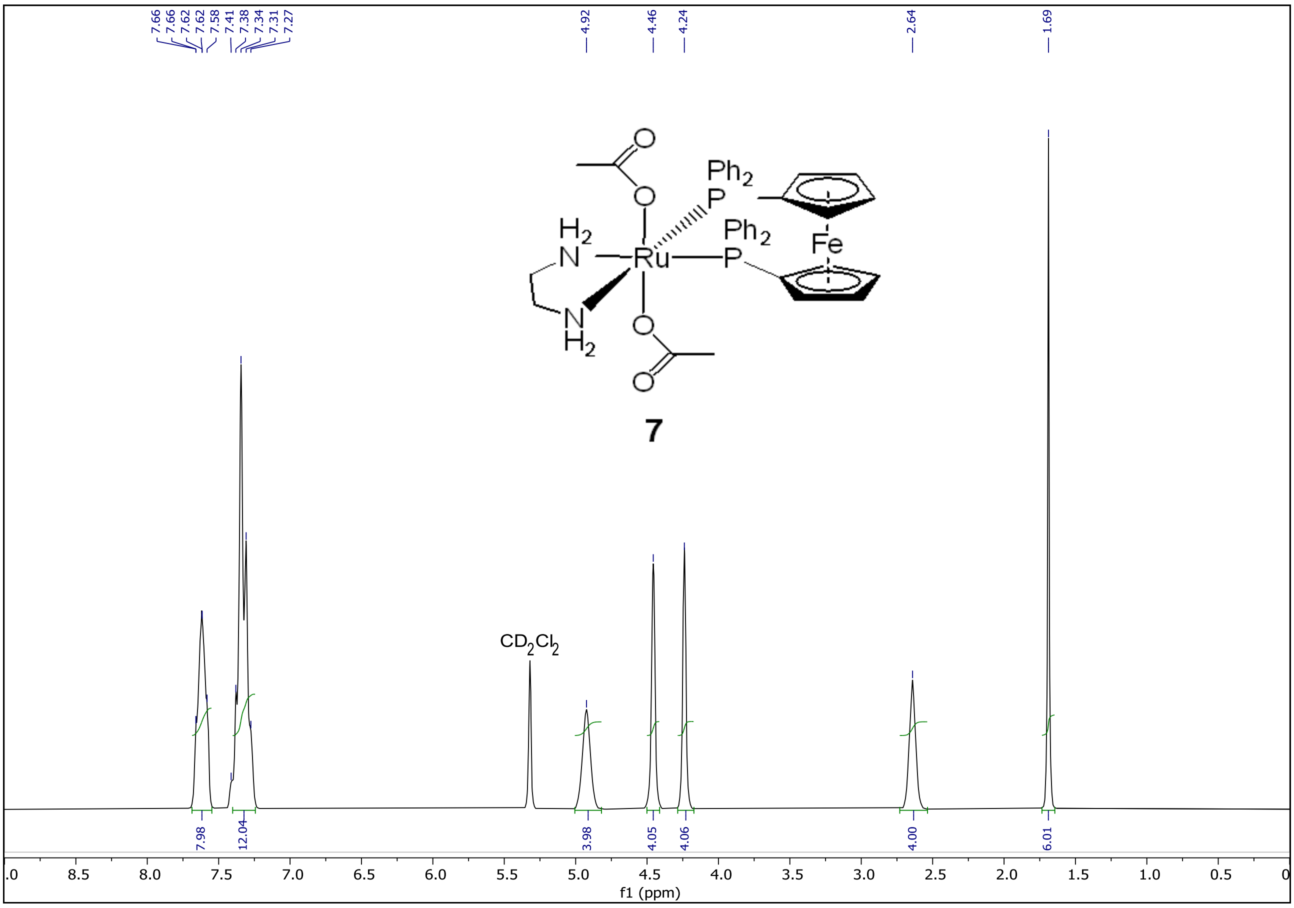

Figure S34. ^{1}H NMR spectrum (200.1 MHz) of *trans*-[Ru(η^1-OAc)$_2$(dppf)(en)] (**7**) in CD_2Cl_2 at 20 °C.

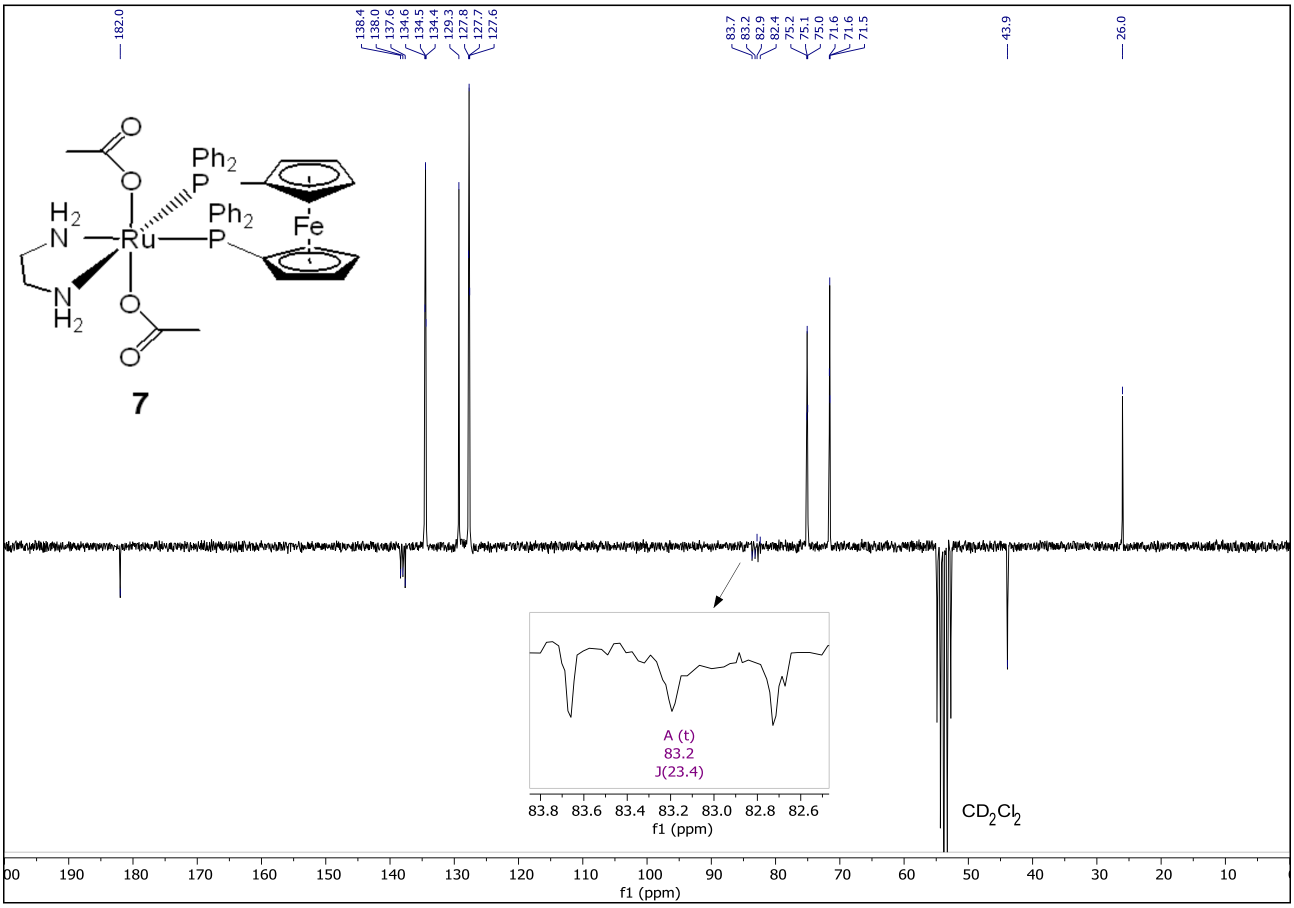

Figure S35. $^{13}C\{^{1}H\}$ PENDANT NMR spectrum (50.3 MHz) of *trans*-[Ru(η[1]-OAc)$_2$(dppf)(en)] (**7**) in CD_2Cl_2 at 20 °C.

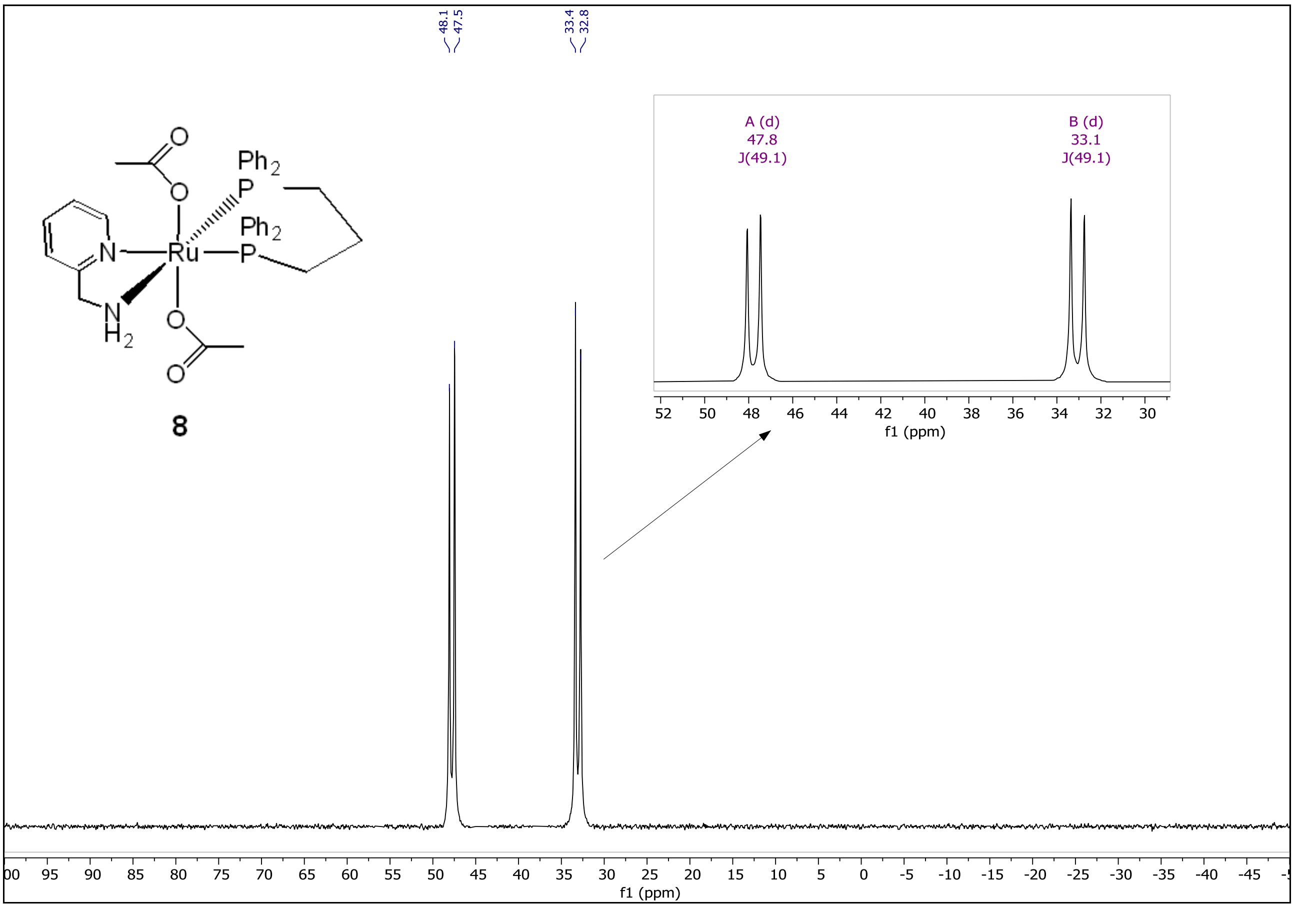

Figure S36. $^{31}P\{^1H\}$ NMR spectrum (81.0 MHz) of *trans*-[Ru(η^1-OAc)$_2$(dppp)(ampy)] (**8**) in CD_2Cl_2 at 20 °C.

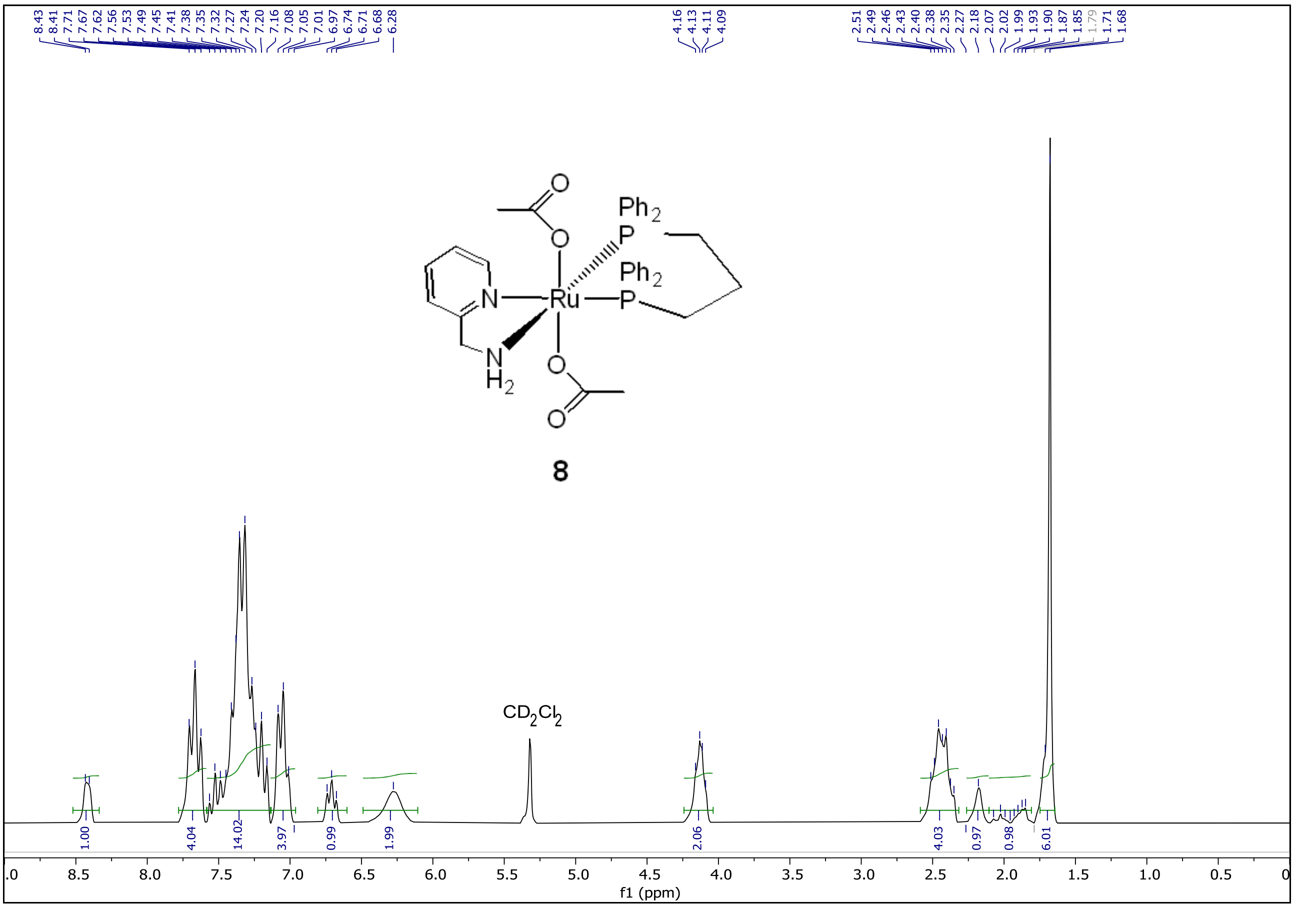

Figure S37. ^{1}H NMR spectrum (200.1 MHz) of *trans*-[Ru(η^1-OAc)$_2$(dppp)(ampy)] (**8**) in CD_2Cl_2 at 20 °C.

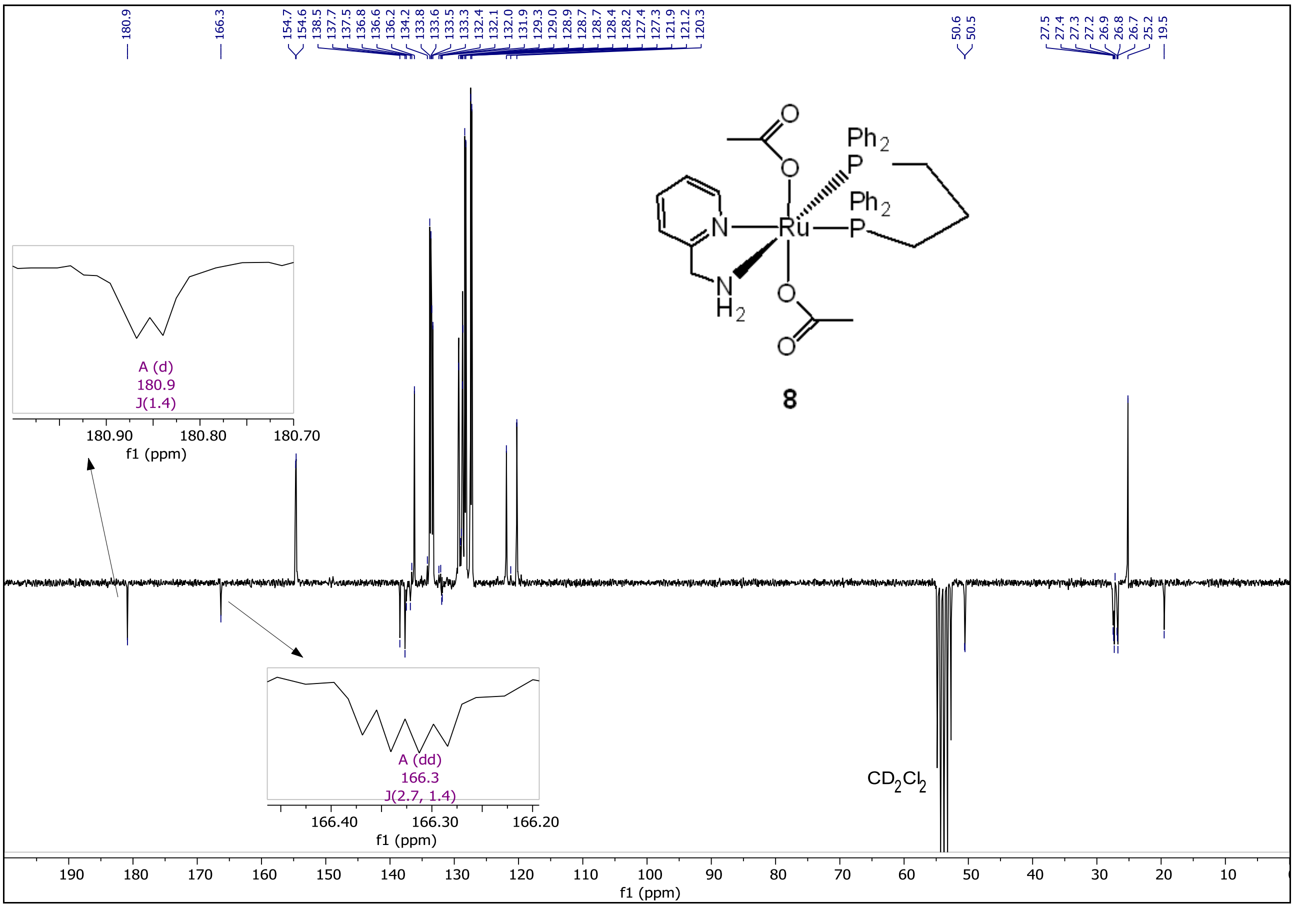

Figure S38. $^{13}C\{^{1}H\}$ PENDANT NMR spectrum (50.3 MHz) of *trans*-[Ru(η^1-OAc)$_2$(dppp)(ampy)] (**8**) in CD_2Cl_2 at 20 °C.

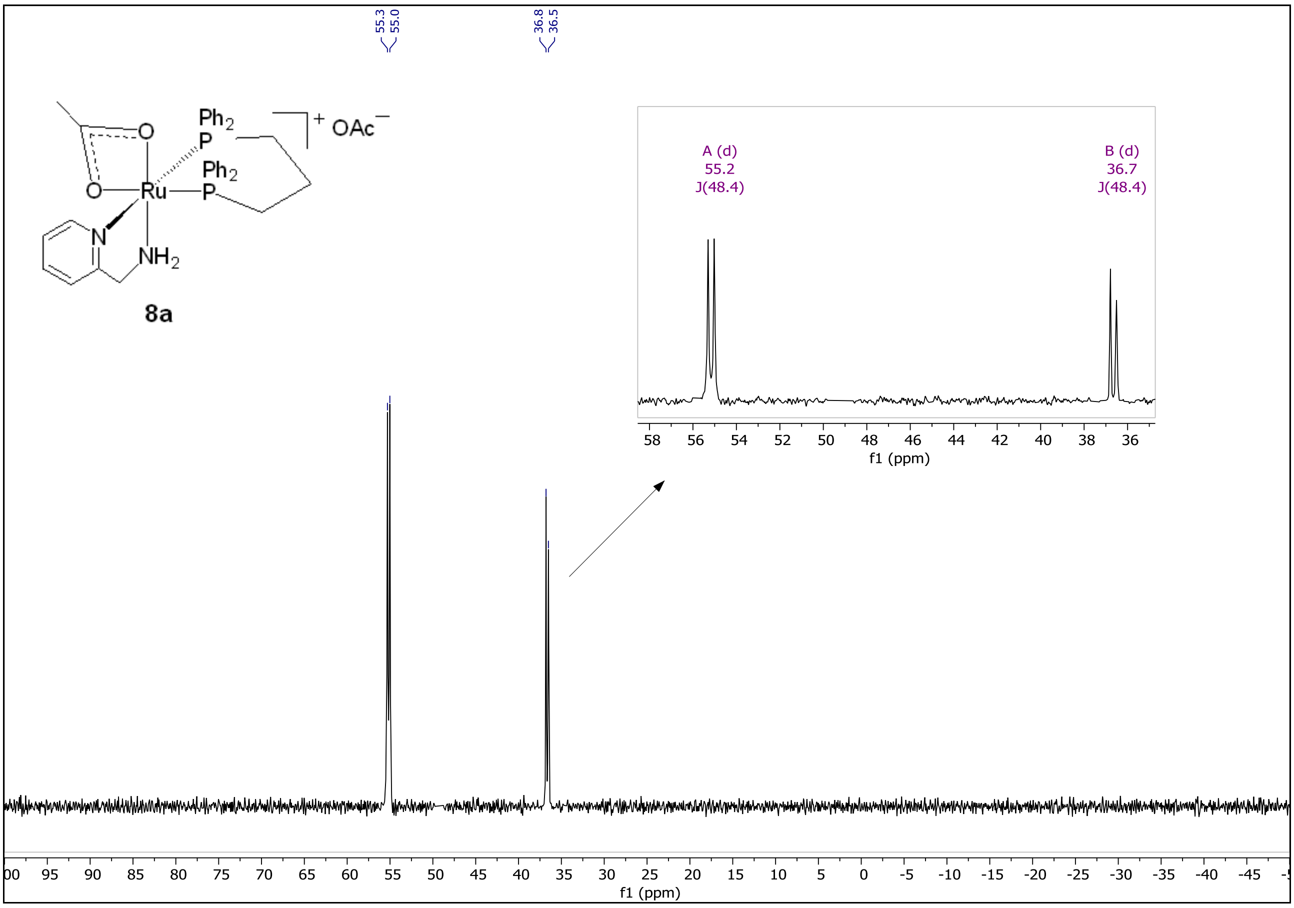

Figure S39. $^{31}P\{^{1}H\}$ NMR spectrum (162.0 MHz) of [Ru(η^2-OAc)(dppp)(ampy)]OAc (**8a**) in CD_3OD at 25 °C.

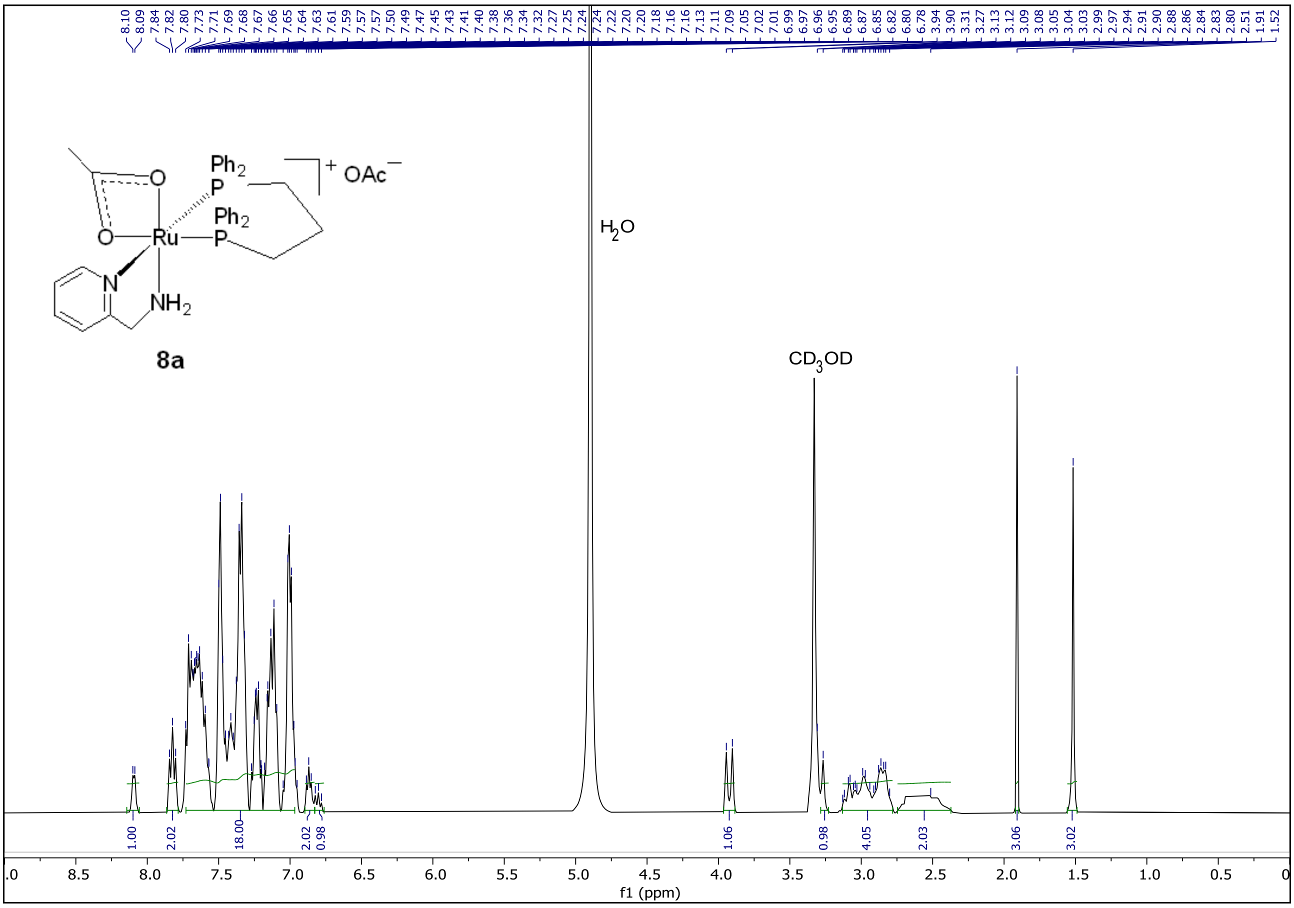

Figure S40. ^{1}H NMR spectrum (400.1 MHz) of [Ru(η^2-OAc)(dppp)(ampy)]OAc (**8a**) in CD_3OD at 25 °C.

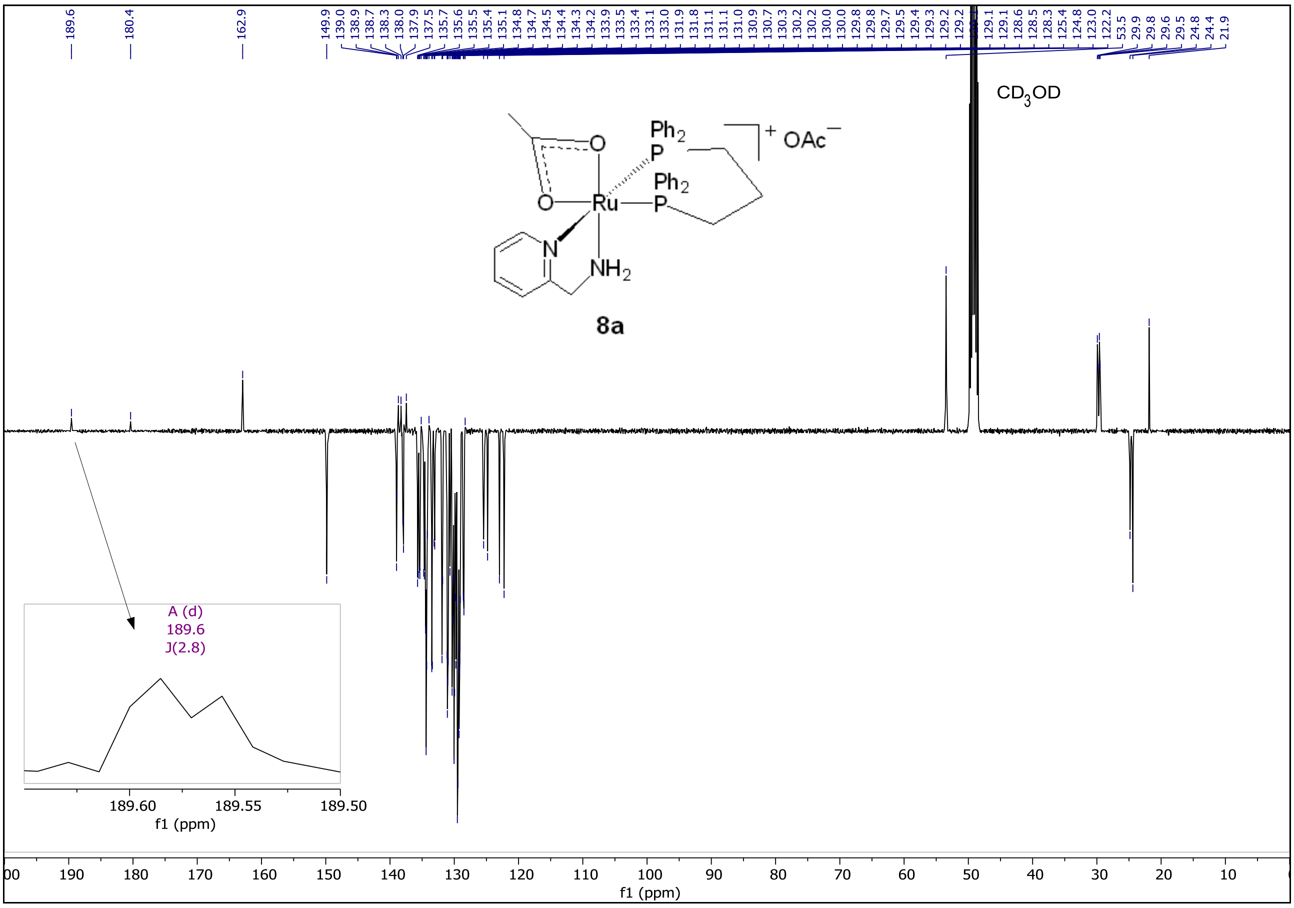

Figure S41. $^{13}C\{^1H\}$ DEPTQ NMR spectrum (100.6 MHz) of [Ru(η^2-OAc)(dppp)(ampy)]OAc (**8a**) in CD_3OD at 25 °C.

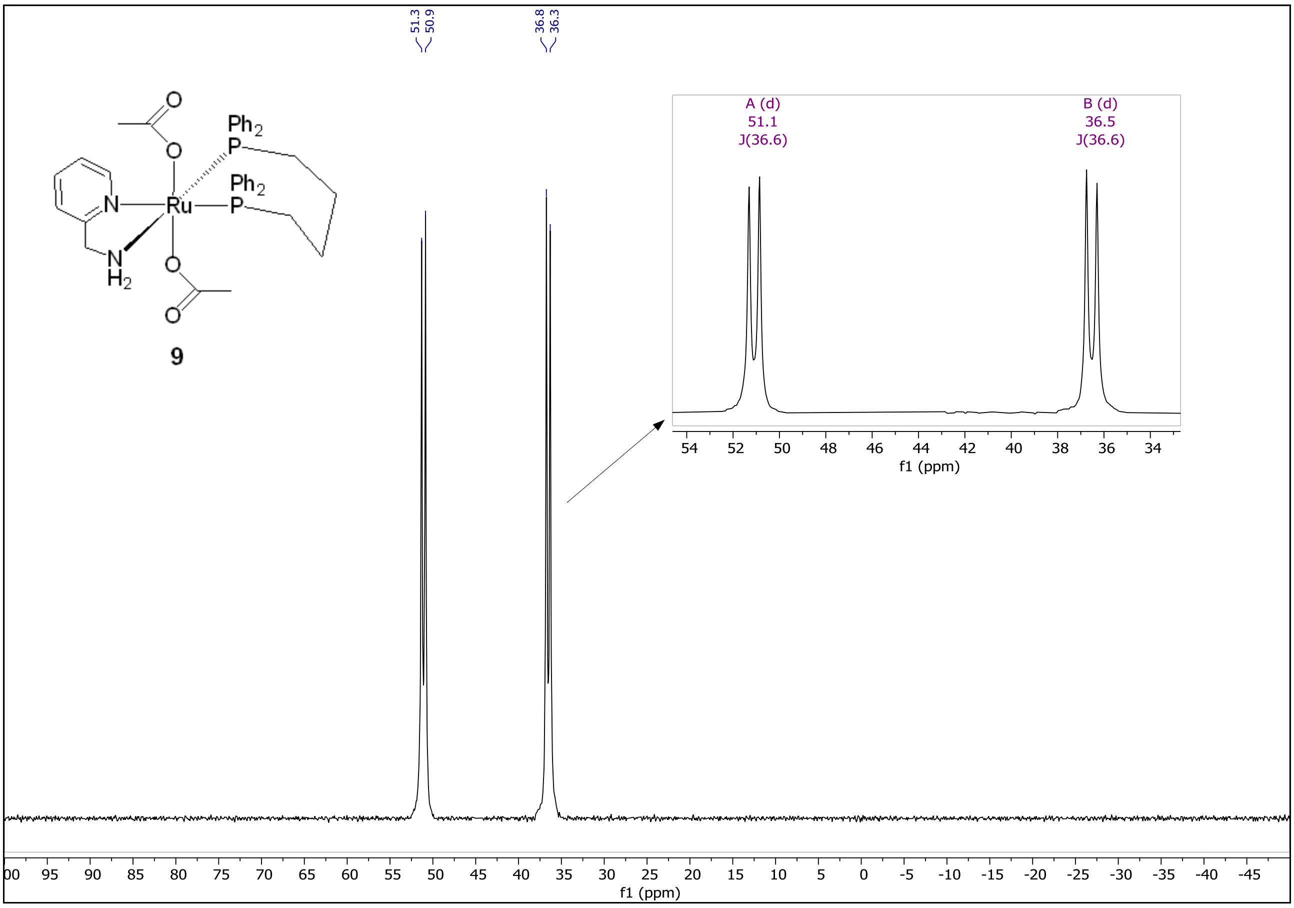

Figure S42. $^{31}P\{^{1}H\}$ NMR spectrum (81.0 MHz) of *trans*-[Ru(η^1-OAc)$_2$(dppb)(ampy)] (**9**) in CD_2Cl_2 at 20 °C.

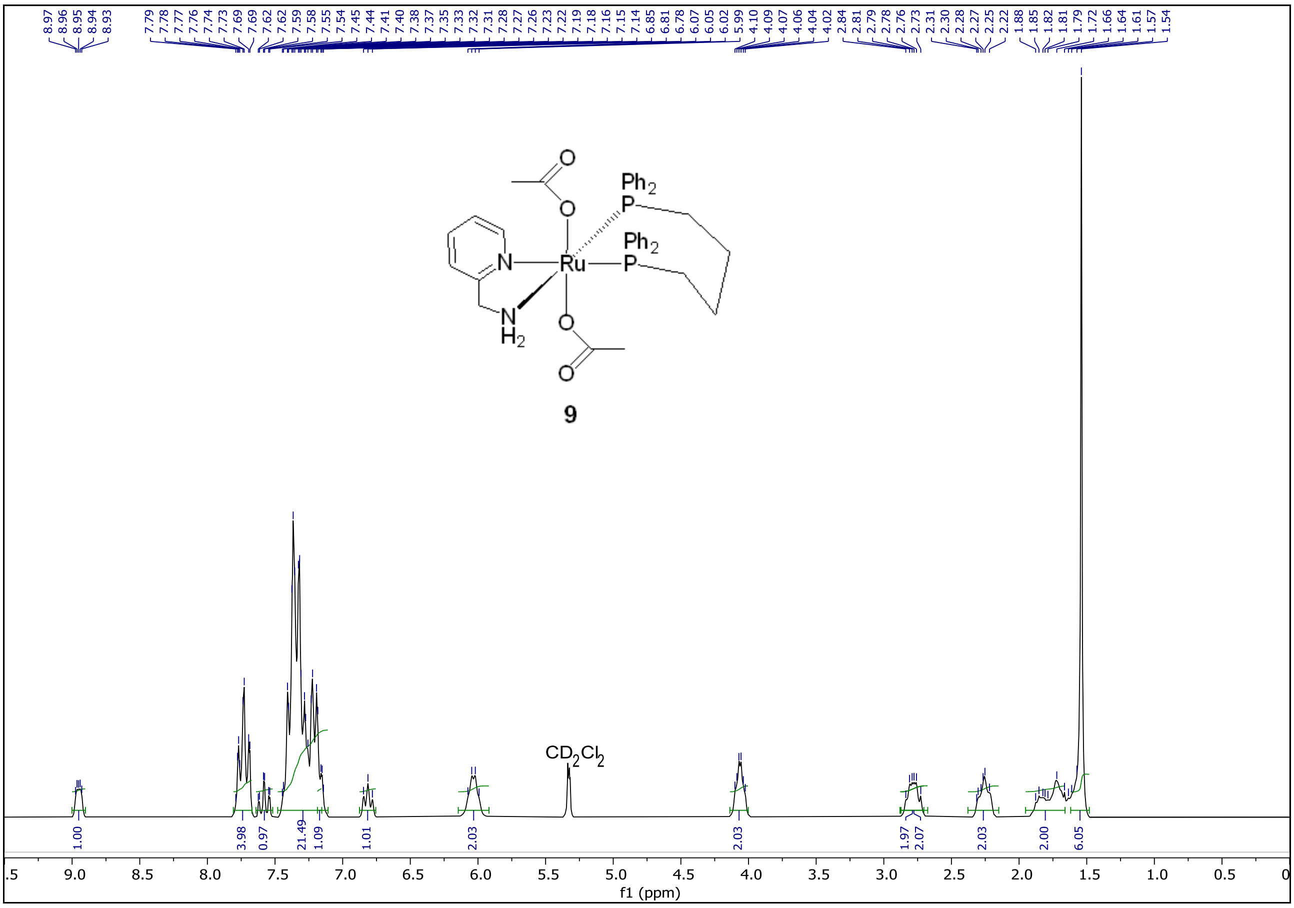

Figure S43. ^{1}H NMR spectrum (200.1 MHz) of *trans*-[Ru(η^1-OAc)$_2$(dppb)(ampy)] (**9**) in CD_2Cl_2 at 20 °C.

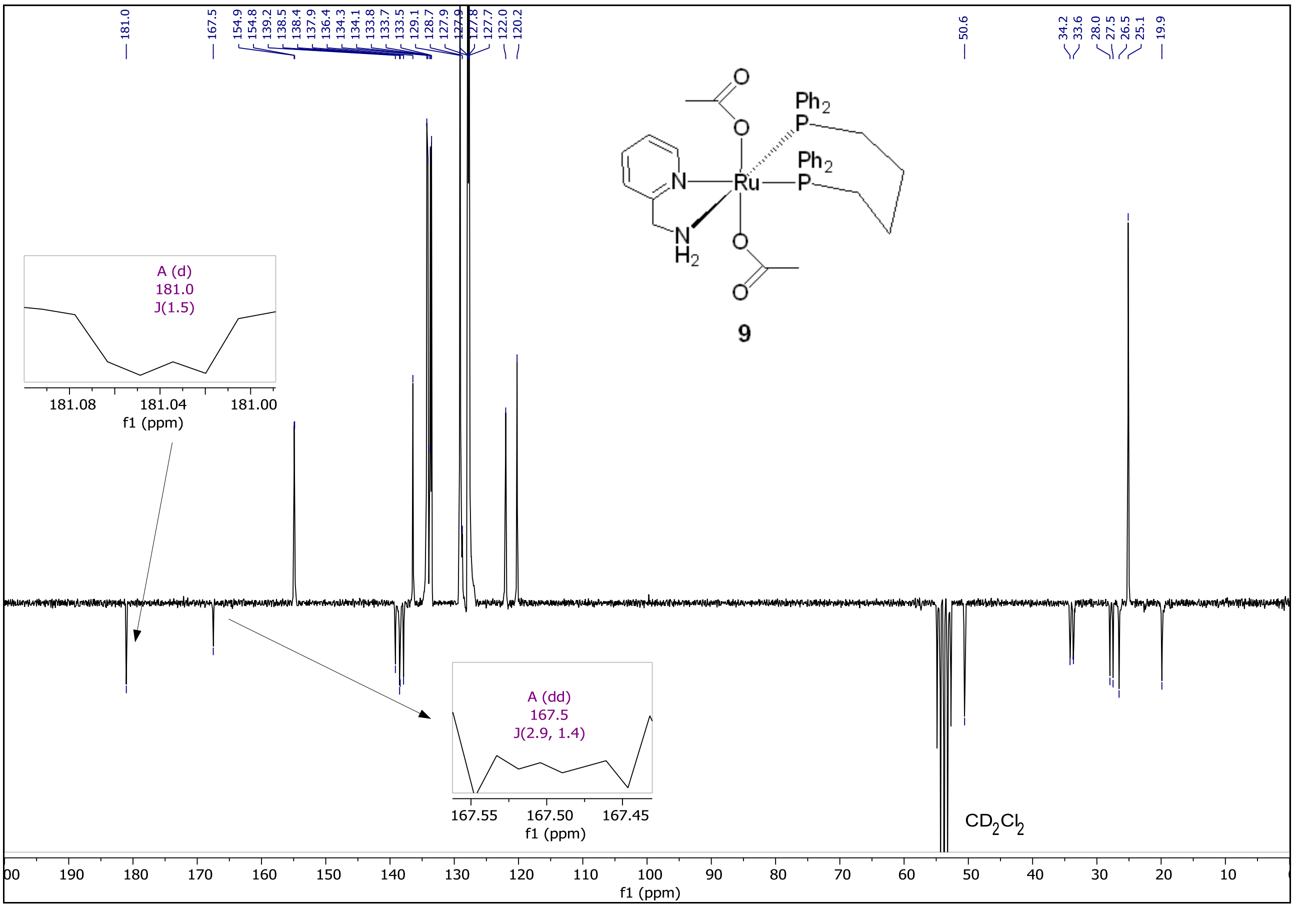

Figure S44. $^{13}C\{^1H\}$ PENDANT NMR spectrum (50.3 MHz) of *trans*-[Ru(η^1-OAc)$_2$(dppb)(ampy)] (**9**) in CD_2Cl_2 at 20 °C.

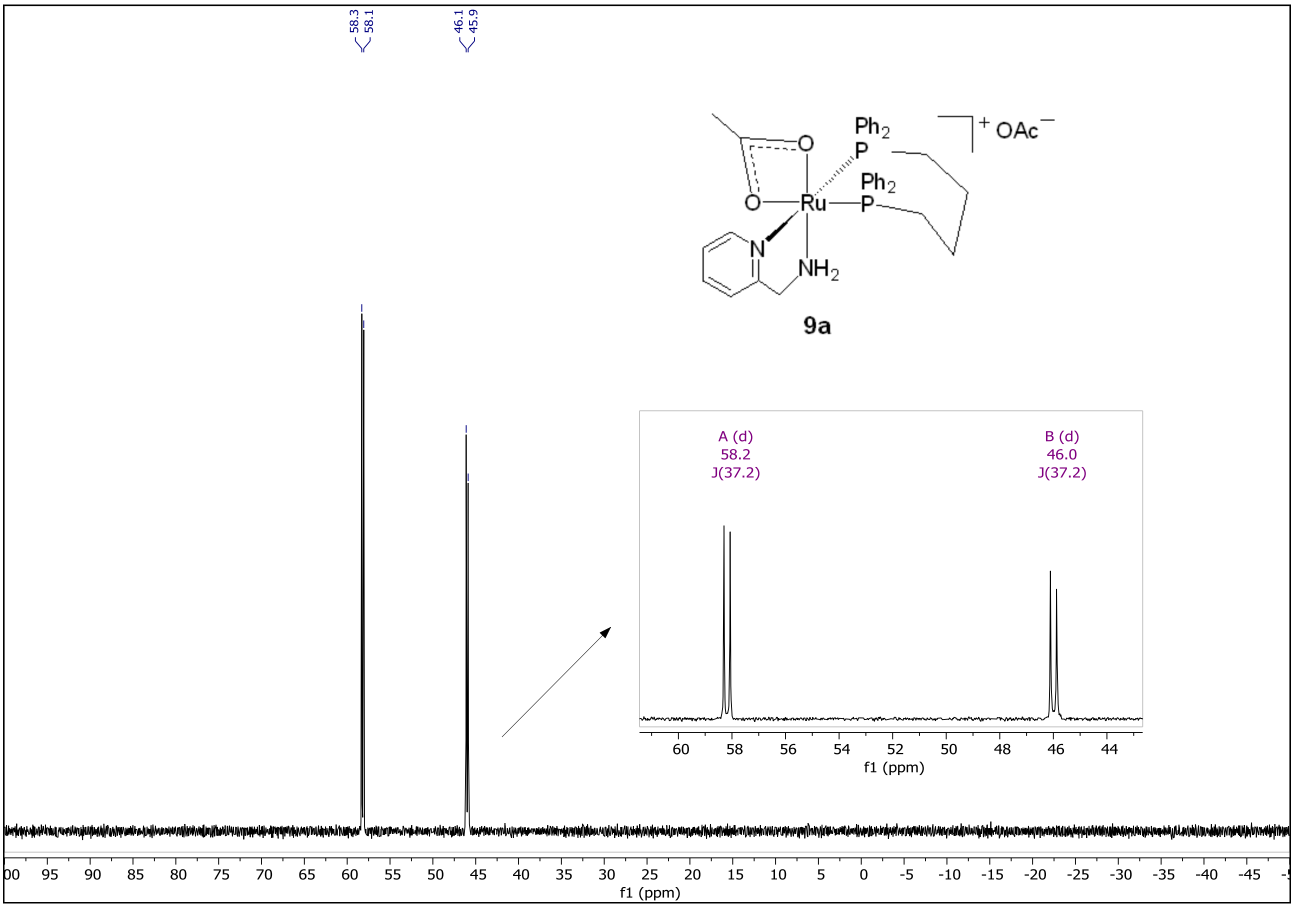

Figure S45. $^{31}P\{^{1}H\}$ NMR spectrum (162.0 MHz) of [Ru(η^2-OAc)(dppb)(ampy)]OAc (**9a**) in CD_3OD at 25 °C.

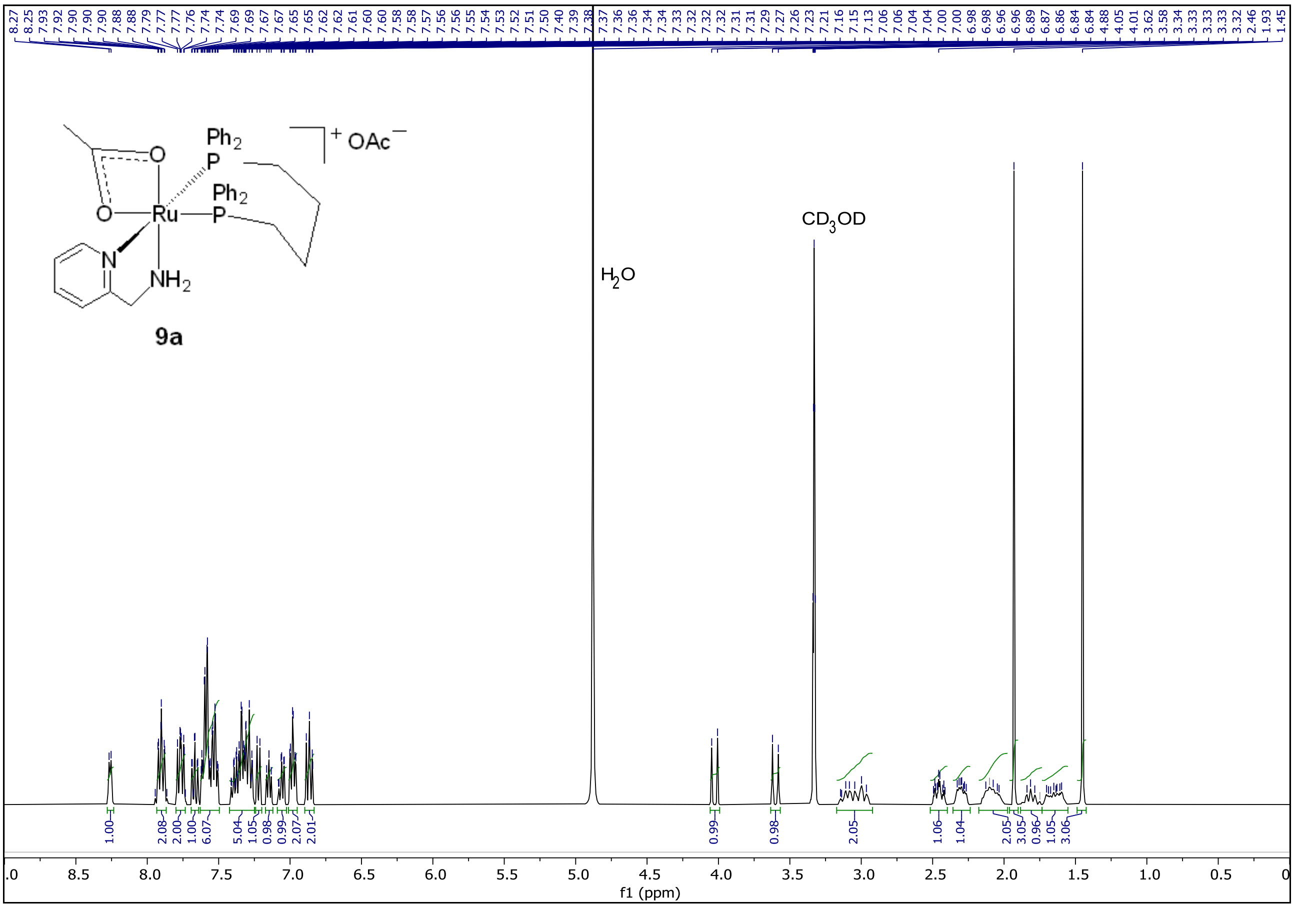

Figure S46. ^{1}H NMR spectrum (400.1 MHz) of [Ru(η^2-OAc)(dppb)(ampy)]OAc (**9a**) in CD_3OD at 25 °C.

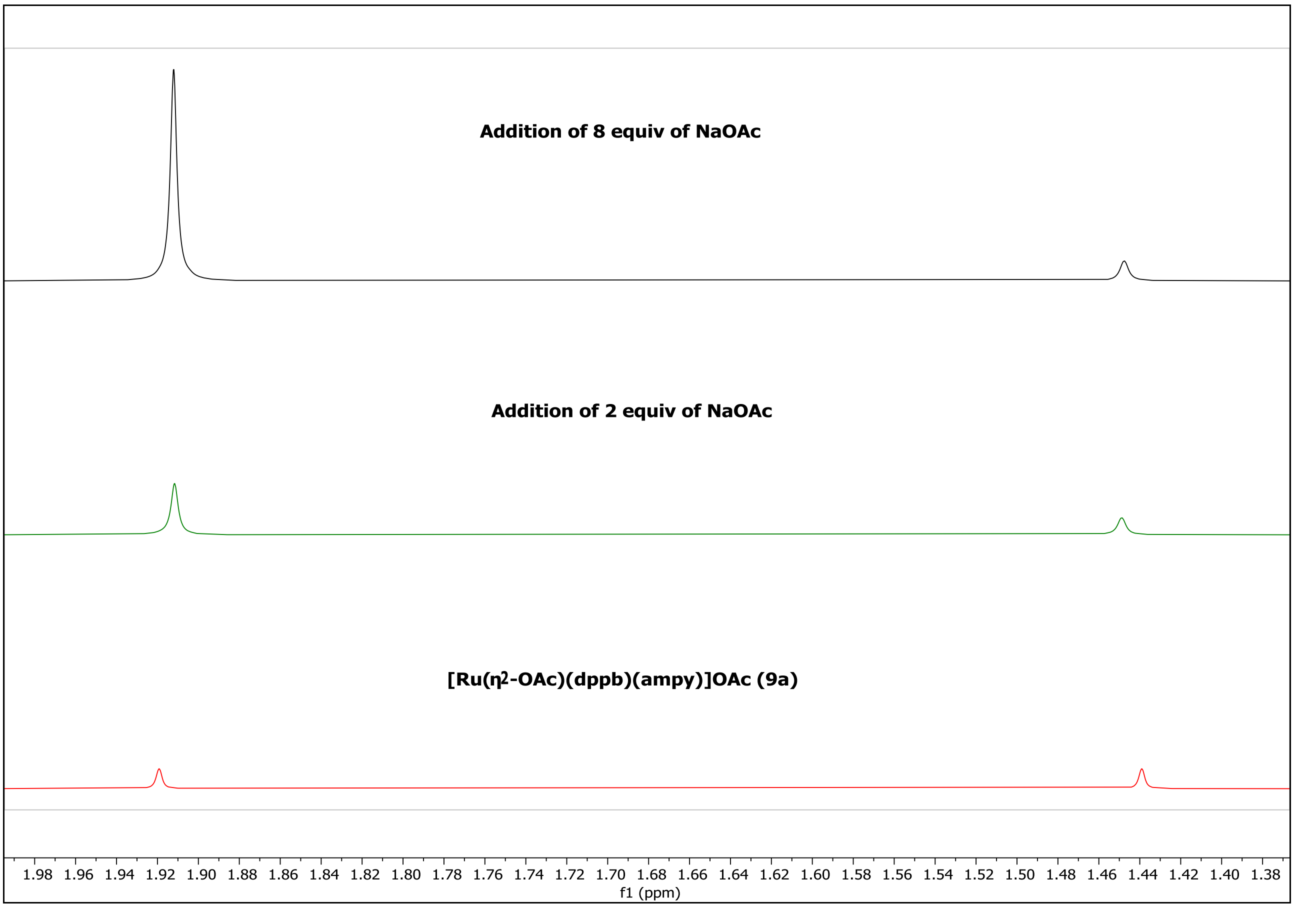

Figure S47. Effect of the addition of NaOAc (2-8 equiv) to [Ru(η^2-OAc)(dppb)(ampy)]OAc (**9a**) in the methyl acetate region of the ^{1}H NMR spectrum (400.1 MHz) in CD_3OD at 25 °C.

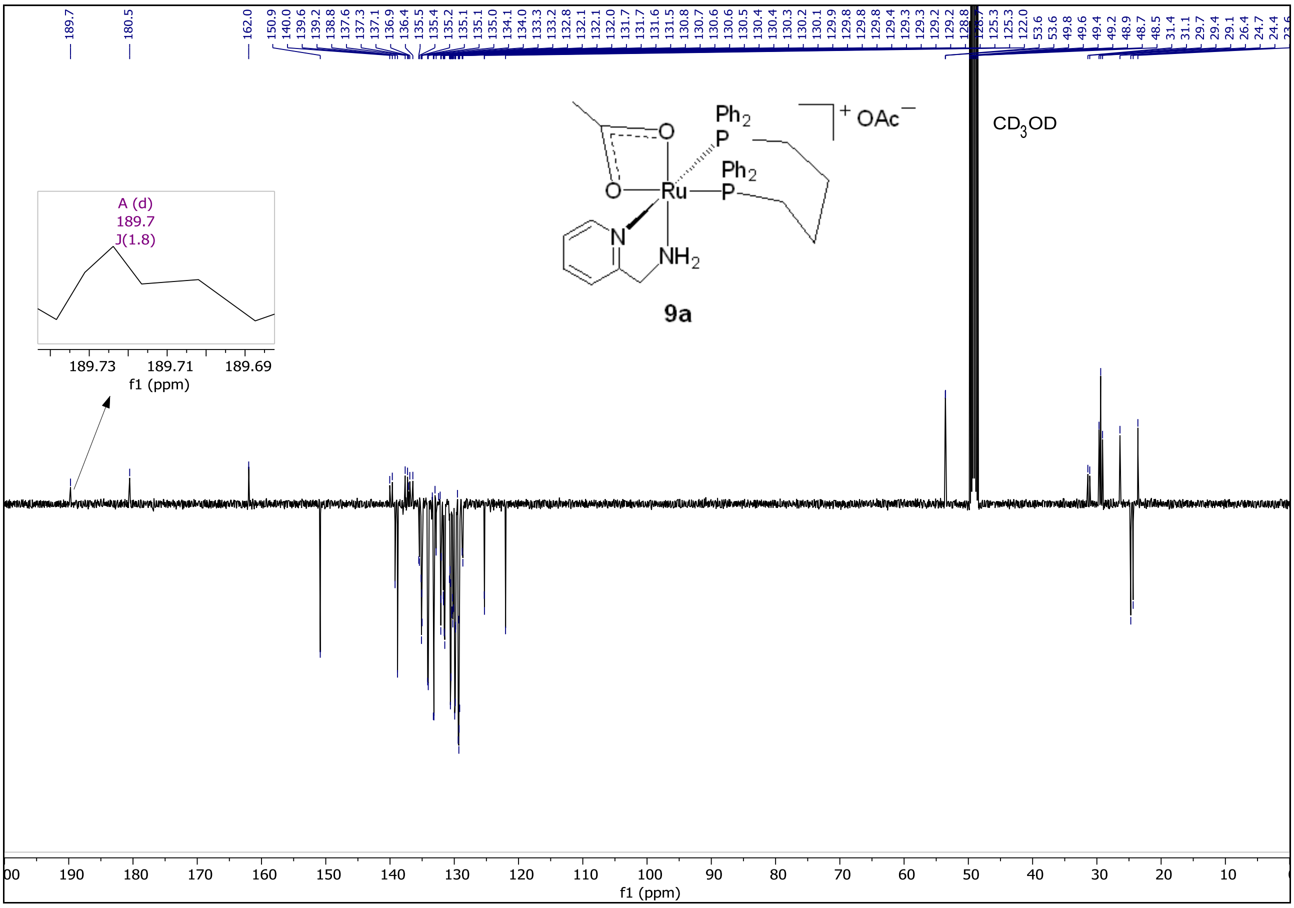

Figure S48. $^{13}C\{^{1}H\}$ DEPTQ NMR spectrum (100.6 MHz) of [Ru(η^2-OAc)(dppb)(ampy)]OAc (**9a**) in CD_3OD at 25 °C.

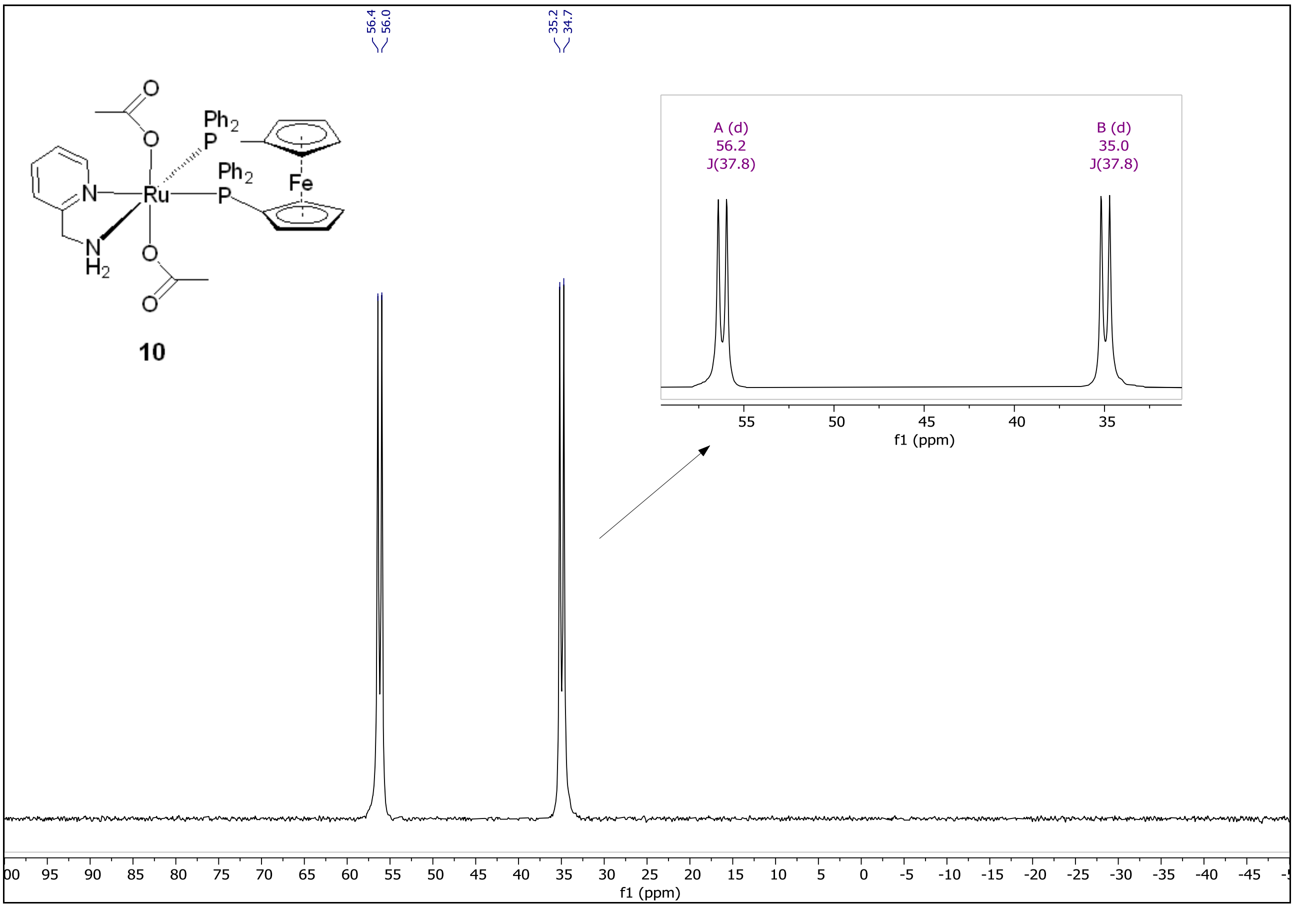

Figure S49. $^{31}P\{^1H\}$ NMR spectrum (81.0 MHz) of *trans*-[Ru(η^1-OAc)$_2$(dppf)(ampy)] (**10**) in CD_2Cl_2 at 20 °C.

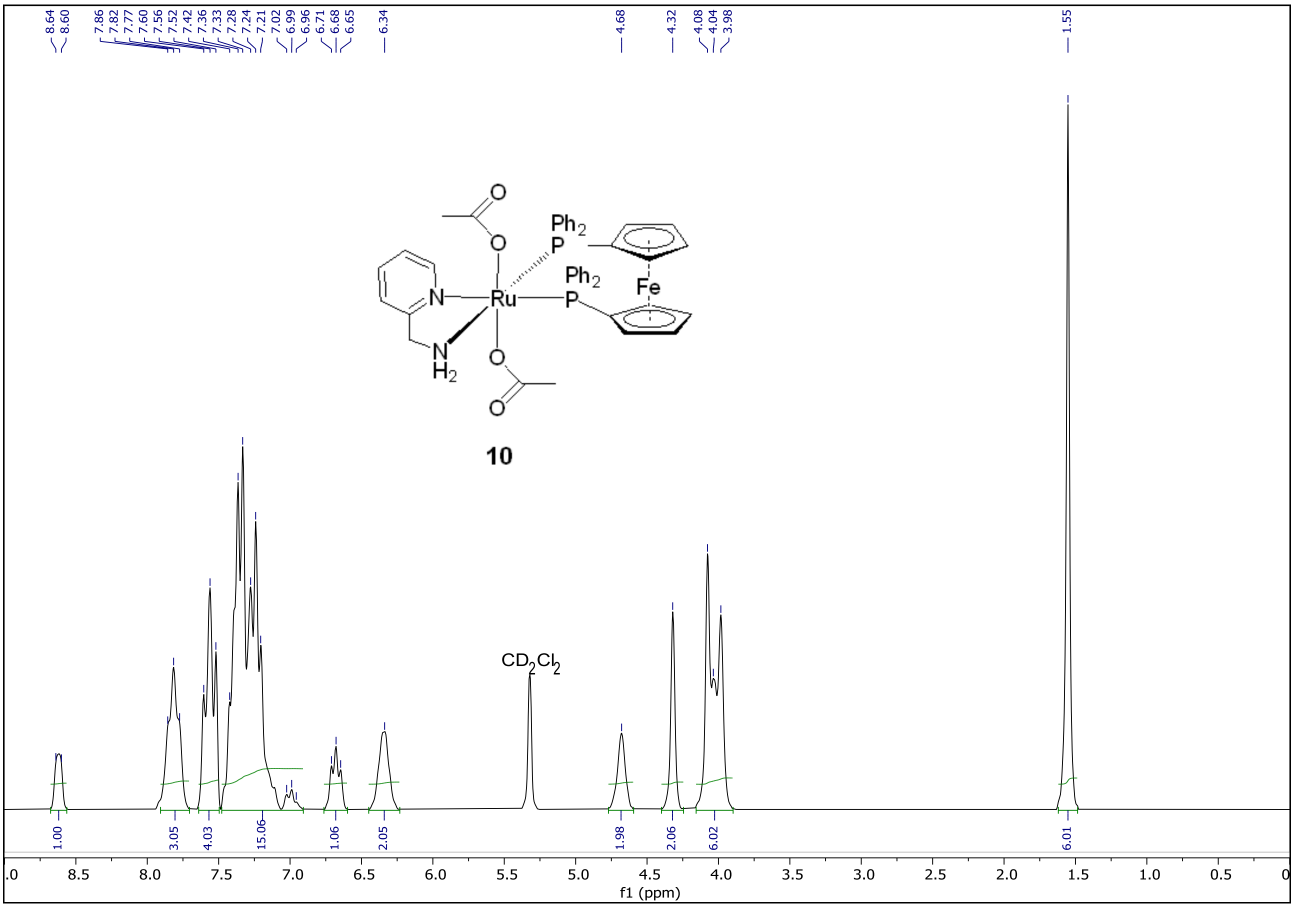

Figure S50. ^{1}H NMR spectrum (200.1 MHz) of *trans*-[Ru(η^1-OAc)$_2$(dppf)(ampy)] (**10**) in CD_2Cl_2 at 20 °C.

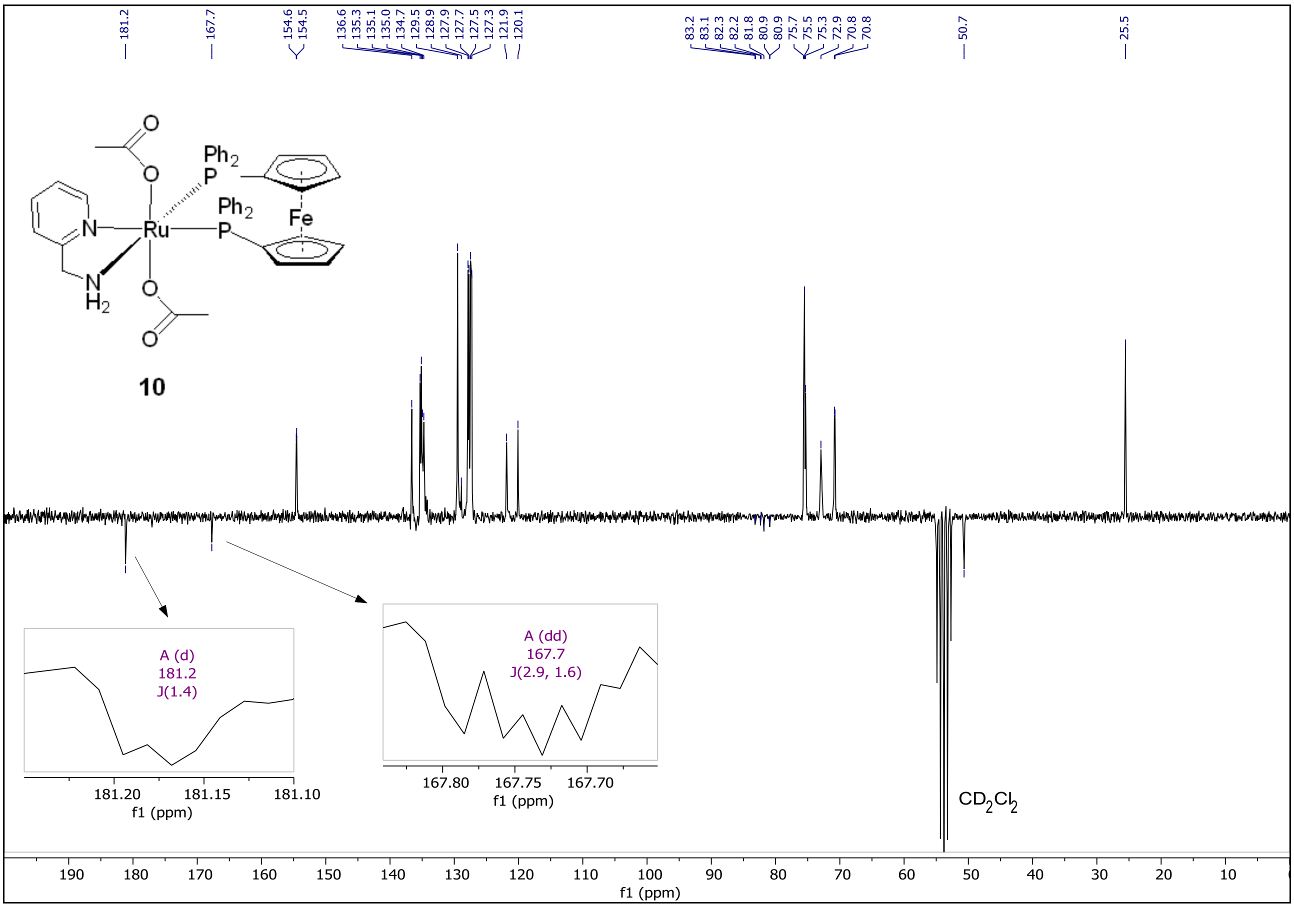

Figure S51. $^{13}C\{^{1}H\}$ PENDANT NMR spectrum (50.3 MHz) of *trans*-[Ru(η^1-OAc)$_2$(dppf)(ampy)] (**10**) in CD_2Cl_2 at 20 °C.

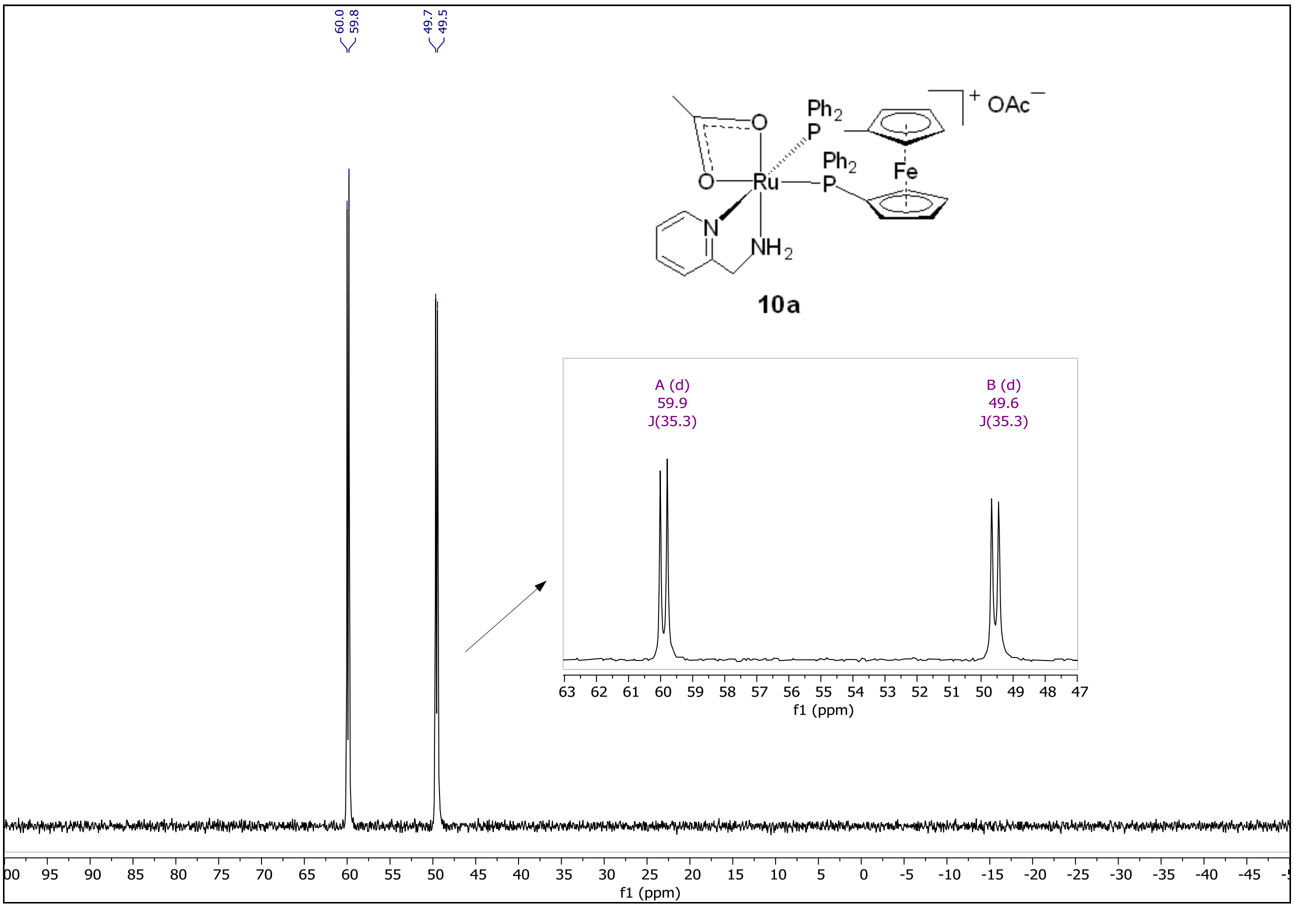

Figure S2. $^{31}P\{^{1}H\}$ NMR spectrum (162.0 MHz) of [Ru(η^2-OAc)(dppf)(ampy)]OAc (**10a**) in CD_3OD at 25 °C.

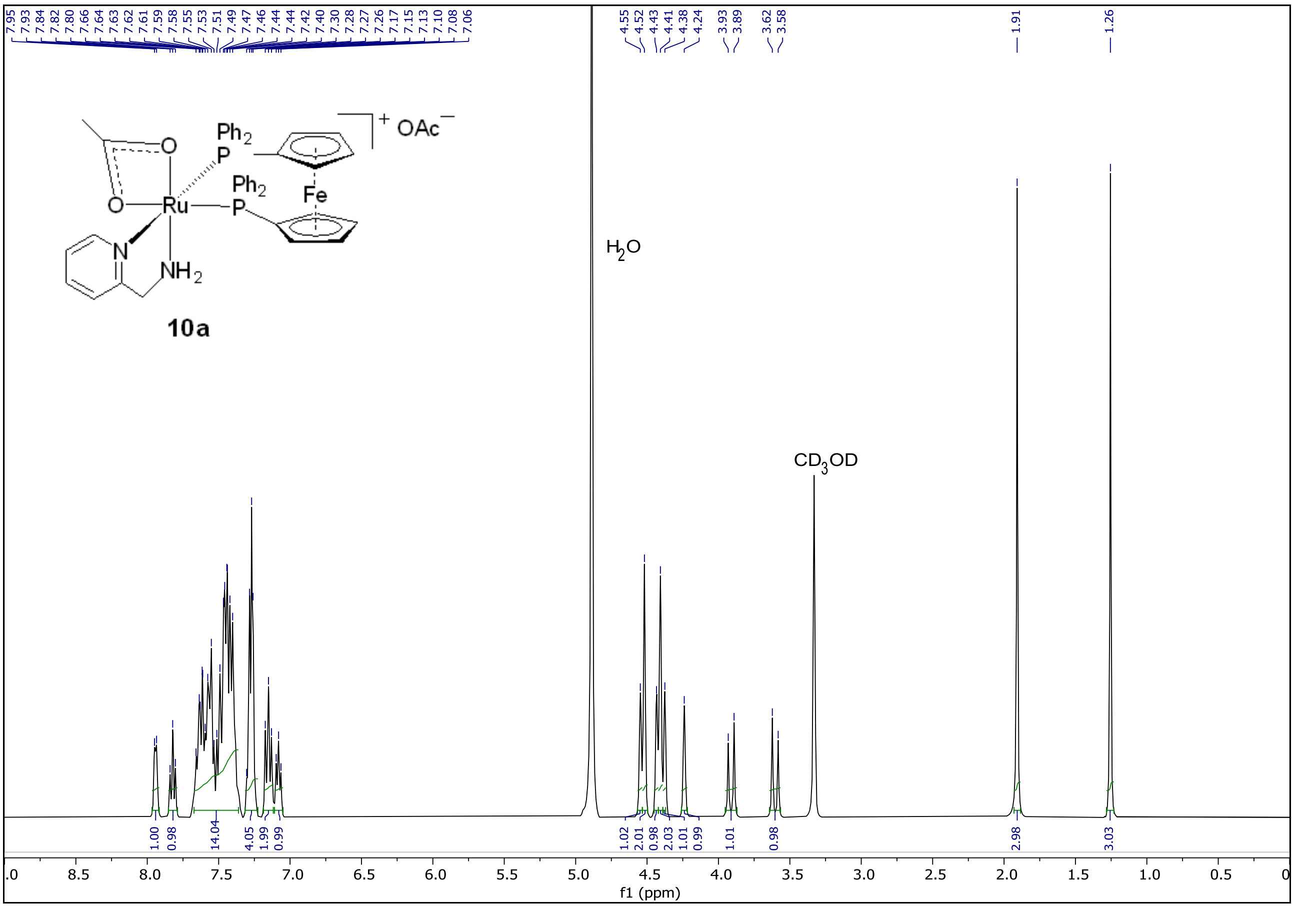

Figure S53. ^{1}H NMR spectrum (400.1 MHz) of [Ru(η^2-OAc)(dppf)(ampy)]OAc (**10a**) in CD_3OD at 25 °C.

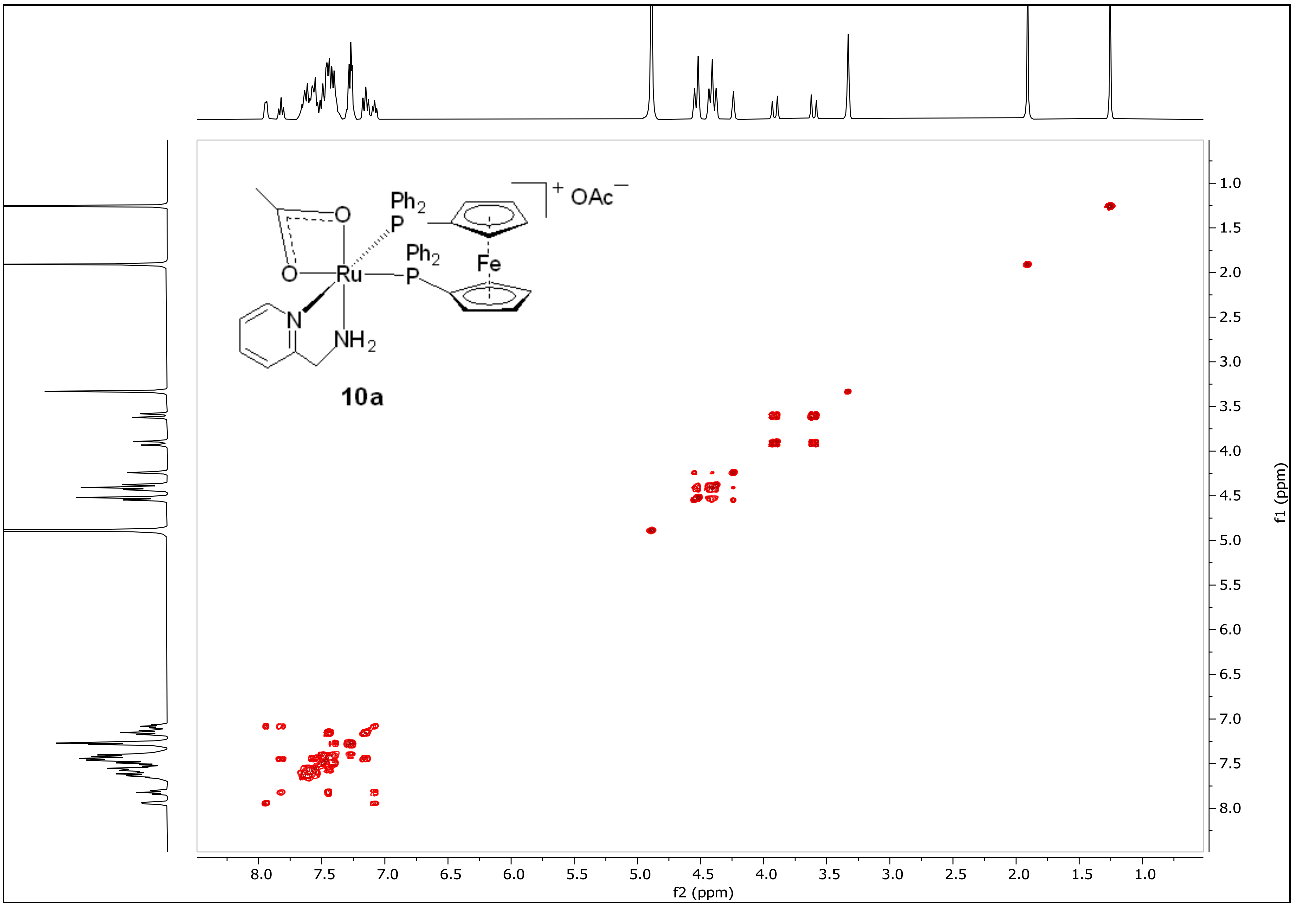

Figure S54. ^{1}H-^{1}H COSY 2D NMR spectrum (400.1 MHz) of [Ru(η^2-OAc)(dppf)(ampy)]OAc (**10a**) in CD_3OD at 25 °C.

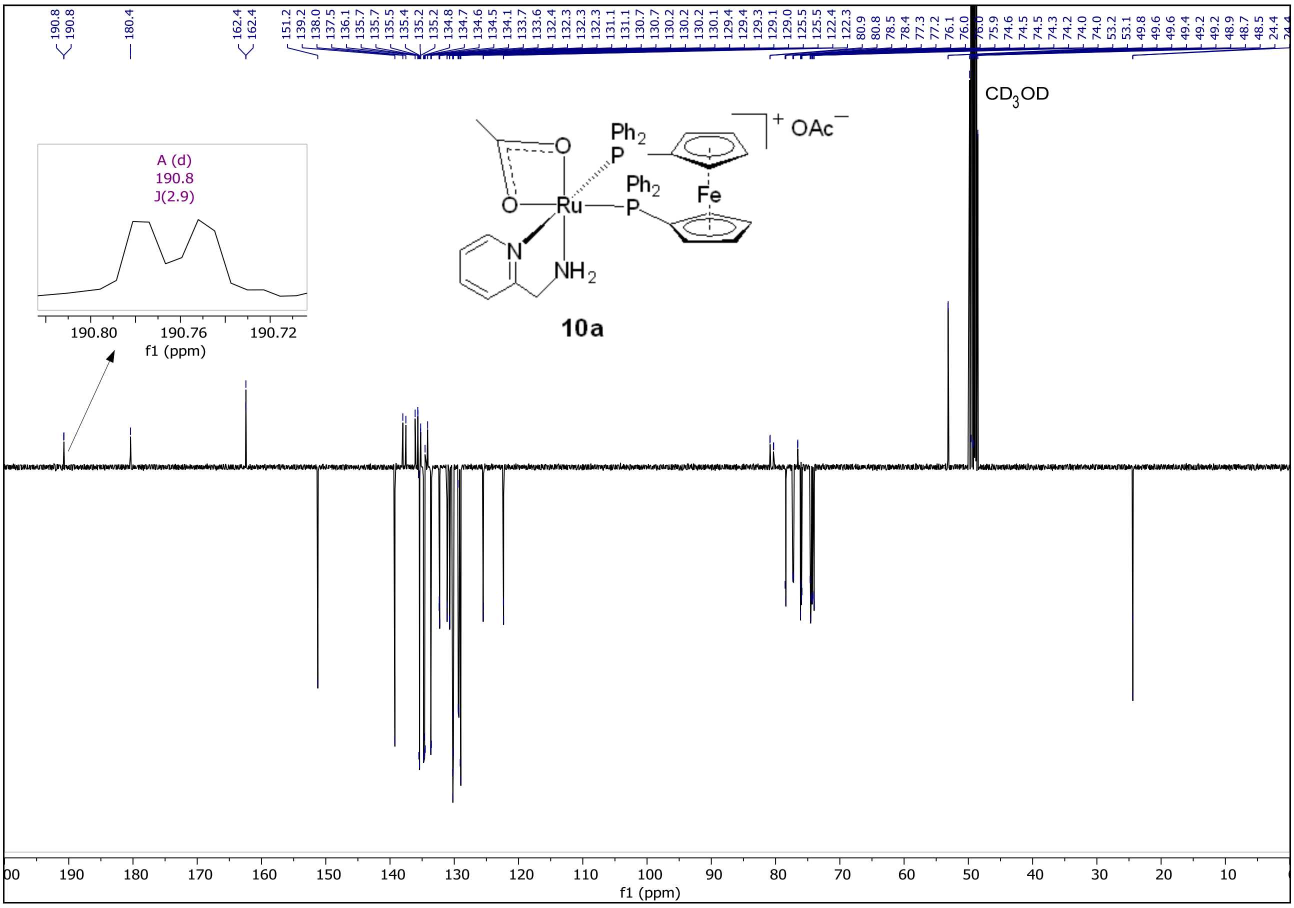

Figure S55. $^{13}C\{^{1}H\}$ DEPTQ NMR spectrum (100.6 MHz) of [Ru(η^2-OAc)(dppf)(ampy)]OAc (**10a**) in CD_3OD at 25 °C.

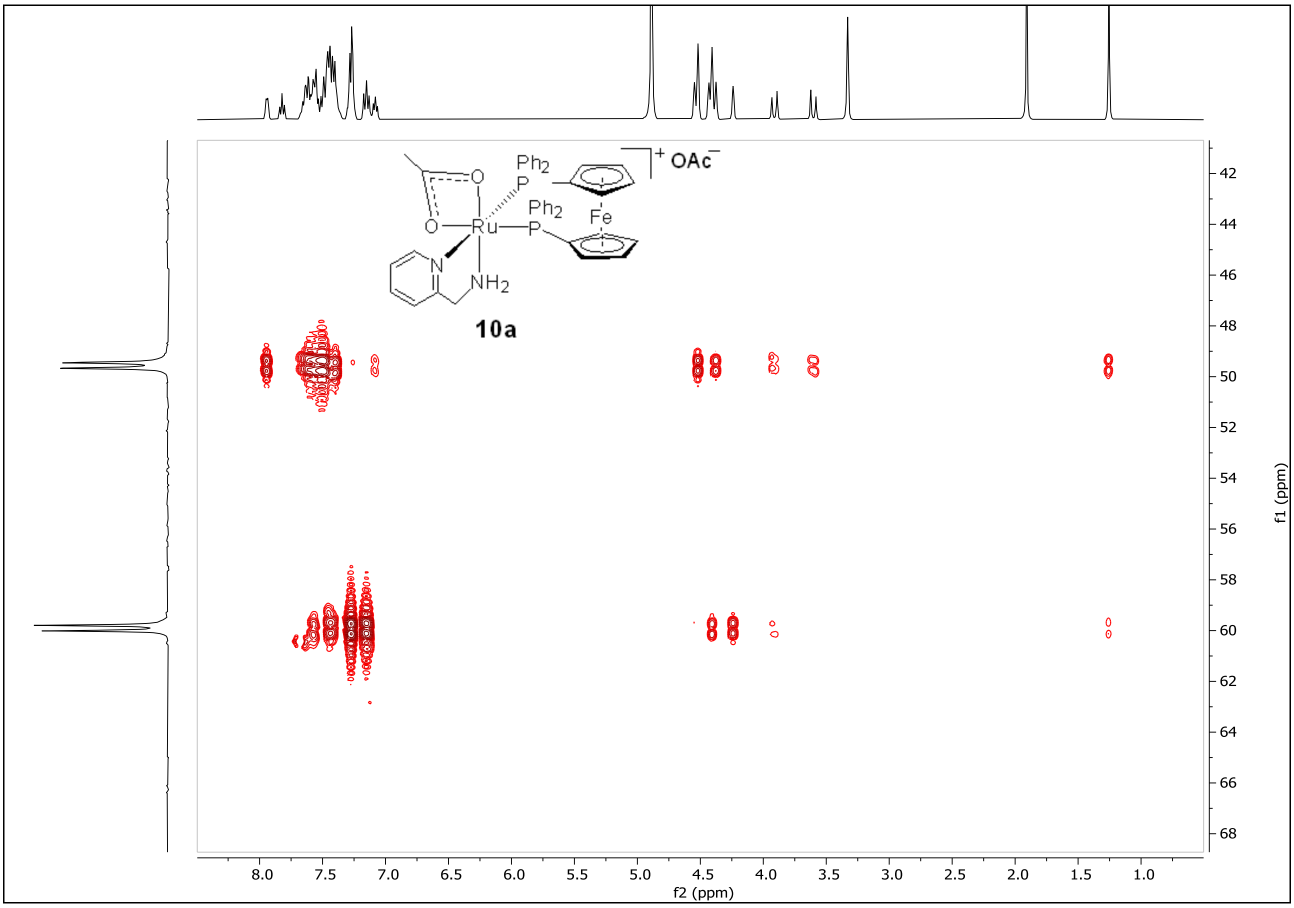

Figure S56. ^{1}H-^{31}P HMBC 2D NMR spectrum of [Ru(η^2-OAc)(dppf)(ampy)]OAc (**10a**) in CD_3OD at 25 °C.

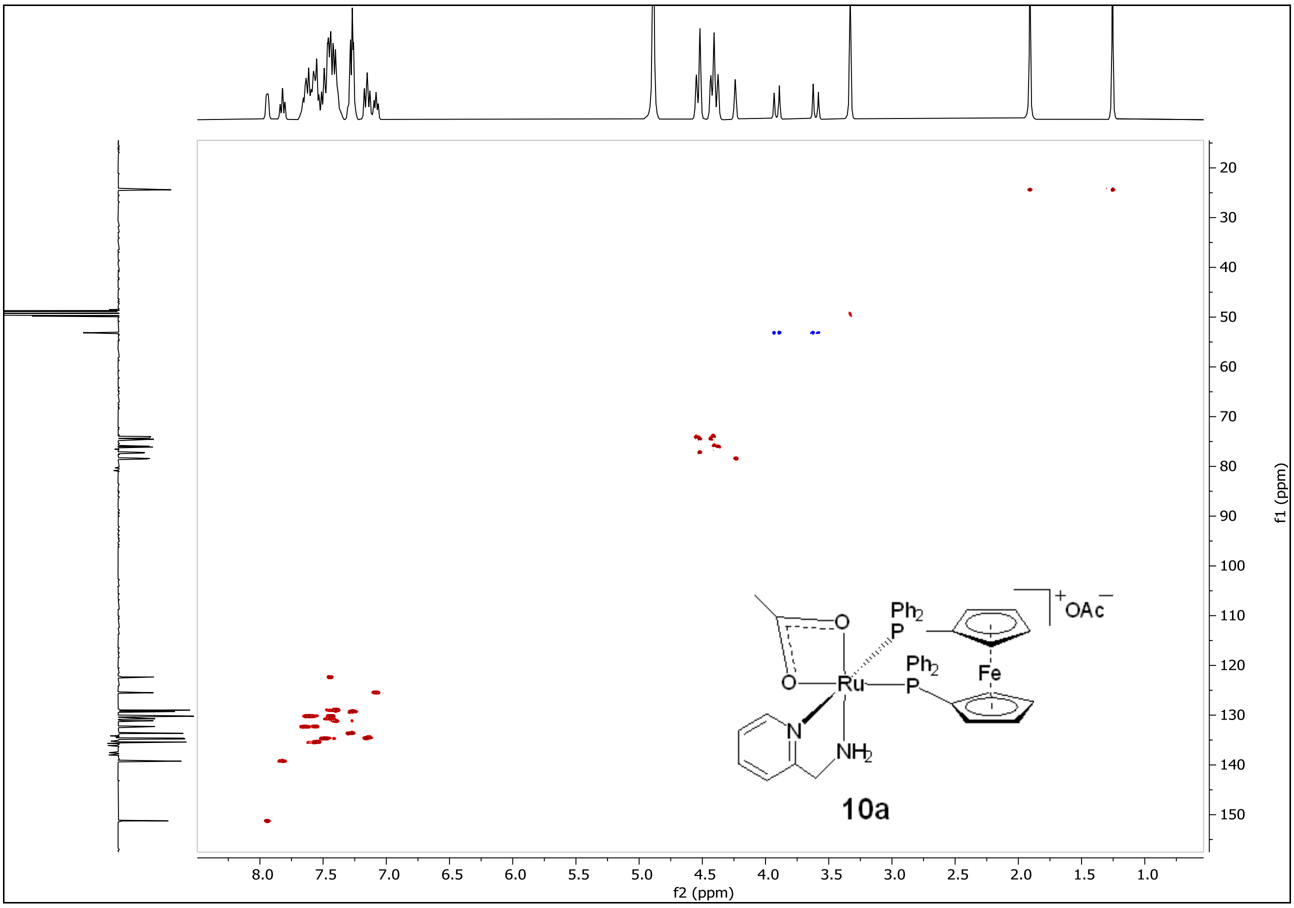

Figure S57. ^{1}H-^{13}C HSQC 2D NMR spectrum of [Ru(η^2-OAc)(dppf)(ampy)]OAc (**10a**) in CD_3OD at 25 °C.

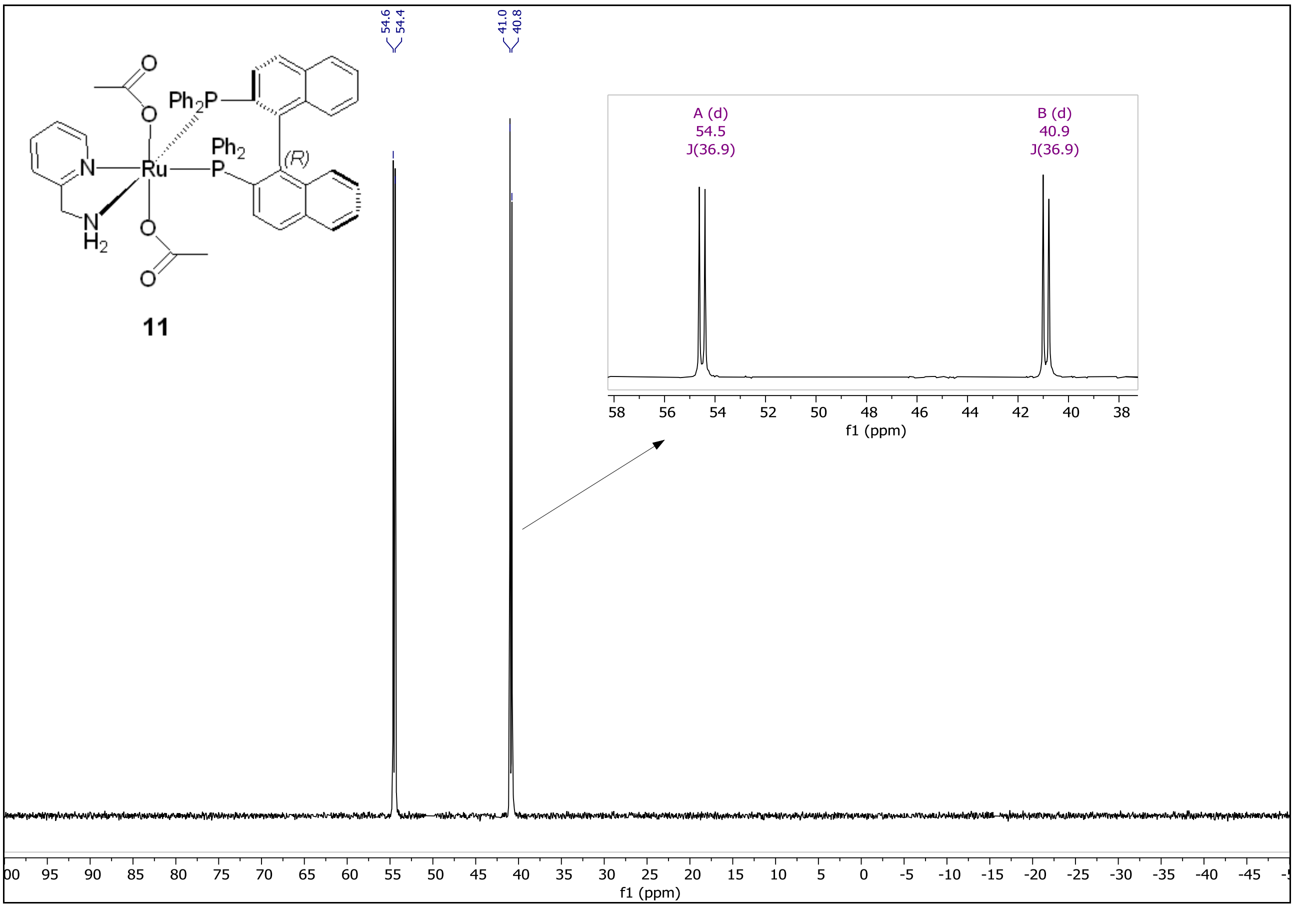

Figure S58. $^{31}P\{^{1}H\}$ NMR spectrum (162.0 MHz) of *trans*-[Ru(η^1-OAc)$_2$((*R*)-BINAP)(ampy)] (**11**) in CD_2Cl_2 at 25 °C.

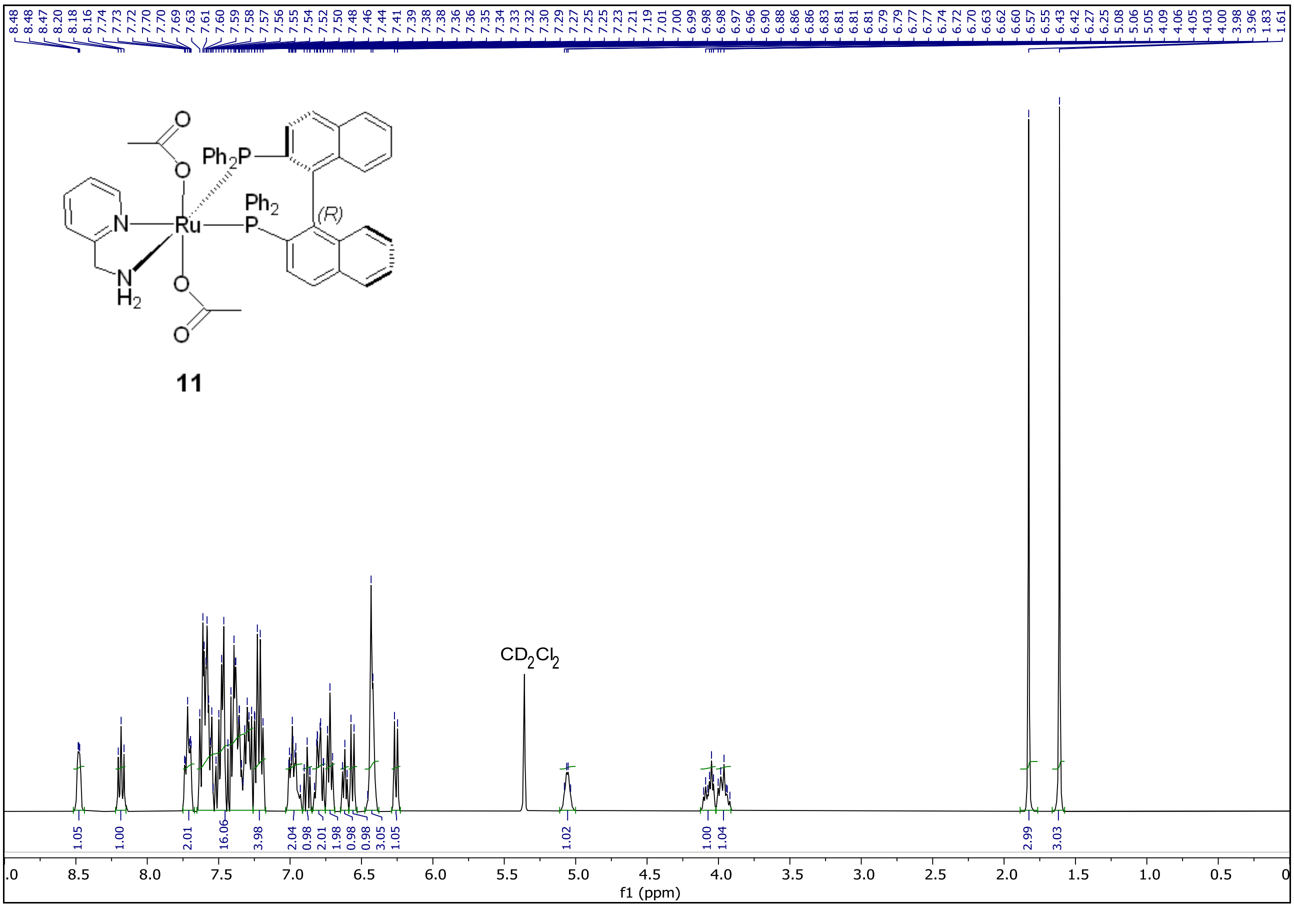

Figure S59. ^{1}H NMR spectrum (400.1 MHz) of *trans*-[Ru(η^{1}-OAc)$_2$((*R*)-BINAP)(ampy)] (**11**) in CD_2Cl_2 at 25 °C.

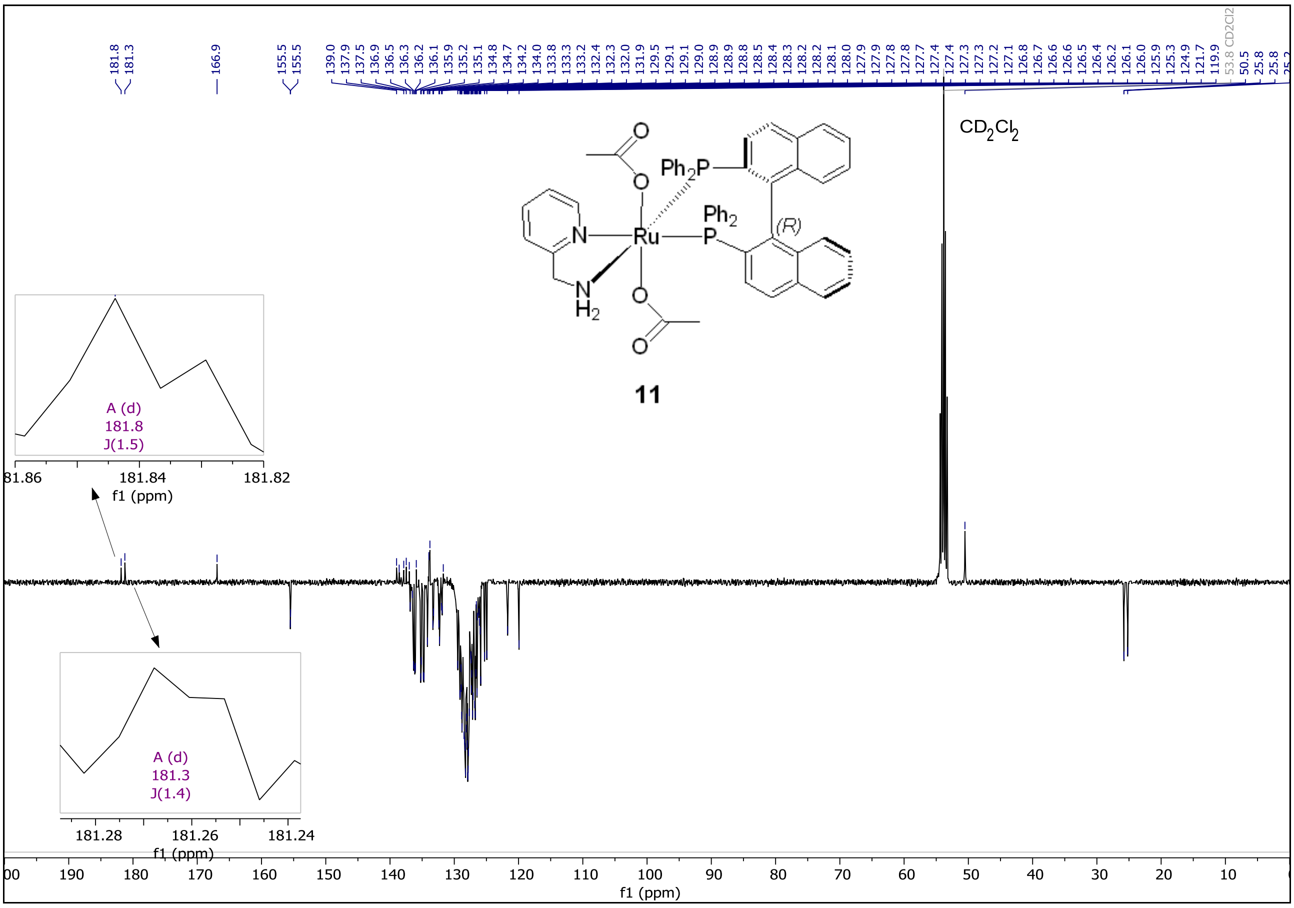

Figure S60. $^{13}C\{^{1}H\}$ DEPTQ NMR spectrum (100.6 MHz) of *trans*-[Ru(η^1-OAc)$_2$((*R*)-BINAP)(ampy)] (**11**) in CD_2Cl_2 at 25 °C.

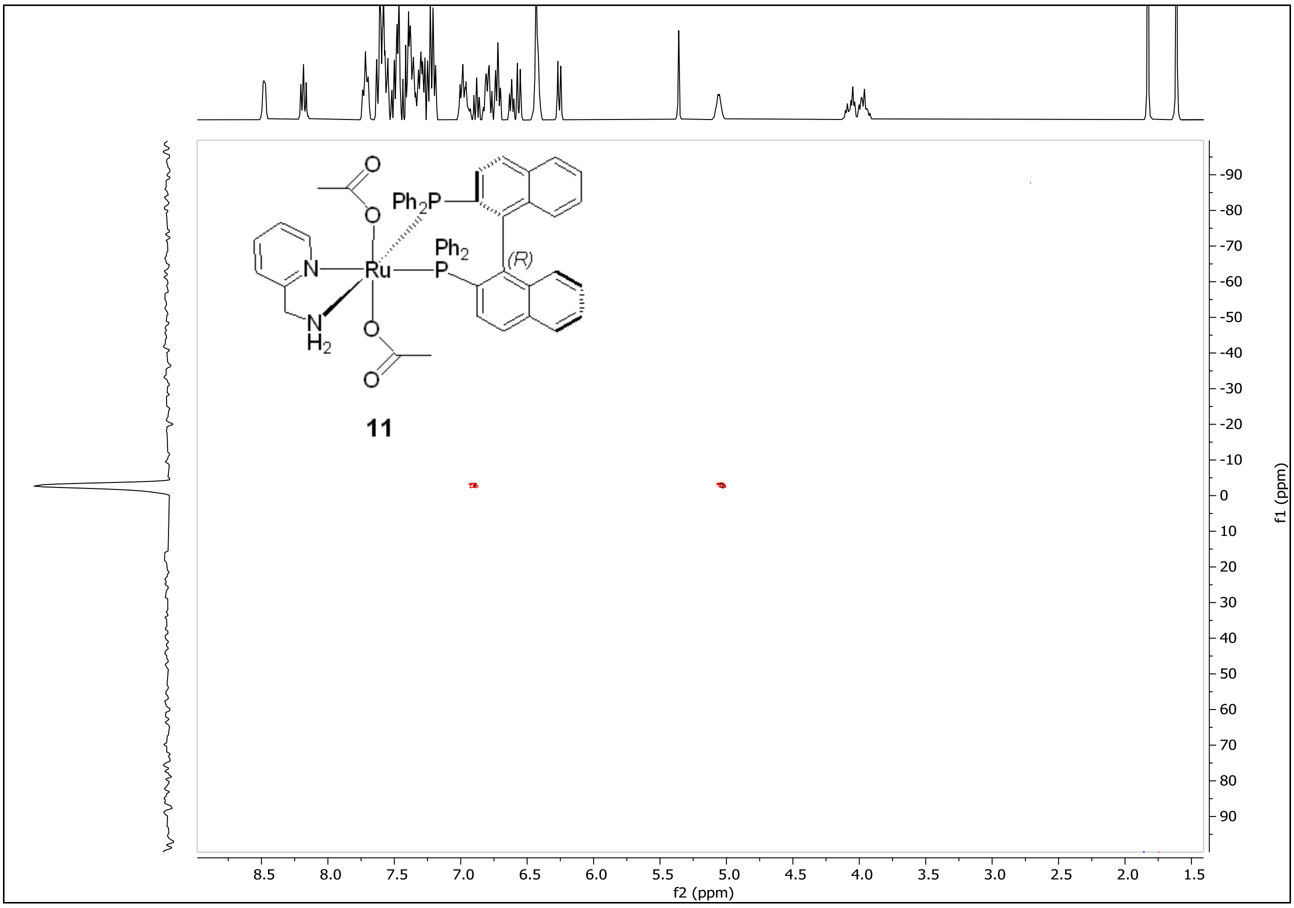

Figure S61. ^{1}H-^{15}N HSQC 2D NMR spectrum of *trans*-[Ru(η^1-OAc)$_2$((*R*)-BINAP)(ampy)] (**11**) in CD_2Cl_2 at 25 °C.

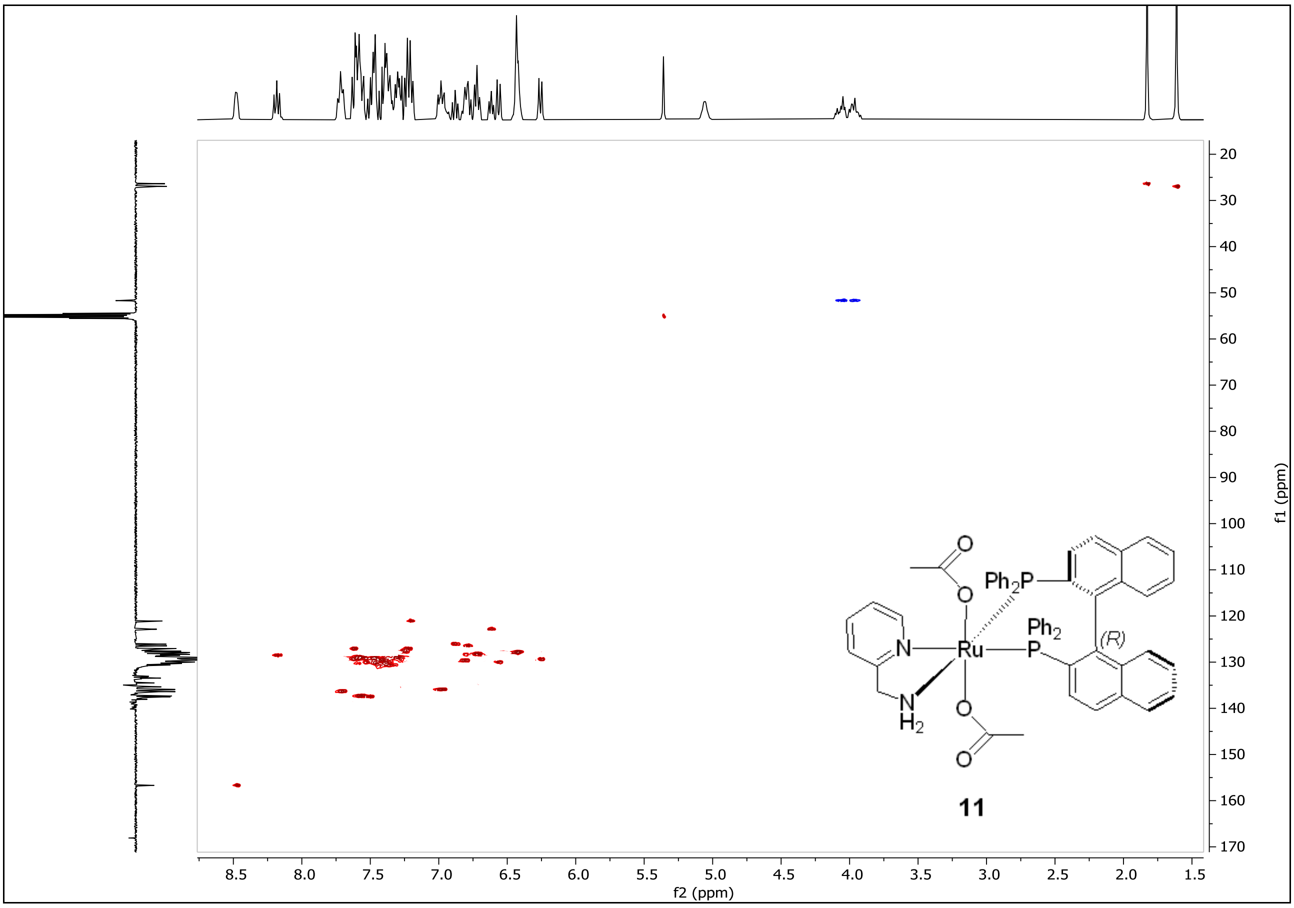

Figure S62. ^{1}H-^{13}C HSQC 2D NMR spectrum of *trans*-[Ru(η^{1}-OAc)$_2$((*R*)-BINAP)(ampy)] (**11**) in CD_2Cl_2 at 25 °C.

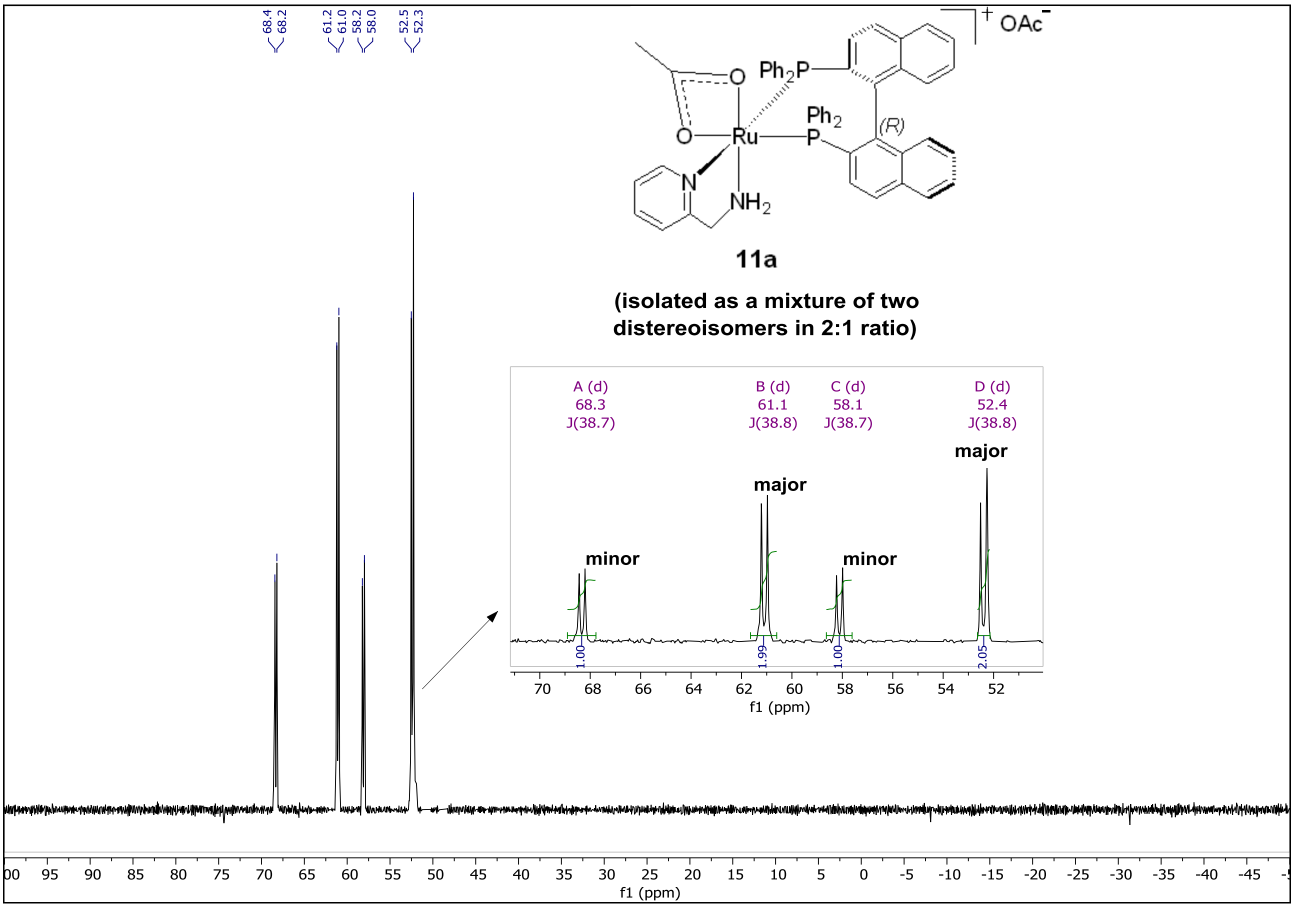

Figure S63. $^{31}P\{^{1}H\}$ NMR spectrum (162.0 MHz) of [Ru(η^2-OAc)((*R*)-BINAP)(ampy)]OAc (**11a**) in CD_3OD at 25 °C.

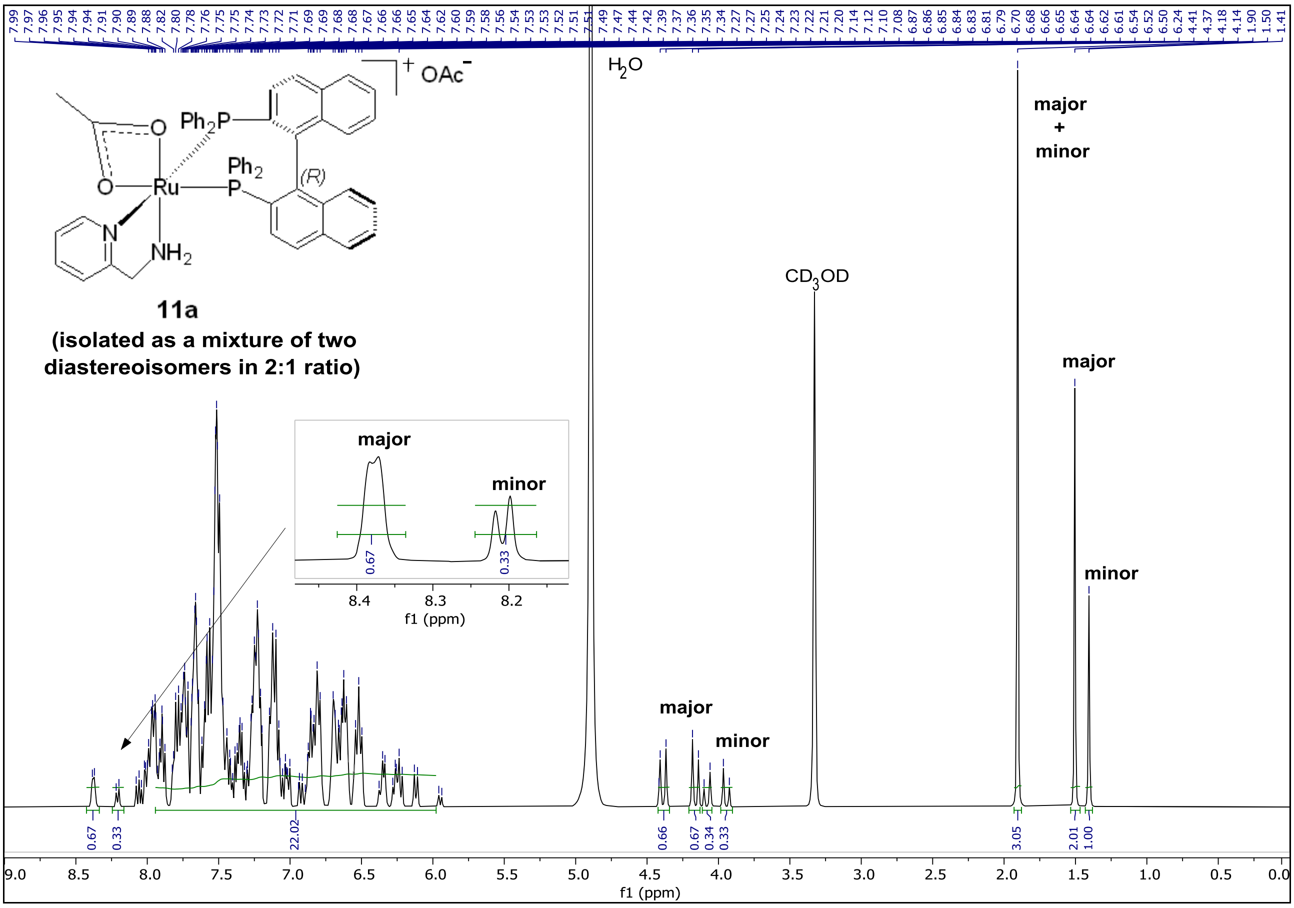

Figure S64. ^{1}H NMR spectrum (400.1 MHz) of [Ru(η^2-OAc)((*R*)-BINAP)(ampy)]OAc (**11a**) in CD_3OD at 25 °C.

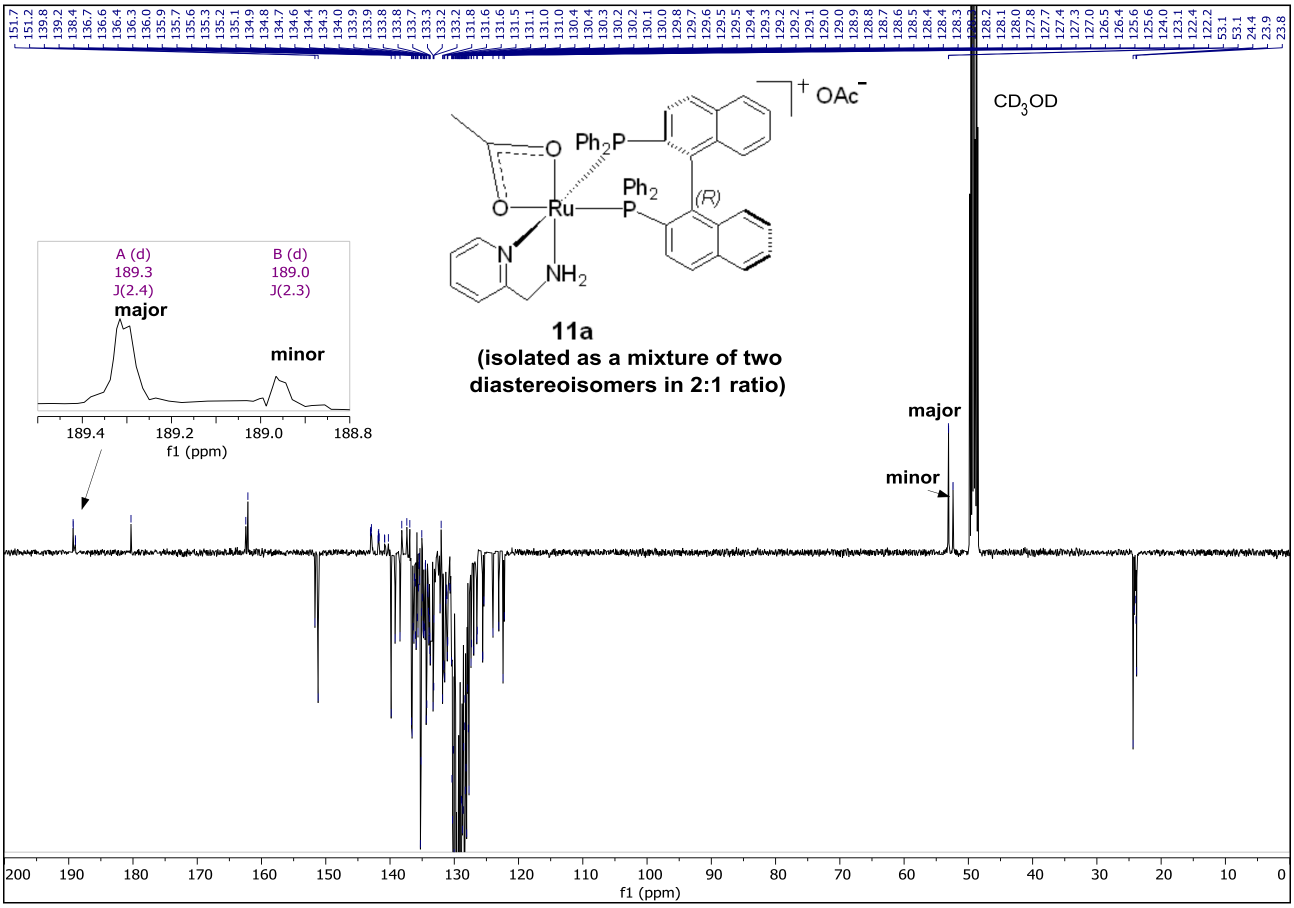

Figure S65. $^{13}C\{^1H\}$ DEPTQ NMR spectrum (100.6 MHz) of [Ru(η^2-OAc)((*R*)-BINAP)(ampy)]OAc (**11a**) in CD_3OD at 25 °C.

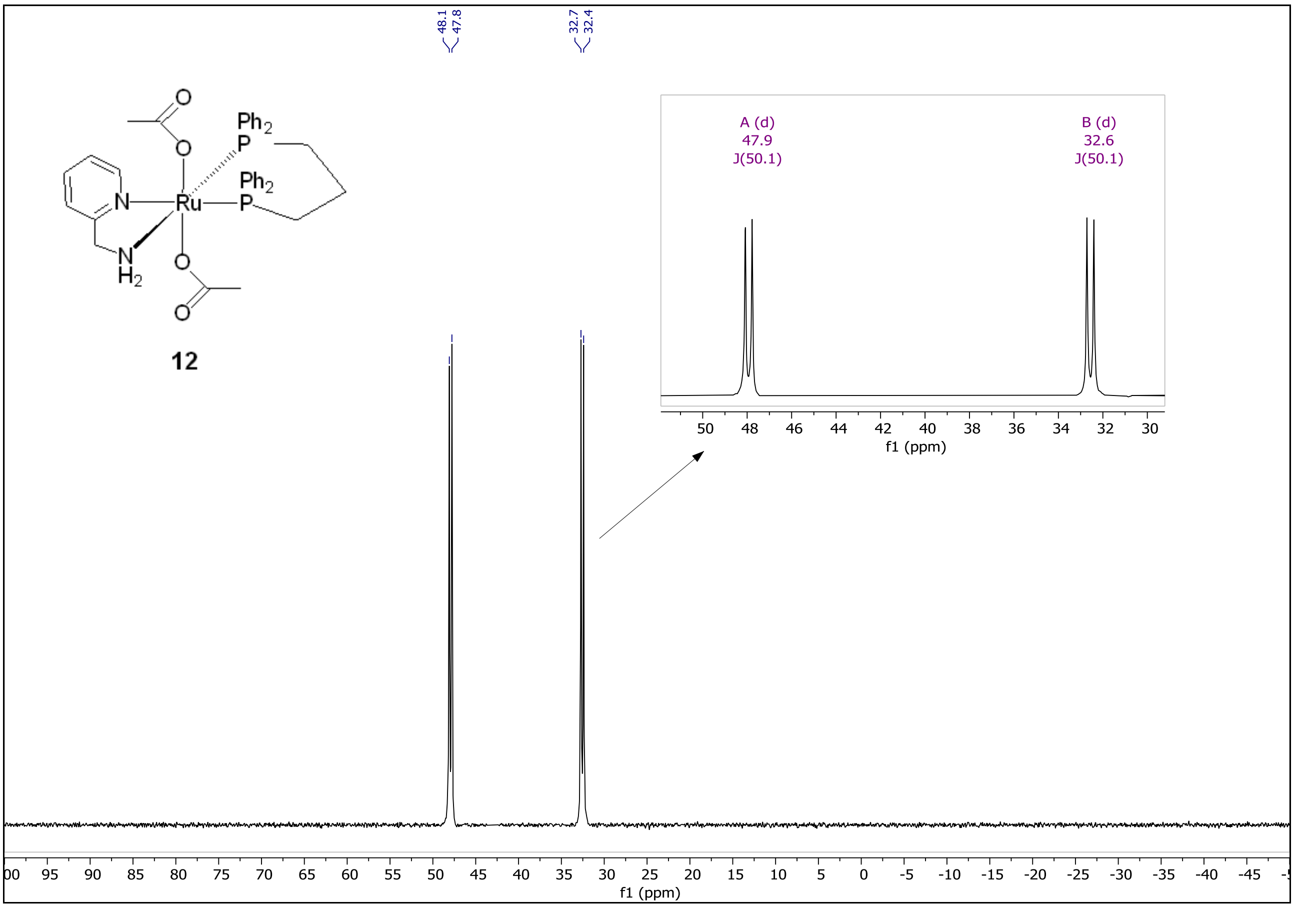

Figure S66. $^{31}P\{^{1}H\}$ NMR spectrum (162.0 MHz) of *trans*-[Ru(η^{1}-OAc)$_2$(dppp)(ampyrim)] (**12**) in CD_2Cl_2 at 25 °C.

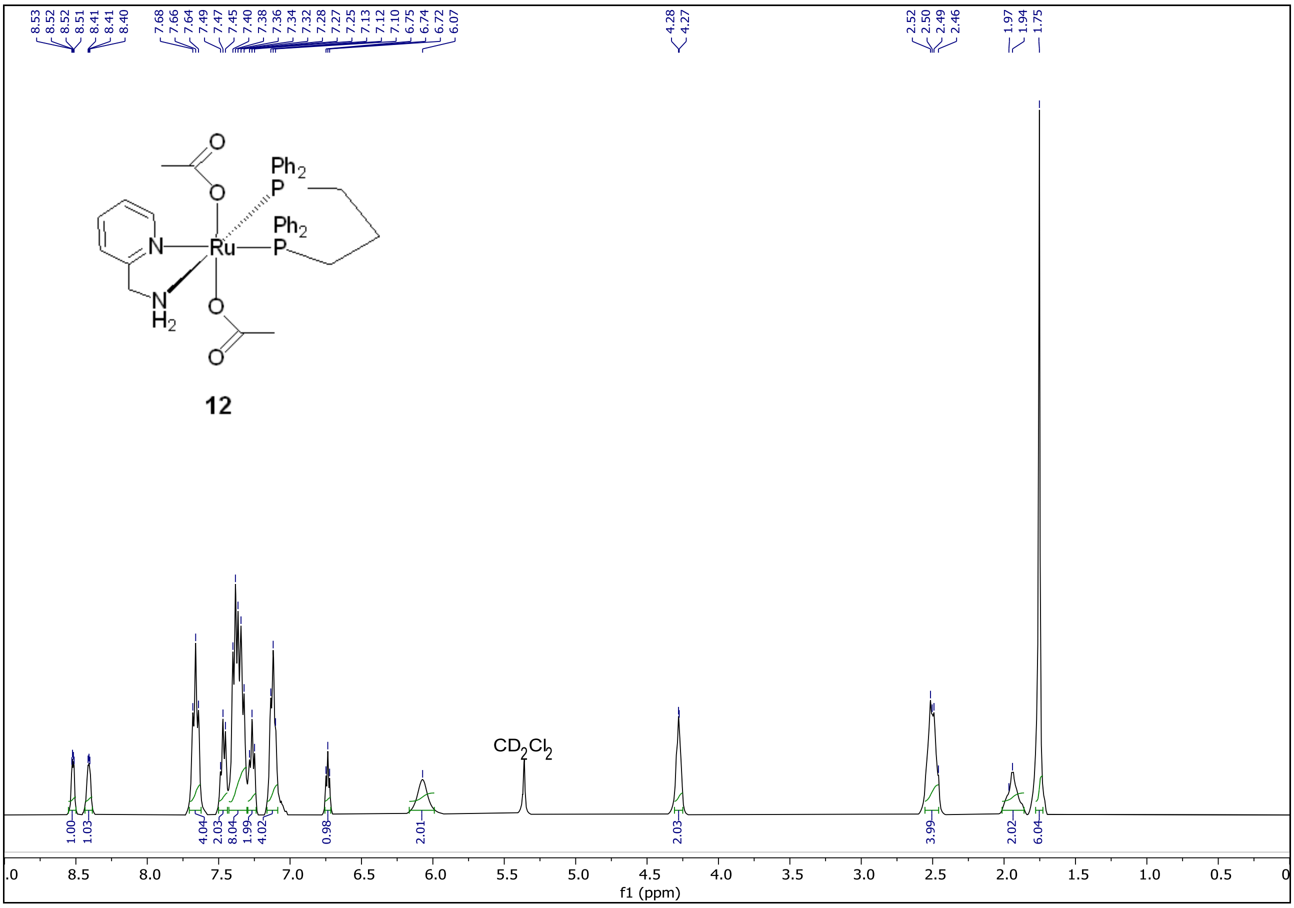

Figure S67. ^{1}H NMR spectrum (400.1 MHz) of *trans*-[Ru(η^1-OAc)$_2$(dppp)(ampyrim)] (**12**) in CD_2Cl_2 at 25 °C.

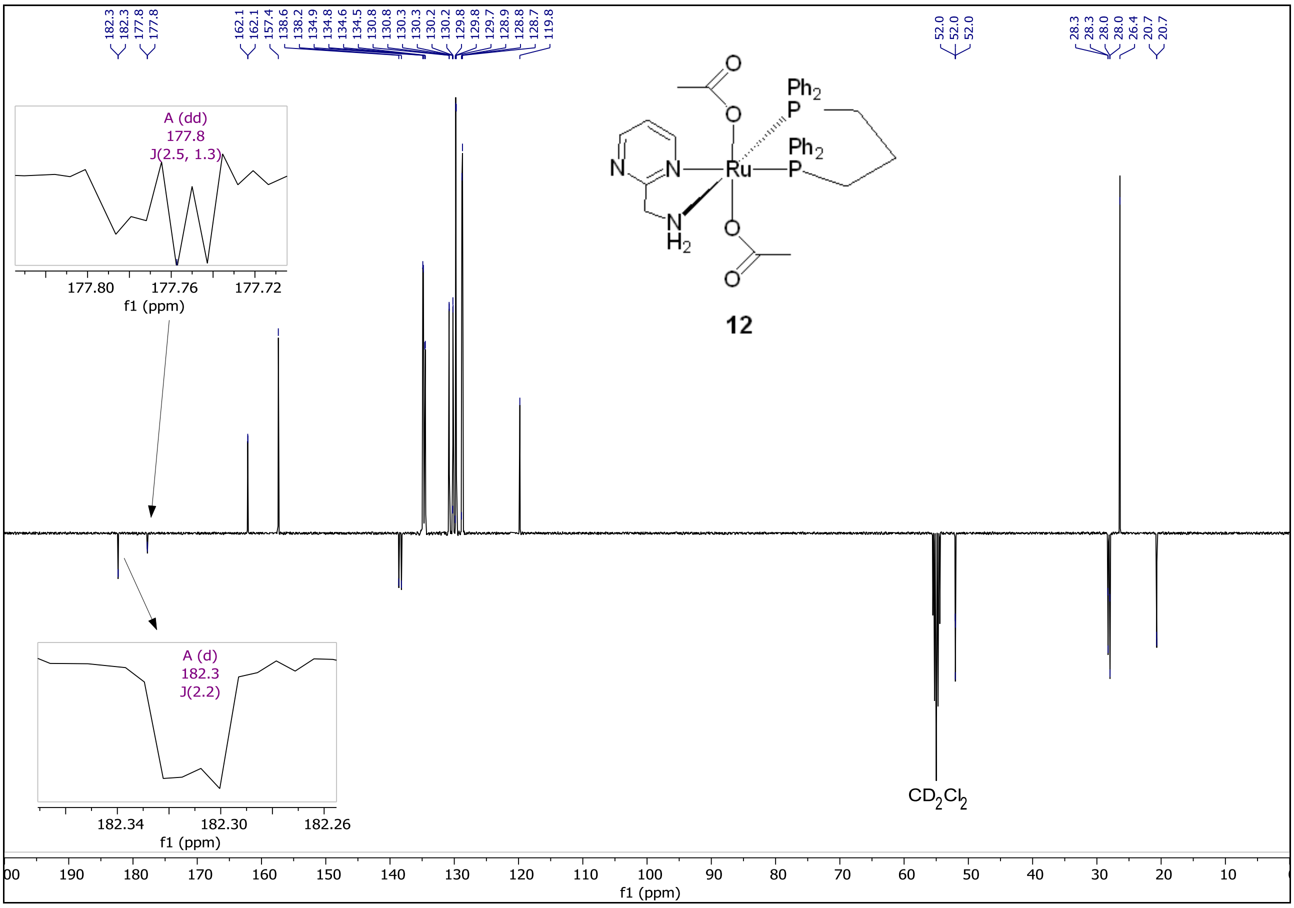

Figure S68. $^{13}C\{^1H\}$ DEPTQ NMR spectrum (100.6 MHz) of *trans*-[Ru(η^1-OAc)$_2$(dppp)(ampyrim)] (**12**) in CD_2Cl_2 at 25 °C.

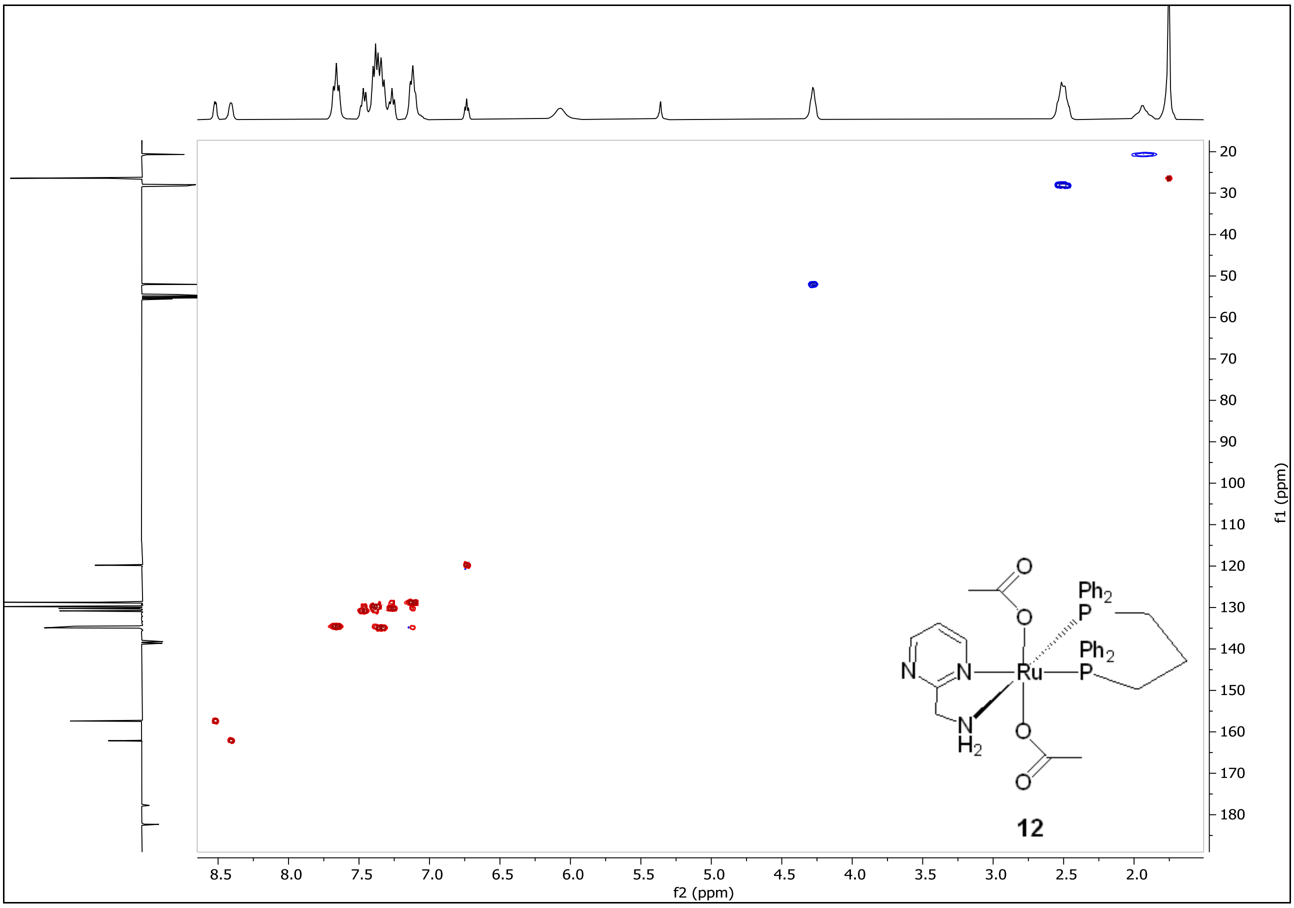

Figure S69. ^{1}H-^{13}C HSQC 2D NMR spectrum of *trans*-[Ru(η^1-OAc)$_2$(dppp)(ampyrim)] (**12**) in CD_2Cl_2 at 25 °C.

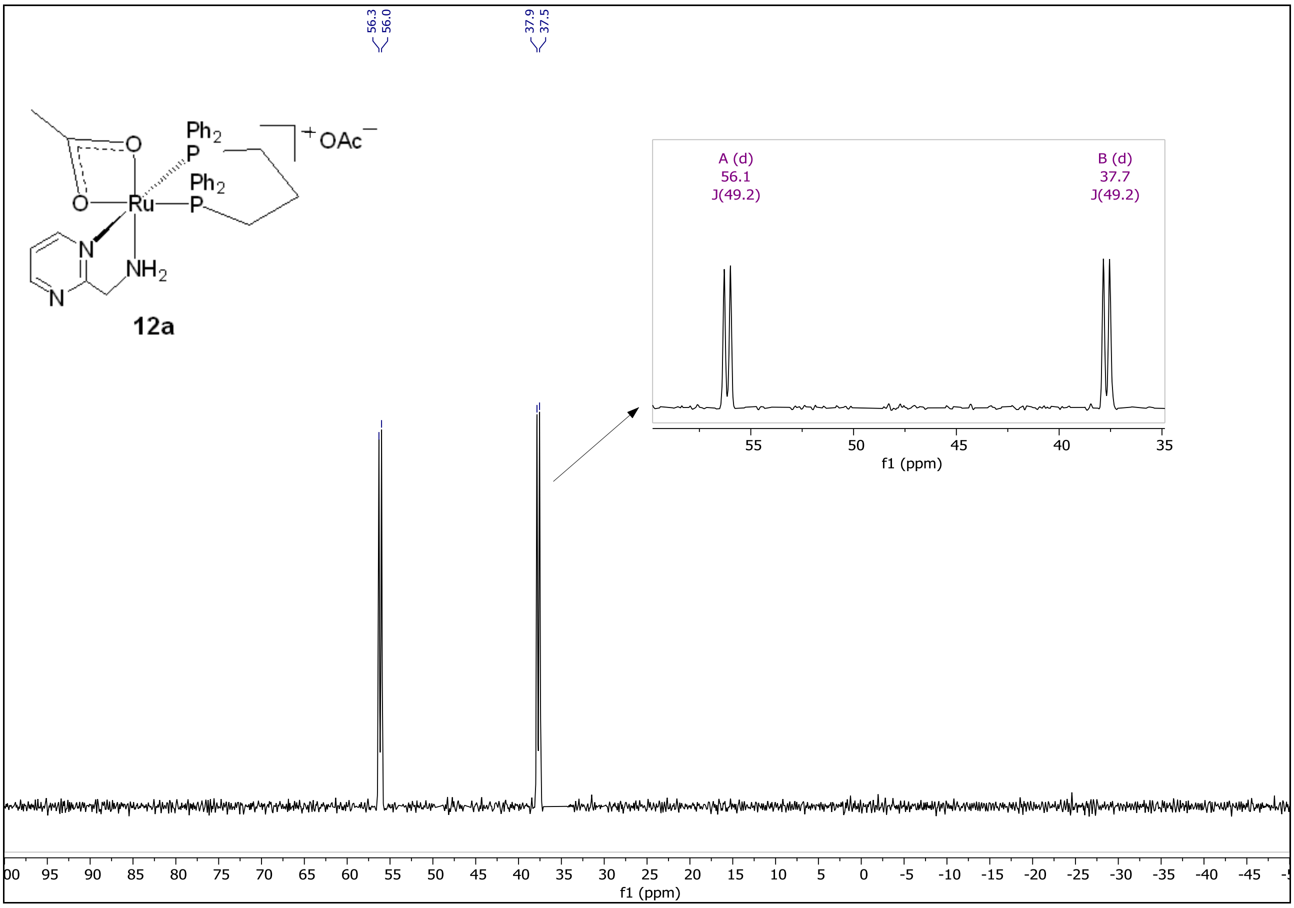

Figure S70. $^{31}P\{^{1}H\}$ NMR spectrum (162.0 MHz) of [Ru(η^2-OAc)(dppp)(ampyrim)]OAc (**12a**) in CD_3OD at 25 °C.

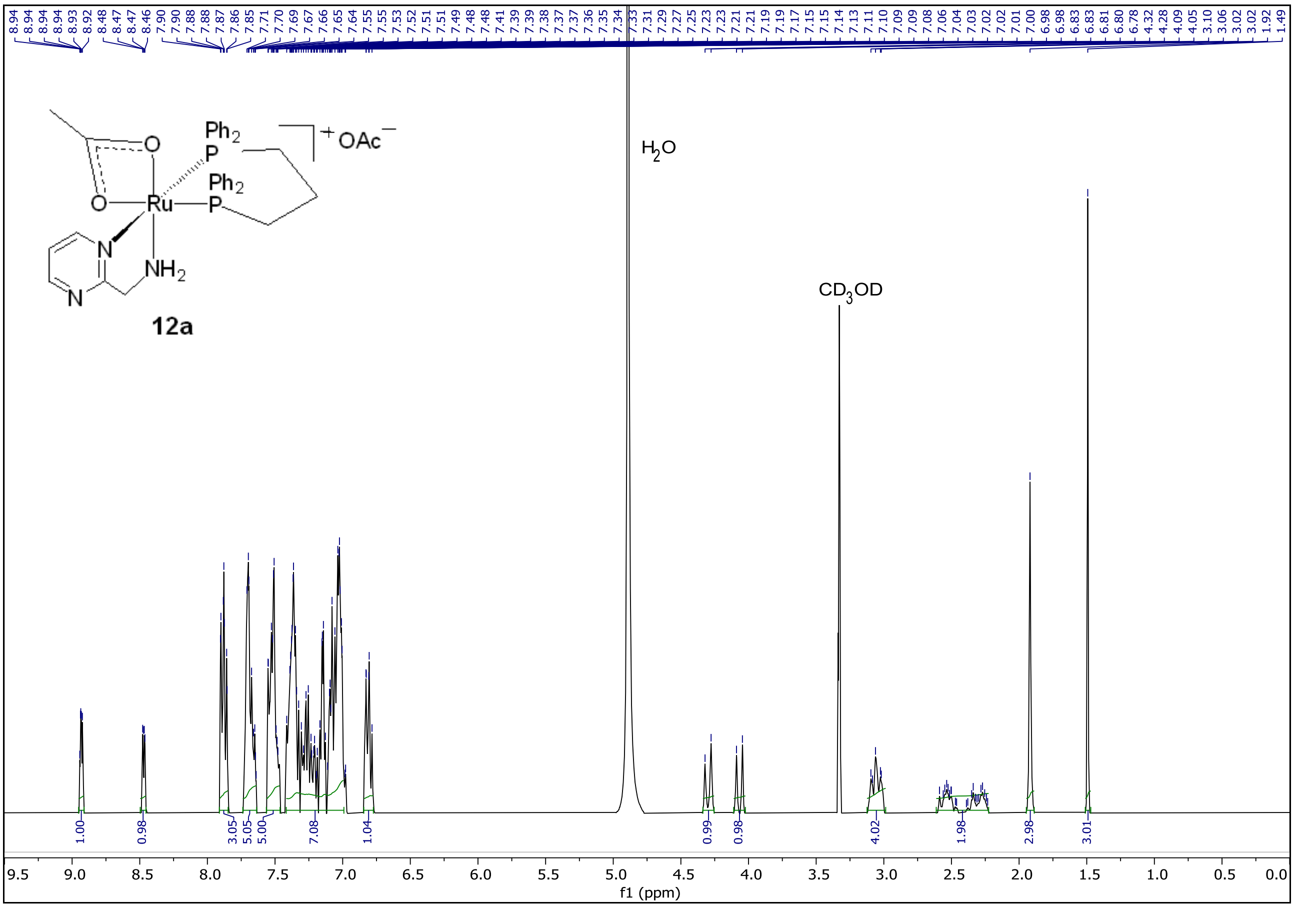

Figure S71. ^{1}H NMR spectrum (400.1 MHz) of [Ru(η^2-OAc)(dppp)(ampyrim)]OAc (**12a**) in CD_3OD at 25 °C.

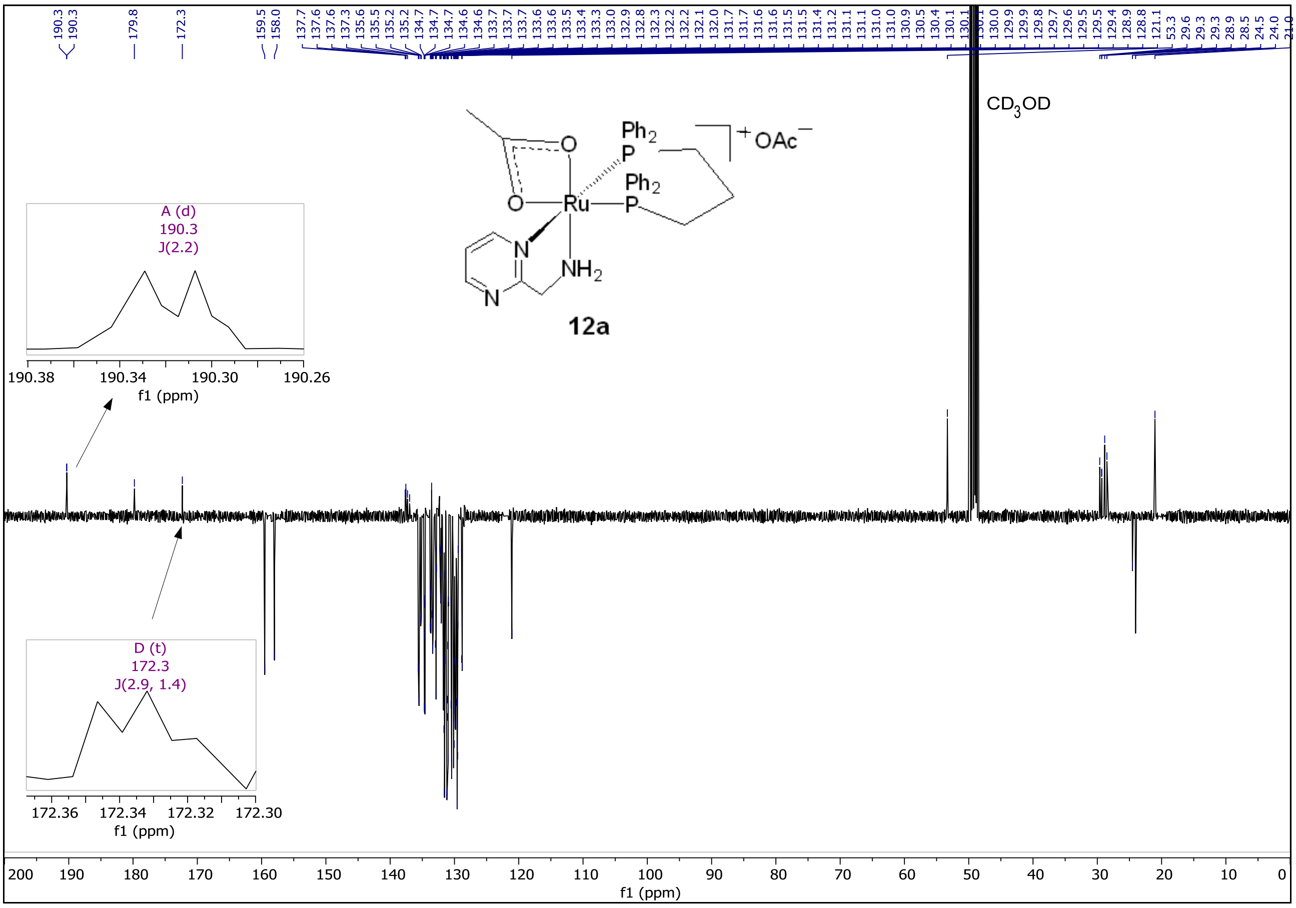

Figure S72. $^{13}C\{^1H\}$ DEPTQ NMR spectrum (100.6 MHz) of [Ru(η^2-OAc)(dppp)(ampyrim)]OAc (**12a**) in CD_3OD at 25 °C.

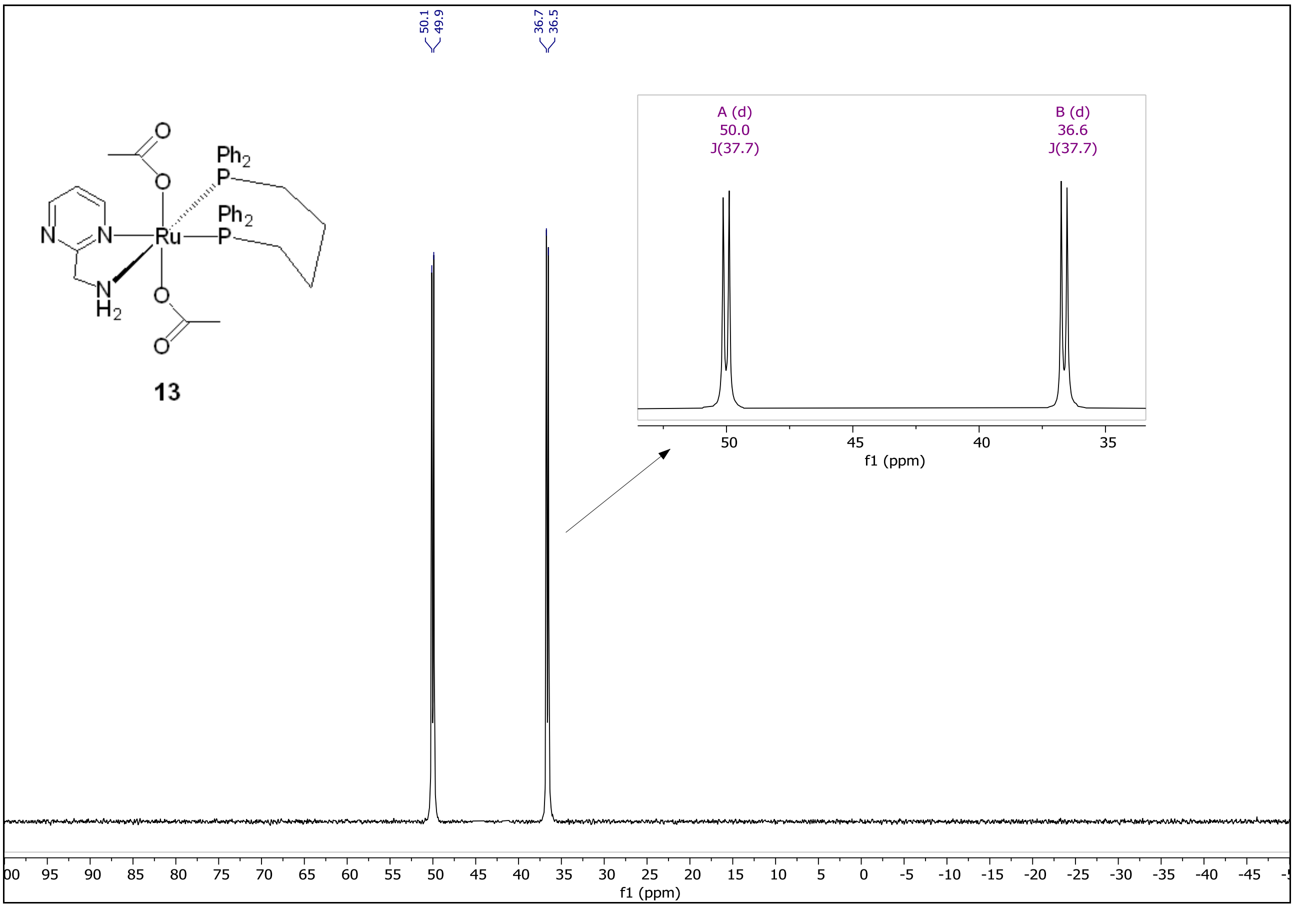

Figure S73. $^{31}P\{^{1}H\}$ NMR spectrum (162.0 MHz) of *trans*-[Ru(η^1-OAc)$_2$(dppb)(ampyrim)] (**13**) in CD_2Cl_2 at 25 °C.

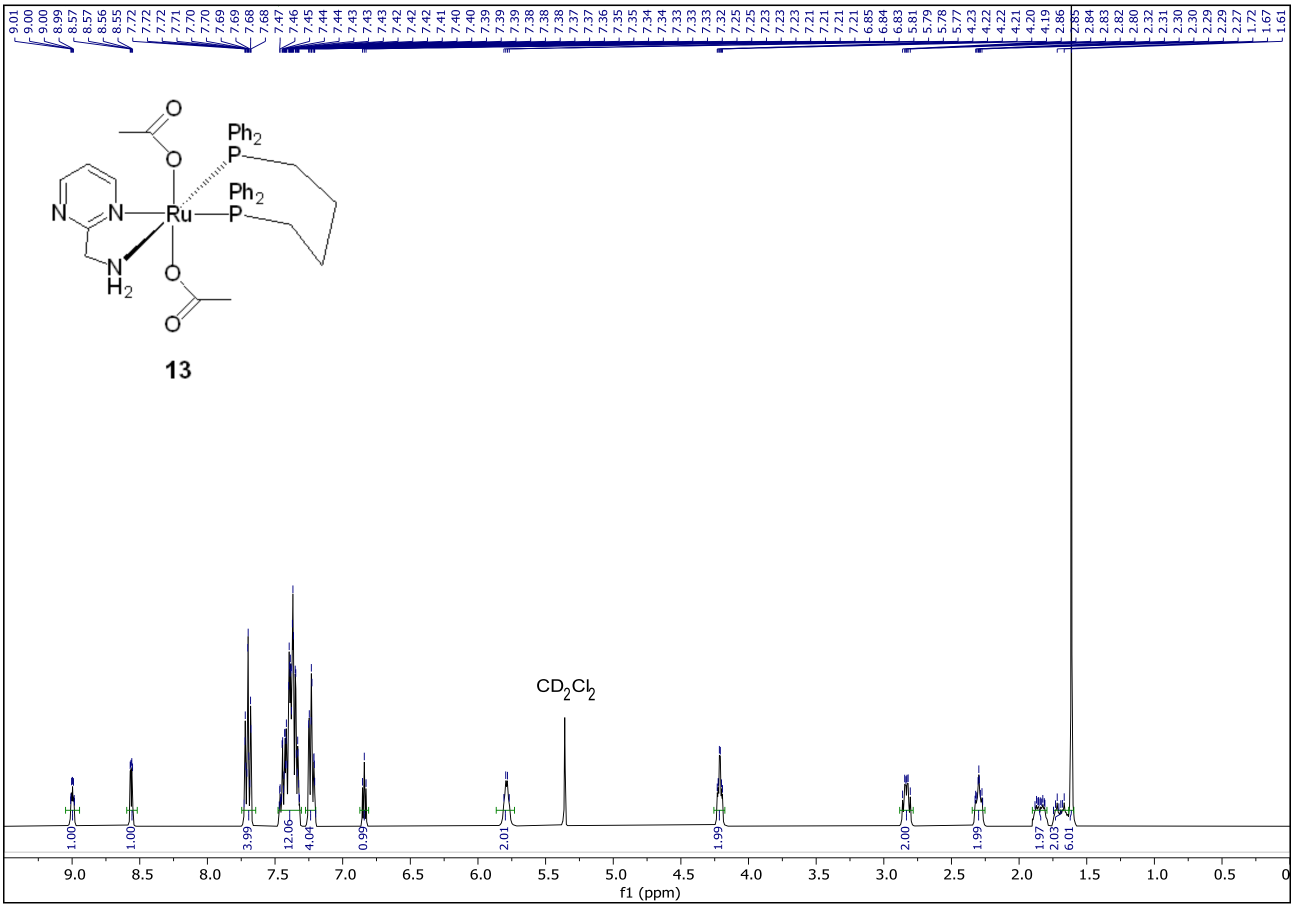

Figure S74. ^{1}H NMR spectrum (400.1 MHz) of *trans*-[Ru(η^1-OAc)$_2$(dppb)(ampyrim)] (**13**) in CD_2Cl_2 at 25 °C.

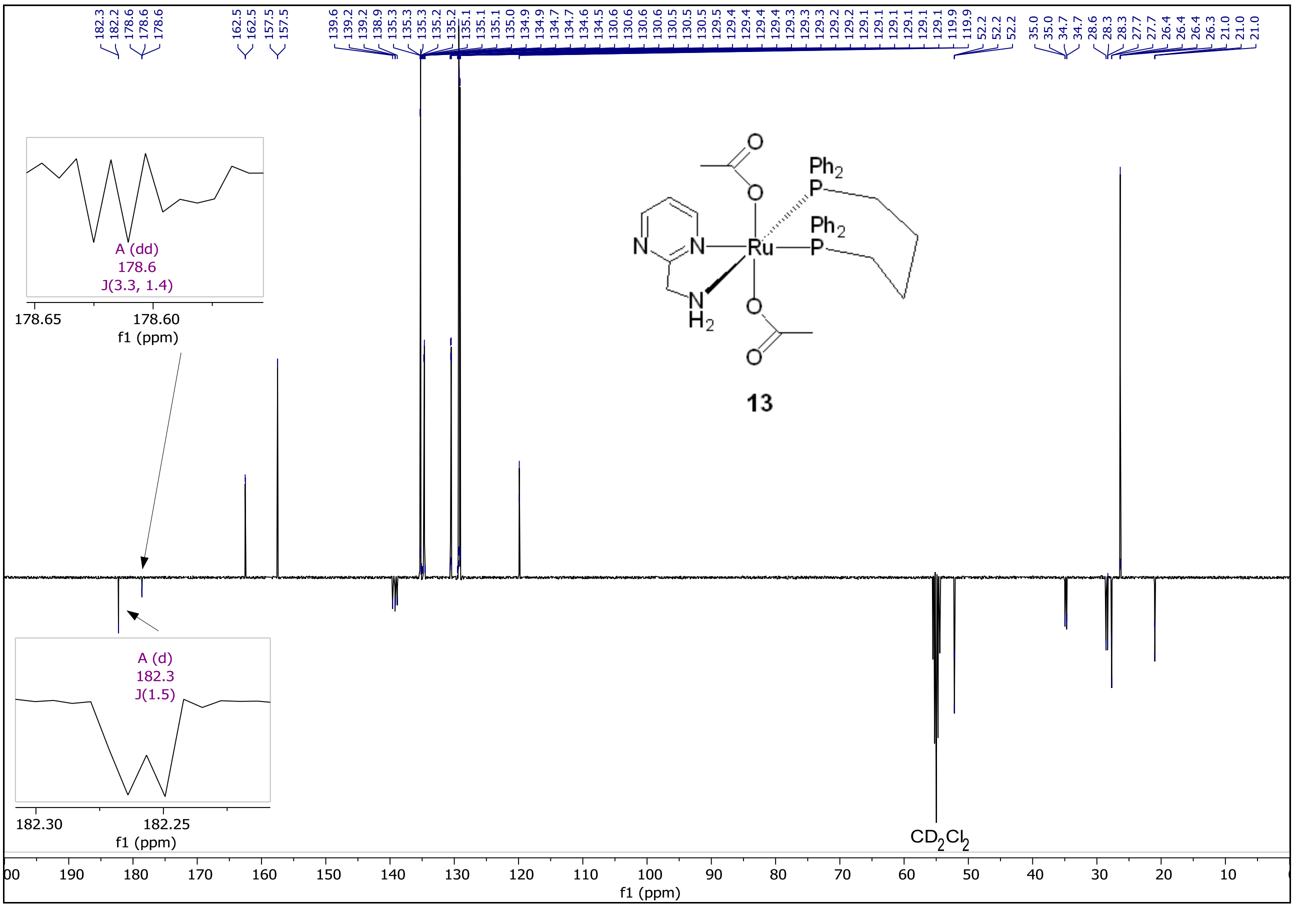

Figure S75. $^{13}C\{^1H\}$ DEPTQ NMR spectrum (100.6 MHz) of *trans*-[Ru(η^1-OAc)$_2$(dppb)(ampyrim)] (**13**) in CD_2Cl_2 at 25 °C.

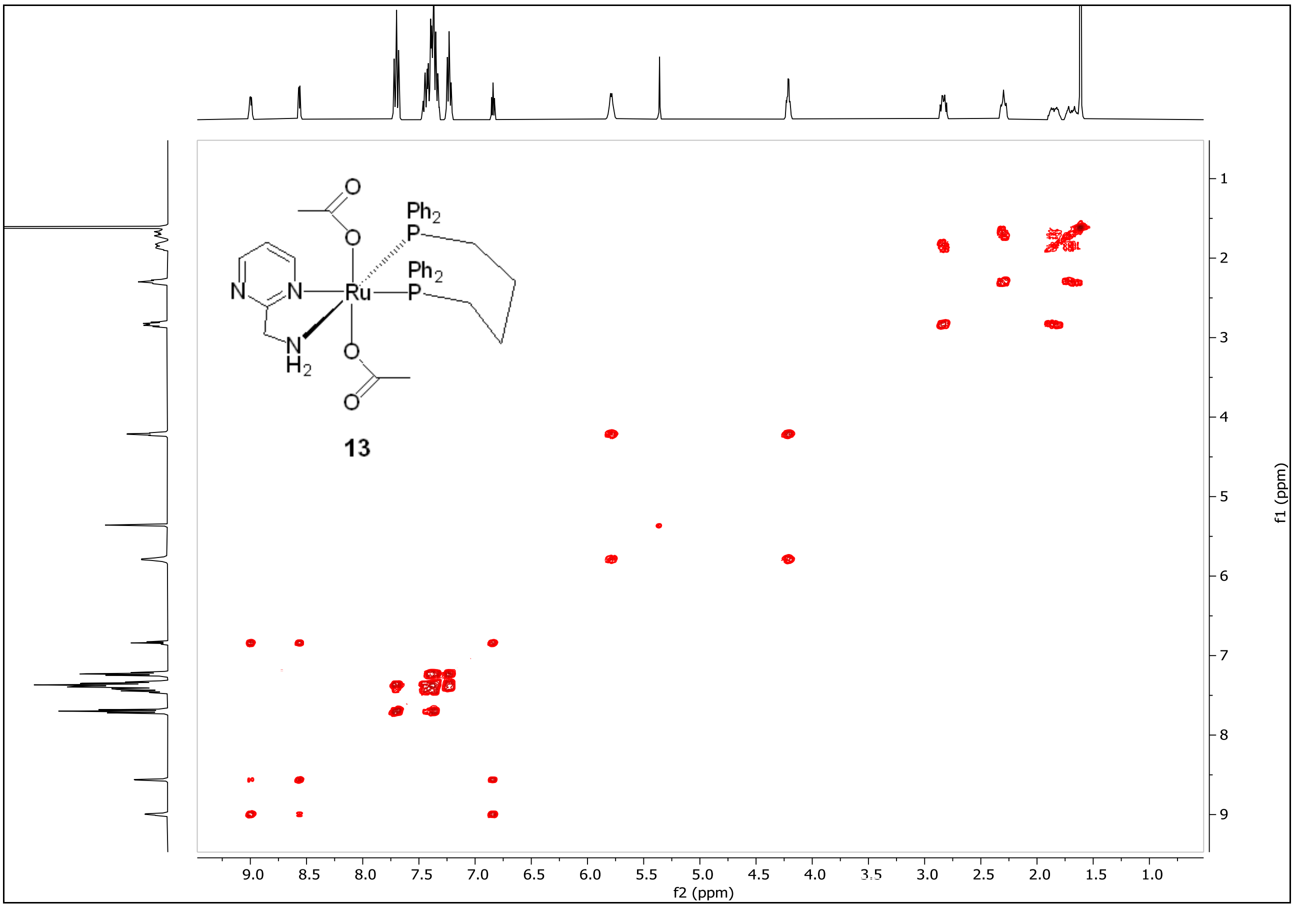

Figure S76. ^1H-^1H COSY 2D NMR spectrum (400.1 MHz) of *trans*-[Ru(η^1-OAc)$_2$(dppb)(ampyrim)] (**13**) in CD_2Cl_2 at 25 °C.

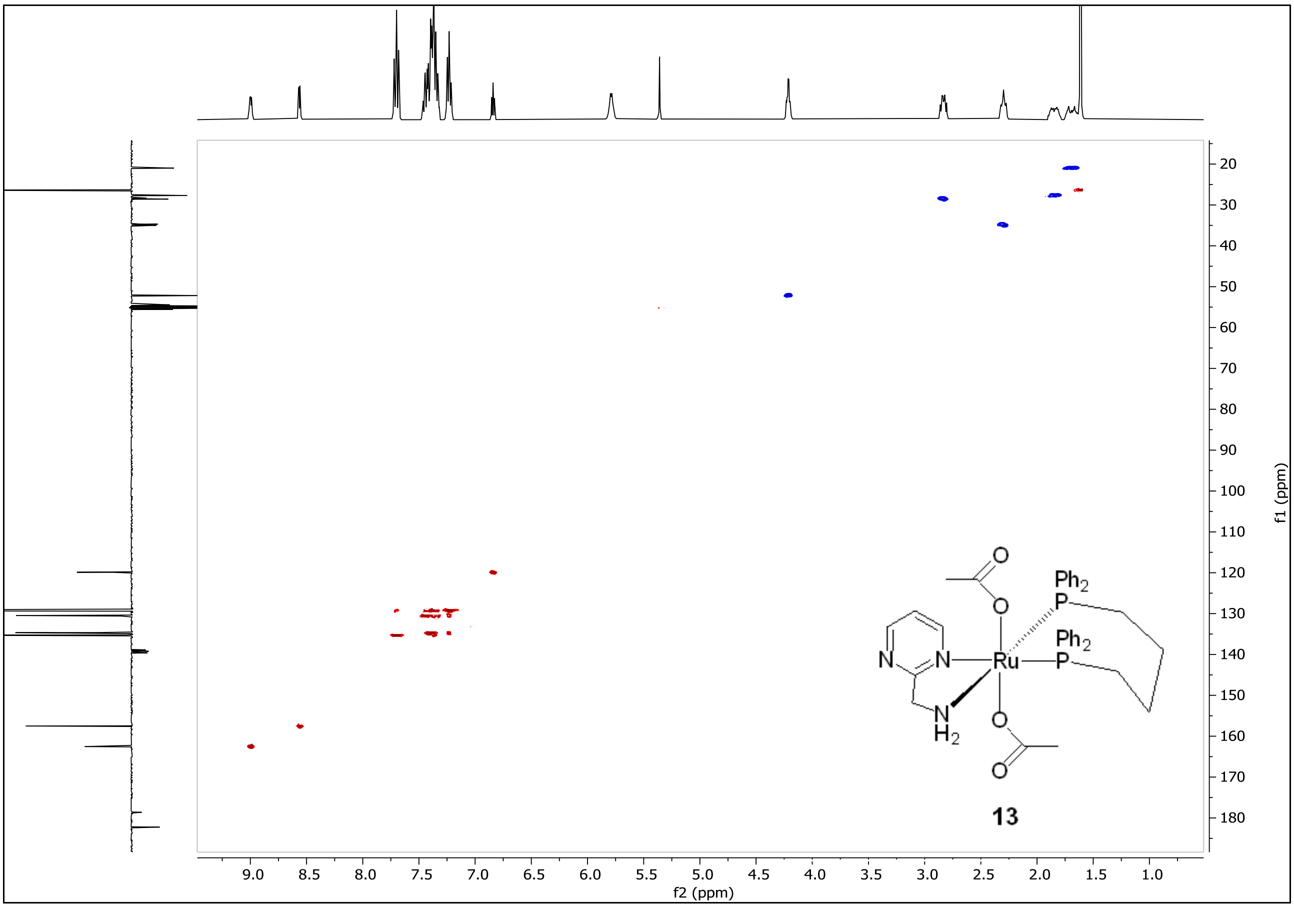

Figure S77. ^{1}H-^{13}C HSQC 2D NMR spectrum of *trans*-[Ru(η^1-OAc)$_2$(dppb)(ampyrim)] (**13**) in CD_2Cl_2 at 25 °C.

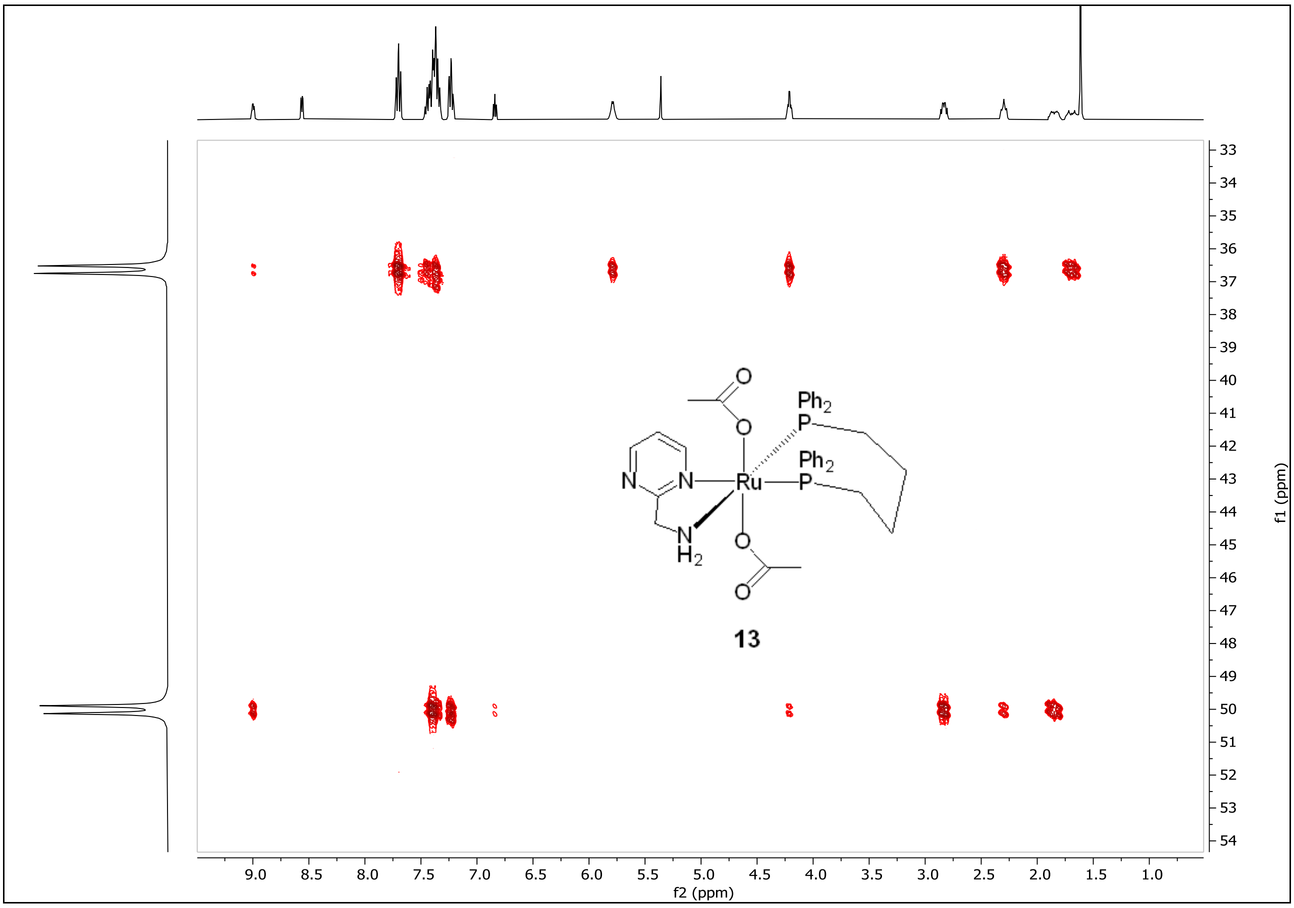

Figure S78. ^{1}H-^{31}P HMBC 2D NMR spectrum of *trans*-[Ru(η^1-OAc)$_2$(dppb)(ampyrim)] (**13**) in CD_2Cl_2 at 25 °C.

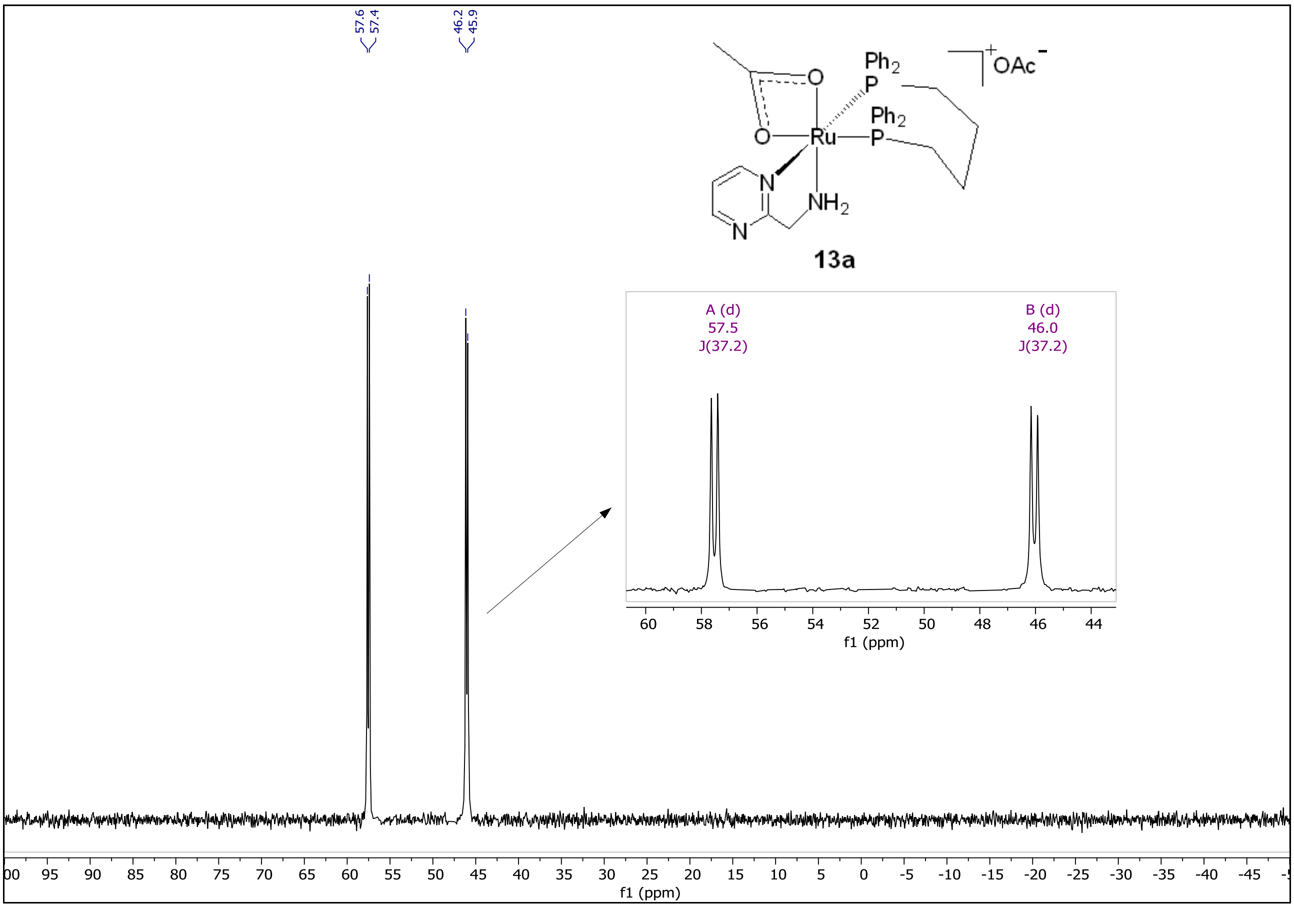

Figure S79. $^{31}P\{^1H\}$ NMR spectrum (162.0 MHz) of [Ru(η²-OAc)(dppb)(ampyrim)]OAc (**13a**) in CD_3OD at 25 °C.

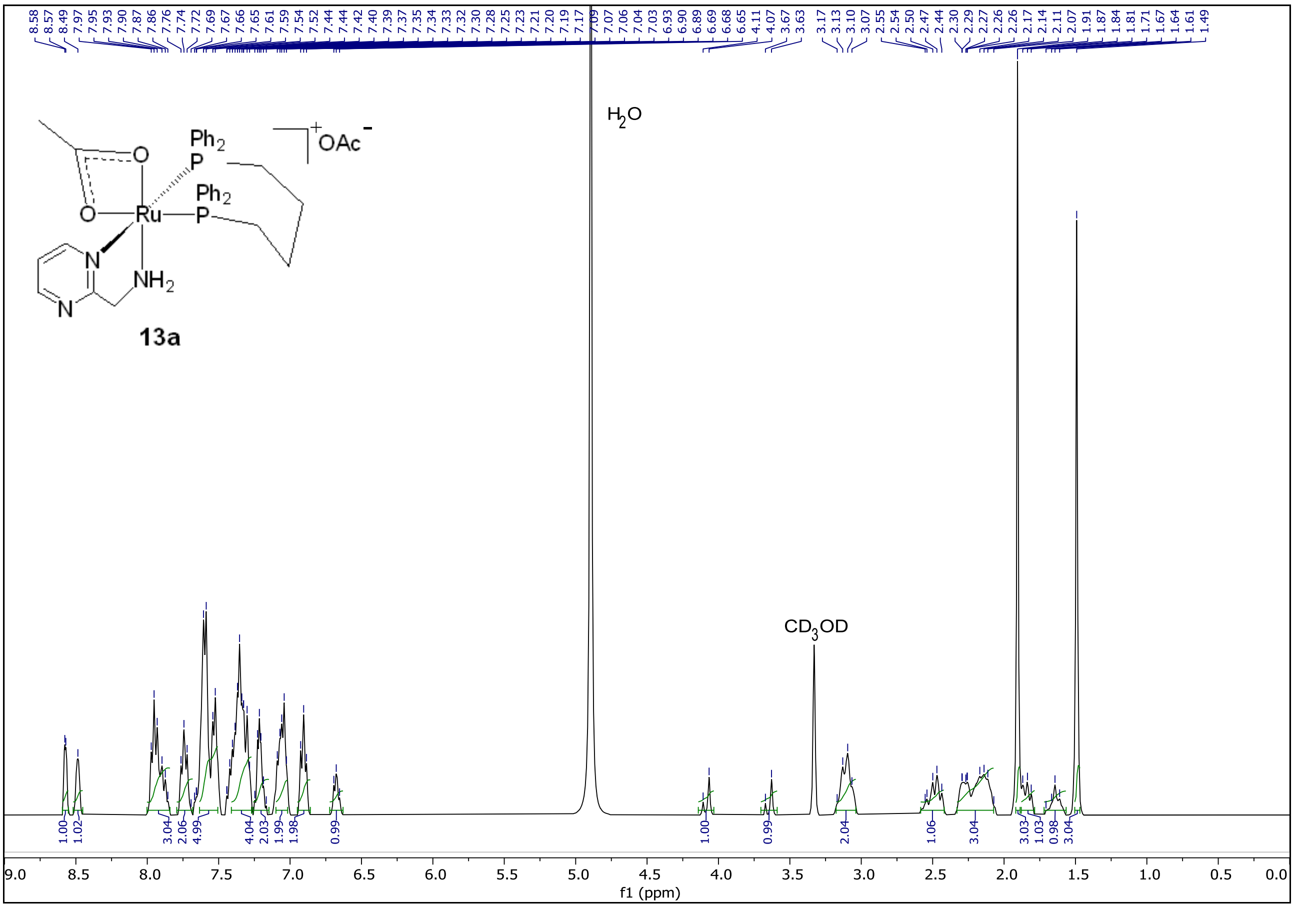

Figure S80. ^{1}H NMR spectrum (400.1 MHz) of [Ru(η^2-OAc)(dppb)(ampyrim)]OAc (**13a**) in CD_3OD at 25 °C.

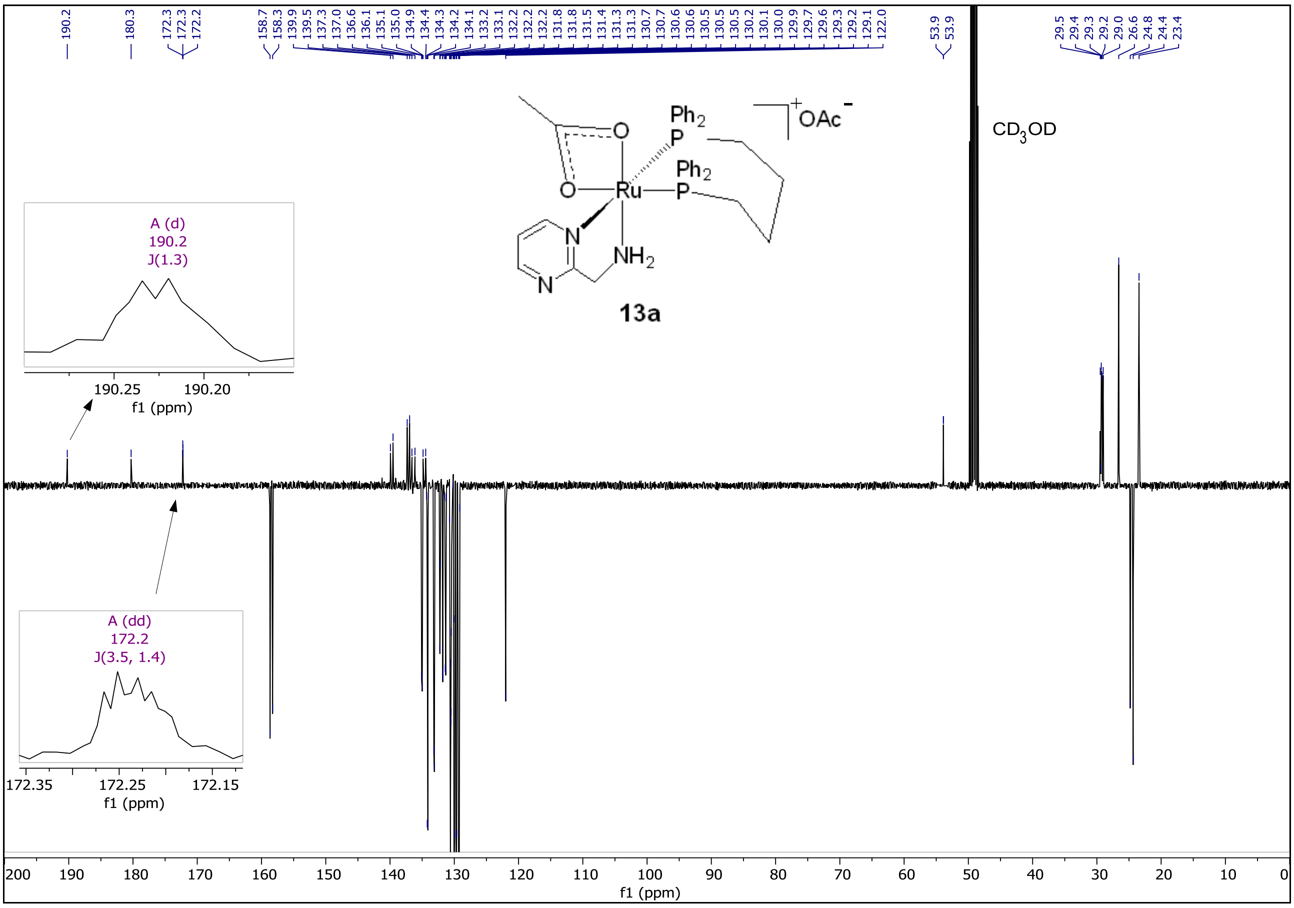

Figure S81. $^{13}C\{^{1}H\}$ DEPTQ NMR spectrum (100.6 MHz) of [Ru(η^2-OAc)(dppb)(ampyrim)]OAc (**13a**) in CD_3OD at 25 °C.

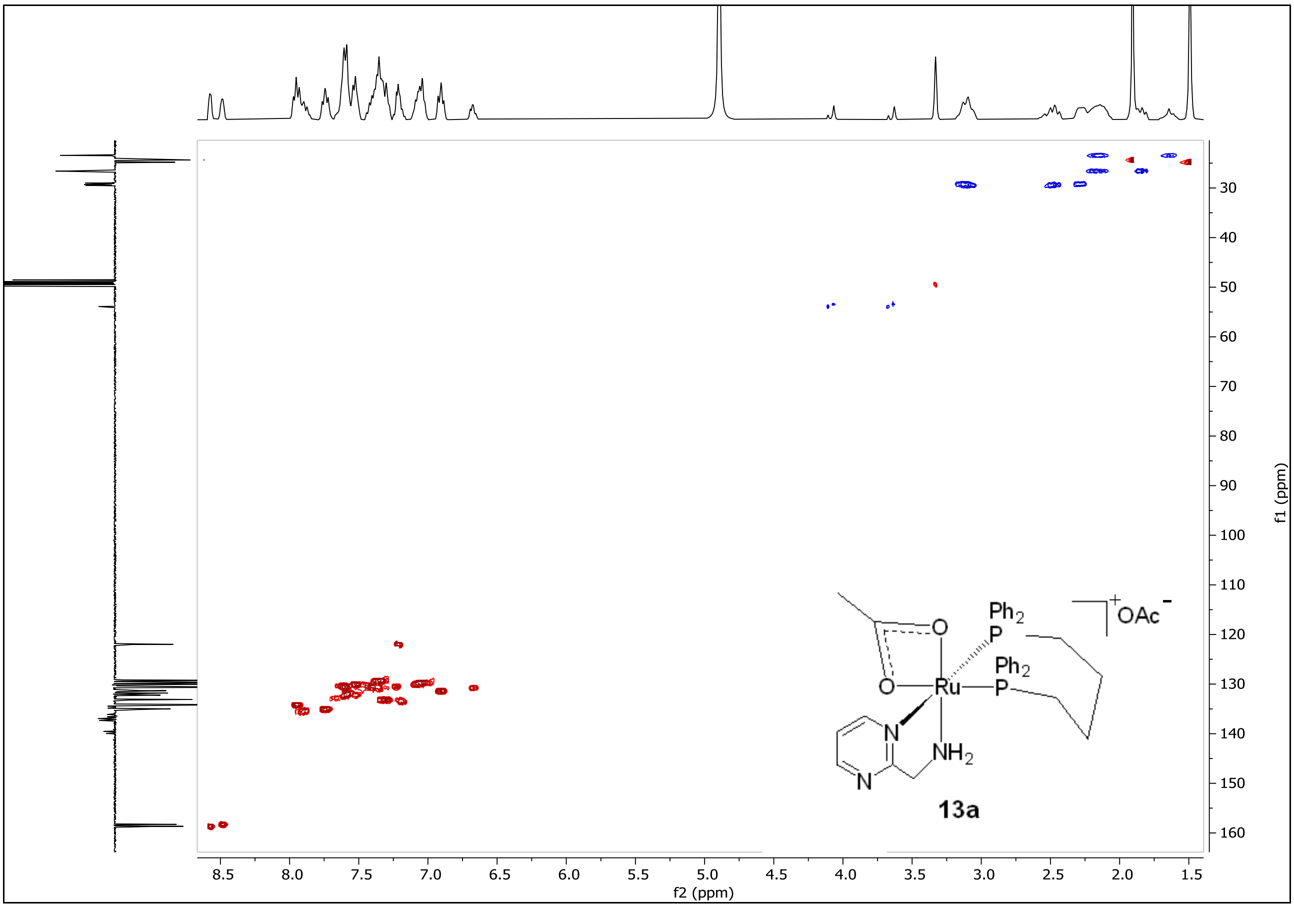

Figure S82. ^{1}H-^{13}C HSQC 2D NMR spectrum of [Ru(η^{2}-OAc)(dppb)(ampyrim)]OAc (**13a**) in CD_3OD at 25 °C.

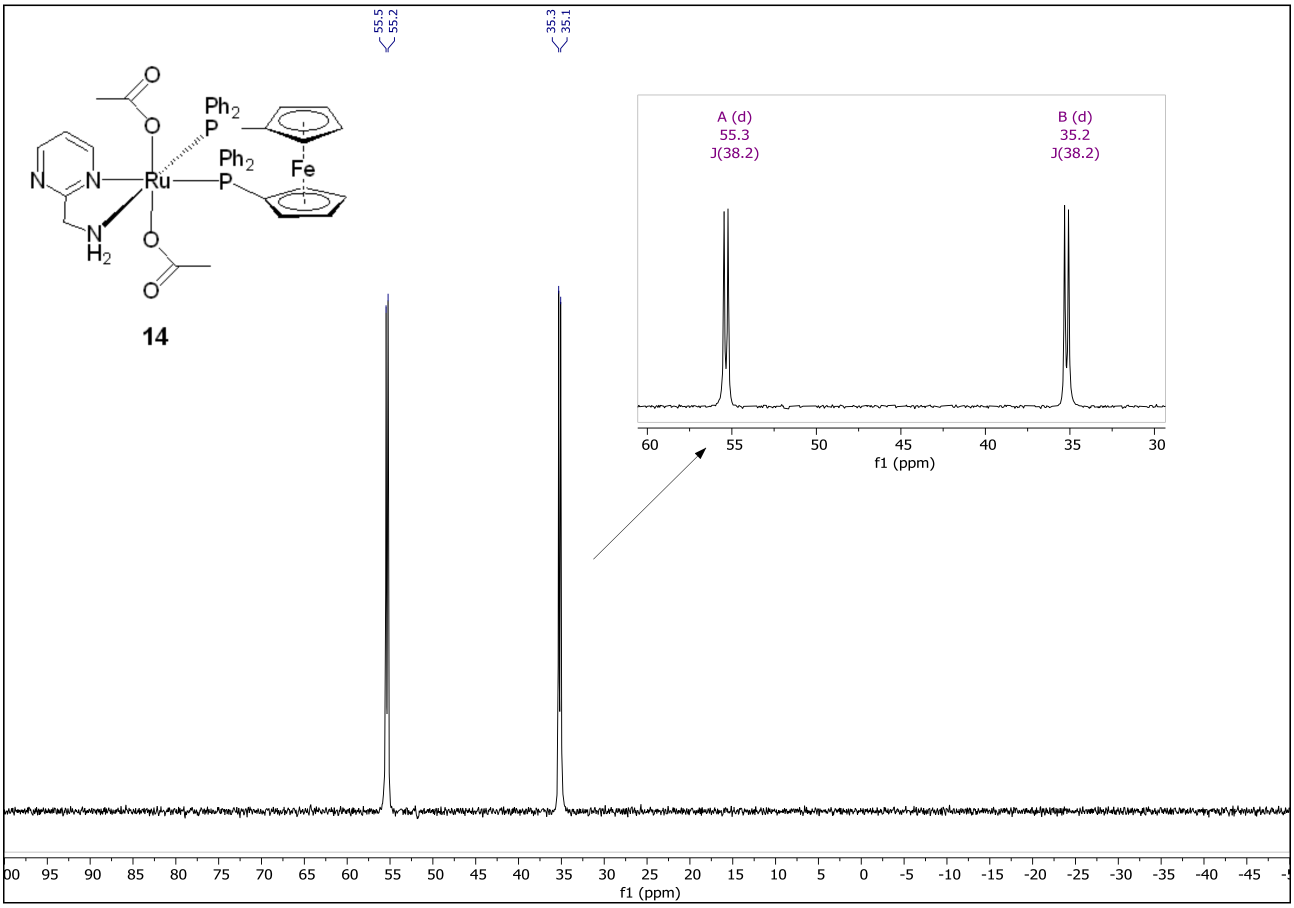

Figure S83. $^{31}P\{^{1}H\}$ NMR spectrum (162.0 MHz) of *trans*-[Ru(η^1-OAc)$_2$(dppf)(ampyrim)] (**14**) in CD_2Cl_2 at 25 °C.

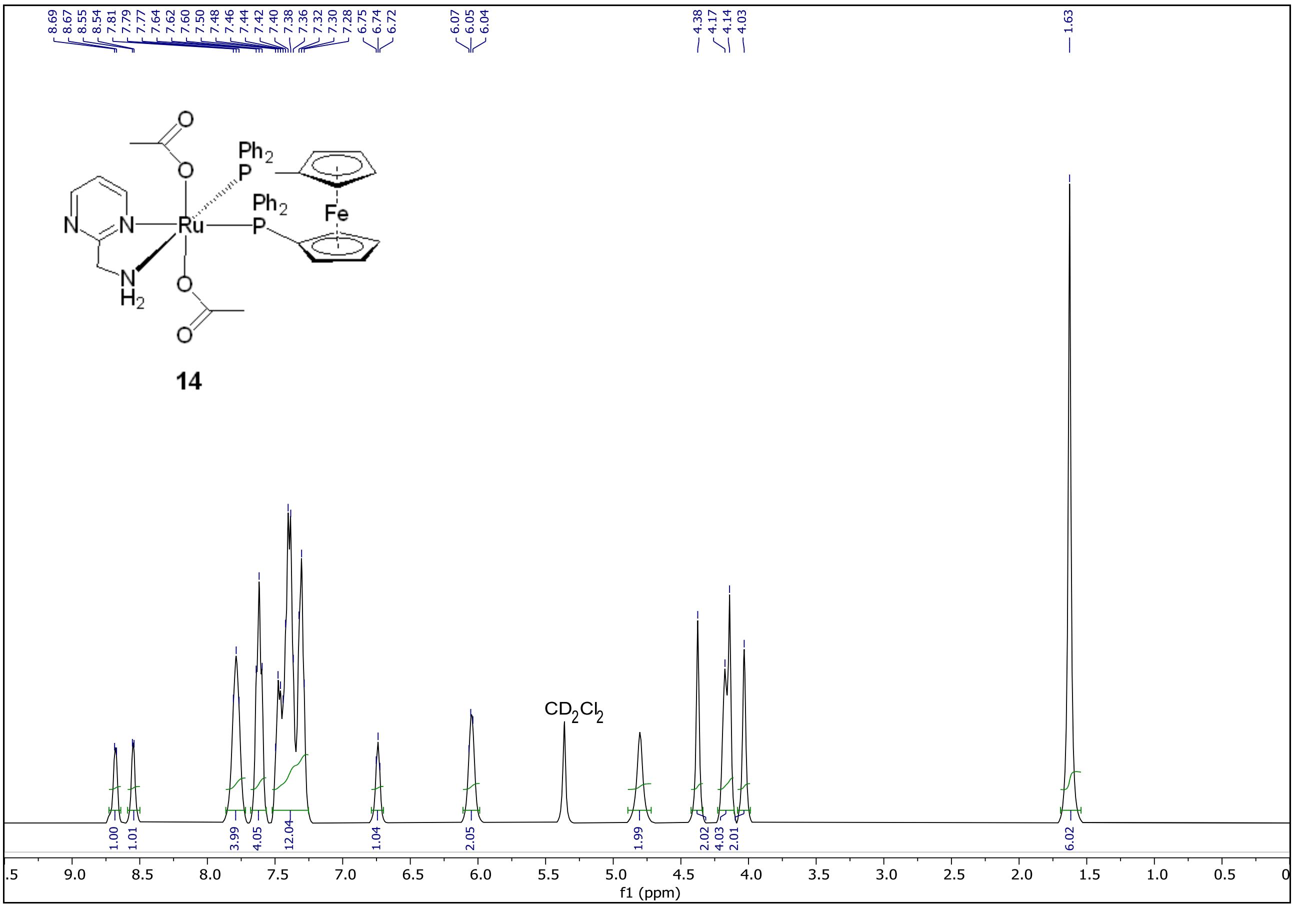

Figure S84. ^{1}H NMR spectrum (400.1 MHz) of *trans*-[Ru(η^1-OAc)$_2$(dppf)(ampyrim)] (**14**) in CD_2Cl_2 at 25 °C.

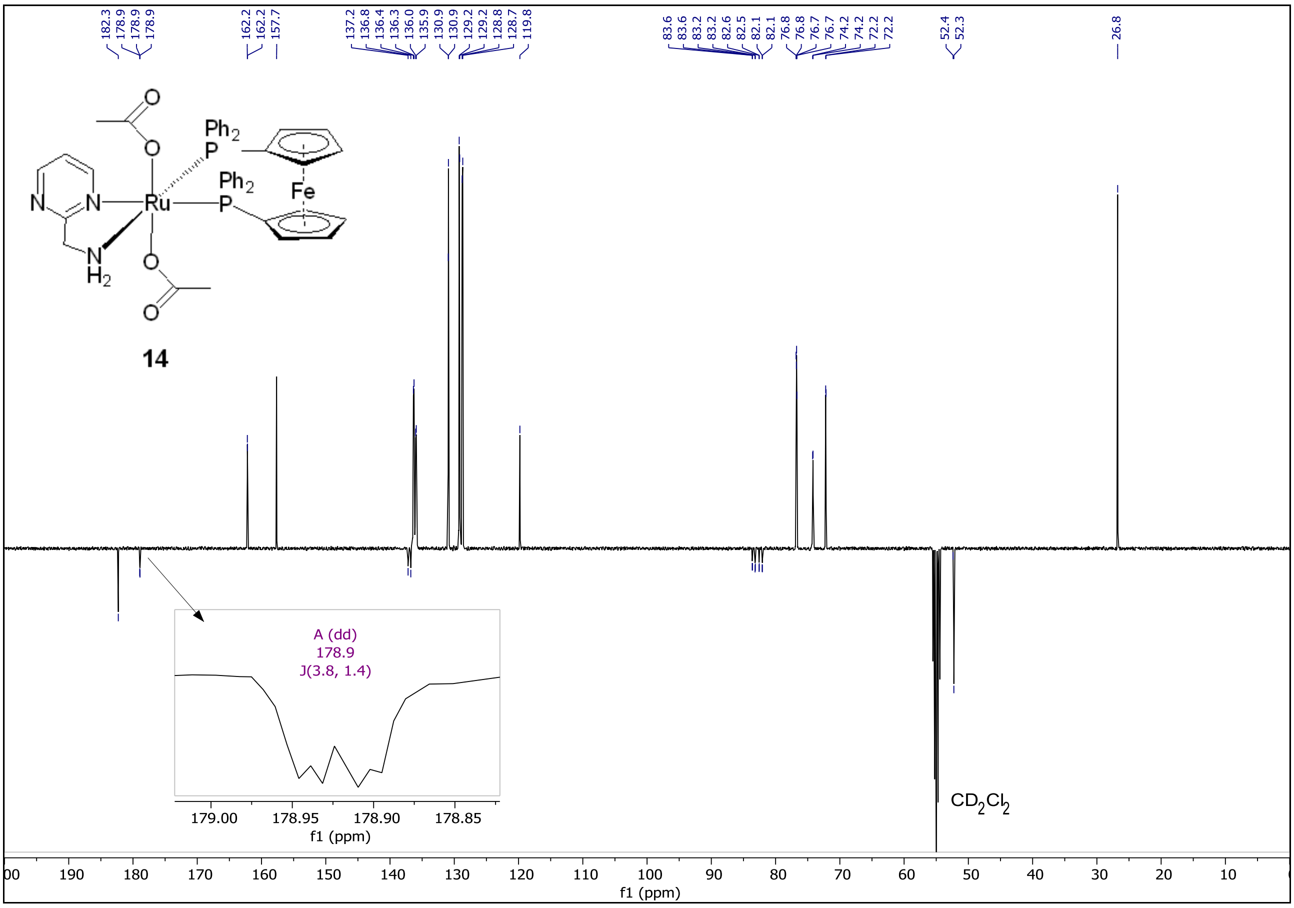

Figure S85. $^{13}C\{^{1}H\}$ DEPTQ NMR spectrum (100.6 MHz) of *trans*-[Ru(η^1-OAc)$_2$(dppf)(ampyrim)] (**14**) in CD_2Cl_2 at 25 °C.

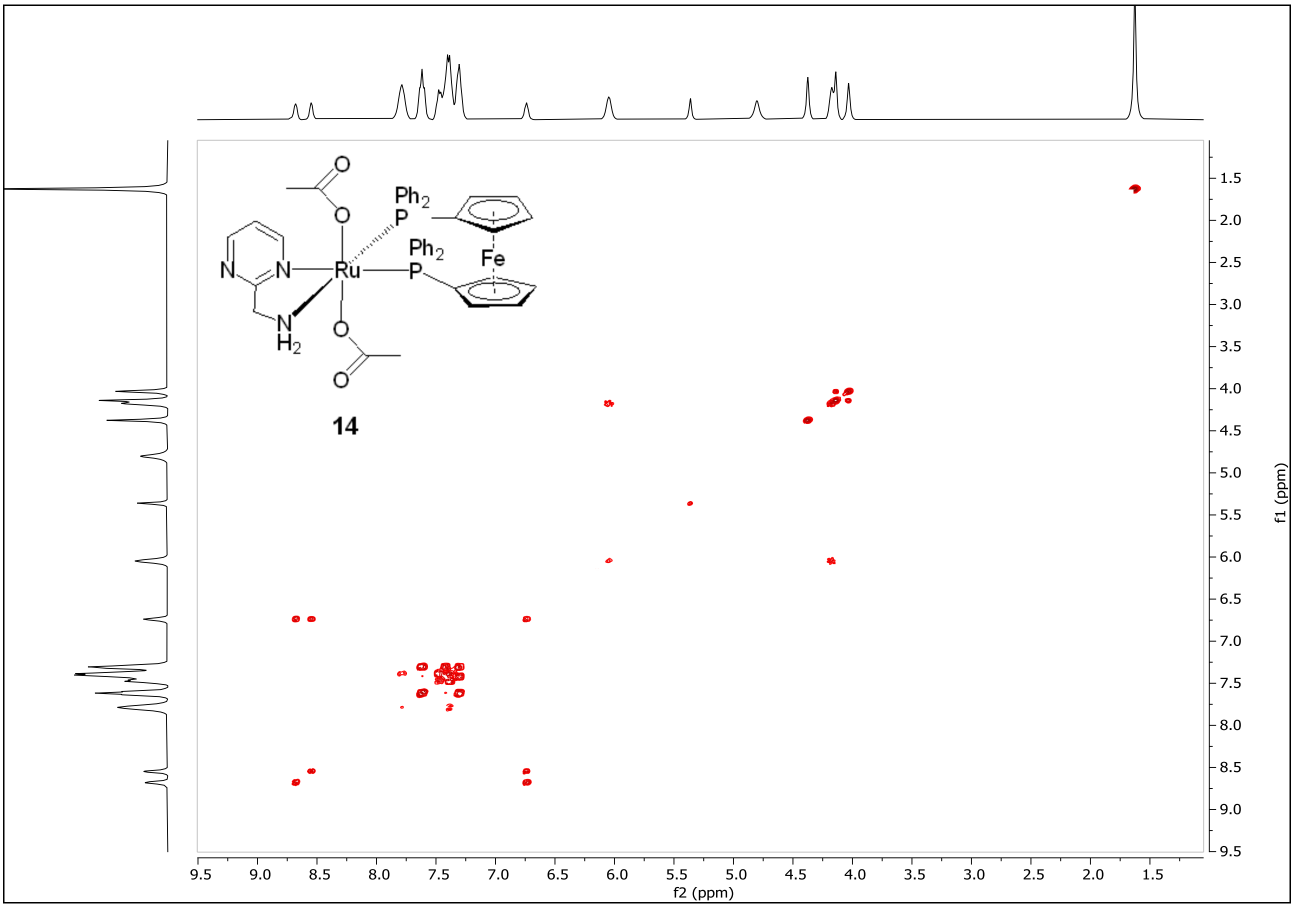

Figure S86. ^{1}H-^{1}H COSY 2D NMR spectrum (400.1 MHz) of *trans*-[Ru(η^{1}-OAc)$_2$(dppf)(ampyrim)] (**14**) in CD_2Cl_2 at 25 °C.

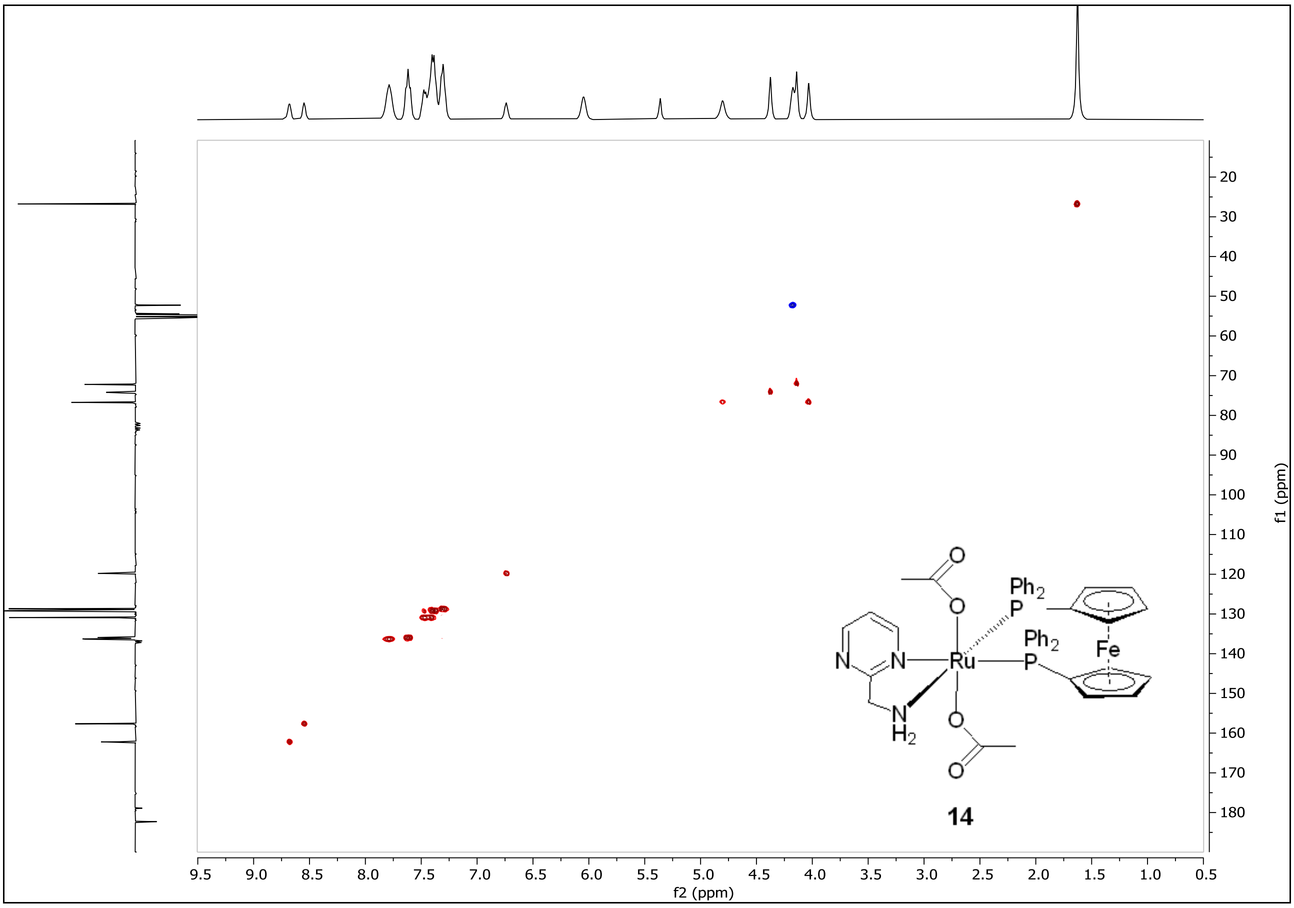

Figure S87. ^{1}H-^{13}C HSQC 2D NMR spectrum of *trans*-[Ru(η^1-OAc)$_2$(dppf)(ampyrim)] (**14**) in CD_2Cl_2 at 25 °C.

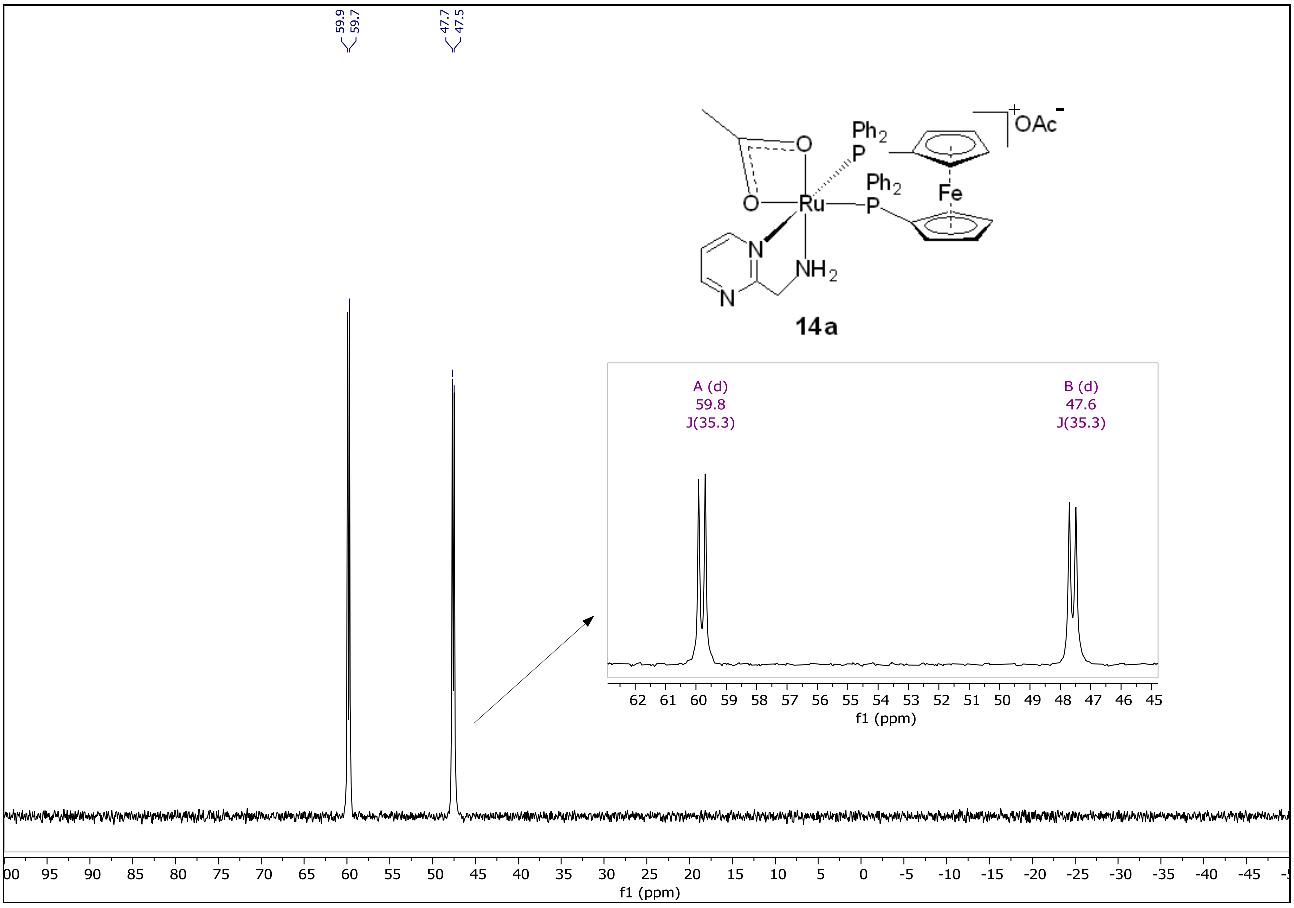

Figure S88. $^{31}P\{^1H\}$ NMR spectrum (162.0 MHz) of [Ru(η^2-OAc)(dppf)(ampyrim)]OAc (**14a**) in CD_3OD at 25 °C.

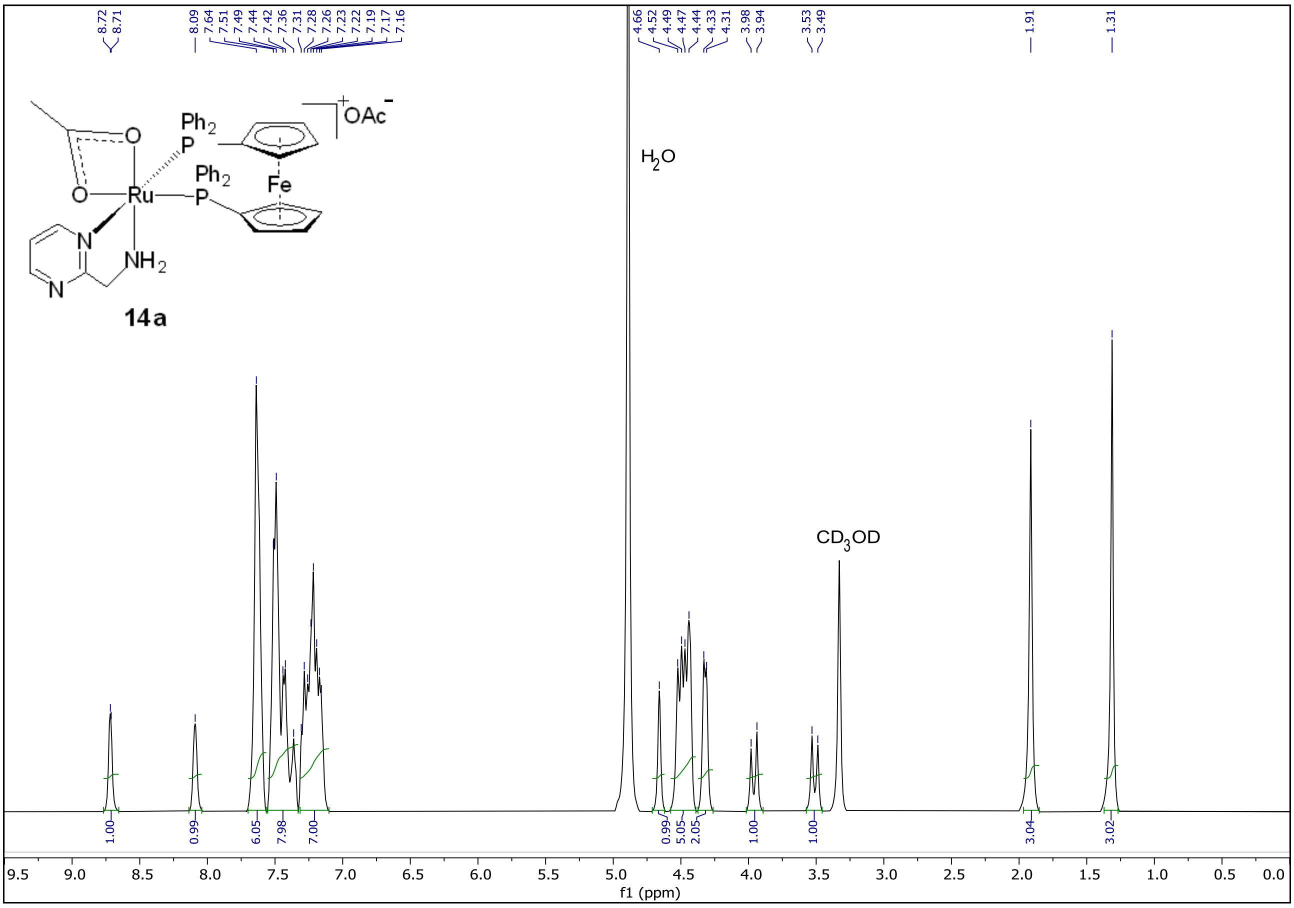

Figure S89. ^{1}H NMR spectrum (400.1 MHz) of [Ru(η^2-OAc)(dppf)(ampyrim)]OAc (**14a**) in CD_3OD at 25 °C.

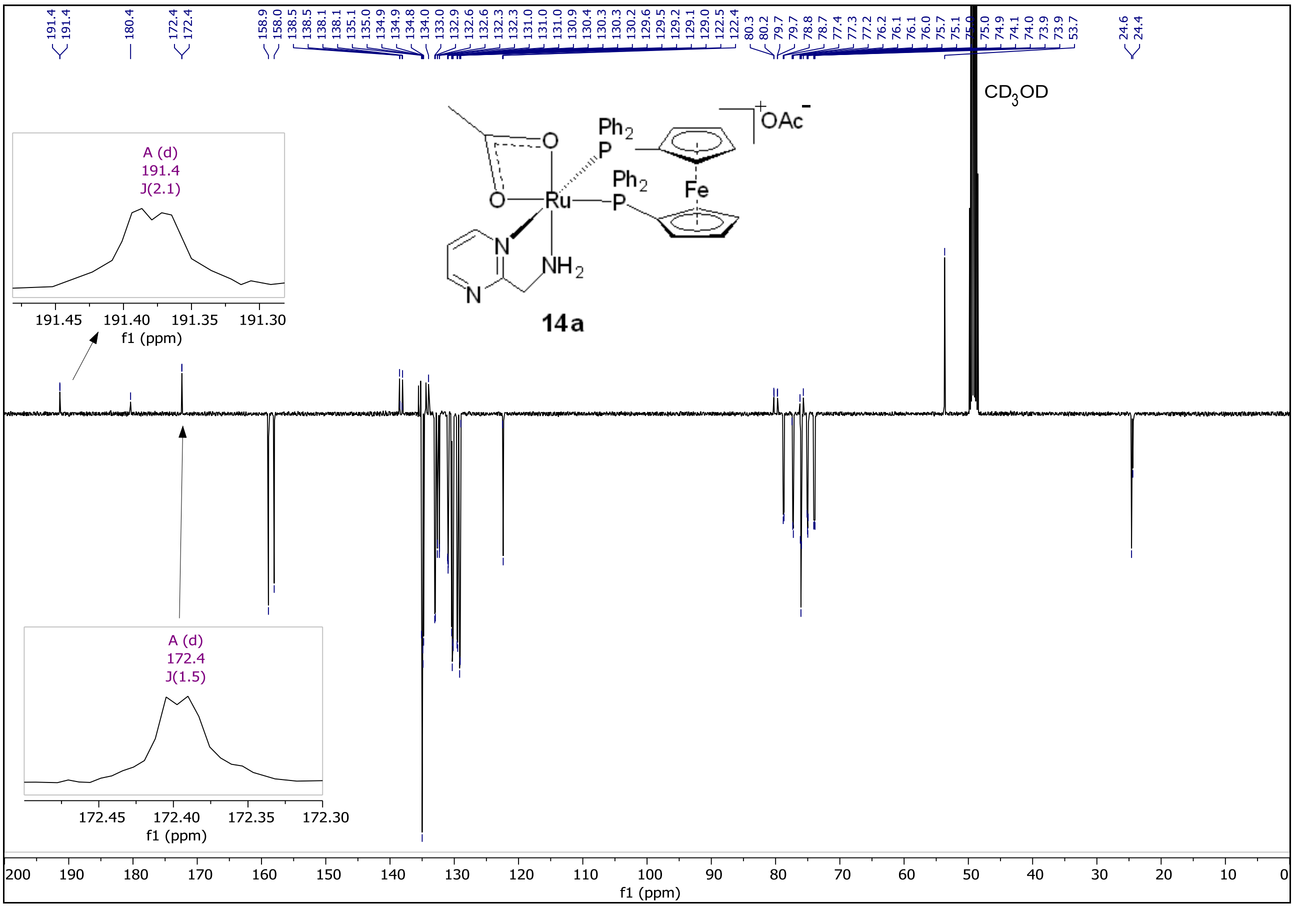

Figure S90. $^{13}C\{^1H\}$ DEPTQ NMR spectrum (100.6 MHz) of [Ru(η^2-OAc)(dppf)(ampyrim)]OAc (**14a**) in CD_3OD at 25 °C.

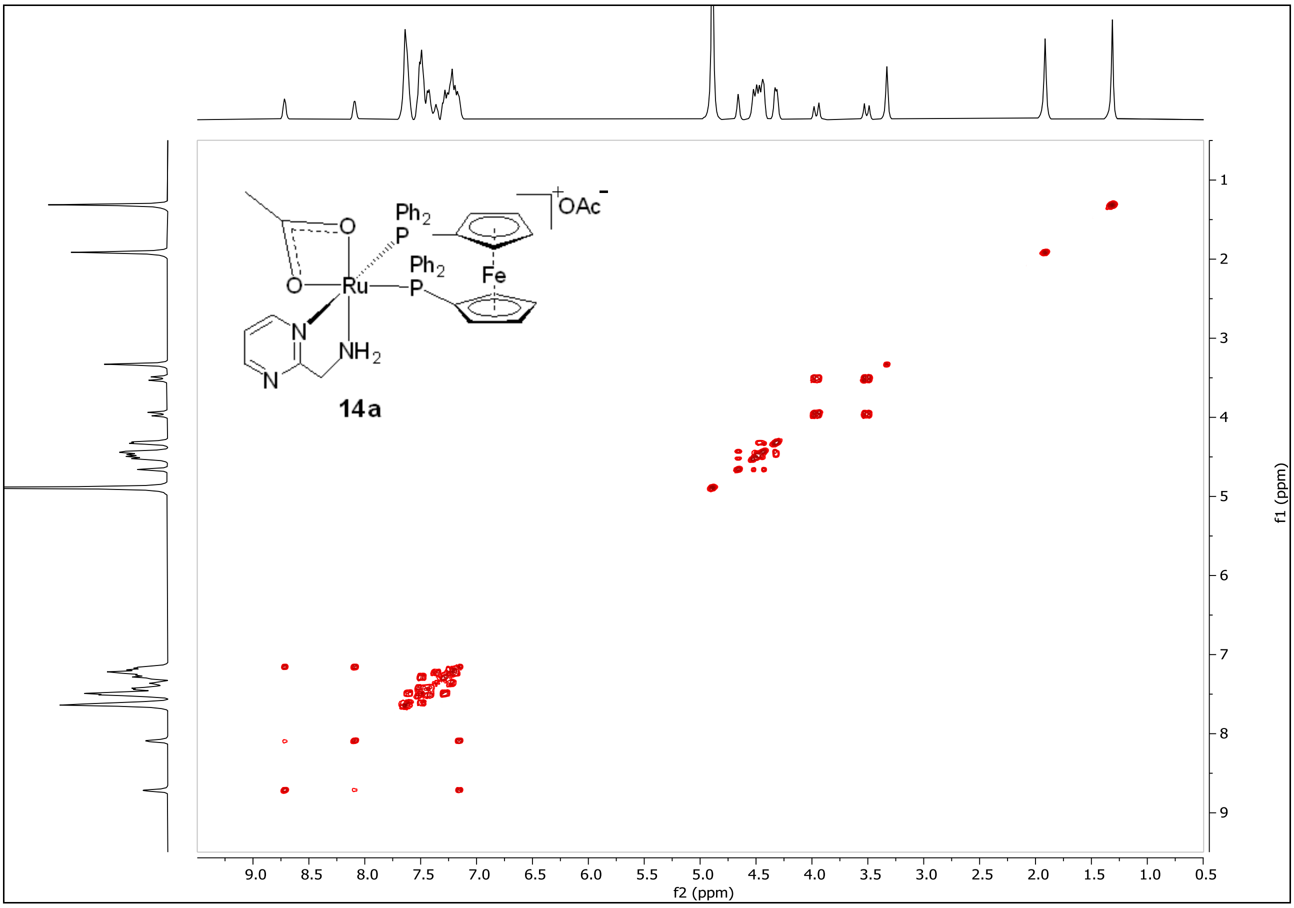

Figure S91. ^{1}H-^{1}H COSY 2D NMR spectrum (400.1 MHz) of [Ru(η^2-OAc)(dppf)(ampyrim)]OAc (**14a**) in CD_3OD at 25 °C.

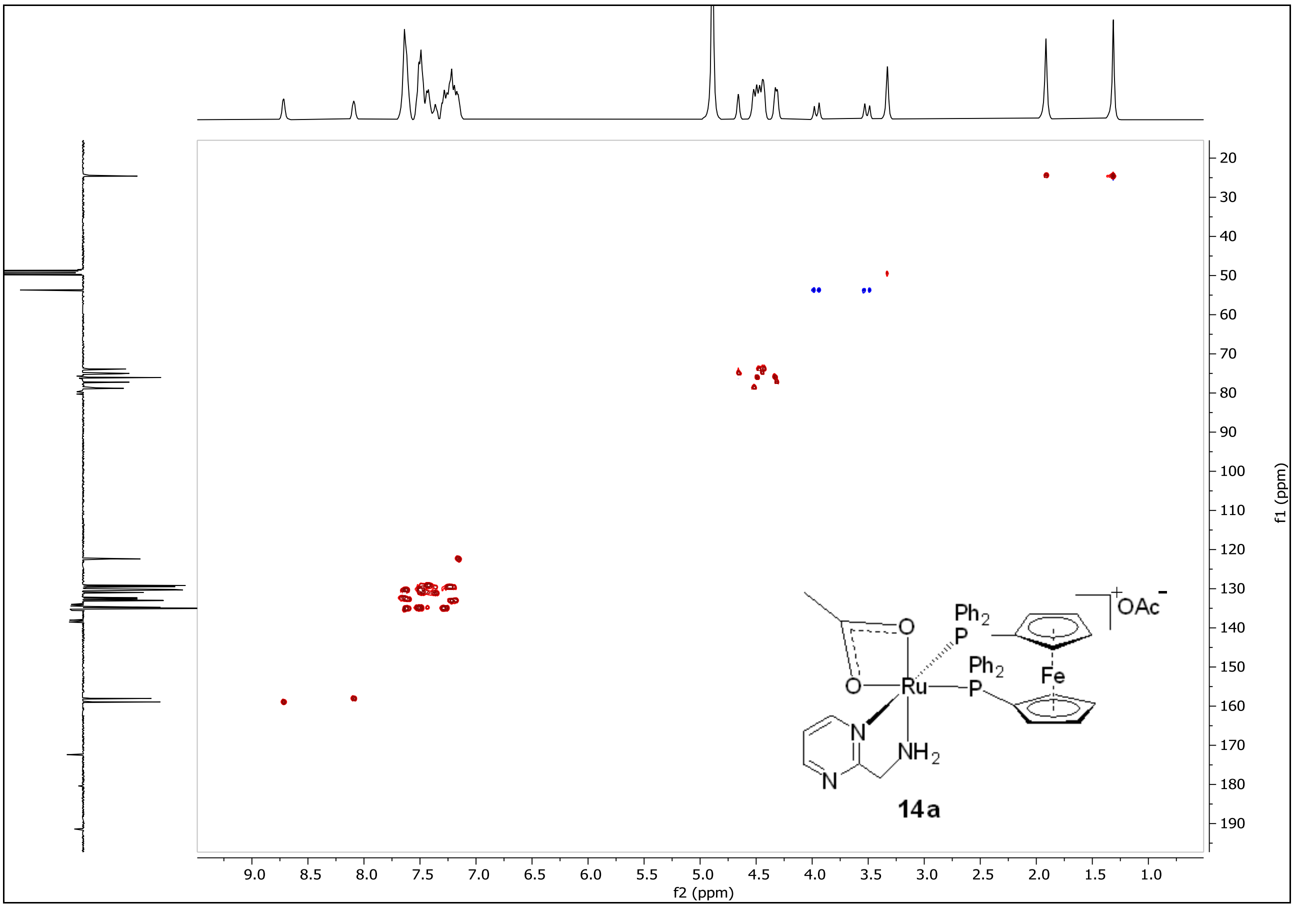

Figure S92. ^{1}H-^{13}C HSQC 2D NMR spectrum of [Ru(η^2-OAc)(dppf)(ampyrim)]OAc (**14a**) in CD_3OD at 25 °C.

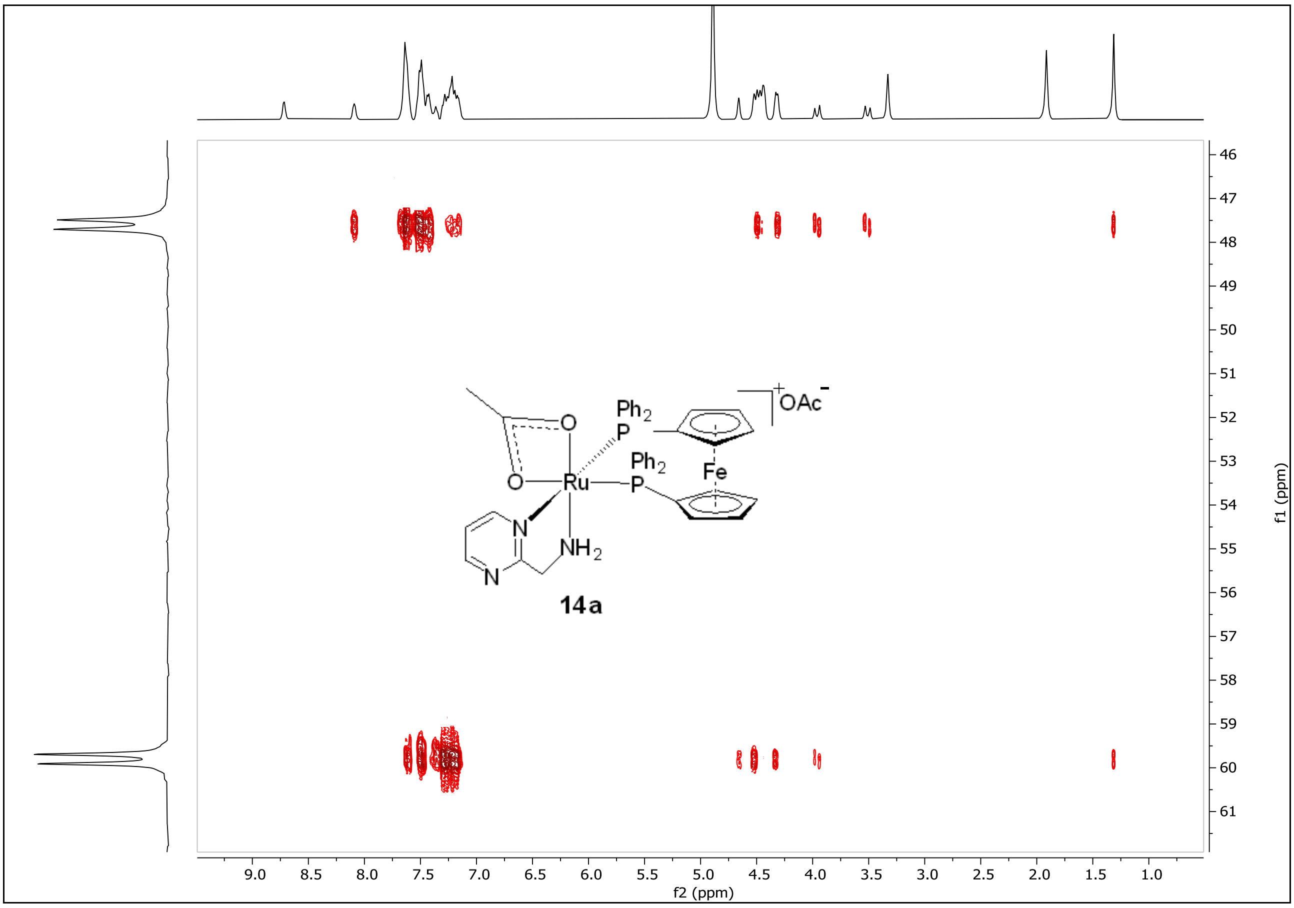

Figure S93. ^{1}H-^{31}P HMBC 2D NMR spectrum of [Ru(η^{2}-OAc)(dppf)(ampyrim)]OAc (**14a**) in CD$_3$OD at 25 °C.

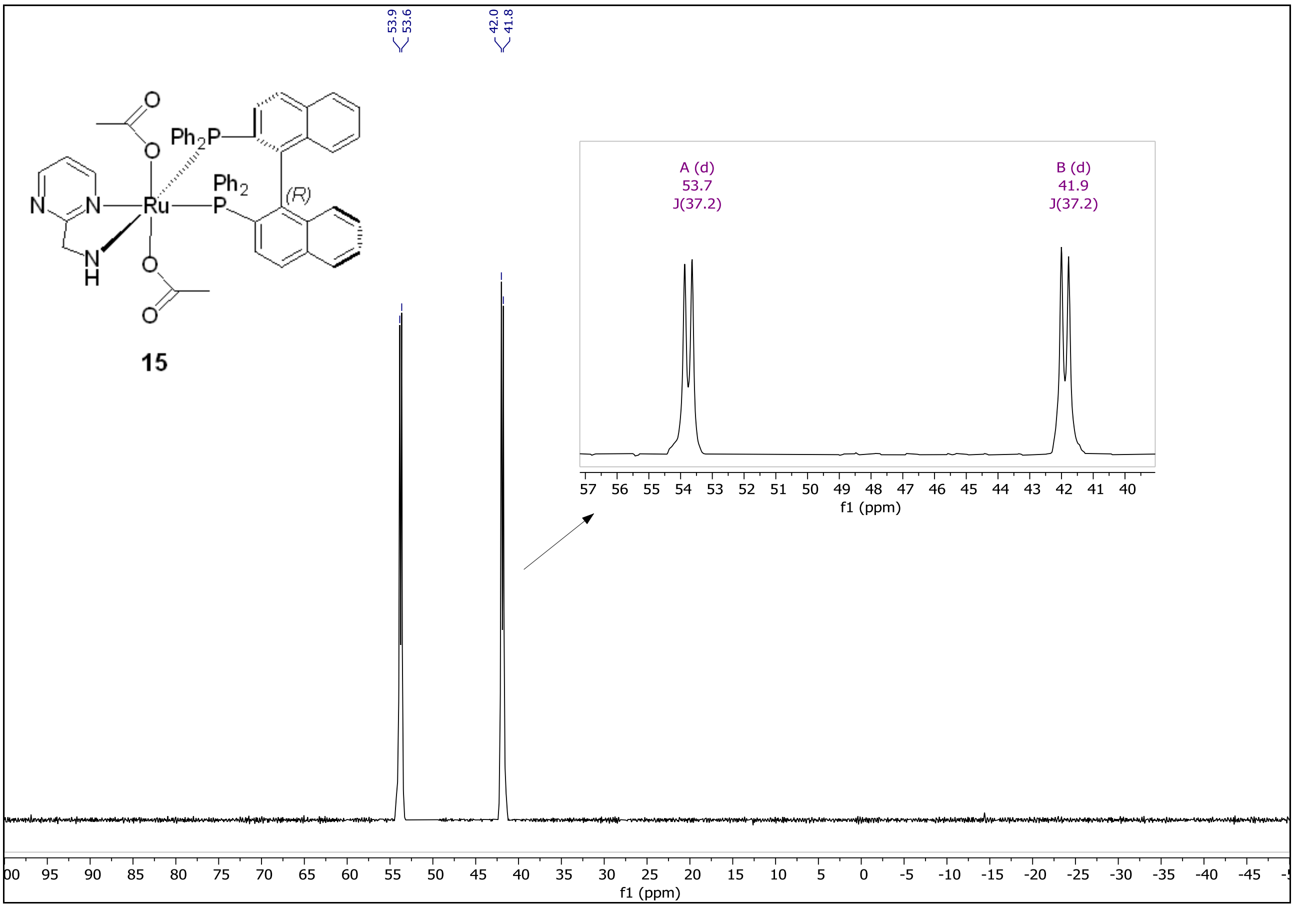

Figure S94. $^{31}P\{^{1}H\}$ NMR spectrum (162.0 MHz) of *trans*-[Ru(η^1-OAc)$_2$((*R*)-BINAP)(ampyrim)] (**15**) in CD_2Cl_2 at 25 °C.

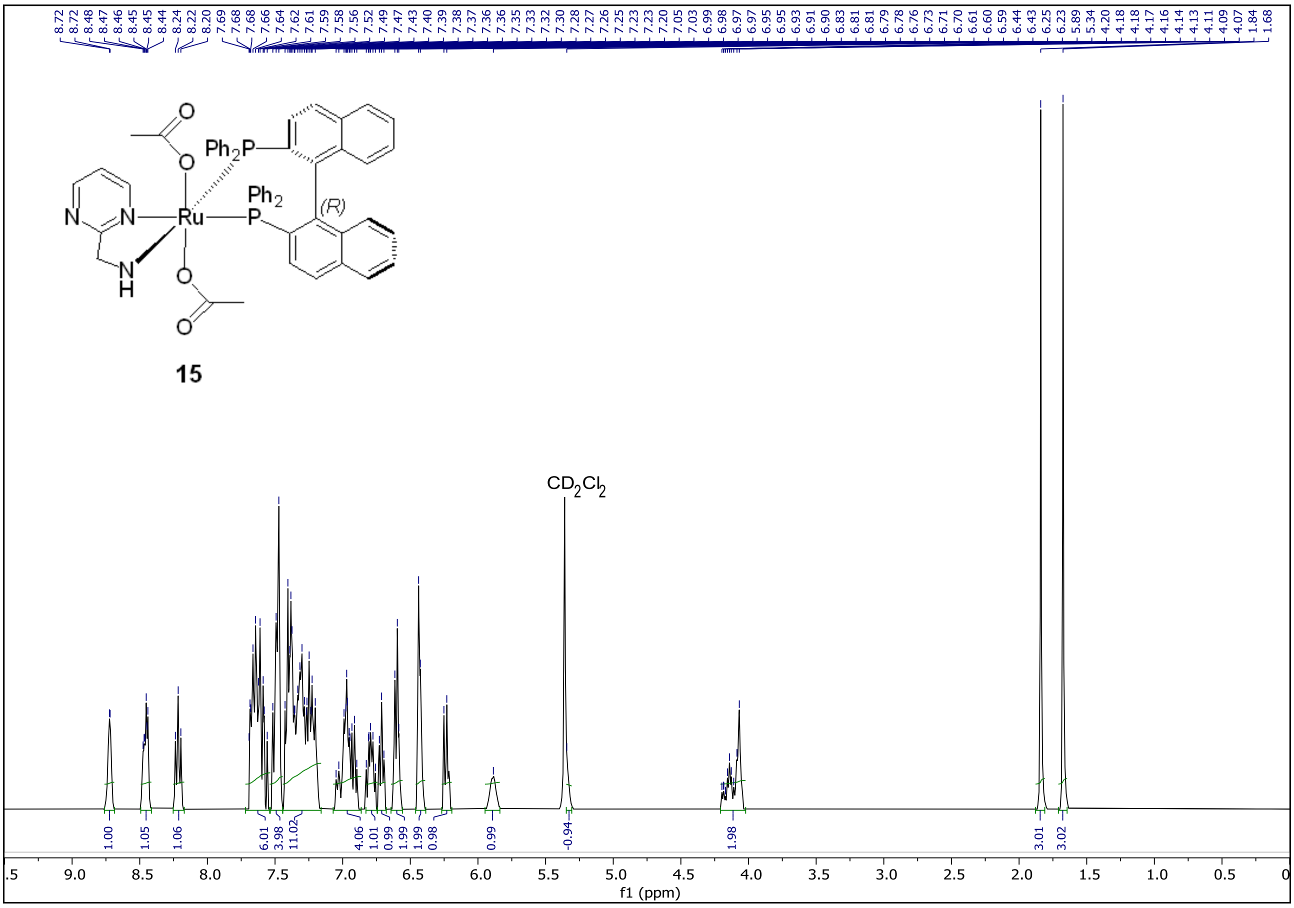

Figure S95. ^{1}H NMR spectrum (400.1 MHz) of *trans*-[Ru(η^{1}-OAc)$_2$((*R*)-BINAP)(ampyrim)] (**15**) in CD_2Cl_2 at 25 °C.

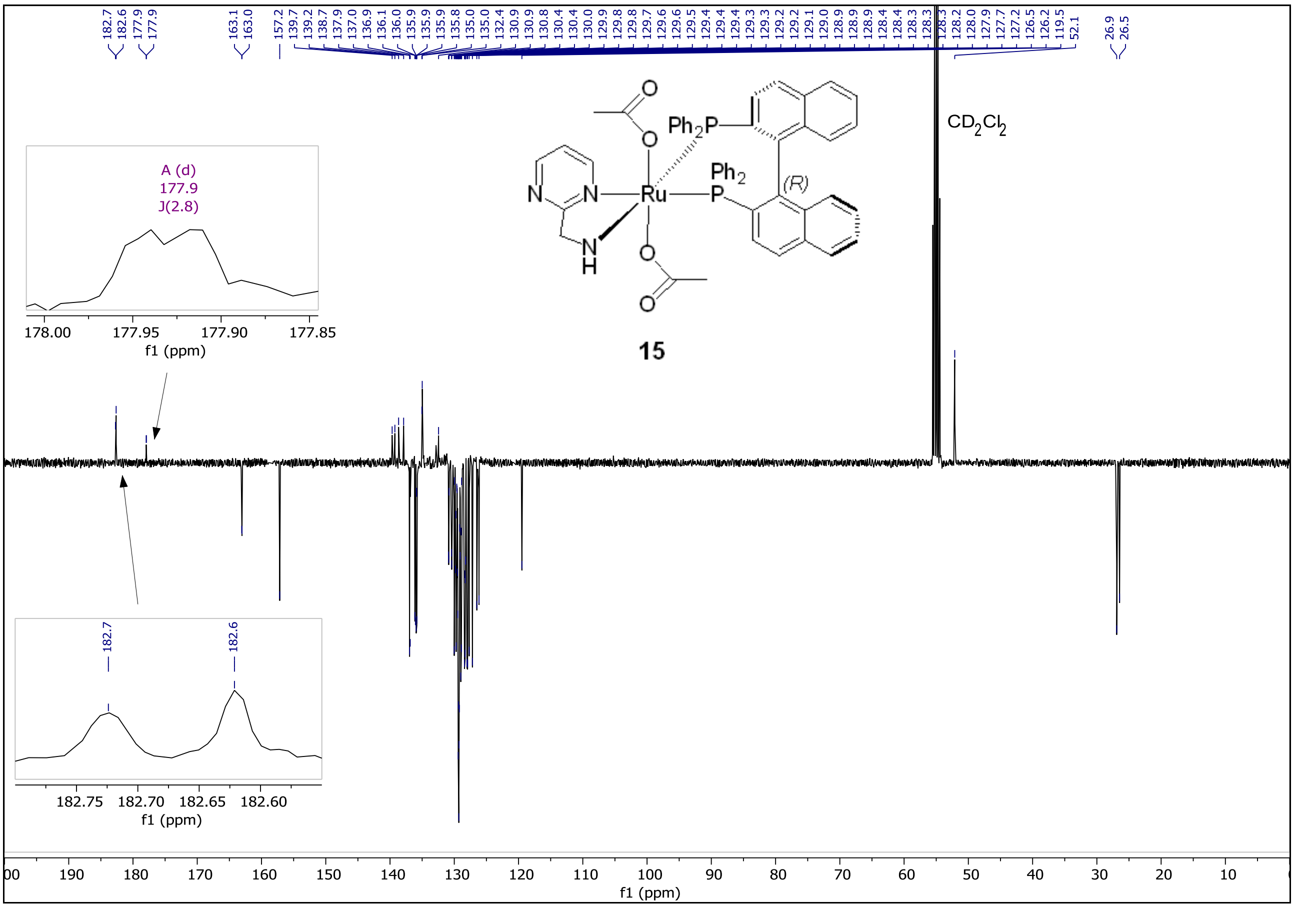

Figure S96. $^{13}C\{^{1}H\}$ DEPTQ NMR spectrum (100.6 MHz) of *trans*-[Ru(η^1-OAc)$_2$((*R*)-BINAP)(ampyrim)] (**15**) in CD_2Cl_2 at 25 °C.

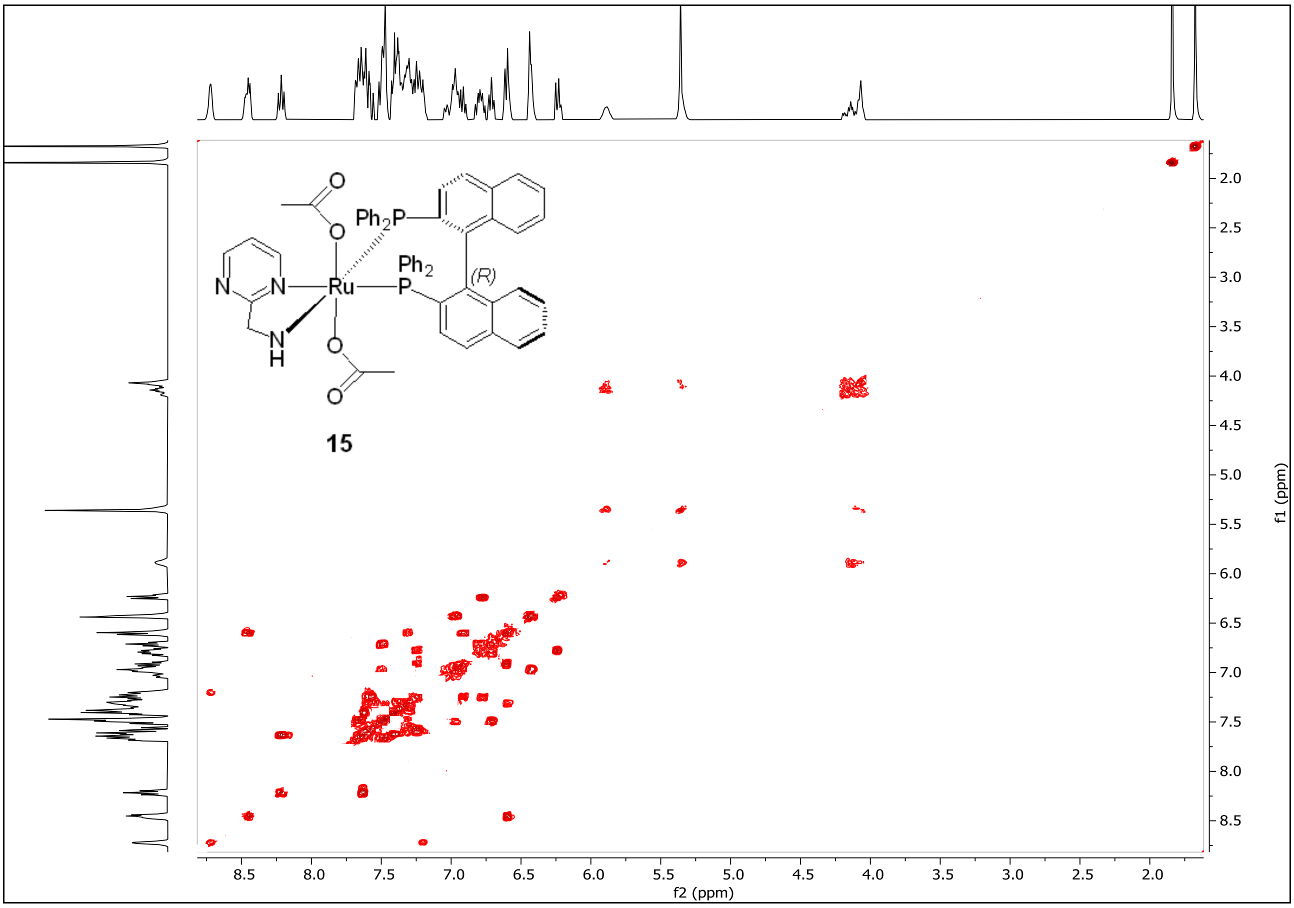

Figure S97. ^{1}H-^{1}H COSY 2D NMR spectrum (400.1 MHz) of *trans*-[Ru(η^1-OAc)$_2$((*R*)-BINAP)(ampyrim)] (**15**) in CD_2Cl_2 at 25 °C.

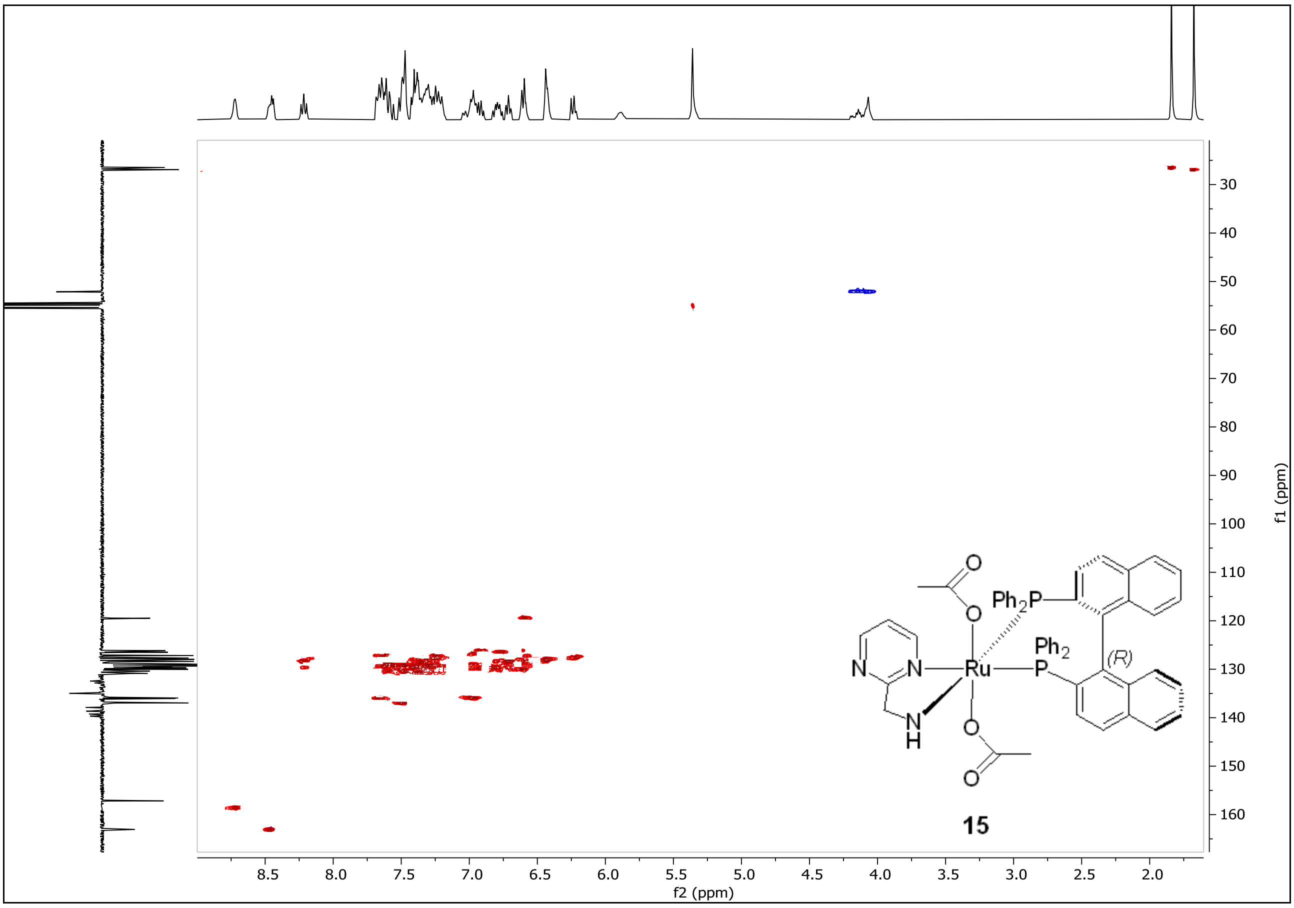

Figure S98. ^{1}H-^{13}C HSQC 2D NMR spectrum of *trans*-[Ru(η^{1}-OAc)$_2$((*R*)-BINAP)(ampyrim)] (**15**) in CD_2Cl_2 at 25 °C.

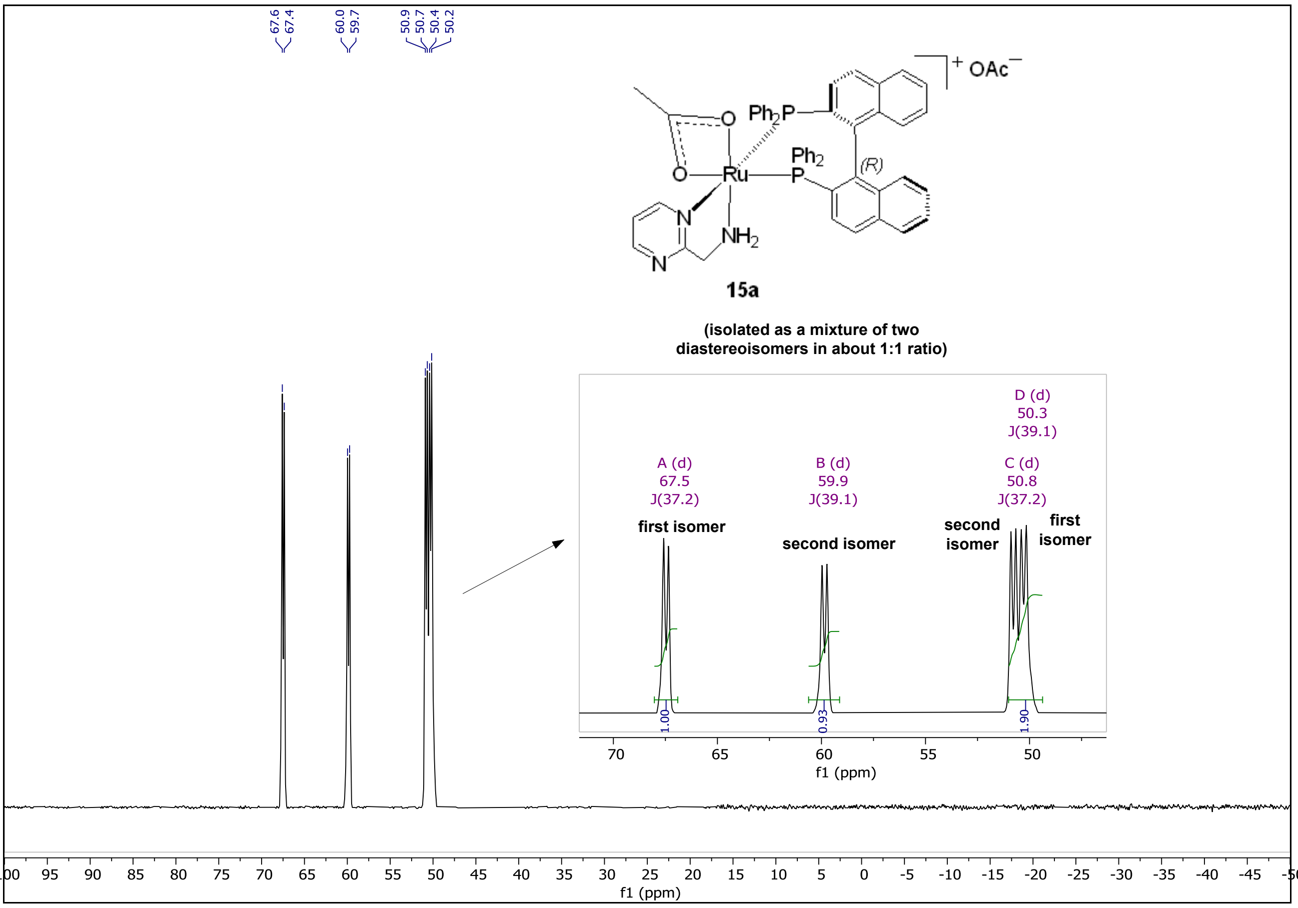

Figure S99. $^{31}P\{^1H\}$ NMR spectrum (162.0 MHz) of [Ru(η^2-OAc)((*R*)-BINAP)(ampyrim)]OAc (**15a**) in CD_3OD at 25 °C.

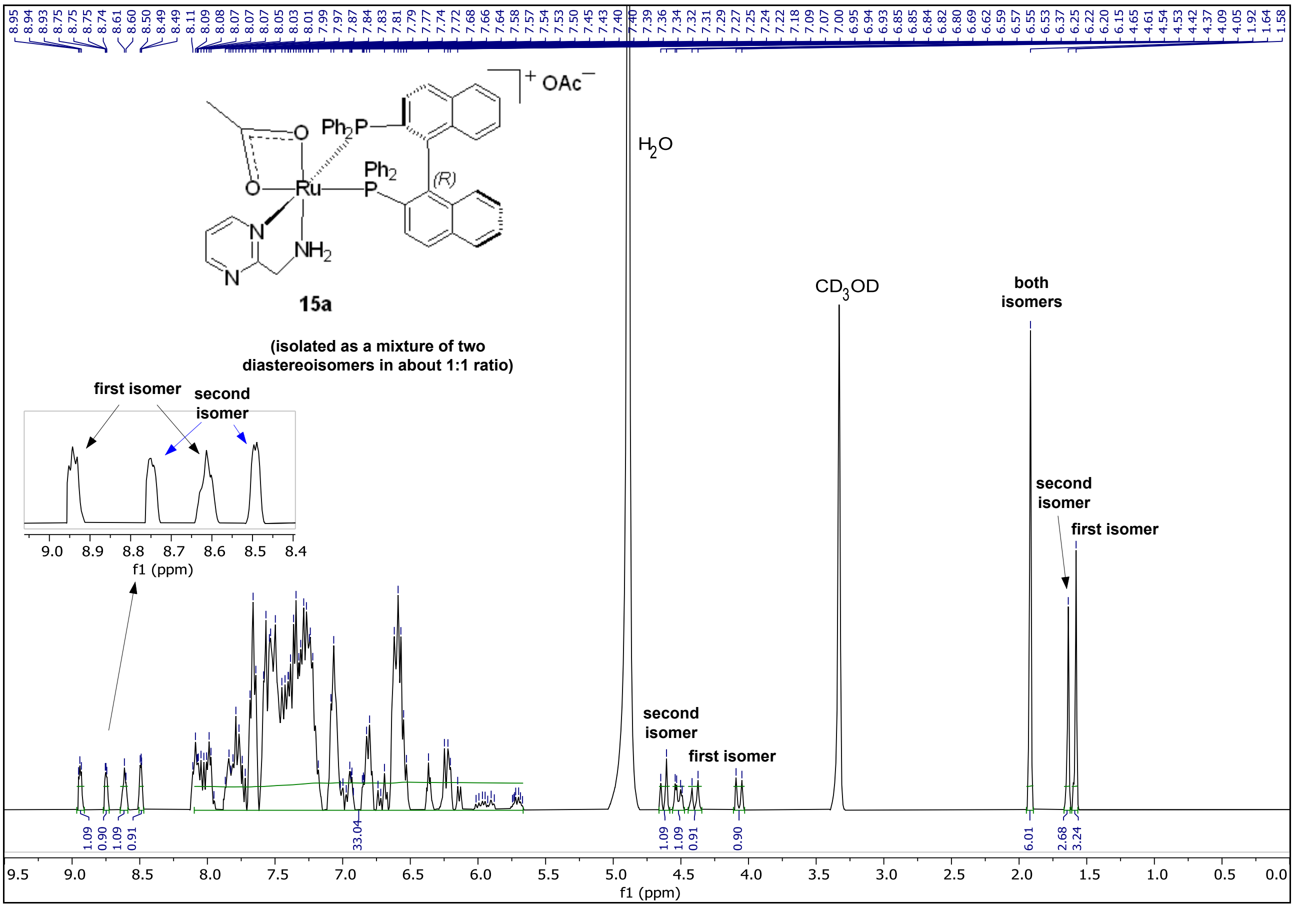

Figure S100. ^{1}H NMR spectrum (400.1 MHz) of [Ru(η^2-OAc)((*R*)-BINAP)(ampyrim)]OAc (**15a**) in CD_3OD at 25 °C.

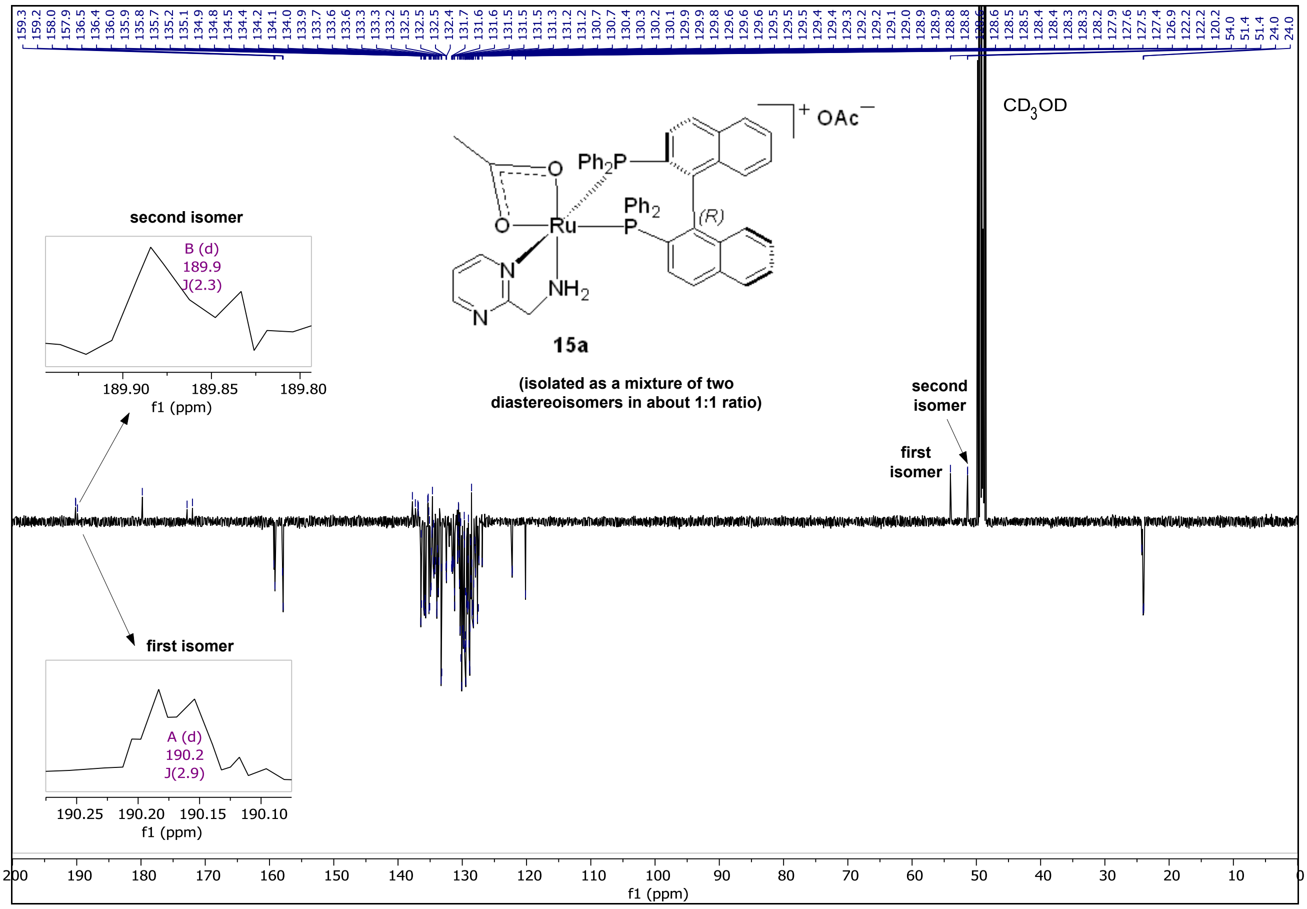

Figure S101. $^{13}C\{^1H\}$ DEPTQ NMR spectrum (100.6 MHz) of [Ru(η^2-OAc)((*R*)-BINAP)(ampyrim)]OAc (**15a**) in CD_3OD at 25 °C.

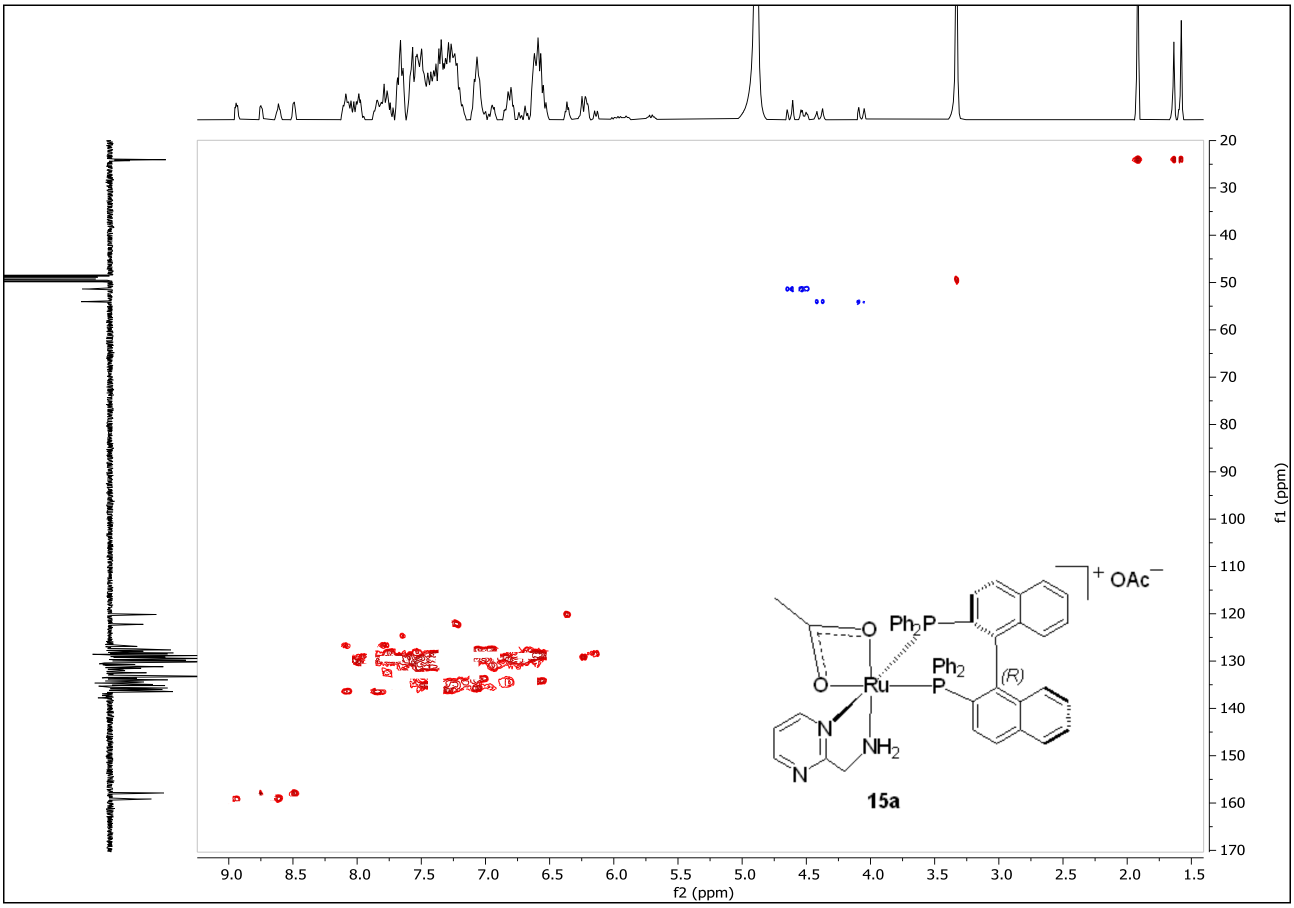

Figure S102. ^{1}H-^{13}C HSQC 2D NMR spectrum of [Ru(η^2-OAc)((*R*)-BINAP)(ampyrim)]OAc (**15a**) in CD_3OD at 25 °C.

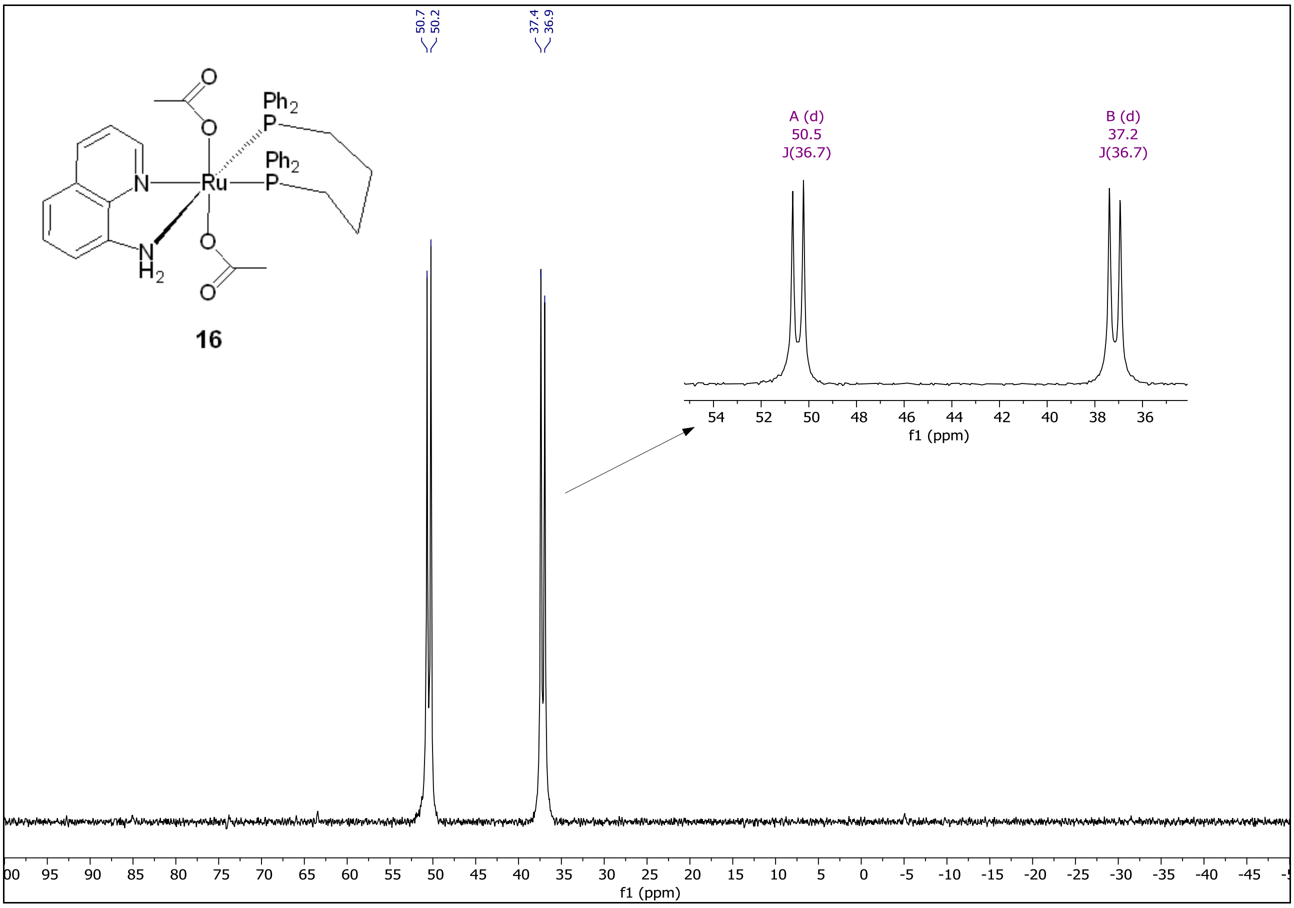

Figure S103. $^{31}P\{^{1}H\}$ NMR spectrum (81.0 MHz) of *trans*-[Ru(η^1-OAc)$_2$(dppb)(8-aminoquinoline)] (**16**) in CD_2Cl_2 at 20 °C.

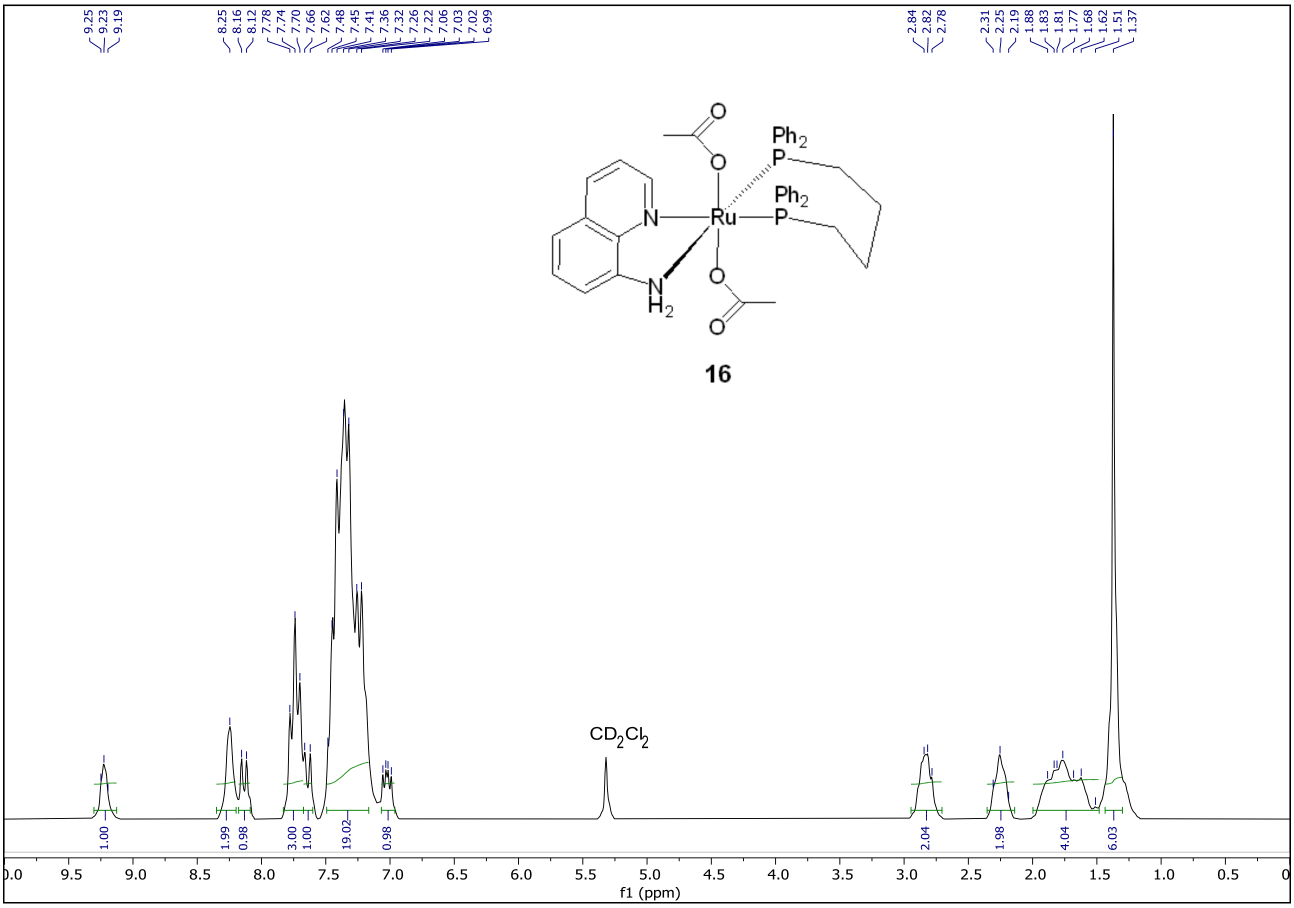

Figure S104. ^{1}H NMR spectrum (200.1 MHz) of *trans*-[Ru(η^{1}-OAc)$_2$(dppb)(8-aminoquinoline)] (**16**) in CD_2Cl_2 at 20 °C.

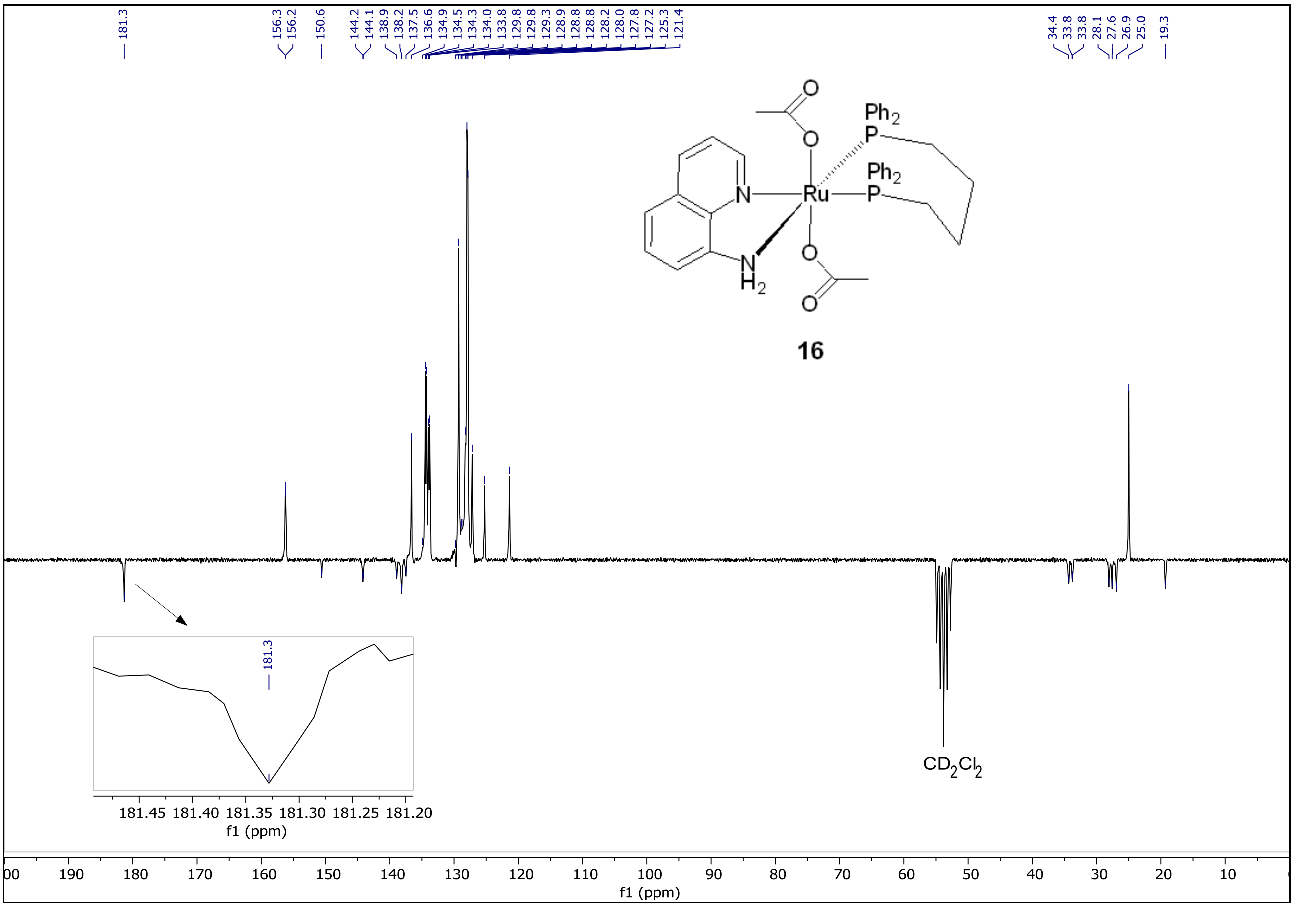

Figure S105. $^{13}C\{^1H\}$ PENDANT NMR spectrum (50.3 MHz) of *trans*-[Ru(η^1-OAc)$_2$(dppb)(8-aminoquinoline)] (**16**) in CD_2Cl_2 at 20 °C.

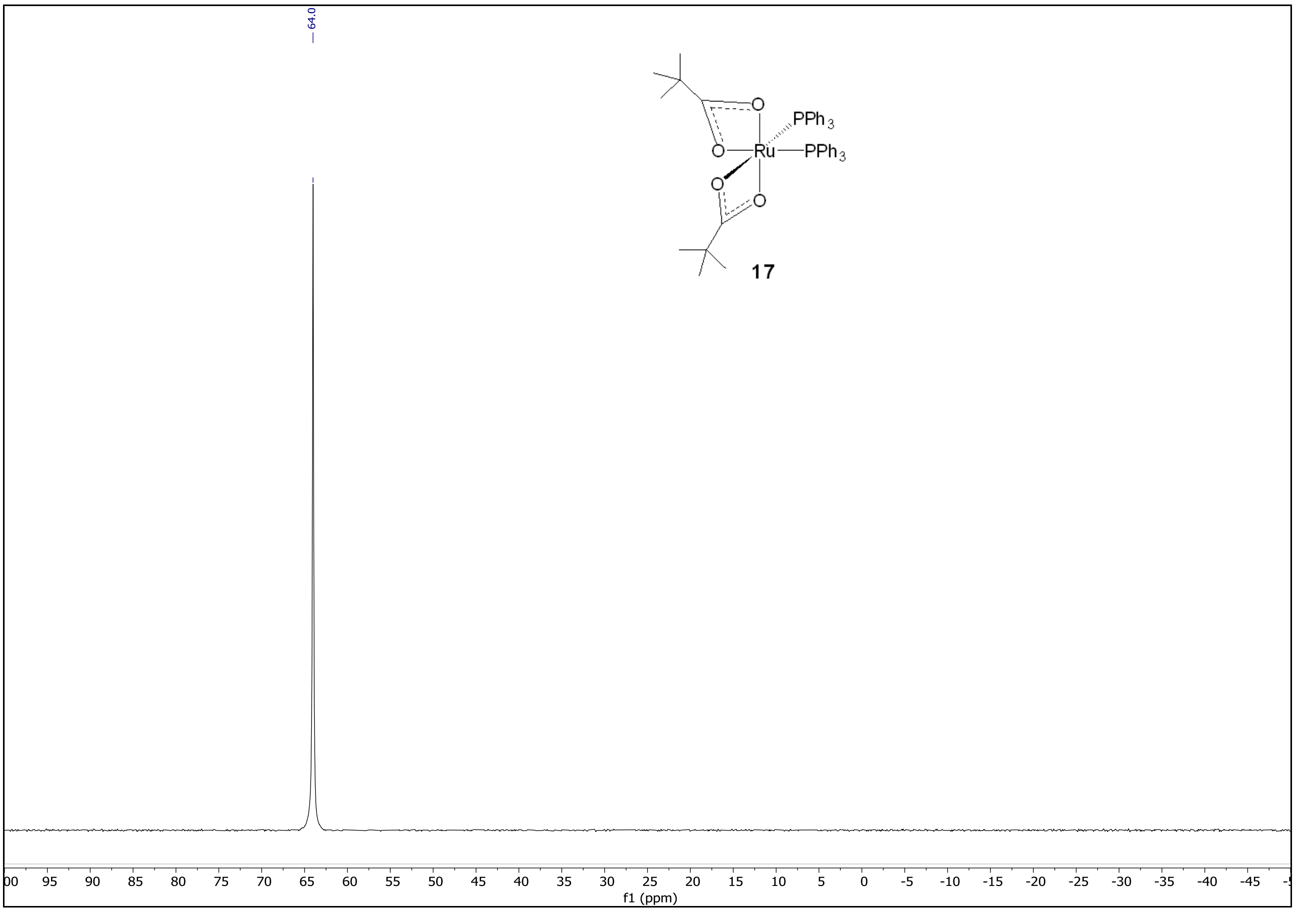

Figure S106. $^{31}P\{^{1}H\}$ NMR spectrum (81.0 MHz) of [Ru(η^2-OPiv)$_2$(PPh$_3$)$_2$] (**17**) in $CDCl_3$ at 20 °C.

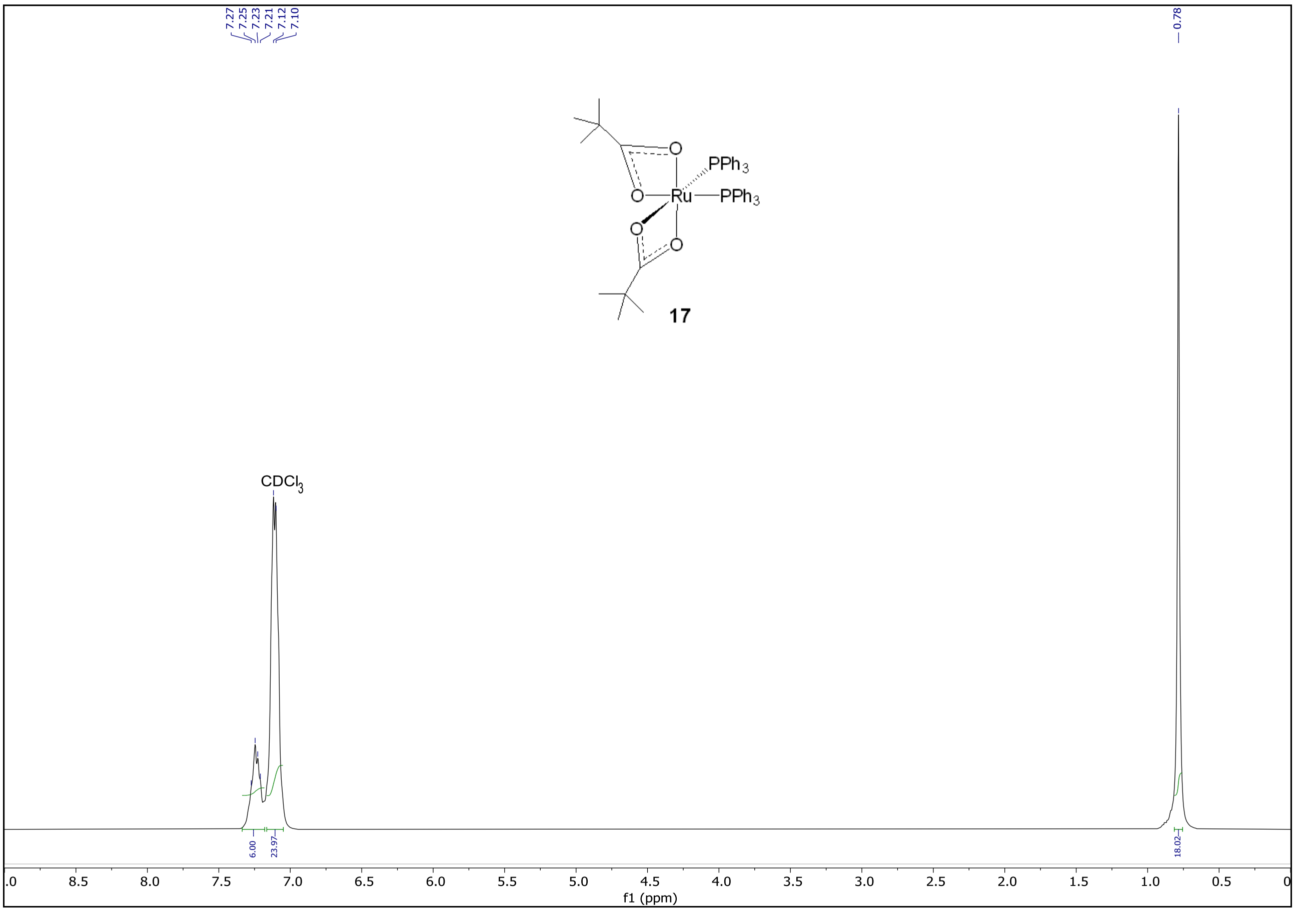

Figure S107. ^{1}H NMR spectrum (200.1 MHz) of [Ru(η^2-OPiv)$_2$(PPh$_3$)$_2$] (**17**) in $CDCl_3$ at 20 °C.

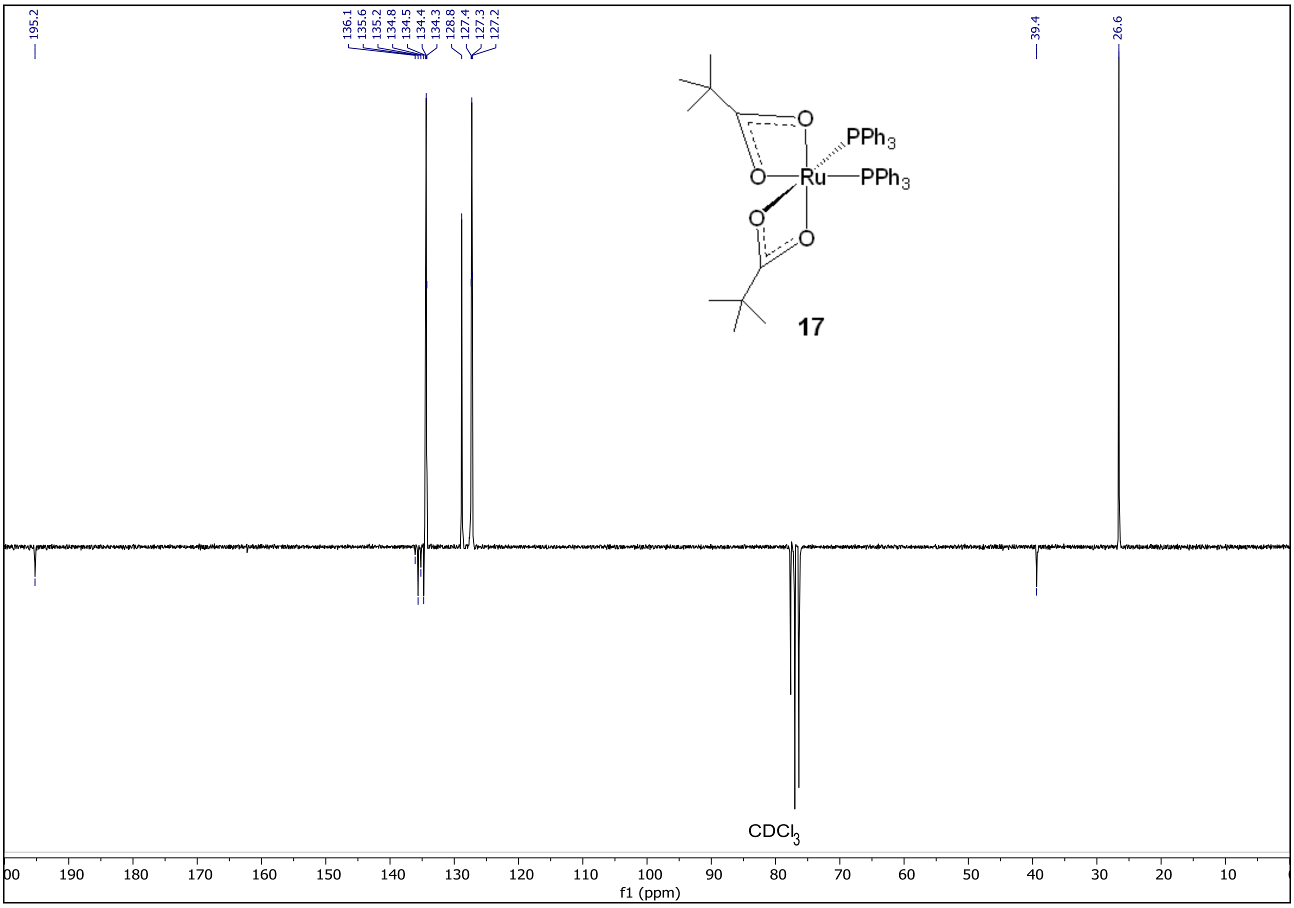

Figure S108. $^{13}C\{^{1}H\}$ PENDANT NMR spectrum (50.3 MHz) of [Ru(η^2-OPiv)$_2$(PPh$_3$)$_2$] (**17**) in $CDCl_3$ at 20 °C.

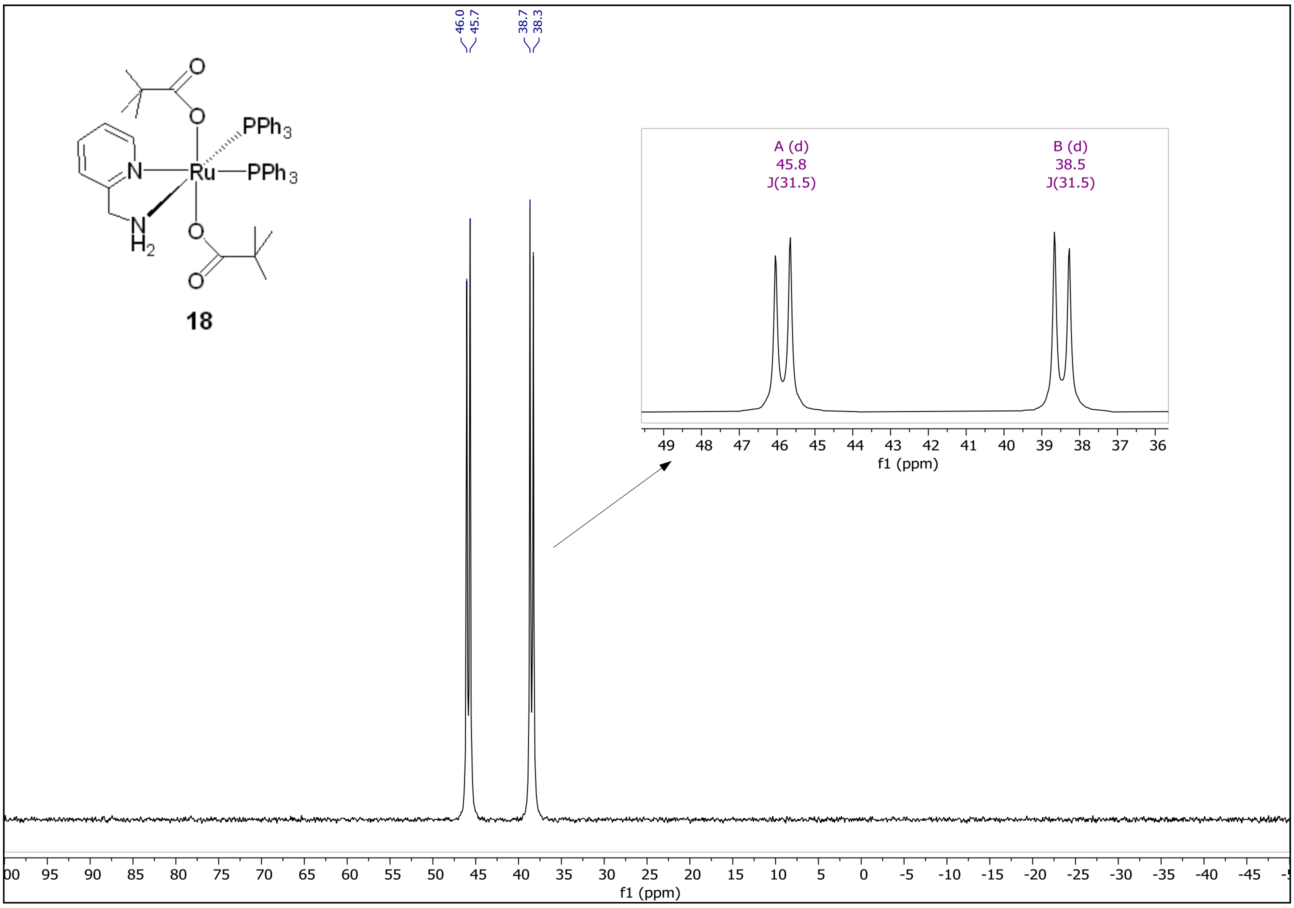

Figure S109. $^{31}P\{^{1}H\}$ NMR spectrum (81.0 MHz) of *trans*,*cis*-[Ru(η^1-OPiv)$_2$(PPh$_3$)$_2$(ampy)] (**18**) in $CDCl_3$ at 20 °C.

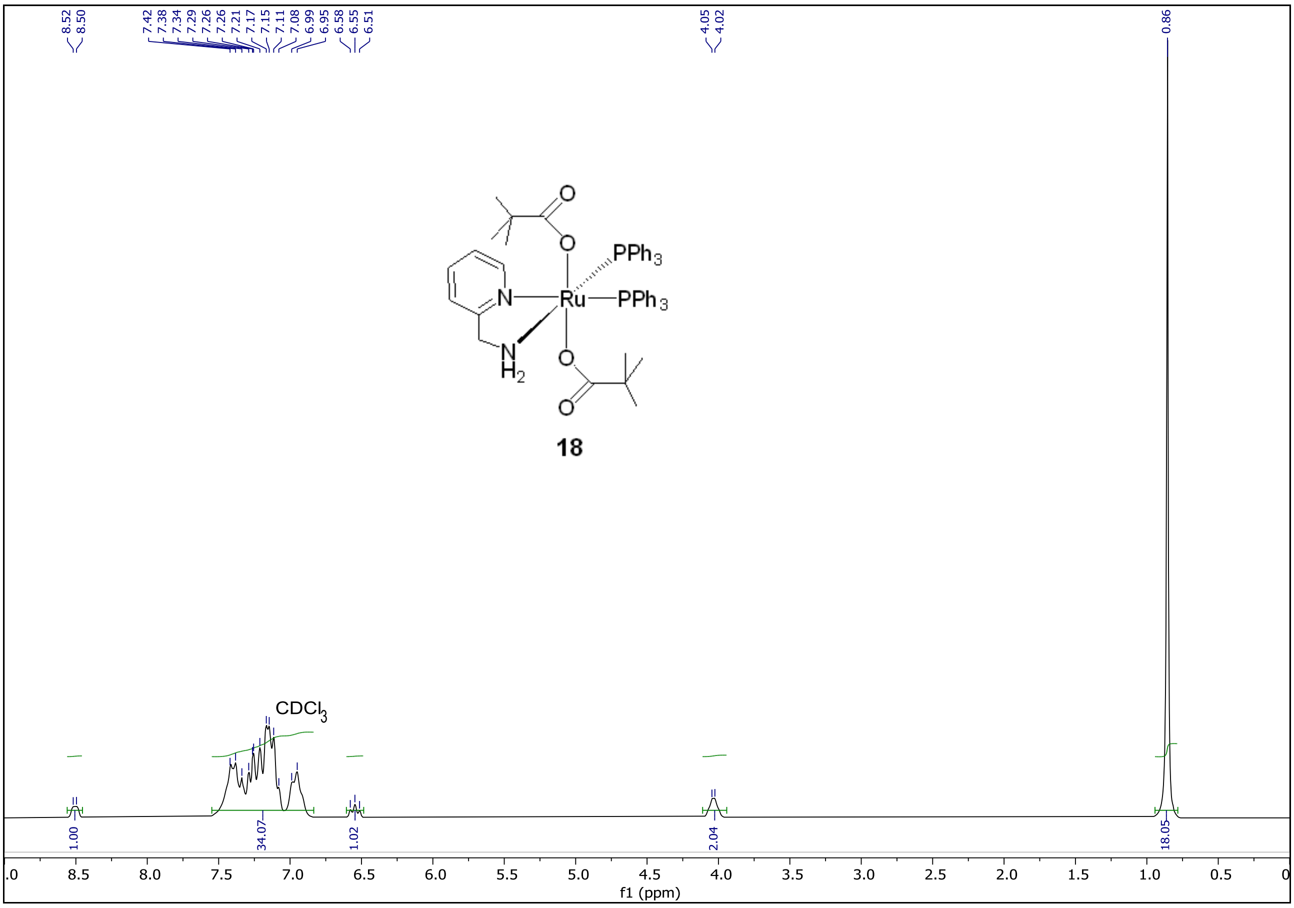

Figure S110. ^{1}H NMR spectrum (200.1 MHz) of *trans*,*cis*-[Ru(η^1-OPiv)$_2$(PPh$_3$)$_2$(ampy)] (**18**) in $CDCl_3$ at 20 °C.

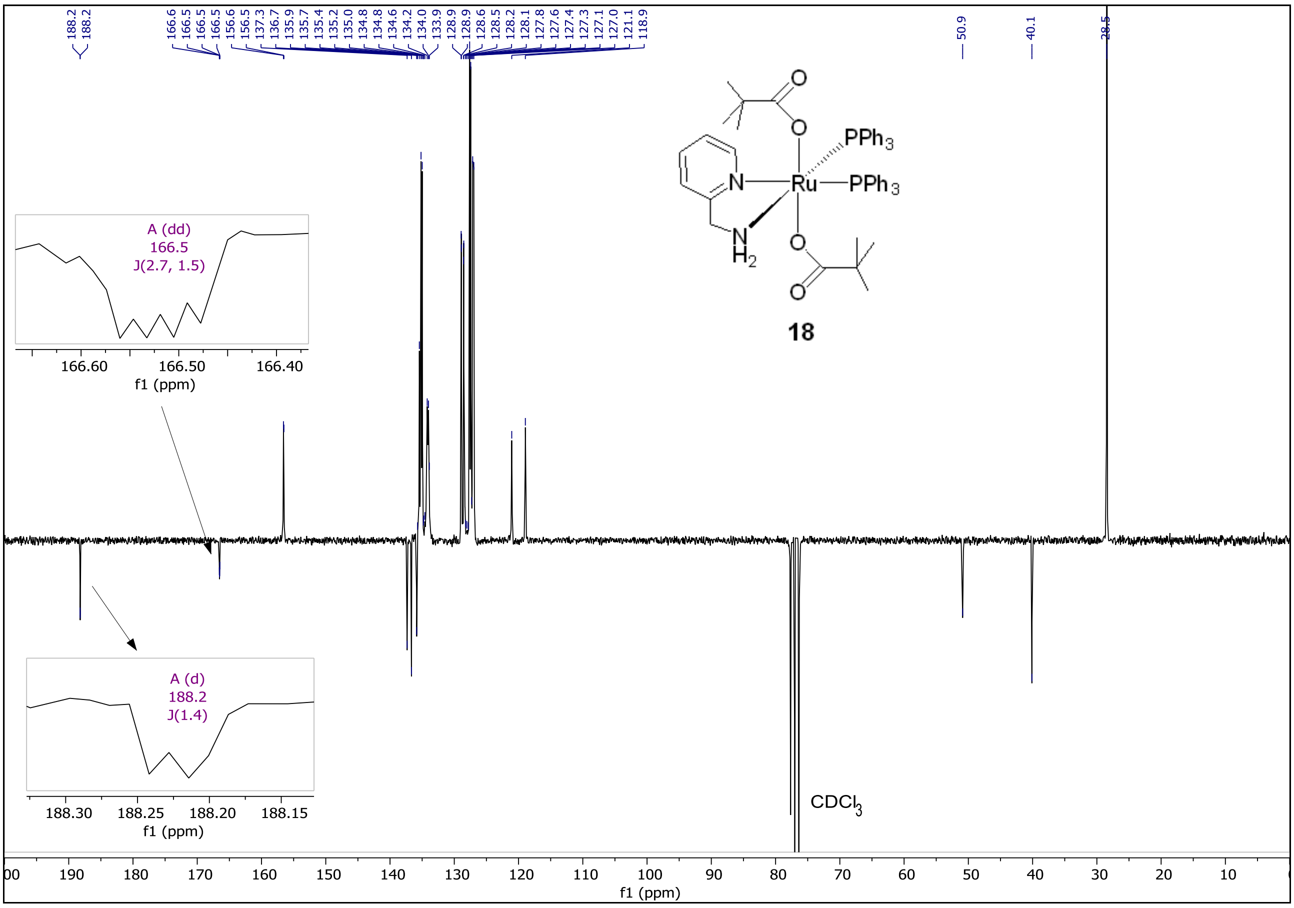

Figure S111. $^{13}C\{^{1}H\}$ PENDANT NMR spectrum (50.3 MHz) of *trans*,*cis*-[Ru(η^{1}-OPiv)$_2$(PPh$_3$)$_2$(ampy)] (**18**) in $CDCl_3$ at 20 °C.

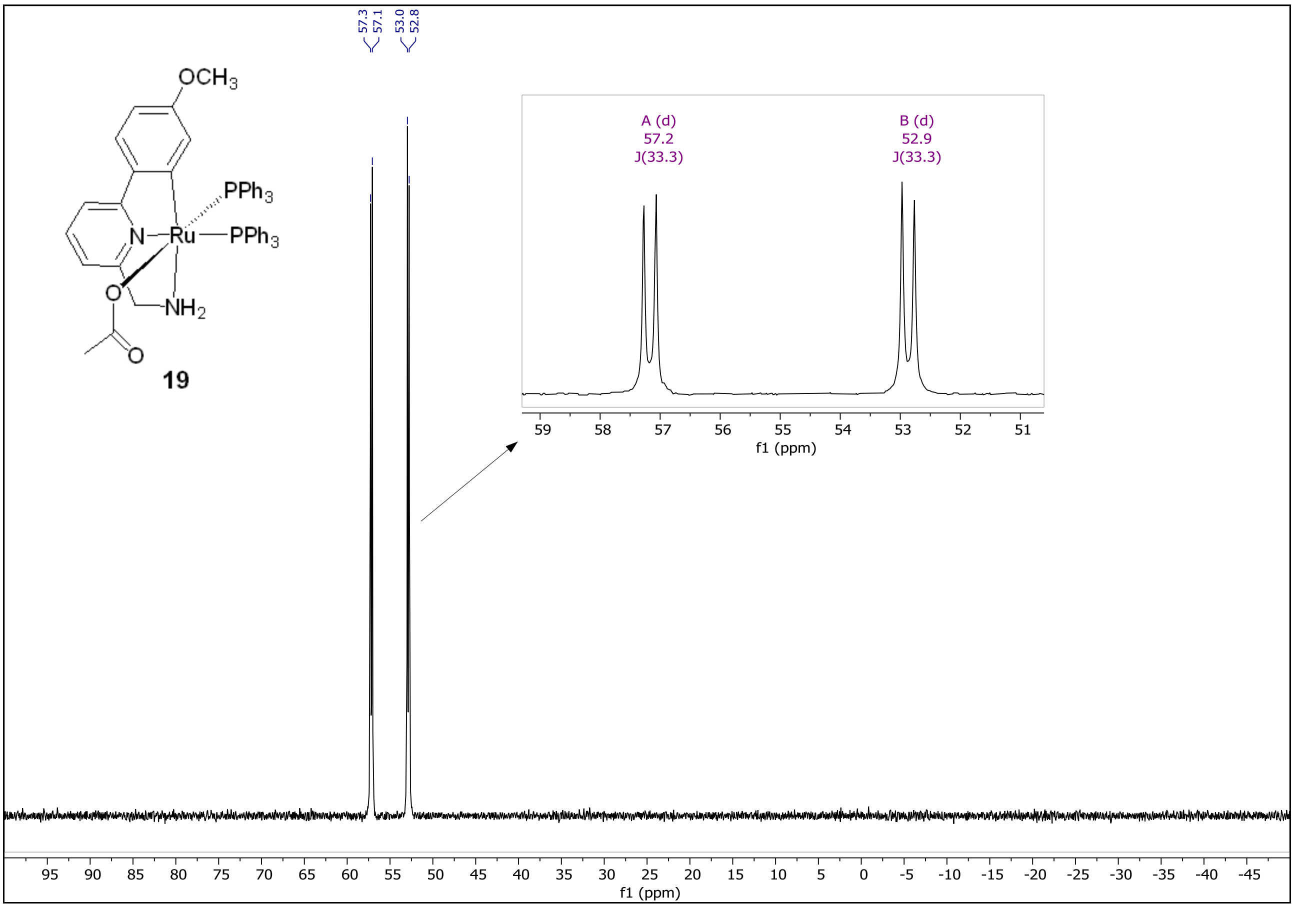

Figure S112. $^{31}P\{^{1}H\}$ NMR spectrum (162.0 MHz) of [Ru(η^1-OAc)(CNN^{OMe})(PPh_3)$_2$] (**19**) in CD_2Cl_2 at 25 °C.

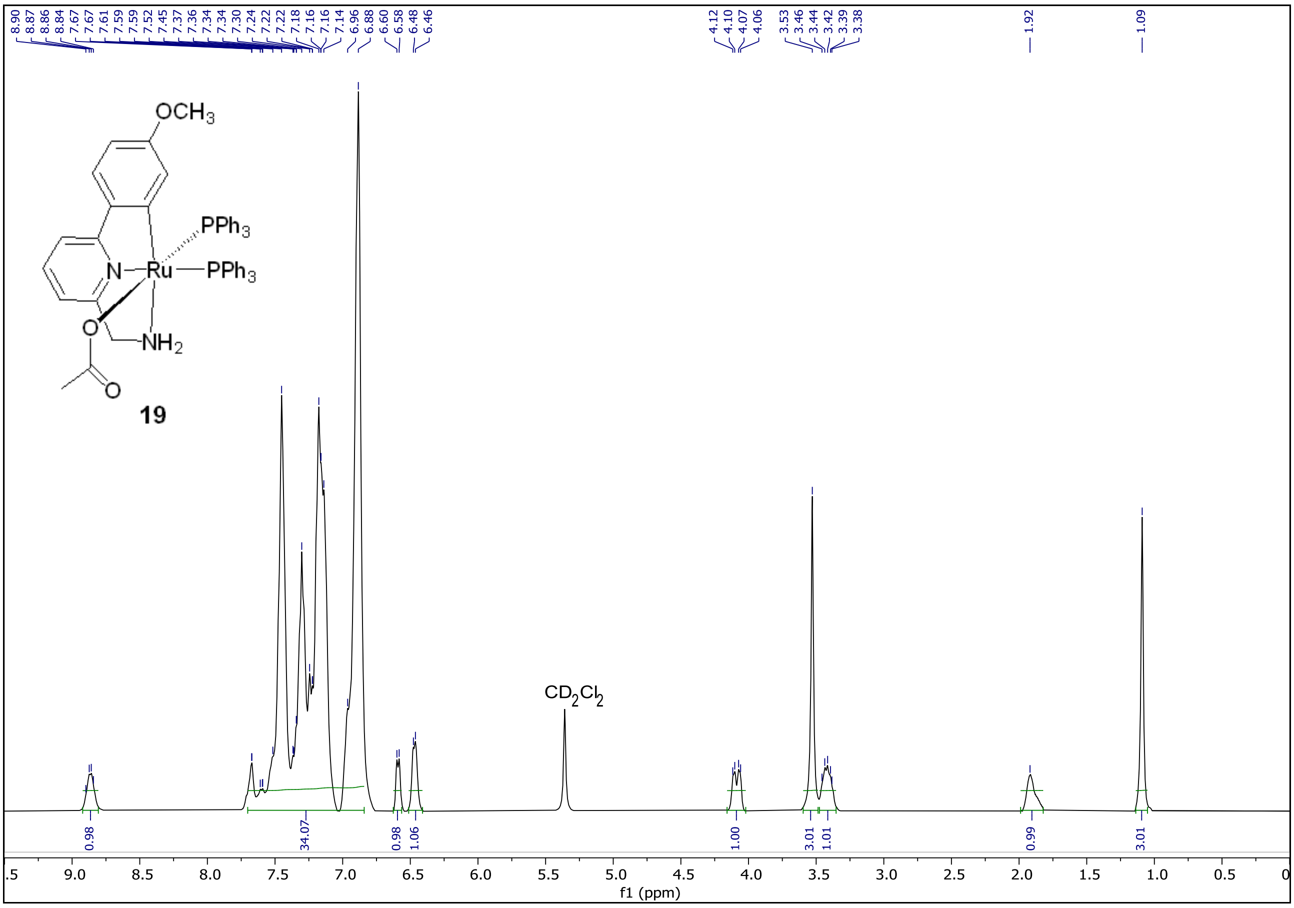

Figure S113. ^{1}H NMR spectrum (400.1 MHz) of [Ru(η^{1}-OAc)(CNN^{OMe})(PPh_3)$_2$] (**19**) in CD_2Cl_2 at 25 °C.

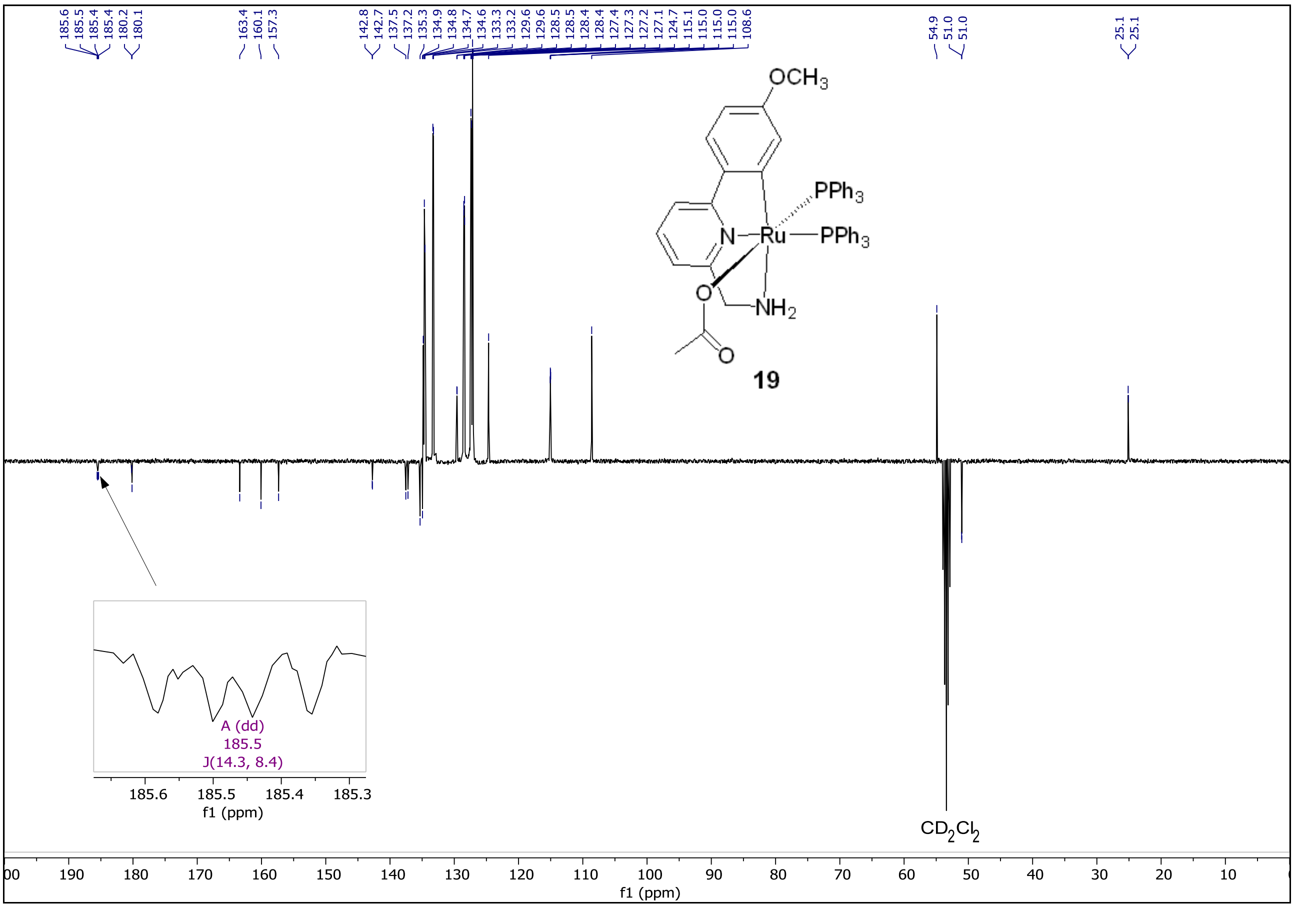

Figure S114. $^{13}C\{^{1}H\}$ DEPTQ NMR spectrum (100.6 MHz) of [Ru(η^1-OAc)(CNN^{OMe})($(PPh_3)_2$)] (**19**) in CD_2Cl_2 at 25 °C.

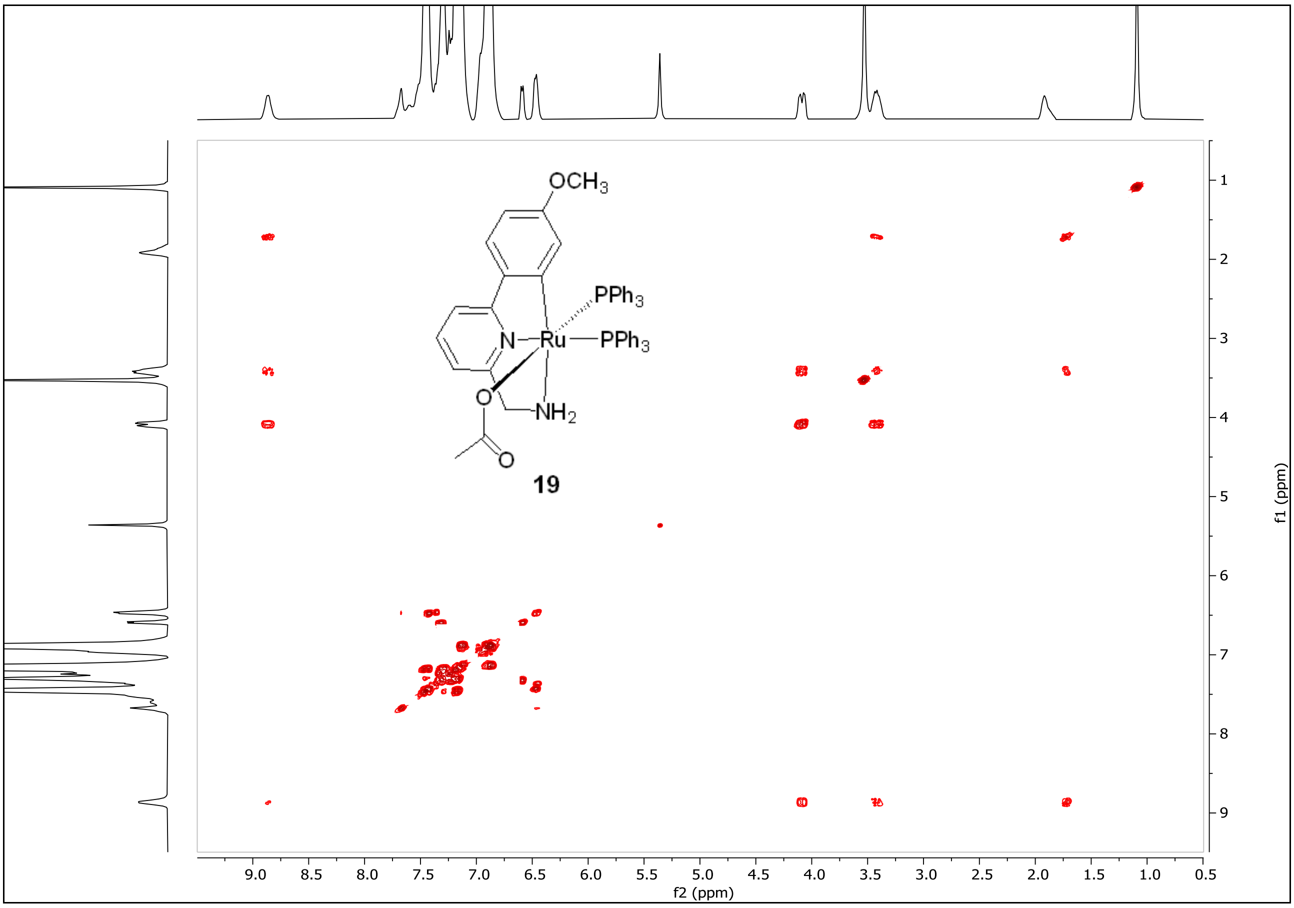

Figure S115. ^{1}H-^{1}H COSY 2D NMR spectrum (400.1 MHz) of [Ru(η^1-OAc)(CNN^{OMe})(PPh_3)$_2$] (**19**) in CD_2Cl_2 at 25 °C.

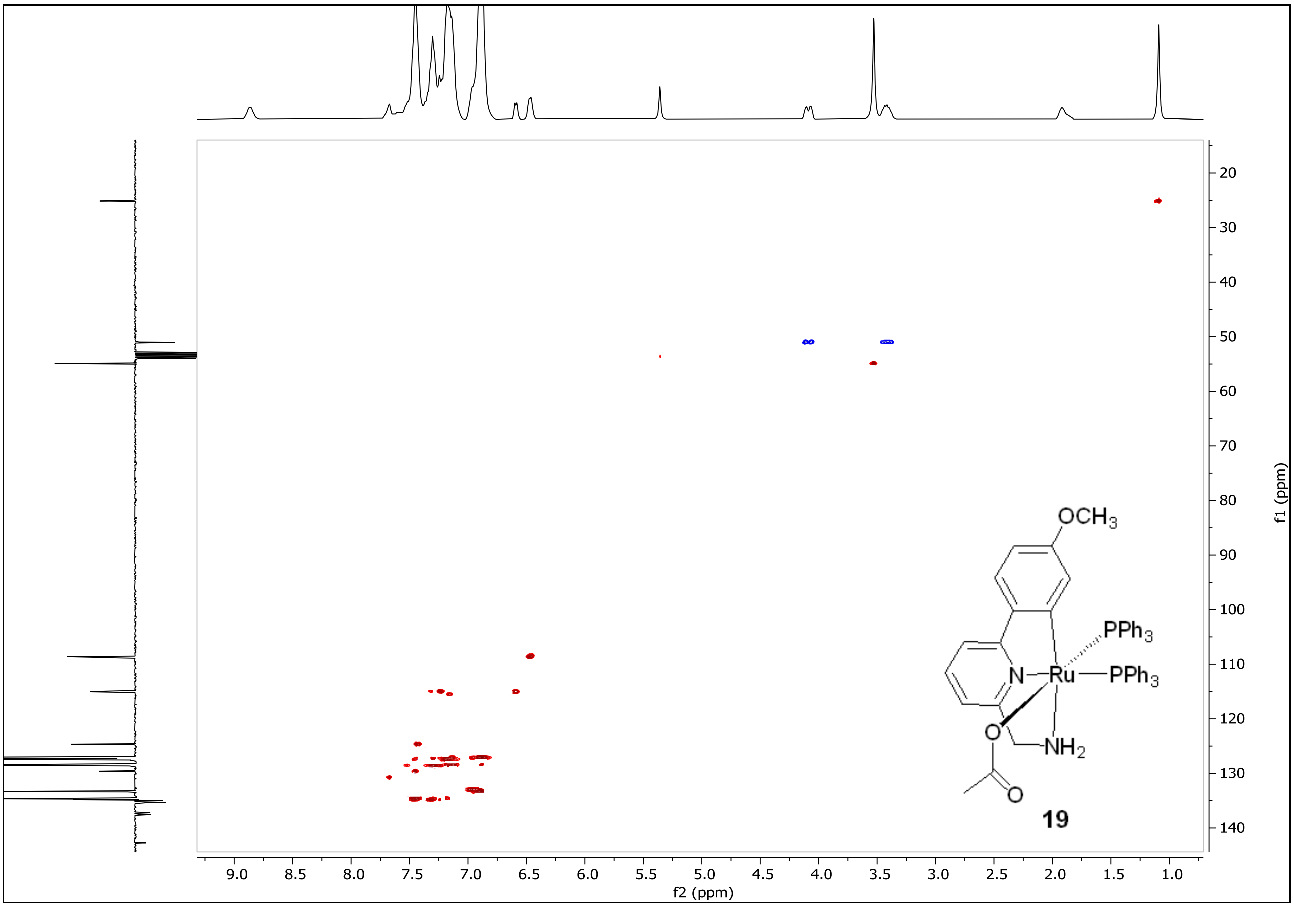

Figure S116. ^{1}H-^{13}C HSQC 2D NMR spectrum of [Ru(η^1-OAc)(CNN^{OMe})(PPh_3)$_2$] (**19**) in CD_2Cl_2 at 25 °C.

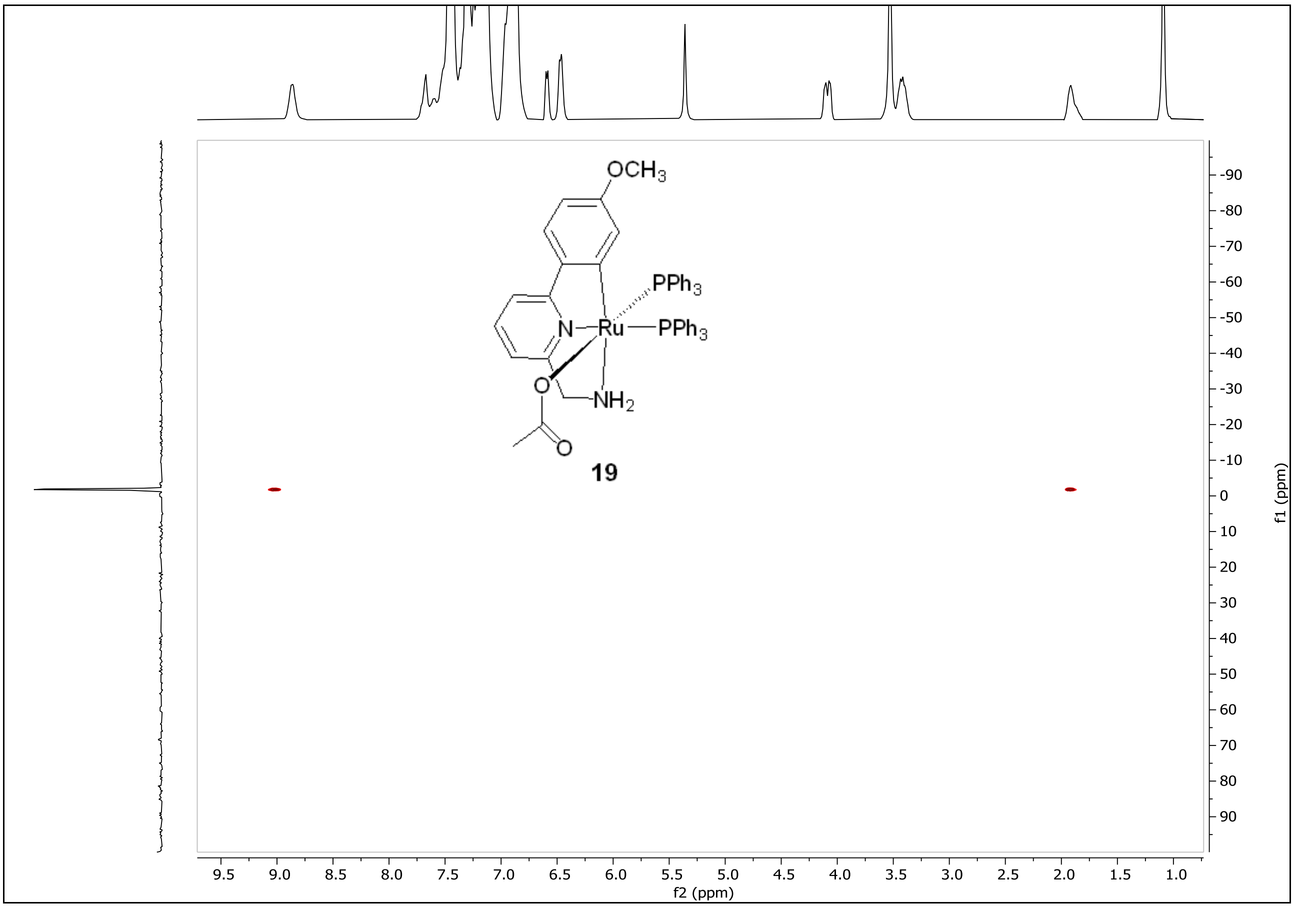

Figure S117. ^{1}H-^{15}N HSQC 2D NMR spectrum of [Ru(η^1-OAc)(CNN^{OMe})(PPh_3)$_2$] (**19**) in CD_2Cl_2 at 25 °C.

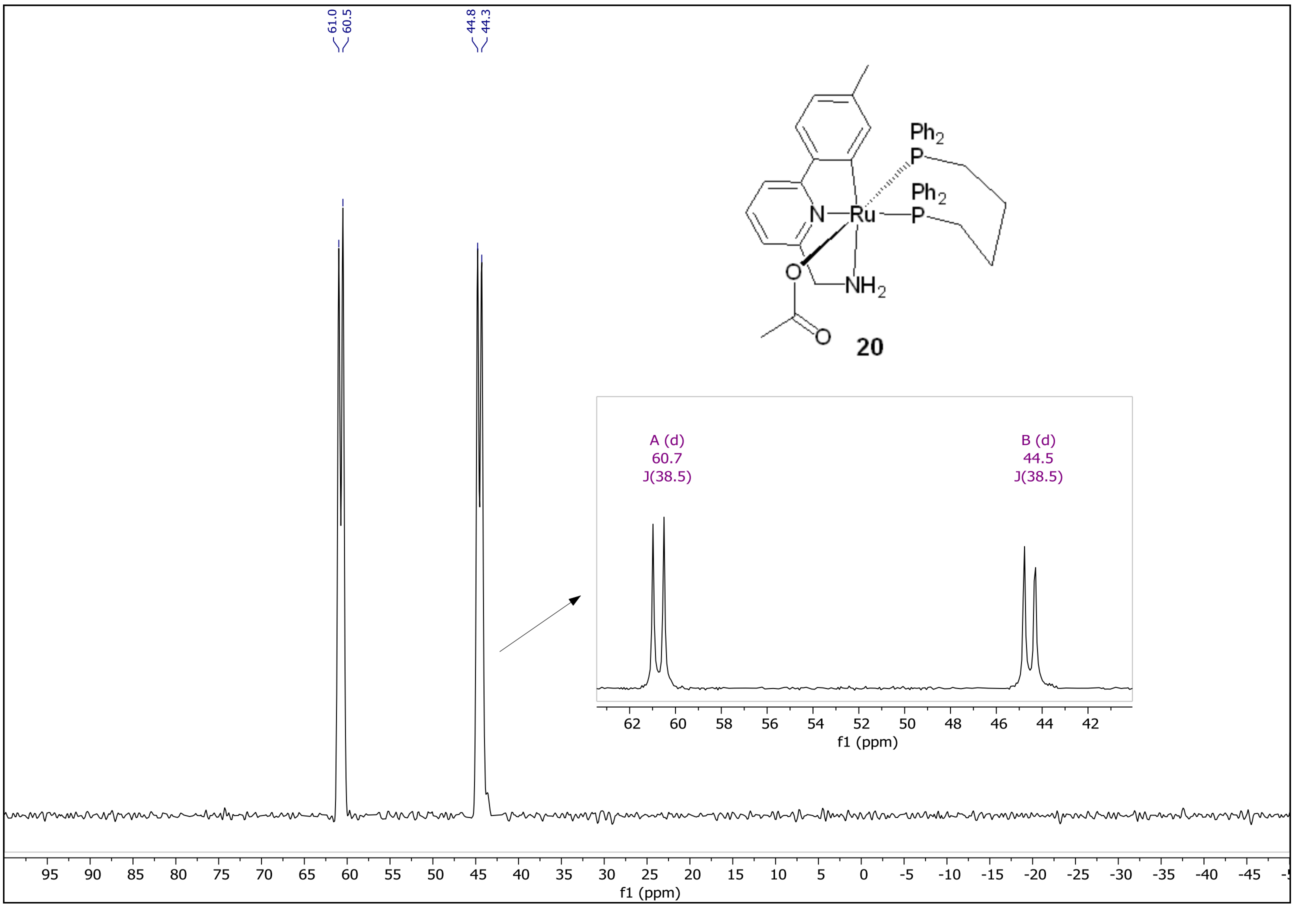

Figure S118. $^{31}P\{^{1}H\}$ NMR spectrum (81.0 MHz) of [Ru(η^1-OAc)(CNN)(dppb)] (**20**) in toluene-d^8 at 20 °C.

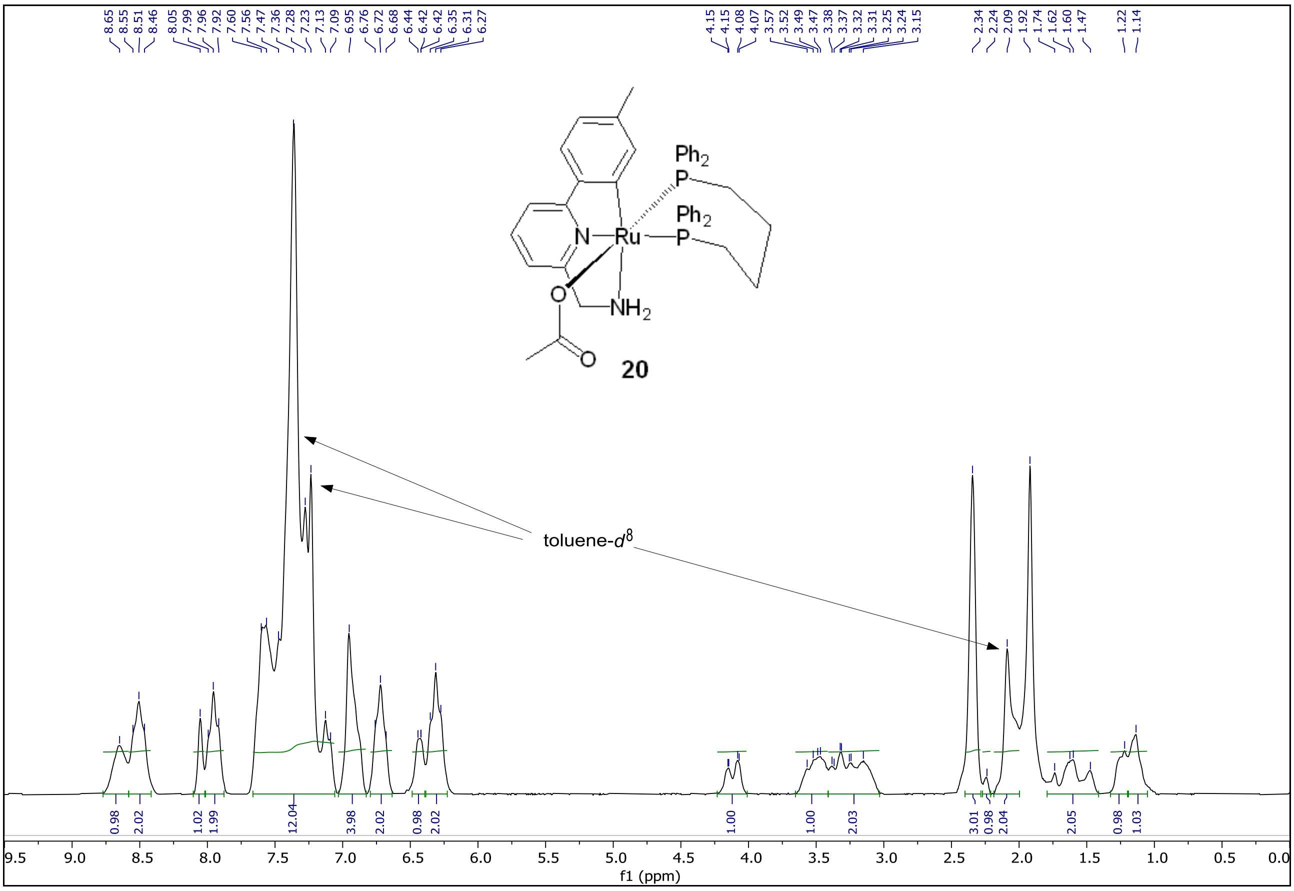

Figure S119. ^{1}H NMR spectrum (200.1 MHz) of [Ru(η^{1}-OAc)(CNN)(dppb)] (**20**) in toluene-d^{8} at 20 °C.

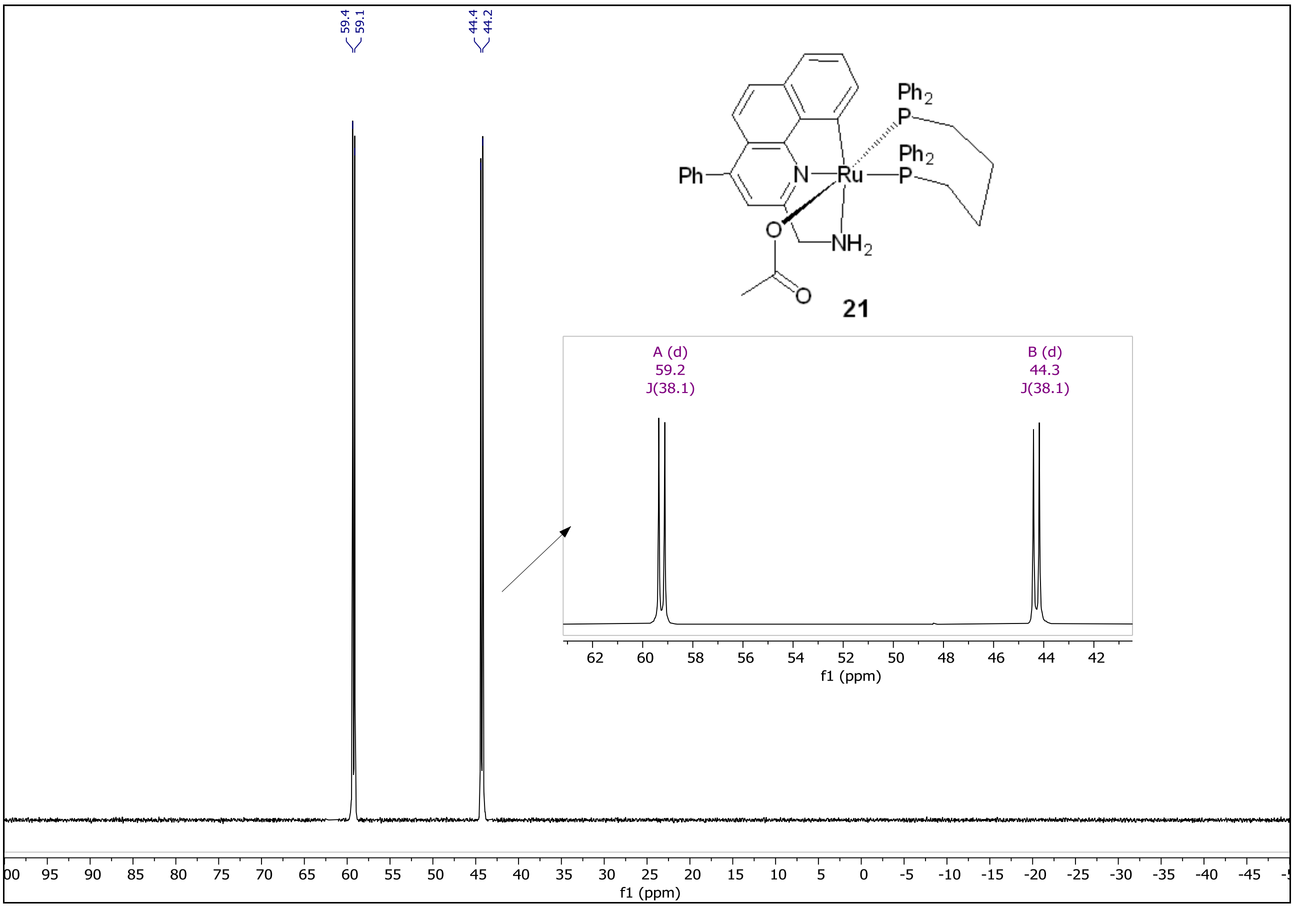

Figure S120. $^{31}P\{^1H\}$ NMR spectrum (162.0 MHz) of [Ru(η^1-OAc)(AMBQ^{Ph})(dppb)] (**21**) in CD_2Cl_2 at 25 °C.

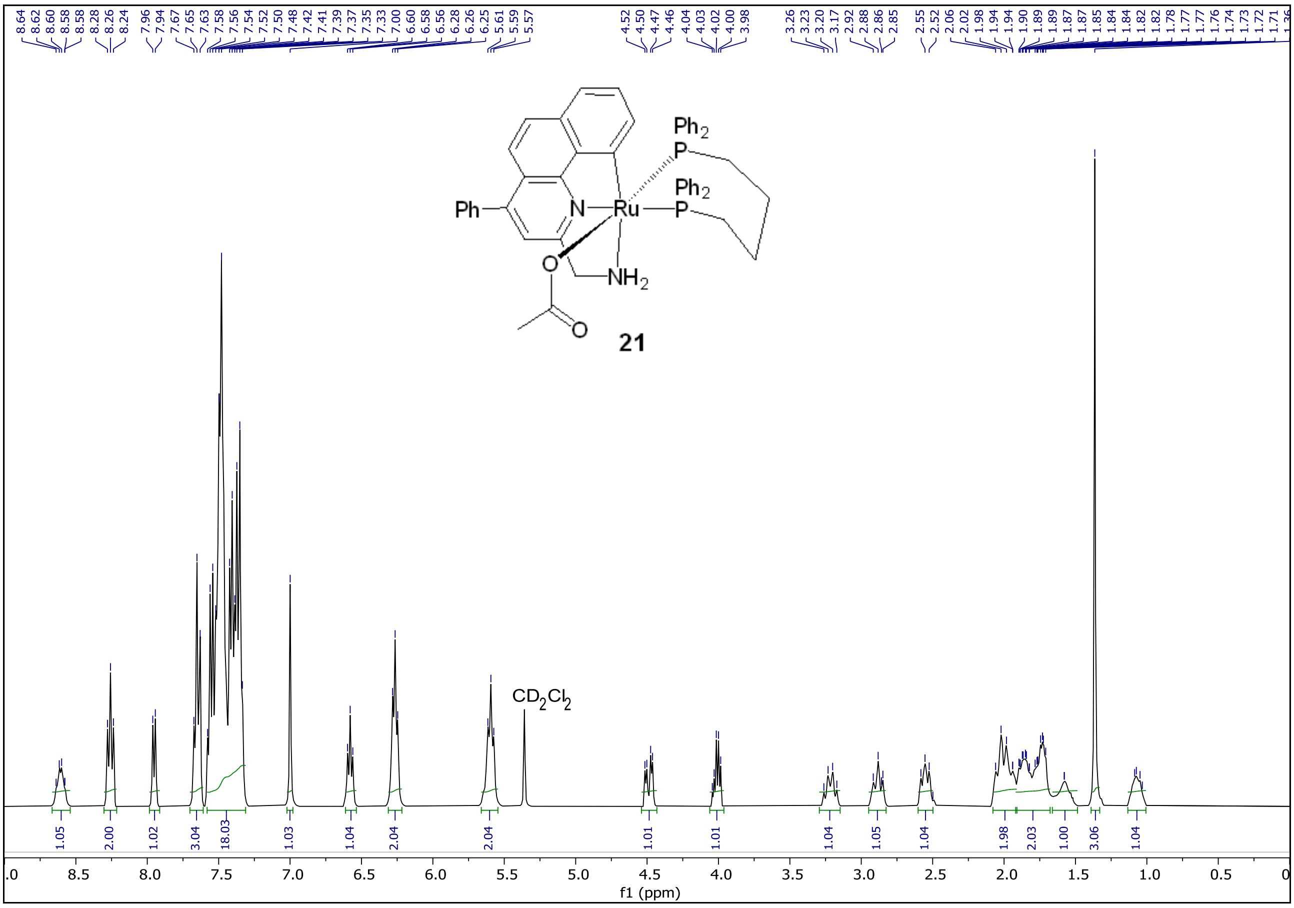

Figure S121. ^{1}H NMR spectrum (400.1 MHz) of [Ru(η^1-OAc)(AMBQ^{Ph})(dppb)] (**21**) in CD_2Cl_2 at 25 °C.

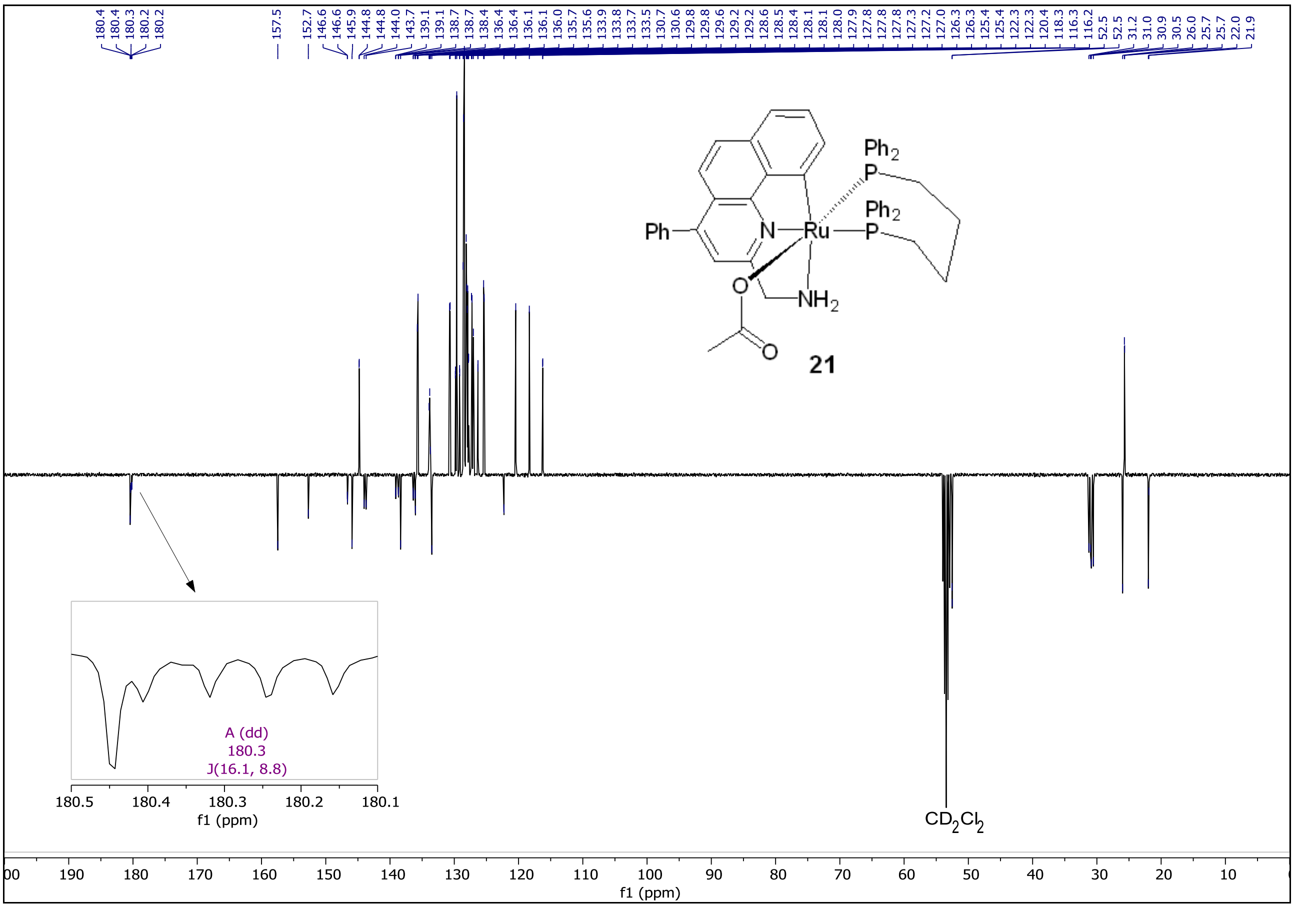

Figure S122. $^{13}C\{^1H\}$ DEPTQ NMR spectrum (100.6 MHz) of [Ru(η^1-OAc)(AMBQ^{Ph})(dppb)] (**21**) in CD_2Cl_2 at 25 °C.

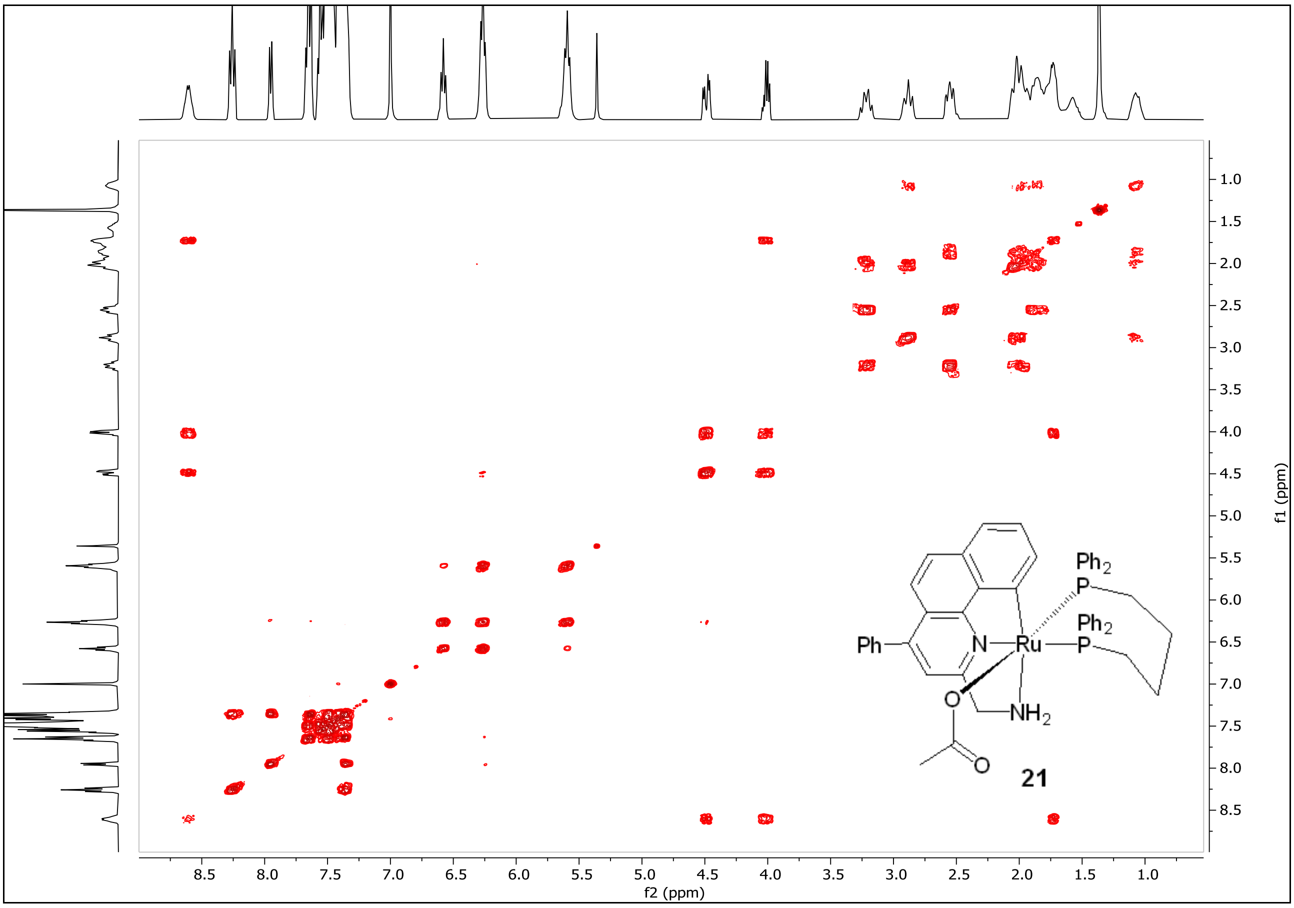

Figure S123. ^{1}H-^{1}H COSY 2D NMR spectrum (400.1 MHz) of [Ru(η^{1}-OAc)(AMBQ^{Ph})(dppb)] (**21**) in CD_2Cl_2 at 25 °C.

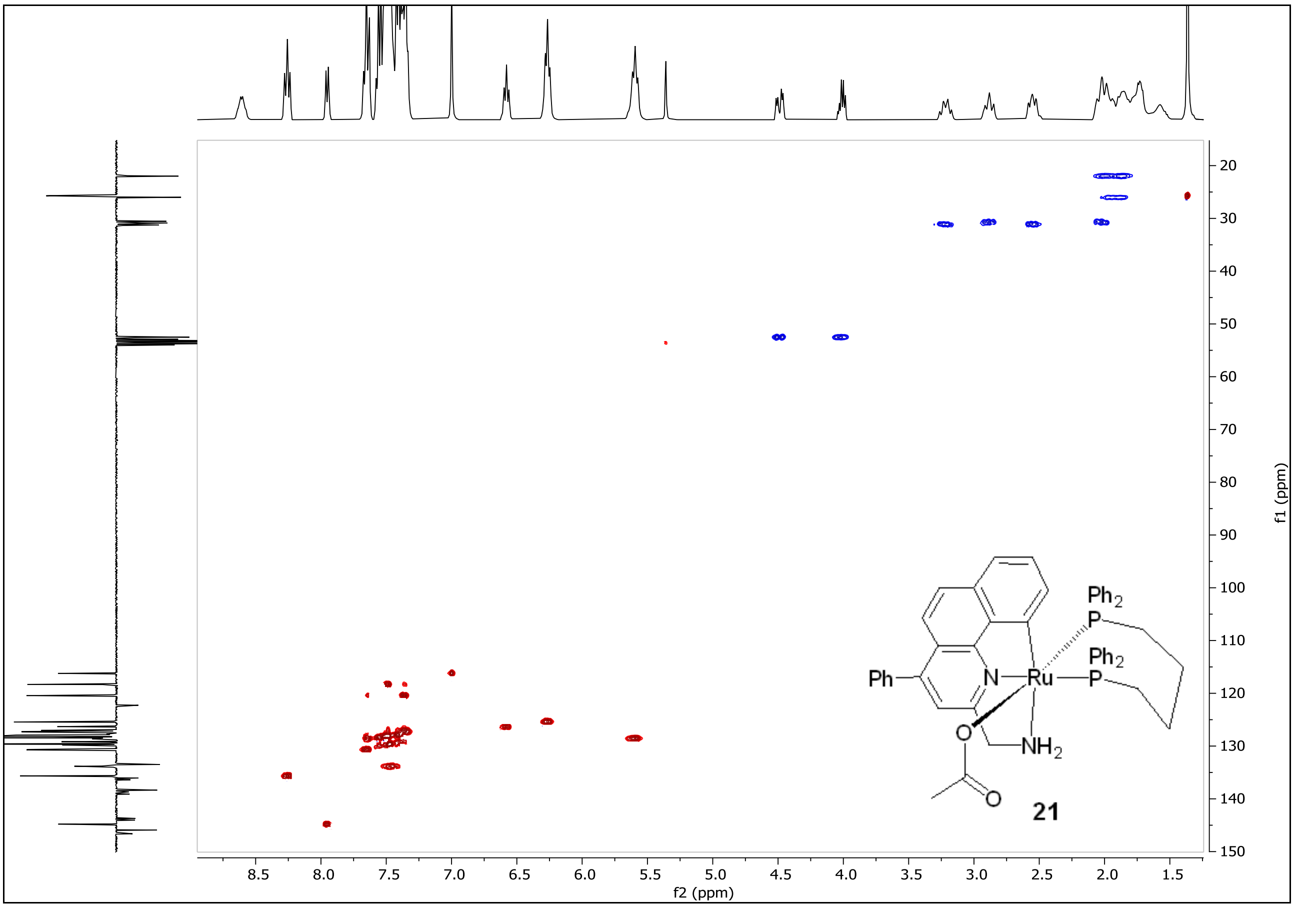

Figure S124. ^{1}H-^{13}C HSQC 2D NMR spectrum of [Ru(η^1-OAc)(AMBQ^{Ph})(dppb)] (**21**) in CD_2Cl_2 at 25 °C.

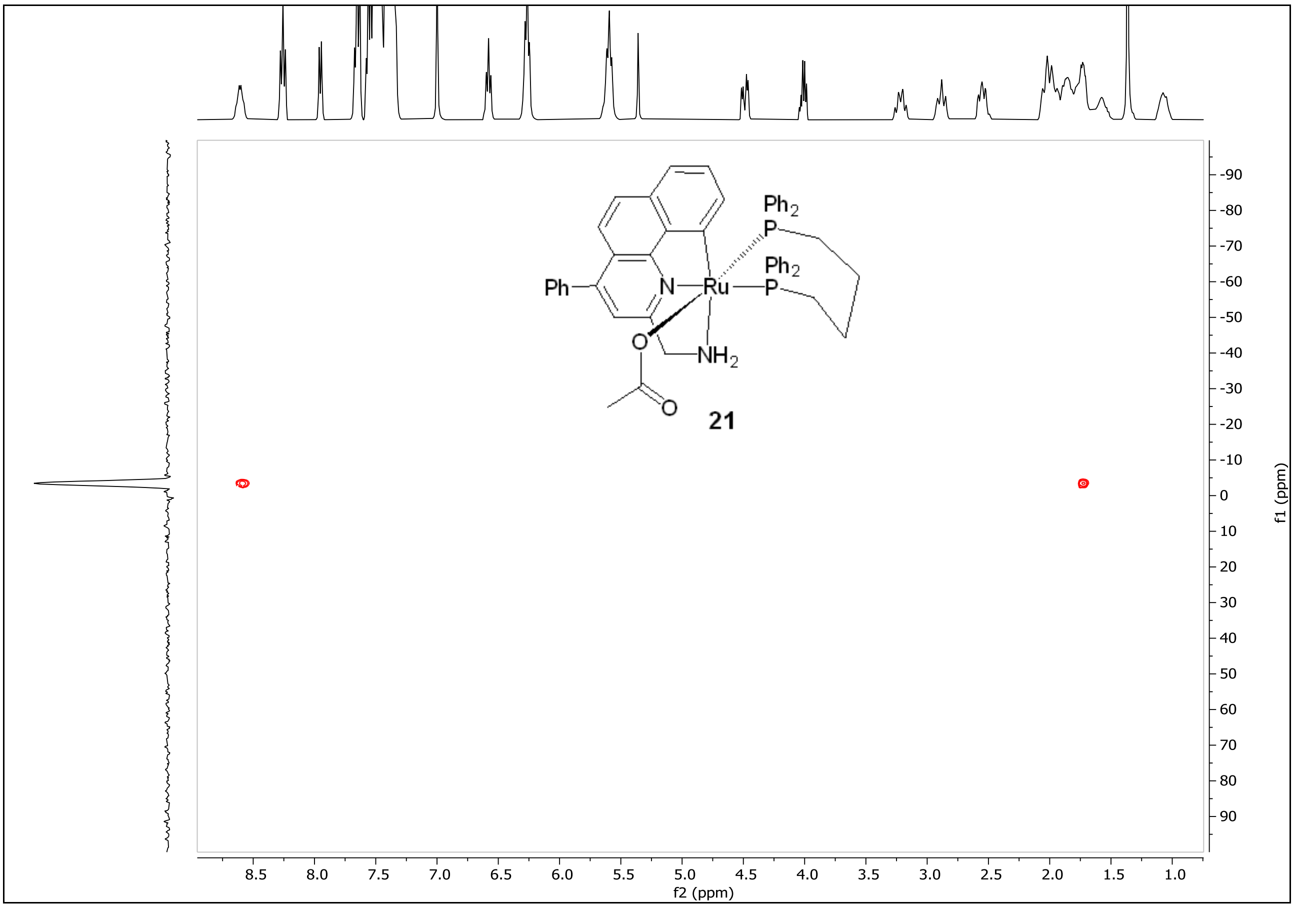

Figure S125. ^{1}H-^{15}N HSQC 2D NMR spectrum of [Ru(η^1-OAc)(AMBQ^{Ph})(dppb)] (**21**) in CD_2Cl_2 at 25 °C.

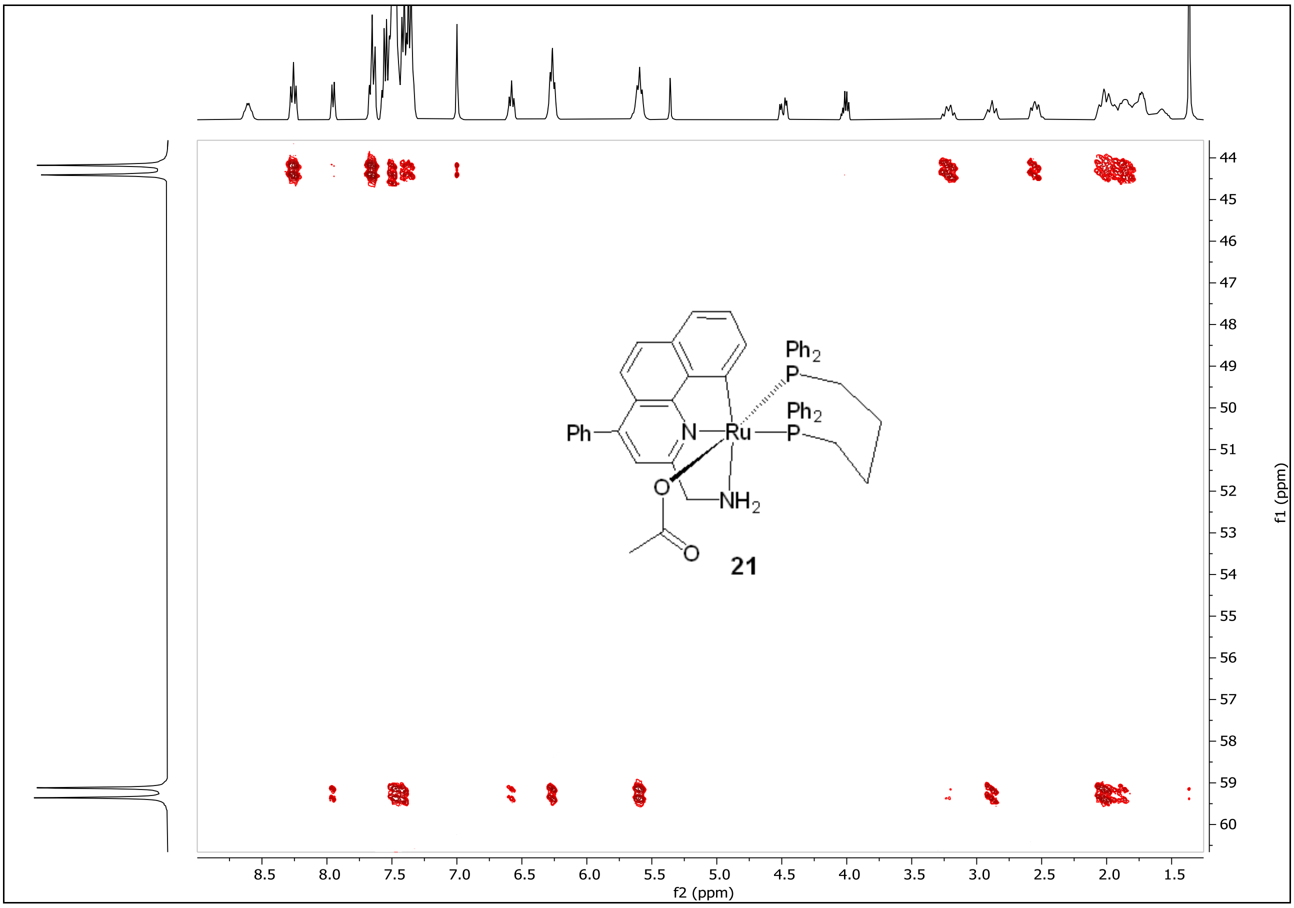

Figure S126. ^{1}H-^{31}P HMBC 2D NMR spectrum of [Ru(η^1-OAc)(AMBQ^{Ph})(dppb)] (**21**) in CD_2Cl_2 at 25 °C.

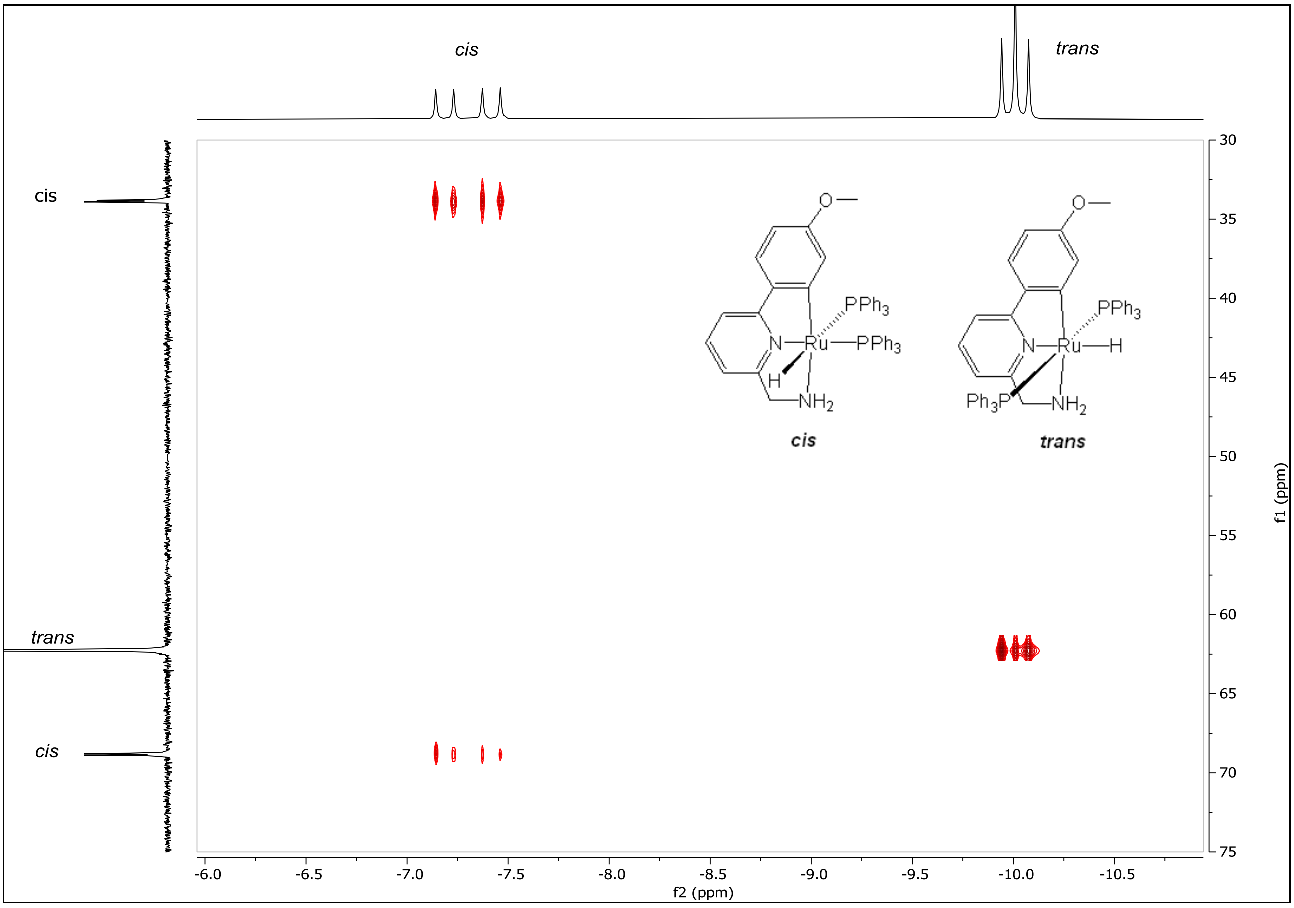

Figure S127. ^{1}H-^{31}P HMBC 2D NMR spectrum (hydridic region) of the mixture 2.8:1 of *trans*- and *cis*-[RuH(PPh_3)$_2$(CNN^{OMe})] in iPrOH/toluene-d^8 at 25 °C.

Preparation of the mixture of trans- and cis-[RuH(PPh_3)$_2$(CNN^{OMe})]

Complex **19** (10.0 mg, 0.011 mmol) was suspended in toluene-d^8 (250 µL), and 223 µL of a solution of NaO*i*Pr (0.1 M, 0.022 mmol) in 2-propanol was added. The mixture was stirred at room temperature for 1 h, until an orange solution containing *trans*- and *cis*-[RuH(PPh_3)$_2$(CNN^{OMe})] was formed, and submitted to NMR analysis.

Single Crystal X-Ray Structure Determination of Compound 7 (CCDC 2058063).

General Data

Data were collected on a single crystal x-ray diffractometer equipped with a CCD detector (Bruker APEX II), a fine focused sealed tube with MoK_{α} radiation (λ = 0.71073 Å) and a graphite monochromator using the APEX 3 software package.[1] Measurements were performed on single crystals coated with perfluorinated ether. The crystals were fixed on top of a kapton micro sampler and frozen under a stream of cold nitrogen. A matrix scan was used to determine the initial lattice parameters. Reflections were corrected for Lorentz and polarisation effects, scan speed, and background using SAINT.[2] Absorption correction, including odd and even ordered spherical harmonics was performed using SADABS.[3] Space group assignment was based upon systematic absences, E statistics, and successful refinement of the structures. The structures were solved using SHELXT with the aid of successive difference Fourier maps, and were refined against all data using SHELXL in conjunction with SHELXLE.[4,5,6] The positions of the nitrogen bound hydrogen atoms were refined using distance restraints (DFIX) and fixed displacement parameters ($U_{iso(H)}$ = -1.2·$U_{eq(C)}$). The other hydrogen atoms were calculated in ideal positions as follows: Methyl hydrogen atoms were refined as part of rigid rotating groups with a C–H distance of 0.98 Å and $U_{iso(H)}$ = 1.5·$U_{eq(C)}$. Other H atoms were placed in calculated positions and refined using a riding model, with methylene and aromatic C–H distances of 0.99 Å and 0.95 Å, respectively, and other C–H distances of 1.00 Å, all with $U_{iso(H)}$ = 1.2·$U_{eq(C)}$. Non-hydrogen atoms were refined with anisotropic displacement parameters. Full-matrix least-squares refinements were carried out by minimizing $\Sigma w(F_o^2 - F_c^2)^2$ with the SHELXL weighting scheme.[4] Neutral atom scattering factors for all atoms and anomalous dispersion corrections for the non-hydrogen atoms were taken from *International Tables for Crystallography*.[7] A split layer refinement was used for disordered groups and additional restraints on geometries and anisotropic displacement parameters were used, if necessary. The unit cell contains 16 highly disordered molecules of diethyl ether which were treated as a diffuse contribution to the overall scattering without specific atom positions by SQUEEZE/PLATON.[8] Images of the crystal structures were generated with Mercury.[9] CCDC 2058063 contains the supplementary crystallographic data for this paper. These data are provided free of charge by The Cambridge Crystallographic Data Centre.

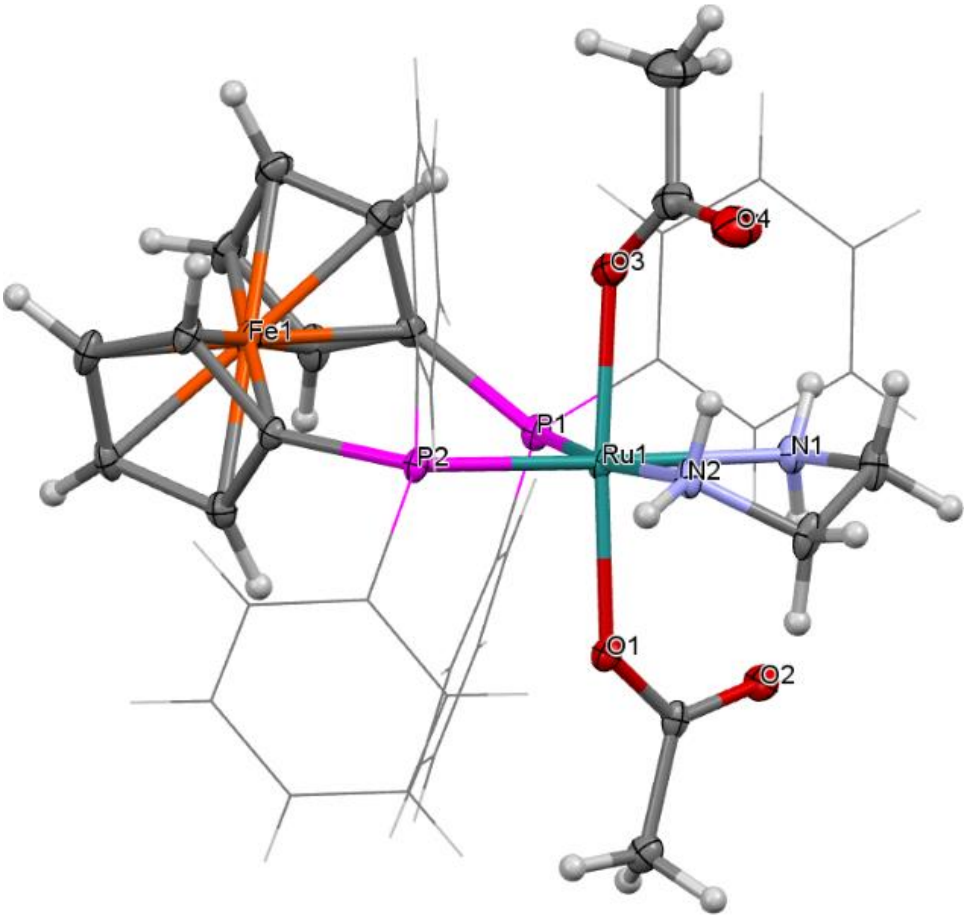

Figure S128. Molecular structure of complex **7** (CCDC 2058063) as determined by single crystal X-ray diffraction. Ellipsoids are displayed at the 50% probability level and the phenyl groups are simplified as wireframes for clarity (as well as disorder of one phenyl group is not shown).

Single Crystal X-Ray Structure Determination of Compound 7 (CCDC 2058063).

Detailed Crystallographic Data.

Diffractometer operator:	A. Pöthig
Scanspeed	10 s per frame
Dx	50 mm
Frames:	1835 measured in 6 data sets
phi-scans with delta phi	0.5
omega-scans with delta omega	0.5

Crystal Data:

Compound	*trans*-[Ru(η^1-OAc)$_2$(dppf)(en)] (**7**)	
Chemical formula of the crystal	$C_{41}H_{44}Cl_2FeN_2O_4P_2Ru$	
Formula weight (M_r)	918.54	
Temperature	123(2) K	
Wavelength	0.71073 Å	
Crystal habit	clear intense yellow fragment	
Crystal size	0.148 x 0.347 x 0.447 mm	
Crystal system	monoclinic	
Space group	*C* 1 2/*c* 1	
Unit cell dimensions	a = 32.5993(9) Å	α = 90°
	b = 12.6190(3) Å	β = 118.876(2)°
	c = 25.5070(7) Å	γ = 90°
Volume	9188.2(4) Å^3	
Z	8	
Density (calculated)	1.328 g/cm^3	
Absorption coefficient	0.868 mm^{-1}	
F(000)	3760	

Data collection and structure refinement:

Diffractometer	Bruker Kappa APEX II CCD
Radiation source	fine-focus tube, Mo
Theta range for data collection	1.82 to 25.42°
Index ranges	-39<=h<=39, -15<=k<=15, -30<=1<=30
Reflections collected	55246
Independent reflections	8472 [R(int) = 0.0925]
Coverage of independent reflections	99.8%
Absorption correction	multi-scan
Max. and min. transmission	0.8820 and 0.6980
Function minimized	$\Sigma w(F_o^2 + 2F_c^2)^2$
Data / restraints / parameters	8472/ 49 / 520
Goodness-of-fit on F^2	1.043
Δ/σ_{max}	0.001
Final R indices	6002 data; I > 2σ(I) R1 = 0.0473, wR2 = 0.1045
	all data R1 = 0.0781, wR2 = 0.1159
Weighting scheme	$w = 1/[\sigma^2(F_o^2) + (0.508P)^2 + 7.8216P]$
	where $P = (F_o^2 + 2F_c^2)/3$
Largest diff. peak and hole	0.921 and -0.691 eÅ^{-3}
R.M.S. deviation from mean	0.098 eÅ^{-3}

References.

(1) *APEX suite of crystallographic software*, APEX 3, Version 2015.5-2, Bruker AXS Inc., Madison, Wisconsin, USA, 2015.

(2) *SAINT*, Version 8.38A, Bruker AXS Inc., Madison, Wisconsin, USA, 2017.

(3) *SADABS*, Version 2016/2, Bruker AXS Inc., Madison, Wisconsin, USA, 2016.

(4) Sheldrick, G. M., SHELXT - Integrated space-group and crystal-structure determination. *Acta Cryst.* **2015**, *A71*, 3-8.

(5) Sheldrick, G. M., Crystal structure refinement with SHELXL. *Acta Cryst.* **2015**, *C71*, 3-8.

(6) Hubschle, C. B.; Sheldrick, G. M.; Dittrich, B., ShelXle: a Qt graphical user interface for SHELXL. *J. Appl. Cryst.* **2011**, *44*, 1281-1284.

(7) *International Tables for Crystallography, Vol. C* (Ed.: A. J. Wilson), Kluwer Academic Publishers, Dordrecht, The Netherlands, **1992**, Tables 6.1.1.4 (pp. 500-502), 4.2.6.8 (pp. 219-222), and 4.2.4.2 (pp. 193-199).

(8) Spek, A. L., PLATON SQUEEZE: a tool for the calculation of the disordered solvent contribution to the calculated structure factors. *Acta Cryst.* **2015**, *C71*, 9-18.

(9) Macrae, C. F.; Bruno, I. J.; Chisholm, J. A.; Edgington, P. R.; McCabe, P.; Pidcock, E.; Rodriguez-Monge, L.; Taylor, R.; van de Streek, J.; Wood, P. A., Mercury CSD 2.0 - new features for the visualization and investigation of crystal structures. *J. Appl. Cryst.* **2008**, *41*, 466-470.

(10) Groom, C. R.; Bruno, I. J.; Lightfoot, M. P.; Ward, S. C., The Cambridge Structural Database. *Acta Cryst.* **2016**, *B72*, 171-179.
